# Supplementary material for: Chemoselective cycloisomerization of O-alkenylbenzamides via concomitant 1,2-aryl migration/elimination mediated by hypervalent iodine reagents
Source: Commun Chem. 2023 Jun 17;6:126. doi: 10.1038/s42004-023-00930-5 (PMC10276869; doi:10.1038/s42004-023-00930-5)
Supplement: Supplementary file 2 — Supplementary Information [file 42004_2023_930_MOESM2_ESM.pdf]

Chemoselective Cycloisomerization of *O*-alkenylbenzamides via Concomitant 1,2-Aryl migration/Elimination Mediated by Hypervalent Iodine Reagents

Jiaxin He[1†], Fenghuan Du[2†], Chi Zhang[2*] and Yunfei Du[1*]

[1] Tianjin Key Laboratory for Modern Drug Delivery & High-Efficiency, School of Pharmaceutical Science and Technology, Tianjin University, Tianjin 300072, China.

[2] State Key Laboratory of Elemento-Organic Chemistry, The Research Institute of Elemento-Organic Chemistry, College of Chemistry, Nankai University, Tianjin 300071, China.

[†] These authors contributed equally: Jiaxin He, Feng-Huan Du

[*] Corresponding authors' email: duyunfeier@tju.edu.cn, zhangchi@nankai.edu.cn

Table of Contents

**Supplementary Methods**		S1
**I**	General Information	S1
**II**	Experimental Procedures and Spectroscopic Data	S2-S37
**III**	X-ray Crystal Structure and Data of Products **2p** and **3t**	S38-S57
**IV**	Supplementary References	S58

Supplementary Methods

I. General Information

^{1}H and ^{13}C NMR spectra were recorded on a 400 MHz or 600 MHz spectrometer at 25 °C. Chemical shifts values are given in ppm and referred as the internal standard to TMS: 0.00 ppm. Chemical shifts were expressed in parts per million (δ) downfield from the internal standard tetramethylsilane, and were reported as s (singlet), d (doublet), t (triplet), q (quadruple), dd (doublet of doublet), m (multiplet), etc. The coupling constants *J*, are reported in Hertz (Hz). High resolution mass spectrometry (HRMS) data were recorded on Q Exactive HF (Q Exactive™ HF/UltiMate™ 3000 RSLCnano) using electron spray ionization (ESI) in positive (or negative) mode. Melting points were determined with a Micromelting point apparatus. TLC plates were visualized by exposure to ultraviolet light.

Reagents and solvents were purchased as reagent grade and were used without further purification. All reactions were performed in standard glassware, heated at 70 °C for 3 h before used. Flash column chromatography was performed over silica gel (200-300 m) using a mixture of ethyl acetate (EtOAc) and petroleum ether (PE).

II. Experimental Procedures and Spectroscopic Data

1. Table S1. Optimization study for the formation of 2a[a]

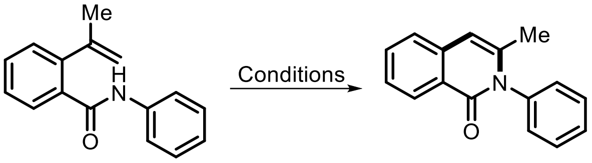

Entry	HIR	Solvent	Additive (equiv)	T (°C)	Yield (%)[b]
1	PhIO	MeOH	—	rt	NR
2	PIDA	MeOH	—	rt	NR
3	PIFA	MeOH	—	rt	34
4	HTIB	MeOH	—	rt	ND
5	PhIO	MeOH	BF_3•OEt_2 (1.5)	rt	43
6	PIDA	MeOH	BF_3•OEt_2 (1.5)	rt	40
7	PhIO	MeOH	BF_3•OEt_2 (1.0)	rt	46
8	PhIO	MeOH	BF_3•OEt_2 (0.5)	rt	49
9	PhIO	MeOH	BF_3•OEt_2 (0.2)	rt	56
10	PhIO	MeOH	BF_3•OEt_2 (0.1)	rt	53
11	PhIO	MeOH	Et_3N (0.2)	rt	NR
12	PhIO	MeOH	TFA (0.2)	rt	61
13	PhIO	MeOH	TfOH (0.2)	rt	63
14	PhIO	MeOH	TMSOTf (0.2)	rt	70
15	PhIO	MeOH	$LiClO_4$ (0.2)	rt	NR
16	PhIO	MeOH	50% H_2SO_4 (0.2)	rt	58
**17**	PhIO	MeOH	TMSOTf (0.2)	40	76
18	**PhIO**	**MeOH**	**TMSOTf (0.2)**	**reflux**	**81**
19	PhIO	HFIP	TMSOTf (0.2)	reflux	trace
20	PhIO	TFE	TMSOTf (0.2)	reflux	trace
21	PhIO	EtOH	TMSOTf (0.2)	reflux	NR
22	PhIO	^{i}PrOH	TMSOTf (0.2)	reflux	NR

23[c]	PhIO	MeOH	TMSOTf (0.2)	reflux	81

[a] Reaction conditions: **1a** (0.5 mmol), HIR (1.5 equiv), solvent (5.0 mL), stirred for 2.0 h. [b] Isolated yield. NR = no reaction. ND = no desired product. [c] PhIO (2.0 equiv).

2. Table S2. Optimization study for the formation of 3a[a]

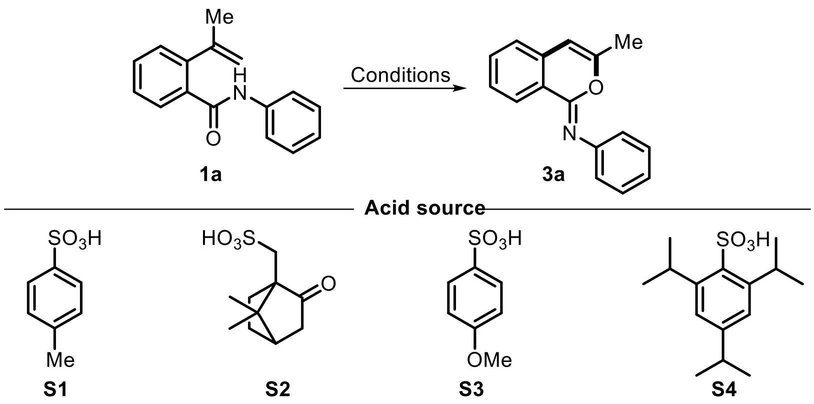

Entry	HIR	Solvent	Additive	T (°C)	Yield (%)[b]
1	HTIB	DCE	—	rt	44
2	HTIB	^{t}BuOH	—	rt	ND
3	HTIB	TFE	—	rt	trace
4	HTIB	HFIP	—	rt	trace
5	HTIB	1,4-dioxane	—	rt	NR
6	HTIB	MeCN	—	rt	36
7	HTIB	DCM	—	rt	29
8	HTIB	MeOH	—	rt	ND
9	PIDA	DCE	—	rt	trace
10	PIFA	DCE	—	rt	trace
11	PhIO	DCE	—	rt	NR
12	HTIB	DCE	—	0	trace
13	HTIB	DCE	—	40	45
14	HTIB	DCE	—	60	49
15	HTIB	DCE	—	80	55
16	PhIO	DCE	S1	80	54

17	PhIO	DCE	S2	80	52
18	PhIO	DCE	S3	80	63
19	PhIO	DCE	S4	80	69
20[c]	PhIO	DCE	S4	80	73
21[d]	PhIO	DCE	S4	80	70
**22[e]**	**PhIO**	**DCE**	**S4**	**80**	**90**
23[e]	PhIO	DCE	S4	rt	trace
24[f]	PhIO	DCE	S4	80	82
25[g]	PhIO	DCE	S4	80	77
26[h]	PhIO	DCE	S4	80	90

[a] Reaction conditions: **1a** (0.5 mmol), HIR (1.5 equiv), solvent (5.0 mL), stirred for 0.5 h. [b]Isolated yield. ND = no desired product. NR = no reaction. [c] $BF_3·OEt_2$ (1.5 equiv) was added, [d]TMSOTf (1.5 equiv) was added, [e] $LiClO_4$ (1.5 equiv) was added, [f] $Zn(ClO_4)_2$ (1.5 equiv) was added, [g] $LiClO_4$ (1.0 equiv) was added, [h] $LiClO_4$ (1.8 equiv) was added.

3. Synthesis of Starting Materials

General procedure for preparation of substrates 1a, 1g-1s.[1-4]

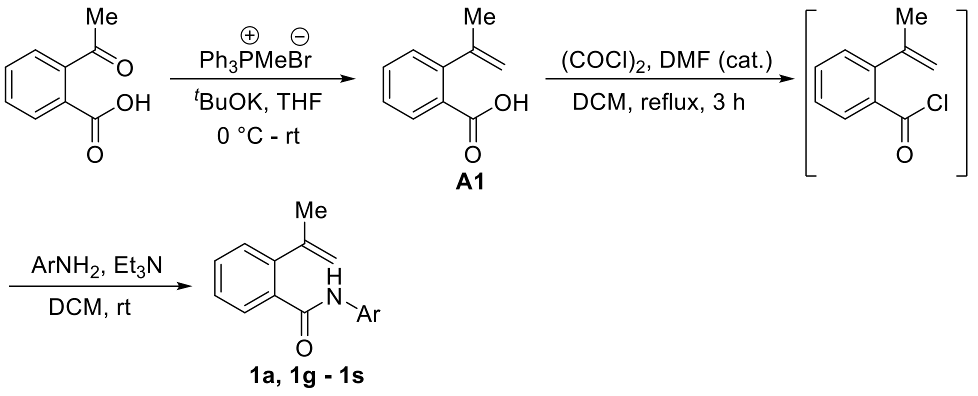

Method A:

2-(Prop-1-en-2-yl)benzoic acid (A1): To a suspension of methyltriphenylphosphonium bromide (9.79 g, 1.8 equiv) in anhydrous THF (200 mL) was added potassium *tert*-butoxide (5.13 g, 3.0 equiv) in a portionwise manner at 0 °C under nitrogen atmosphere. The mixture was stirred at 0 °C for 2 h. Then 2-acetylbenzoic acid (2.5 g, 1.0 equiv) was added at the same temperature. The mixture

was allowed to warm overnight to ambient temperature. After evaporation of THF, the mixture was treated with aq. NaOH (50 mL, 3.0 M). Then the aqueous layers were washed with EtOAc (30 mL×3) and acidified with HCl (3.0 M) to pH = 1. The acidified aqueous layer was next extracted with dichloromethane (30 mL × 3). The combined organic layer was washed with brine (30 mL), dried over anhydrous Na_2SO_4, and concentrated in vacuum. The residue was purified by flash chromatography on silica gel (EtOAc/petroleum ether = 1/5) to give product **A1** (1.87 g, 76%) as a white solid.

1a: To a solution of aniline (500 mg, 1.0 equiv) in CH_2Cl_2 (30 mL) under nitrogen atmosphere was added Et_3N (814.94 mg, 1.5 equiv) and acyl chloride, which was freshly prepared from **A1** (914.31 mg, 1.05 equiv) and oxalyl chloride (1.13 g, 1.5 equiv) in dichloromethane (30 mL). The resulting mixture was stirred at room temperature for overnight. The mixture was diluted with dichloromethane (20 mL) and washed with 10% HCl (20 mL × 2), sat. aq. $NaHCO_3$ (20 mL) and brine (20 mL) sequentially. The organic layer was dried over anhydrous Na_2SO_4, filtered and concentrated in vacuum. The residue was purified by silica gel chromatography (PE/EtOAc = 10/1, v/v) to give the product **1a** as a white solid.

***N*-Phenyl-2-(prop-1-en-2-yl)benzamide (1a)**

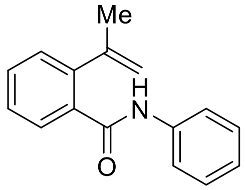

1a (1.09 g, 69% yield, a white solid, mp: 102 - 104 °C).

^{1}H NMR (400 MHz, $CDCl_3$) δ 7.98 (s, 1H), 7.79 (d, *J* = 7.6 Hz, 1H), 7.59 (d, *J* = 7.7 Hz, 2H), 7.46 (td, *J* = 7.5, 1.5 Hz, 1H), 7.42 – 7.34 (m, 3H), 7.28 (dd, *J* = 7.5, 1.2 Hz, 1H), 7.15 (t, *J* = 7.4 Hz, 1H), 5.33 (s, 1H), 5.21 (s, 1H), 2.10 (s, 3H). ^{13}C NMR (101 MHz, $CDCl_3$) δ 167.0, 146.9, 141.8, 138.1, 133.8, 130.9, 129.1, 129.1, 129.0, 127.9, 124.5, 119.7, 116.3, 24.3. HRMS (ESI) calcd for $C_{16}H_{15}NNaO^+$ [M + Na^+] 260.1046, found 260.1049.

N-(2-Fluorophenyl)-2-(prop-1-en-2-yl)benzamide (1g)

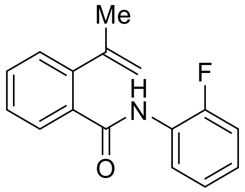

1g (932 mg, 81% yield, a white solid, mp: 76 - 78 °C).

^{1}H NMR (400 MHz, $CDCl_3$) δ 8.50 (t, J = 7.9 Hz, 1H), 8.33 (s, 1H), 7.83 (d, J = 7.1 Hz, 1H), 7.47 (td, J = 7.5, 1.3 Hz, 1H), 7.40 (td, J = 7.5, 1.2 Hz, 1H), 7.32 – 7.27 (m, 1H), 7.18 (t, J = 7.3 Hz, 1H), 7.14 – 7.09 (m, 1H), 7.07 (d, J = 7.0 Hz, 1H), 5.34 (s, 1H), 5.20 (s, 1H), 2.11 (s, 3H). ^{13}C NMR (101 MHz, $CDCl_3$) δ 166.9, 152.7, (d, $^{1}J_{C\text{-}F}$ = 244.5 Hz), 146.1, 142.0, 133.6, 131.1, 129.2, (d, $^{3}J_{C\text{-}F}$ = 6.2 Hz), 127.8, 126.6, (d, $^{2}J_{C\text{-}F}$ = 10.4 Hz), 124.7, (d, $^{4}J_{C\text{-}F}$ = 3.7 Hz), 124.4, (d, $^{3}J_{C\text{-}F}$ = 7.5 Hz), 121.7, 117.0, 114.8, (d, $^{2}J_{C\text{-}F}$ = 19.1 Hz), 24.4. HRMS (ESI) calcd for $C_{16}H_{14}FNNaO^{+}$ [M + Na^{+}] 278.0952, found 278.0950.

N-(2-Ethylphenyl)-2-(prop-1-en-2-yl)benzamide (1h)

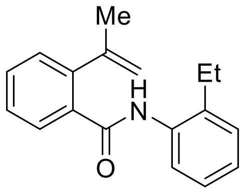

1h (970 mg, 89% yield, a white solid, mp: 112 - 114 °C).

^{1}H NMR (400 MHz, $CDCl_3$) δ 8.08 (d, J = 8.0 Hz, 1H), 7.82 – 7.75 (m, 2H), 7.46 (td, J = 7.4, 1.4 Hz, 1H), 7.40 (td, J = 7.5, 1.3 Hz, 1H), 7.31 – 7.27 (m, 2H), 7.23 (d, J = 7.6 Hz, 1H), 7.15 (t, J = 7.2 Hz, 1H), 5.29 (s, 1H), 5.22 (s, 1H), 2.59 (q, J = 7.6 Hz, 2H), 2.15 (s, 3H), 1.21 (t, J = 7.6 Hz, 3H). ^{13}C NMR (101 MHz, $CDCl_3$) δ 167.3, 146.5, 141.6, 135.3, 134.7, 134.4, 130.7, 129.1, 129.0, 128.7, 127.8, 126.8, 125.4, 125.3, 123.0, 116.5, 24.5, 24.2, 14.4. HRMS (ESI) calcd for $C_{18}H_{19}NNaO^{+}$ [M + Na^{+}] 288.1359, found 288.1358.

2-(Prop-1-en-2-yl)-N-(m-tolyl)benzamide (1i)

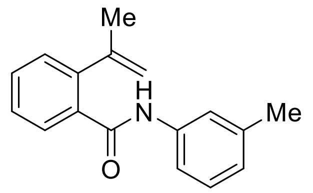

1i (878 mg, 75% yield, a white solid, mp: 87 - 88 °C).

^{1}H NMR (400 MHz, CDCl$_3$) δ 7.95 (s, 1H), 7.81 (d, *J* = 7.0 Hz, 1H), 7.52 – 7.28 (m, 5H), 7.26 (d, *J* = 7.8 Hz, 1H), 6.99 (d, *J* = 7.0 Hz, 1H), 5.36 (s, 1H), 5.23 (s, 1H), 2.40 (s, 3H), 2.13 (s, 3H). ^{13}C NMR (101 MHz, CDCl$_3$) δ 166.9, 146.8, 141.8, 139.1, 138.0, 133.9, 130.9, 129.1, 129.0, 128.9, 127.8, 125.4, 120.3, 116.7, 116.3, 24.3, 21.6. HRMS (ESI) calcd for $C_{17}H_{17}NNaO^+$ [M + Na$^+$] 274.1202, found 274.1205.

***N*-(3-Nitrophenyl)-2-(prop-1-en-2-yl)benzamide (1j)**

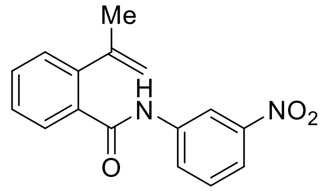

1j (745 mg, 73% yield, yellow liquid).

^{1}H NMR (400 MHz, CDCl$_3$) δ 8.74 (s, 1H), 8.48 (s, 1H), 7.89 (t, *J* = 6.9 Hz, 2H), 7.54 (d, *J* = 7.4 Hz, 1H), 7.44 (t, *J* = 8.1 Hz, 1H), 7.38 (t, *J* = 7.4 Hz, 1H), 7.24 (d, *J* = 7.1 Hz, 2H), 5.25 (s, 1H), 5.10 (s, 1H), 2.03 (s, 3H). ^{13}C NMR (101 MHz, CDCl$_3$) δ 168.1, 148.5, 145.7, 141.9, 139.3, 133.3, 131.0, 129.8, 129.0, 128.4, 127.6, 125.6, 119.0, 116.5, 114.6, 24.1. HRMS (ESI) calcd for $C_{16}H_{14}N_2NaO_3^+$ [M + Na$^+$] 305.0897, found 305.0894.

***N*-(3,4-Dichlorophenyl)-2-(prop-1-en-2-yl)benzamide (1k)**

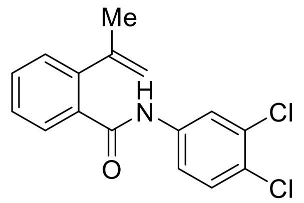

1k (775 mg, 82% yield, a white solid, mp: 107 - 109 °C).

^{1}H NMR (600 MHz, CDCl$_3$) δ 8.11 (s, 1H), 7.83 (s, 1H), 7.78 – 7.70 (m, 1H), 7.49 – 7.44 (m, 1H), 7.38 (t, *J* = 5.9 Hz, 3H), 7.27 (d, *J* = 7.6 Hz, 1H), 5.34 (s, 1H), 5.19 (s,

1H), 2.07 (s, 3H). ^{13}C NMR (151 MHz, $CDCl_3$) δ 167.0, 146.8, 141.8, 137.6, 133.1, 132.9, 131.2, 130.6, 129.2, 129.0, 127.9, 127.7, 121.3, 118.8, 116.6, 24.3. HRMS (ESI) calcd for $C_{16}H_{13}Cl_2NNaO^+$ [M + Na$^+$] 328.0266, found 328.0264.

***N*-(4-Fluorophenyl)-2-(prop-1-en-2-yl)benzamide (1l)**

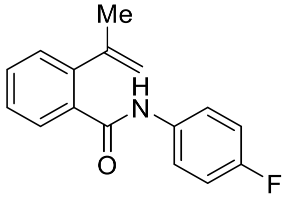

1l (874 mg, 76% yield, a white solid, mp: 114 - 116 °C).

^{1}H NMR (600 MHz, $CDCl_3$) δ 7.98 (s, 1H), 7.77 (d, *J* = 7.6 Hz, 1H), 7.54 (dd, *J* = 8.9, 4.8 Hz, 2H), 7.45 (t, *J* = 7.4 Hz, 1H), 7.39 (t, *J* = 7.5 Hz, 1H), 7.28 (d, *J* = 7.5 Hz, 1H), 7.05 (t, *J* = 8.6 Hz, 2H), 5.33 (s, 1H), 5.20 (s, 1H), 2.09 (s, 3H). ^{13}C NMR (101 MHz, $CDCl_3$) δ 166.9, 159.5, (d, $^1J_{C\text{-}F}$ = 244.7 Hz), 146.8, 141.7, 134.1, (d, $^4J_{C\text{-}F}$ = 2.8 Hz), 133.6, 131.0, 129.1, 129.0, 127.9, 121.4, (d, $^3J_{C\text{-}F}$ = 7.9 Hz), 116.4, 115.8, (d, $^2J_{C\text{-}F}$ = 22.6 Hz), 24.3. HRMS (ESI) calcd for $C_{16}H_{14}FNNaO^+$ [M + Na$^+$] 278.0952, found 278.0955.

***N*-(4-Bromophenyl)-2-(prop-1-en-2-yl)benzamide (1m)**

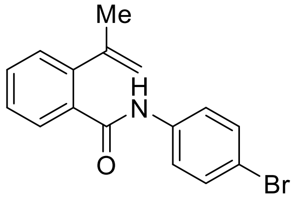

1m (818 mg, 89% yield, a white solid, mp: 147 - 149 °C).

^{1}H NMR (400 MHz, $CDCl_3$) δ 7.99 (s, 1H), 7.78 (d, *J* = 7.5 Hz, 1H), 7.47 (m, *J* = 9.3, 5.2 Hz, 5H), 7.40 (t, *J* = 7.5 Hz, 1H), 7.28 (d, *J* = 8.2 Hz, 1H), 5.34 (s, 1H), 5.21 (s, 1H), 2.08 (s, 3H). ^{13}C NMR (101 MHz, $CDCl_3$) δ 166.9, 146.9, 141.8, 137.2, 133.4, 132.1, 131.1, 129.1, 129.1, 127.9, 121.1, 117.1, 116.4, 24.3. HRMS (ESI) calcd for $C_{16}H_{14}BrNNaO^+$ [M + Na$^+$] 338.0151, found 338.0153.

***N*-(4- Iodophenyl)-2-(prop-1-en-2-yl)benzamide (1n)**

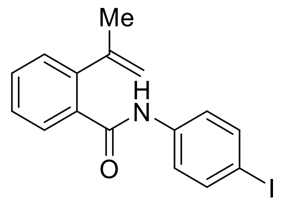

1n (730 mg, 88% yield, a white solid, mp: 159 - 160 °C).

^{1}H NMR (400 MHz, $CDCl_3$) δ 8.03 (s, 1H), 7.75 (d, *J* = 7.1 Hz, 1H), 7.64 (d, *J* = 8.7 Hz, 2H), 7.45 (td, *J* = 7.5, 1.3 Hz, 1H), 7.38 (m, *J* = 7.7 Hz, 3H), 7.27 (d, *J* = 8.5 Hz, 1H), 5.32 (s, 1H), 5.19 (s, 1H), 2.07 (s, 3H). ^{13}C NMR (151 MHz, $CDCl_3$) δ 166.9, 146.8, 141.8, 138.0, 137.9, 133.5, 131.1, 129.1, 129.0, 127.9, 121.4, 116.4, 87.6, 24.3. HRMS (ESI) calcd for $C_{16}H_{14}INNaO^+$ [M + Na^+] 386.0012, found 386.0015.

_N_-(4-Ethylphenyl)-2-(prop-1-en-2-yl)benzamide (1o)

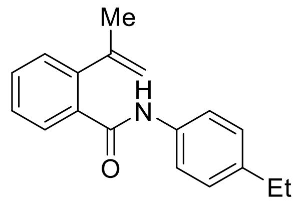

1o (807 mg, 74% yield, a white solid, mp: 100 - 101 °C).

^{1}H NMR (400 MHz, $CDCl_3$) δ 7.96 (s, 1H), 7.77 (d, *J* = 7.5 Hz, 1H), 7.50 (d, *J* = 8.4 Hz, 2H), 7.44 (td, *J* = 7.5, 1.4 Hz, 1H), 7.40 – 7.35 (m, 1H), 7.27 (dd, *J* = 7.7, 1.2 Hz, 1H), 7.19 (d, *J* = 8.4 Hz, 2H), 5.32 (s, 1H), 5.20 (s, 1H), 2.64 (q, *J* = 7.6 Hz, 2H), 2.10 (s, 3H), 1.24 (t, *J* = 7.6 Hz, 3H). ^{13}C NMR (101 MHz, $CDCl_3$) δ 166.9, 146.8, 141.8, 140.6, 135.8, 134.0, 130.8, 129.1, 129.0, 128.4, 127.8, 119.8, 116.3, 28.4, 24.3, 15.7. HRMS (ESI) calcd for $C_{18}H_{19}NNaO^+$ [M + Na^+] 288.1359, found 288.1356.

_N_-(4- (_tert_-Butyl)phenyl)-2-(prop-1-en-2-yl)benzamide (1p)

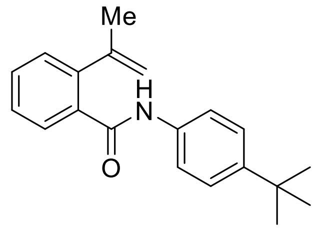

1p (885 mg, 90% yield, a white solid, mp: 110 - 112 °C).

^{1}H NMR (400 MHz, $CDCl_3$) δ 7.94 (s, 1H), 7.78 (d, *J* = 7.5 Hz, 1H), 7.51 (d, *J* = 8.6

Hz, 2H), 7.45 (td, *J* = 7.4, 1.2 Hz, 1H), 7.41 – 7.36 (m, 3H), 7.28 (d, *J* = 7.7 Hz, 1H), 5.31 (s, 1H), 5.20 (s, 1H), 2.10 (s, 3H), 1.32 (s, 9H). ^{13}C NMR (101 MHz, $CDCl_3$) δ 166.9, 147.5, 146.8, 141.7, 135.5, 134.0, 130.8, 129.1, 129.0, 127.8, 125.9, 119.5, 116.3, 34.4, 31.4, 24.3. HRMS (ESI) calcd for $C_{20}H_{23}NNaO^+$ [M + Na^+] 316.1672, found 316.1671.

N-(4-Methoxyphenyl)-2-(prop-1-en-2-yl)benzamide (1q)

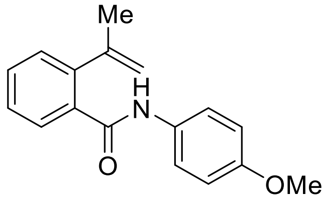

1q (937 mg, 86% yield, a white solid, mp: 119 - 121 °C).

^{1}H NMR (400 MHz, $CDCl_3$) δ 7.91 (s, 1H), 7.75 (d, *J* = 7.3 Hz, 1H), 7.49 (d, *J* = 8.7 Hz, 2H), 7.46 – 7.40 (m, 1H), 7.37 (t, *J* = 7.2 Hz, 1H), 7.27 (d, *J* = 6.9 Hz, 1H), 6.89 (d, *J* = 8.7 Hz, 2H), 5.31 (s, 1H), 5.19 (s, 1H), 3.80 (s, 3H), 2.10 (s, 3H). ^{13}C NMR (101 MHz, $CDCl_3$) δ 166.8, 156.6, 146.8, 141.7, 134.0, 131.3, 130.7, 129.0, 128.9, 127.8, 121.5, 116.2, 114.3, 55.5, 24.3. HRMS (ESI) calcd for $C_{17}H_{17}NNaO_2^+$ [M + Na^+] 290.1151, found 290.1148.

N- ([1,1'-Biphenyl]-4-yl)-2-(prop-1-en-2-yl)benzamide (1r)

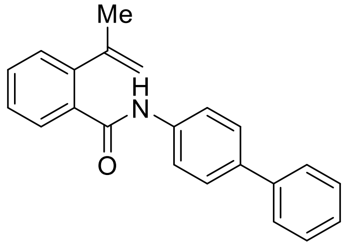

1r (732 mg, 79% yield, a brown solid, mp: 137 - 139 °C).

^{1}H NMR (600 MHz, $CDCl_3$) δ 8.09 (s, 1H), 7.80 (d, *J* = 7.4 Hz, 1H), 7.68 (d, *J* = 8.3 Hz, 2H), 7.62 – 7.58 (m, 4H), 7.48 – 7.39 (m, 4H), 7.35 (t, *J* = 7.4 Hz, 1H), 7.30 (d, *J* = 7.5 Hz, 1H), 5.36 (s, 1H), 5.24 (s, 1H), 2.13 (s, 3H). ^{13}C NMR (151 MHz, $CDCl_3$) δ 167.0, 146.8, 141.8, 140.5, 137.5, 133.9, 130.9, 129.1, 129.0, 128.8, 127.8, 127.8, 127.5,

127.1, 126.9, 120.0, 116.3, 24.3. HRMS (ESI) calcd for $C_{22}H_{19}NNaO^+$ [M + Na^+] 336.1359, found 336.1361.

N- (Naphthalen-2-yl)-2-(prop-1-en-2-yl)benzamide (1s)

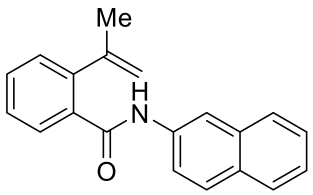

1s (676 mg, 68% yield, a white solid, mp: 108 - 110 °C).

^{1}H NMR (400 MHz, $CDCl_3$) δ 8.34 (s, 1H), 8.17 (s, 1H), 7.88 – 7.77 (m, 4H), 7.52 – 7.39 (m, 5H), 7.33 – 7.29 (m, 1H), 5.38 (s, 1H), 5.26 (s, 1H), 2.12 (s, 3H). ^{13}C NMR (101 MHz, $CDCl_3$) δ 167.1, 146.9, 141.8, 135.6, 133.9, 133.8, 131.0, 130.8, 129.2, 129.1, 128.9, 127.9, 127.8, 127.6, 126.6, 125.1, 119.5, 116.5, 116.4, 24.4. HRMS (ESI) calcd for $C_{20}H_{17}NNaO^+$ [M + Na^+] 310.1202, found 310.1205.

General procedure for preparation of substrates 1b - 1f, 1t.[5]

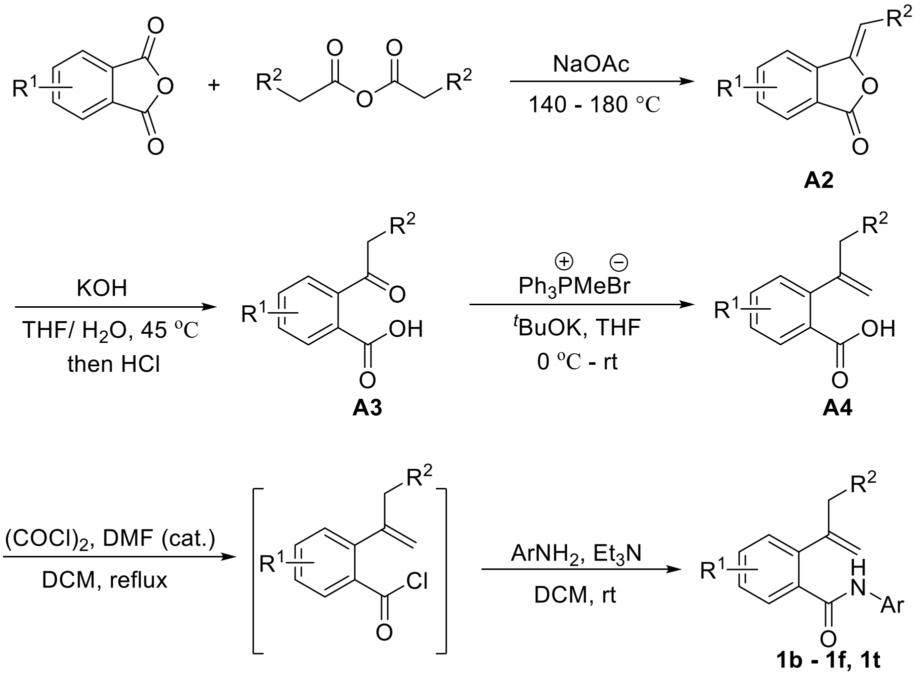

Method B:

A2: To a dried three-necked vessel was added phthalic anhydride (5.0 g, 1.0 equiv), propionic anhydride (6.59 g, 1.5 equiv) and sodium acetate (1.38 g, 0.5 equiv). After

being stirred at 140 °C for 3 h until no gas was produced, sodium acetate (1.38 g, 0.5 equiv) was added, and the mixture was then heated to 180 °C and stirred for 1 h. After that, the mixture was cooled to room temperature. Then water (150 mL) was added to the mixture and alkalified with ammonium hydroxide to pH = 8 - 9. The mixture was next extracted with dichloromethane (50 mL × 3), and the combined organic layer was washed with brine and dried over anhydrous Na_2SO_4. After filtration, the mixture was concentrated under vacuum, and the residue was purified by flash column chromatography on silica gel (EtOAc/petroleum ether = 1/20, v/v) to give compound **A2** (4.83 g, 89% yield) as light yellow oil.

A4: Product **A2** (4.5 g, 1.0 equiv) was added to a solution of KOH (4.73 g, 3.0 equiv) in THF/H_2O (1/1 = V/V, 50 mL). The reaction mixture was then allowed to stirred at 45 °C for 5 h. After cooling to room temperature, the mixture was quenched with a sat. aq. NH_4Cl (20 mL), and diluted with EtOAc (30 mL). The organic layer was extracted with aq. NaOH (3.0 M, 30 mL x 3). The combined aqueous layers were acidified with concentrated HCl to pH = 1 and then extracted with EtOAc (30 mL x 3). Then the combined organic layer was washed with brine (50 mL), dried with anhydrous Na_2SO_4, and concentrated in *vacuo* to afford compound **A3** (4.78 g, 95%), which was directly used for next step without further purification.

1b: The following preparation procedure from **A3** to **1b** is same to that of method A.

2-(But-1-en-2-yl)-*N*-phenylbenzamide (1b)

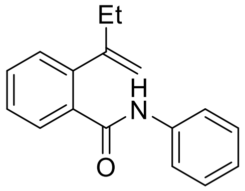

1b (932 mg, 69% yield, a white solid, mp: 75 - 77 °C).

^{1}H NMR (400 MHz, $CDCl_3$) δ 8.11 (s, 1H), 7.82 (d, *J* = 7.7 Hz, 1H), 7.58 (d, *J* = 7.2 Hz, 2H), 7.45 (td, *J* = 7.5, 1.6 Hz, 1H), 7.40 (dd, *J* = 7.5, 1.5 Hz, 1H), 7.38 – 7.32 (m,

2H), 7.24 (dd, J = 7.4, 1.2 Hz, 1H), 7.14 (t, J = 7.5 Hz, 1H), 5.35 (s, 1H), 5.23 (s, 1H), 2.39 (q, J = 7.4 Hz, 3H), 1.01 (t, J = 7.4 Hz, 3H). ^{13}C NMR (101 MHz, $CDCl_3$) δ 166.8, 153.1, 141.3, 138.1, 133.7, 130.8, 129.7, 129.3, 129.1, 127.9, 124.5, 119.6, 114.2, 30.9, 12.6. HRMS (ESI) calcd for $C_{17}H_{17}NNaO^+$ [M + Na^+] 274.1202, found 274.1205.

2-(3-Methylbut-1-en-2-yl)-*N*-phenylbenzamide (1c)

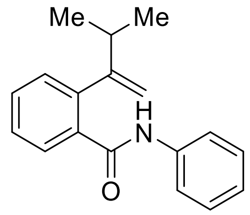

1c (767 mg, 54% yield, a white solid, mp: 95 - 97 °C).

^{1}H NMR (400 MHz, $CDCl_3$) δ 8.10 (s, 1H), 7.87 (dd, J = 7.4, 1.5 Hz, 1H), 7.58 (d, J = 7.7 Hz, 2H), 7.48 – 7.39 (m, 2H), 7.35 (t, J = 7.9 Hz, 2H), 7.20 (dd, J = 7.3, 1.4 Hz, 1H), 7.13 (t, J = 7.4 Hz, 1H), 5.37 (s, 1H), 5.24 (s, 1H), 2.57 (hept, J = 6.8 Hz, 1H), 1.01 (d, J = 6.8 Hz, 6H). ^{13}C NMR (101 MHz, $CDCl_3$) δ 166.7, 158.3, 141.4, 138.1, 133.6, 130.7, 130.2, 129.5, 129.1, 127.9, 124.4, 119.5, 112.5, 35.3, 21.5. HRMS (ESI) calcd for $C_{18}H_{19}NNaO^+$ [M + Na^+] 288.1359, found 288.1356.

2-(Hex-1-en-2-yl)-*N*-phenylbenzamide (1d)

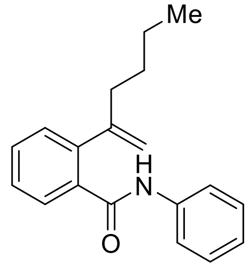

1d (915 mg, 61% yield, a white solid, mp: 103 - 105 °C).

^{1}H NMR (400 MHz, $CDCl_3$) δ 8.04 (s, 1H), 7.83 (d, J = 7.4 Hz, 1H), 7.57 (d, J = 7.9 Hz, 2H), 7.47 – 7.39 (m, 2H), 7.36 (t, J = 7.9 Hz, 2H), 7.25 – 7.22 (m, 1H), 7.14 (t, J = 7.4 Hz, 1H), 5.34 (s, 1H), 5.24 (s, 1H), 2.37 (t, J = 7.4 Hz, 2H), 1.38 – 1.19 (m, 4H), 0.80 (t, J = 7.1 Hz, 3H). ^{13}C NMR (101 MHz, $CDCl_3$) δ 166.8, 151.9, 141.2, 138.1, 133.7, 130.8, 129.8, 129.3, 129.1, 127.9, 124.5, 119.6, 115.1, 37.6, 30.2, 22.3, 13.8.

HRMS (ESI) calcd for $C_{19}H_{21}NNaO^+$ [M + Na$^+$] 302.1515, found 302.1517.

2-(Hept-1-en-2-yl)-*N*-phenylbenzamide (1e)

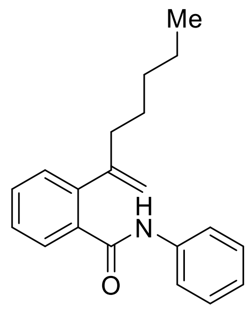

1e (1.03 g, 65% yield, a white solid, mp: 62 - 64 °C).

^{1}H NMR (400 MHz, $CDCl_3$) δ 8.07 (s, 1H), 7.82 (d, *J* = 7.5 Hz, 1H), 7.58 (d, *J* = 8.0 Hz, 2H), 7.45 (td, *J* = 7.4, 1.4 Hz, 1H), 7.42 – 7.39 (m, 1H), 7.36 (t, *J* = 7.9 Hz, 2H), 7.25 – 7.22 (m, 1H), 7.14 (t, *J* = 7.4 Hz, 1H), 5.37 – 5.31 (m, 1H), 5.24 (s, 1H), 2.37 (t, *J* = 7.6 Hz, 2H), 1.36 (p, *J* = 6.8 Hz, 2H), 1.24 – 1.17 (m, 4H), 0.80 (t, *J* = 6.8 Hz, 3H). ^{13}C NMR (101 MHz, $CDCl_3$) δ 166.8, 151.9, 141.2, 138.1, 133.7, 130.8, 129.8, 129.3, 129.1, 127.8, 124.5, 119.6, 115.1, 37.9, 31.3, 27.7, 22.4, 14.0. HRMS (ESI) calcd for $C_{20}H_{23}NNaO^+$ [M + Na$^+$] 316.1672, found 316.1675.

2-(Oct-1-en-2-yl)-*N*-phenylbenzamide (1f)

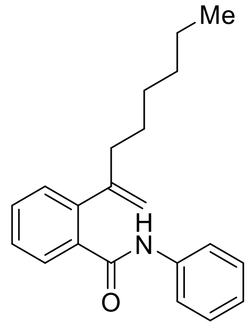

1f (1.27 g, 77% yield, a light yellow solid, mp: 56 - 58 °C).

^{1}H NMR (400 MHz, $CDCl_3$) δ 8.05 (s, 1H), 7.83 (d, *J* = 7.5 Hz, 1H), 7.57 (d, *J* = 7.8 Hz, 2H), 7.45 (td, *J* = 7.4, 1.7 Hz, 1H), 7.41 (dd, *J* = 7.6, 1.2 Hz, 1H), 7.36 (t, *J* = 7.9 Hz, 2H), 7.23 (dd, *J* = 7.4, 1.4 Hz, 1H), 7.14 (t, *J* = 7.4 Hz, 1H), 5.34 (d, *J* = 1.4 Hz, 1H), 5.24 (s, 1H), 2.36 (t, *J* = 7.6 Hz, 2H), 1.34 (p, *J* = 7.5, 7.1 Hz, 2H), 1.19 (m, 6H), 0.81 (t, *J* = 6.8 Hz, 3H). ^{13}C NMR (101 MHz, $CDCl_3$) δ 166.8, 152.0, 141.2, 138.1,

133.7, 130.8, 129.8, 129.3, 129.1, 127.9, 124.5, 119.6, 115.1, 37.9, 31.5, 28.8, 28.0, 22.5, 14.0. HRMS (ESI) calcd for $C_{21}H_{25}NNaO^+$ [M + Na^+] 330.1828, found 330.1831.

2-(But-1-en-2-yl)-4,5-dichloro-*N*-phenylbenzamide (1t)

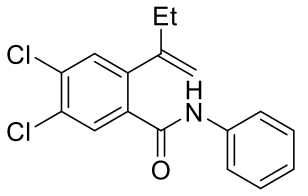

1t (1.43 g, 83% yield, a white solid, mp: 112 - 113 °C).

^{1}H NMR (400 MHz, $CDCl_3$) δ 8.00 (s, 1H), 7.93 (s, 1H), 7.53 (d, *J* = 7.7 Hz, 2H), 7.40 – 7.32 (m, 3H), 7.16 (t, *J* = 7.4 Hz, 1H), 5.40 (s, 1H), 5.27 (s, 1H), 2.37 (q, *J* = 7.4 Hz, 2H), 1.02 (t, *J* = 7.4 Hz, 3H). ^{13}C NMR (101 MHz, $CDCl_3$) δ 164.3, 151.1, 140.8, 137.6, 134.9, 133.3, 132.2, 131.3, 129.2, 124.9, 119.7, 119.6, 115.5, 30.6, 12.5. HRMS (ESI) calcd for $C_{17}H_{15}Cl_2NNaO^+$ [M + Na^+] 342.0423, found 342.0422.

General procedure for preparation of substrates 1u and 1v. [6-8]

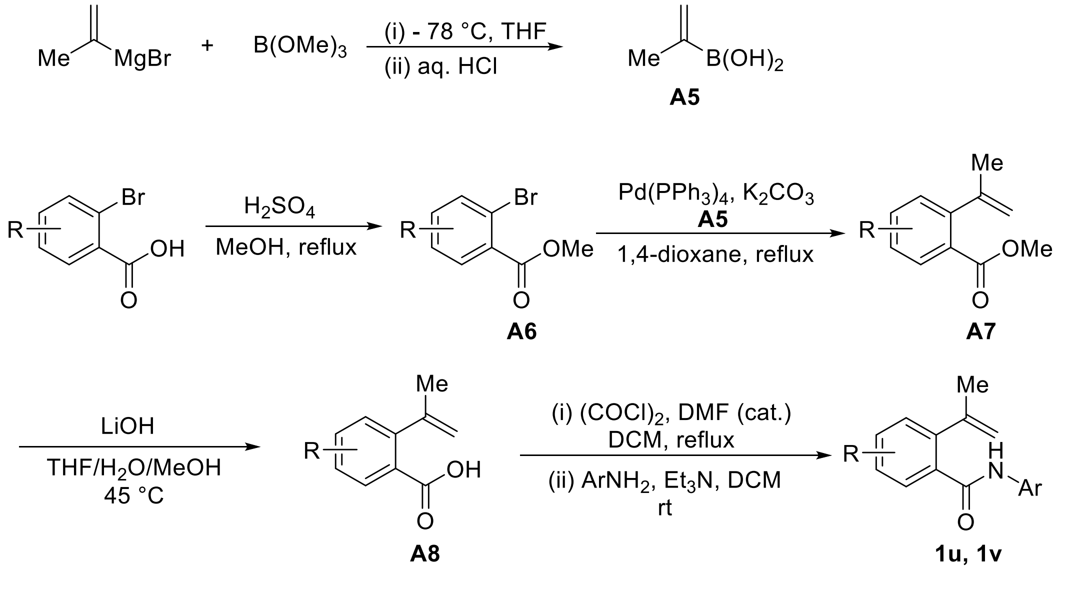

Method C:

A5: A solution of trimethyl borate (9.35 g, 1.8 equiv) in dry THF (50 mL) was cooled to -78 °C under nitrogen atmosphere. Then a solution of isopropenylmagnesium bromide in THF (1.0 M, 50 mL, 1.0 quiv) was added dropwise to the above solution. The resulting white slurry was allowed to stir and warm to room temperature overnight.

The reaction was then quenched with addition of water (50 mL). The remaining slurry was cooled to 0 °C and then aq. HCl (3.0 M, 50 mL) was added and the mixture was stirred for 30 min under nitrogen atmosphere. It was then extracted with diethyl ether (20 mL x 3), and the organic layer was dried with anhydrous Na_2SO_4 and concentrated in vacuum to give boronic acid **A5** (3.65 g, 85 % yield).

A7: To a solution of 2-bromo-3-methylbenzoic acid (2.0 g, 1.0 equiv) in methanol (50 mL) was added concentrated H_2SO_4 (1.02 mL, 2.0 equiv). The resulting mixture was refluxed for 10 h. Then the reaction was cooled to room temperature and concentrated *in vacuo* to remove ~50% of the MeOH. The resulting residue was slowly quenched with sat. aq. $NaHCO_3$ (20 mL). The aqueous layer was extracted with EtOAc (30 mL x 3). The combined organic layer was washed with brine (50 mL), dried with anhydrous Na_2SO_4, filtered and concentrated *in vacuo* to afford the crude ester. Purification by flash column chromatography on silica gel afforded ester **A6** as clear oil (2.02 g, 95%).

A solution of ester **A6** (2.0 g, 1.0 equiv) and tetrakis (triphenylphosphine)palladium (100.89 mg, 0.01 equiv) in 1,4-dioxane (50 mL) was stirred at room temperature under nitrogen atmosphere for 20 min. Then K_2CO_3 (1.21 g, 1.0 equiv) and **A5** (1.12 g, 1.5 equiv) were added sequentially. The reaction was allowed to reflux for 24 h until the completion of the reaction. Then the mixture was cooled to room temperature and quenched with sat. aq. NH_4Cl (30 mL). The aqueous layer was then extracted with EtOAc (30 mL x 3). The combined organic layer was washed with brine (50 mL), dried with anhydrous Na_2SO_4, filtered and concentrated in *vacuo* to afford the crude olefin. Purification by column chromatography afforded **A7** as pale oil (1.14 g, 69%).

1u: Product **A7** (1.14 g, 1.0 equiv) was added to a solution of LiOH (430.5 mg, 3.0 equiv) in THF/H_2O/MeOH (4/1/1 = v/v/v, 50 mL). The reaction was allowed to stir at 45 °C for 5 h. Then the reaction mixture was cooled to room temperature and acidified with HCl (3.0 M) to pH = 1. The aqueous layer was then extracted with dichloromethane (30 mL × 3). The combined organic layer was washed with brine (30

mL), dried over anhydrous Na_2SO_4, and concentrated in vacuum to give product **A8** (1.04 g, 98%). The following preparation procedure from **A8** to **1u** is same to that of method A.

3-Methyl-*N*-phenyl-2-(prop-1-en-2-yl)benzamide (1u)

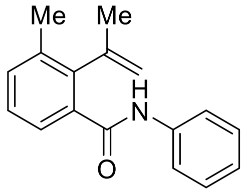

1u (958.5 mg, 71% yield, a white solid, mp: 147 - 149 °C).

^{1}H NMR (400 MHz, Chloroform-*d*) δ 8.24 (s, 1H), 7.58 (d, *J* = 7.8 Hz, 3H), 7.38 – 7.31 (m, 3H), 7.25 (t, *J* = 7.6 Hz, 1H), 7.14 (t, *J* = 7.4 Hz, 1H), 5.44 (s, 1H), 5.03 (s, 1H), 2.34 (s, 3H), 2.07 (s, 3H). ^{13}C NMR (101 MHz, $CDCl_3$) δ 167.3, 145.5, 140.8, 138.2, 135.5, 134.6, 132.5, 129.1, 127.1, 126.1, 124.4, 119.8, 116.6, 24.7, 19.9. HRMS (ESI) calcd for $C_{17}H_{17}NNaO^+$ [M + Na$^+$] 274.1202, found 274.1205.

4-Methoxy-*N*-phenyl-2-(prop-1-en-2-yl)benzamide (1v)

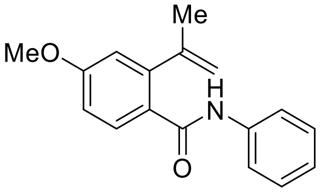

1v (1.04 g, 72% yield, a white solid, mp: 113 - 115 °C).

^{1}H NMR (400 MHz, $CDCl_3$) δ 8.21 (s, 1H), 7.90 – 7.10 (m, 6H), 6.91 (s, 1H), 6.79 (s, 1H), 5.37 (s, 1H), 5.25 (s, 1H), 3.88 (s, 3H), 2.11 (s, 3H). ^{13}C NMR (101 MHz, $CDCl_3$) δ 166.5, 161.4, 147.3, 143.8, 138.4, 131.3, 129.1, 126.0, 124.3, 119.6, 116.3, 114.6, 112.8, 55.5, 24.4. HRMS (ESI) calcd for $C_{17}H_{17}NNaO_2^+$ [M + Na$^+$] 290.1151, found 290.1152.

4. Typical Synthetic Procedure A and Spectroscopic Data of 2a-t

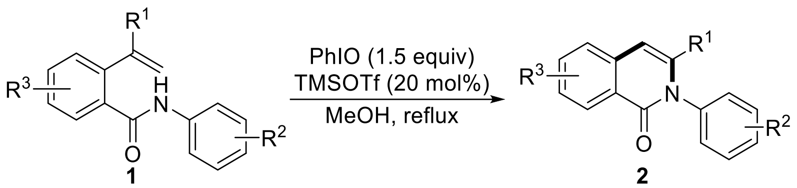

1 **2**

To a reaction flask filled with iodosobenzene (1.5 equiv, 0.75 mmol) in MeOH (5.0 mL) was added TMSOTf (20 mol %). The mixture was stirred at reflux temperature for 5 min and then reactant **1** (0.5 mmol) was added. The resulting mixture was kept stirring until TLC indicated the total consumption of substrate **1**. Then the reaction mixture was quenched with sat. aq. $NaHCO_3$ (5 mL), and extracted with EtOAc (10 mL x 3). The combined organic layer was dried over anhydrous Na_2SO_4 and the solvent was removed *in vacuo*. The residue was purified by flash column chromatography on silica gel to afford target product **2**.

3-Methyl-2-phenylisoquinolin-1(2*H*)-one (2a)

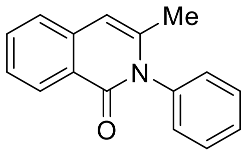

According to the procedure A, **2a** was purified by silica gel chromatography (PE/EtOAc = 10/1). A white solid (95.3 mg, yield: 81%). mp: 134 - 137 °C. ^{1}H NMR (400 MHz, $CDCl_3$) δ 8.40 (d, *J* = 8.1 Hz, 1H), 7.66 (ddd, *J* = 8.2, 7.1, 1.4 Hz, 1H), 7.57 – 7.52 (m, 2H), 7.51 – 7.43 (m, 3H), 7.28 (d, *J* = 1.4 Hz, 2H), 6.46 (s, 1H), 2.03 (s, 3H). ^{13}C NMR (101 MHz, $CDCl_3$) δ 163.5, 139.4, 139.2, 137.2, 132.6, 129.6, 128.6, 128.5, 128.2, 126.1, 125.2, 124.8, 105.5, 21.7. HRMS (ESI) calcd for $C_{16}H_{13}NNaO^+$ [M + Na^+] 258.0889, found 258.0887.

3-Ethyl-2-phenylisoquinolin-1(2*H*)-one (2b)

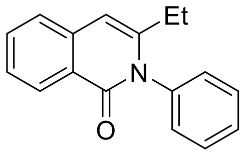

According to the procedure A, **2b** was purified by silica gel chromatography (PE/EtOAc = 10/1). A white solid (103.5 mg, yield: 83%). mp: 102 - 105 °C. ^{1}H NMR

(400 MHz, $CDCl_3$) δ 8.38 (d, *J* = 8.0 Hz, 1H), 7.68 – 7.62 (m, 1H), 7.56 – 7.49 (m, 3H), 7.49 – 7.41 (m, 2H), 7.25 (d, *J* = 6.9 Hz, 2H), 6.44 (s, 1H), 2.27 (q, *J* = 7.3 Hz, 2H), 1.13 (t, *J* = 7.4 Hz, 3H). ^{13}C NMR (101 MHz, $CDCl_3$) δ 163.6, 144.9, 138.7, 137.2, 132.6, 129.5, 128.8, 128.5, 128.1, 126.1, 125.5, 124.8, 103.4, 26.8, 12.3. HRMS (ESI) calcd for $C_{17}H_{15}NNaO^+$ [M + Na^+] 272.1046, found 272.1049.

3-Isopropyl-2-phenylisoquinolin-1(2*H*)-one (2c)

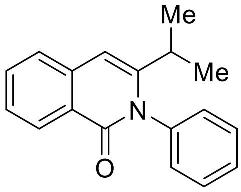

According to the procedure A, **2c** was purified by silica gel chromatography (PE/EtOAc = 10/1). A white solid (81.6 mg, yield: 62%). mp: 122 - 125 °C. ^1H NMR (400 MHz, $CDCl_3$) δ 8.37 (d, *J* = 8.0 Hz, 1H), 7.67 – 7.61 (m, 1H), 7.56 – 7.50 (m, 3H), 7.49 – 7.40 (m, 2H), 7.26 (d, *J* = 7.0 Hz, 2H), 6.49 (s, 1H), 2.57 (hept, *J* = 6.8 Hz, 1H), 1.15 (d, *J* = 6.8 Hz, 6H). ^{13}C NMR (101 MHz, $CDCl_3$) δ 163.6, 150.2, 138.7, 137.2, 132.5, 129.5, 129.0, 128.5, 128.1, 126.1, 125.5, 124.8, 101.9, 30.1, 22.9. HRMS (ESI) calcd for $C_{18}H_{17}NNaO^+$ [M + Na^+] 286.1202, found 286.1204.

3-Butyl-2-phenylisoquinolin-1(2*H*)-one (2d)

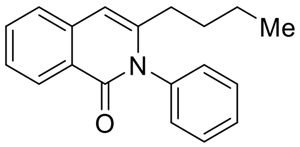

According to the procedure A, **2d** was purified by silica gel chromatography (PE/EtOAc = 10/1). Colorless liquid (104.0 mg, yield: 75%). ^1H NMR (400 MHz, $CDCl_3$) δ 8.40 (d, *J* = 8.0 Hz, 1H), 7.69 – 7.63 (m, 1H), 7.57 – 7.42 (m, 5H), 7.31 – 7.25 (m, 2H), 6.46 (s, 1H), 2.32 – 2.26 (m, 2H), 1.53 – 1.44 (m, 2H), 1.24 (h, *J* = 7.3 Hz, 2H), 0.81 (t, *J* = 7.3 Hz, 3H). ^{13}C NMR (101 MHz, $CDCl_3$) δ 163.6, 143.7, 138.7, 137.2, 132.5, 129.4, 128.9, 128.5, 128.1, 126.1, 125.4, 124.8, 104.4, 33.3, 30.4, 22.2, 13.7. HRMS (ESI) calcd for $C_{19}H_{19}NNaO^+$ [M + Na^+] 300.1359, found 300.1357.

3-Pentyl-2-phenylisoquinolin-1(2*H*)-one (2e)

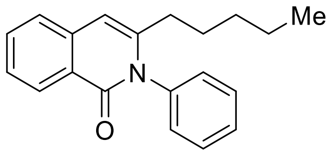

According to the procedure A, **2e** was purified by silica gel chromatography (PE/EtOAc = 10/1). A white solid (99.1 mg, yield: 68%). mp: 107 - 110 °C. ^{1}H NMR (400 MHz, $CDCl_3$) δ 8.38 (d, *J* = 8.0 Hz, 1H), 7.66 – 7.61 (m, 1H), 7.56 – 7.47 (m, 3H), 7.47 – 7.40 (m, 2H), 7.25 (d, *J* = 7.1 Hz, 2H), 6.43 (s, 1H), 2.29 – 2.23 (m, 2H), 1.48 (p, *J* = 7.5 Hz, 2H), 1.24 – 1.13 (m, 4H), 0.82 (t, *J* = 6.8 Hz, 3H). ^{13}C NMR (101 MHz, $CDCl_3$) δ 163.6, 143.7, 138.7, 137.1, 132.5, 129.4, 128.9, 128.5, 128.1, 126.1, 125.4, 124.8, 104.4, 33.6, 31.3, 27.9, 22.2, 13.9. HRMS (ESI) calcd for $C_{20}H_{21}NNaO^+$ [M + Na^+] 314.1515, found 314.1513.

3-Hexyl-2-phenylisoquinolin-1(2*H*)-one (2f)

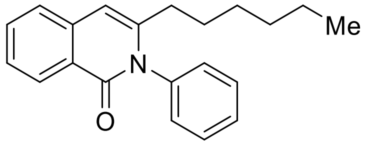

According to the procedure A, **2f** was purified by silica gel chromatography (PE/EtOAc = 10/1). A white solid (108.4 mg, yield: 71%). mp: 95 - 97 °C. ^{1}H NMR (400 MHz, $CDCl_3$) δ 8.38 (d, *J* = 8.0 Hz, 1H), 7.67 – 7.60 (m, 1H), 7.55 – 7.40 (m, 5H), 7.25 (d, *J* = 8.4 Hz, 2H), 6.43 (s, 1H), 2.29 – 2.22 (m, 2H), 1.47 (p, *J* = 7.6 Hz, 2H), 1.25 – 1.10 (m, 6H), 0.83 (t, *J* = 7.0 Hz, 3H). ^{13}C NMR (101 MHz, $CDCl_3$) δ 163.6, 143.7, 138.7, 137.2, 132.5, 129.4, 128.9, 128.5, 128.1, 126.1, 125.4, 124.8, 104.5, 33.6, 31.4, 28.8, 28.2, 22.4, 14.0. HRMS (ESI) calcd for $C_{21}H_{23}NNaO^+$ [M + Na^+] 328.1672, found 328.1675.

3-Methyl-2-(*m*-tolyl)isoquinolin-1(2*H*)-one (2i)

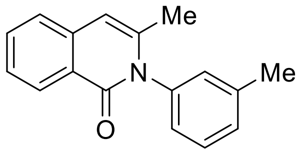

According to the procedure A, **2i** was purified by silica gel chromatography (PE/EtOAc

= 10/1). Colorless liquid (91.0 mg, yield: 73%). ^{1}H NMR (400 MHz, $CDCl_3$) δ 8.38 (d, *J* = 8.0 Hz, 1H), 7.63 (t, *J* = 7.2 Hz, 1H), 7.46 (d, *J* = 8.0 Hz, 1H), 7.43 (d, *J* = 7.8 Hz, 1H), 7.39 (d, *J* = 7.7 Hz, 1H), 7.26 (d, *J* = 7.3 Hz, 1H), 7.07 – 7.03 (m, 2H), 6.42 (s, 1H), 2.41 (s, 3H), 2.01 (s, 3H). ^{13}C NMR (101 MHz, $CDCl_3$) δ 163.5, 139.6, 139.6, 139.1, 137.2, 132.5, 129.4, 129.4, 129.0, 128.2, 126.0, 125.4, 125.1, 124.9, 105.4, 21.6, 21.4. HRMS (ESI) calcd for $C_{17}H_{15}NNaO^+$ [M + Na$^+$] 272.1046, found 272.1047.

3-Methyl-2-(3-nitrophenyl)isoquinolin-1(2*H*)-one (2j)

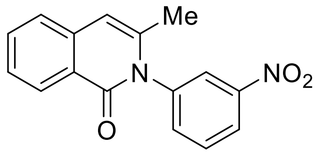

According to the procedure A, **2j** was purified by silica gel chromatography (PE/EtOAc = 10/1). A yellow solid (120.5 mg, yield: 86%). mp: 146 - 148 °C. ^{1}H NMR (400 MHz, $CDCl_3$) δ 8.36 – 8.30 (m, 2H), 8.16 (t, *J* = 1.9 Hz, 1H), 7.72 (t, *J* = 8.0 Hz, 1H), 7.68 – 7.61 (m, 2H), 7.48 (d, *J* = 8.0 Hz, 1H), 7.45 (t, *J* = 7.7 Hz, 1H), 6.48 (s, 1H), 2.01 (s, 3H). ^{13}C NMR (101 MHz, $CDCl_3$) δ 163.4, 149.0, 140.2, 138.1, 137.0, 135.2, 133.1, 130.5, 128.1, 126.6, 125.5, 124.5, 124.3, 123.7, 106.4, 21.7. HRMS (ESI) calcd for $C_{16}H_{12}N_2NaO_3^+$ [M + Na$^+$] 303.0740, found 303.0742.

2-(3,4-Dichlorophenyl)-3-methylisoquinolin-1(2*H*)-one (2k)

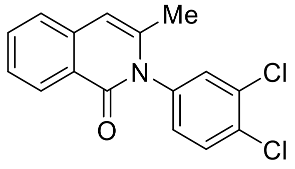

According to the procedure A, **2k** was purified by silica gel chromatography (PE/EtOAc = 10/1). A white solid (109.5 mg, yield: 72%). mp: 133 - 135 °C. ^{1}H NMR (400 MHz, $CDCl_3$) δ 8.35 (d, *J* = 7.9 Hz, 1H), 7.69 – 7.58 (m, 2H), 7.49 – 7.42 (m, 2H), 7.40 (s, 1H), 7.13 (d, *J* = 7.4 Hz, 1H), 6.45 (s, 1H), 2.03 (s, 3H). ^{13}C NMR (101 MHz, $CDCl_3$) δ 163.4, 138.5, 138.4, 137.0, 133.6, 133.2, 133.0, 131.3, 130.8, 128.2, 128.1, 126.5, 125.4, 124.6, 106.1, 21.6. HRMS (ESI) calcd for $C_{16}H_{11}Cl_2NNaO^+$ [M + Na$^+$] 326.0110, found 326.0107.

2-(4-Fluorophenyl)-3-methylisoquinolin-1(2*H*)-one (2l)

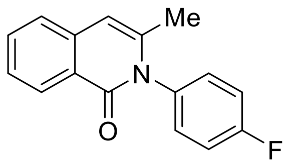

According to the procedure A, **2l** was purified by silica gel chromatography (PE/EtOAc = 10/1). A white solid (102.2 mg, yield: 82%). mp: 151 - 153 °C. ^{1}H NMR (600 MHz, $CDCl_3$) δ 8.36 (d, J = 8.0 Hz, 1H), 7.64 (t, J = 7.5 Hz, 1H), 7.48 – 7.41 (m, 2H), 7.25 – 7.18 (m, 4H), 6.44 (s, 1H), 2.01 (s, 3H). ^{13}C NMR (151 MHz, $CDCl_3$) δ 163.6, 162.4, (d, $^1J_{C\text{-}F}$ = 248.4 Hz), 139.2, 137.1, 135.0, 132.7, 130.3, (d, $^3J_{C\text{-}F}$ = 8.7 Hz), 128.2, 126.2, 125.2, 124.7, 116.6, (d, $^2J_{C\text{-}F}$ = 23.0 Hz), 105.7, (d, $^4J_{C\text{-}F}$ = 4.1 Hz), 21.7. HRMS (ESI) calcd for $C_{16}H_{12}FNNaO^+$ [M + Na^+] 276.0795, found 276.0797.

2-(4-Bromophenyl)-3-methylisoquinolin-1(2*H*)-one (2m)

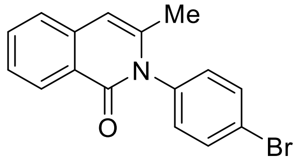

According to the procedure A, **2m** was purified by silica gel chromatography (PE/EtOAc = 10/1). A white solid (127.2 mg, yield: 81%). mp: 121 - 123 °C. ^{1}H NMR (400 MHz, $CDCl_3$) δ 8.36 (d, J = 8.0 Hz, 1H), 7.64 (t, J = 7.8 Hz, 3H), 7.48 – 7.40 (m, 2H), 7.14 (d, J = 8.5 Hz, 2H), 6.44 (s, 1H), 2.01 (s, 3H). ^{13}C NMR (101 MHz, $CDCl_3$) δ 163.4, 138.9, 138.1, 137.1, 132.9, 132.8, 130.3, 128.1, 126.3, 125.3, 124.7, 122.6, 105.8, 21.7. HRMS (ESI) calcd for $C_{16}H_{12}BrNNaO^+$ [M + Na^+] 335.9994, found 335.9991.

2-(4-Iodophenyl)-3-methylisoquinolin-1(2*H*)-one (2n)

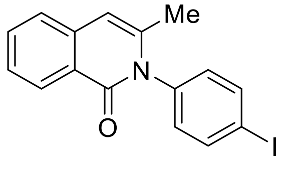

According to the procedure A, **2n** was purified by silica gel chromatography (PE/EtOAc = 10/1). A white solid (137.2 mg, yield: 76%). mp: 129 - 131 °C. ^{1}H NMR

(400 MHz, $CDCl_3$) δ 8.35 (d, *J* = 8.1 Hz, 1H), 7.88 – 7.81 (m, 2H), 7.64 (ddd, *J* = 8.2, 7.1, 1.3 Hz, 1H), 7.49 – 7.39 (m, 2H), 7.04 – 6.97 (m, 2H), 6.43 (s, 1H), 2.01 (s, 3H). ^{13}C NMR (101 MHz, $CDCl_3$) δ 163.4, 138.9, 138.8, 137.1, 132.8, 130.5, 128.2, 126.3, 125.2, 124.7, 105.8, 94.2, 21.7. HRMS (ESI) calcd for $C_{16}H_{12}INNaO^+$ [M + Na$^+$] 383.9856, found 383.9858.

2-(4-Ethylphenyl)-3-methylisoquinolin-1(2*H*)-one (2o)

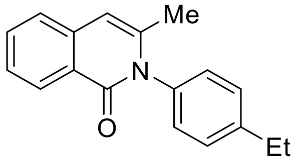

According to the procedure A, **2o** was purified by silica gel chromatography (PE/EtOAc = 10/1). A white solid (110.6 mg, yield: 84%). mp: 120 - 122 °C. ^{1}H NMR (400 MHz, $CDCl_3$) δ 8.38 (d, *J* = 8.0 Hz, 1H), 7.66 – 7.58 (m, 1H), 7.48 – 7.38 (m, 2H), 7.34 (d, *J* = 8.2 Hz, 2H), 7.15 (d, *J* = 8.2 Hz, 2H), 6.42 (s, 1H), 2.73 (q, *J* = 7.6 Hz, 2H), 2.01 (s, 3H), 1.30 (t, *J* = 7.6 Hz, 3H). ^{13}C NMR (101 MHz, $CDCl_3$) δ 163.6, 144.6, 139.8, 137.2, 136.6, 132.5, 129.0, 128.2, 128.2, 126.0, 125.1, 124.9, 105.3, 28.6, 21.7, 15.3. HRMS (ESI) calcd for $C_{18}H_{17}NNaO^+$ [M + Na$^+$] 286.1202, found 286.1205.

2-(4-(*tert*-Butyl)phenyl)-3-methylisoquinolin-1(2*H*)-one (2p)

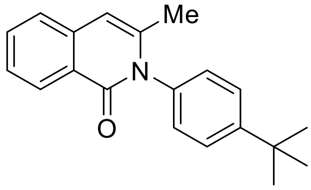

According to the procedure A, **2p** was purified by silica gel chromatography (PE/EtOAc = 10/1). A white solid (115.1 mg, yield: 79%). mp: 152 - 154 °C. ^{1}H NMR (400 MHz, $CDCl_3$) δ 8.37 (d, *J* = 8.0 Hz, 1H), 7.63 (ddd, *J* = 8.2, 7.2, 1.3 Hz, 1H), 7.54 – 7.49 (m, 2H), 7.47 – 7.39 (m, 2H), 7.20 – 7.12 (m, 2H), 6.42 (s, 1H), 2.02 (s, 2H), 1.37 (s, 9H). ^{13}C NMR (101 MHz, $CDCl_3$) δ 163.6, 151.4, 139.8, 137.2, 136.4, 132.5, 128.2, 127.8, 126.6, 126.0, 125.1, 124.9, 105.3, 34.8, 31.4, 21.7. HRMS (ESI) calcd for $C_{20}H_{21}NNaO^+$ [M + Na$^+$] 314.1515, found 314.1514.

2-(4-Methoxyphenyl)-3-methylisoquinolin-1(2*H*)-one (2q)

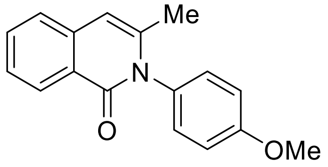

According to the procedure A, **2q** was purified by silica gel chromatography (PE/EtOAc = 10/1). A white solid (95.5 mg, yield: 72%). mp: 134 - 136 °C. ^{1}H NMR (400 MHz, $CDCl_3$) δ 8.37 (d, *J* = 8.0 Hz, 1H), 7.67 – 7.58 (m, 1H), 7.49 – 7.38 (m, 2H), 7.16 (d, *J* = 8.8 Hz, 2H), 7.02 (d, *J* = 8.8 Hz, 2H), 6.42 (s, 1H), 3.86 (s, 3H), 2.02 (s, 3H). ^{13}C NMR (101 MHz, $CDCl_3$) δ 163.8, 159.4, 139.9, 137.1, 132.5, 131.8, 129.4, 128.2, 126.0, 125.1, 124.8, 114.9, 105.3, 55.5, 21.7. HRMS (ESI) calcd for $C_{17}H_{15}NNaO_2^+$ [M + Na^+] 288.0995, found 288.0997.

2-([1,1'-Biphenyl]-4-yl)-3-methylisoquinolin-1(2*H*)-one (2r)

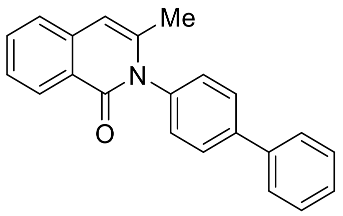

According to the procedure A, **2r** was purified by silica gel chromatography (PE/EtOAc = 10/1). A white solid (127.7 mg, yield: 82%). mp: 169 - 171 °C. ^{1}H NMR (400 MHz, $CDCl_3$) δ 8.41 (d, *J* = 7.9 Hz, 1H), 7.74 (d, *J* = 8.4 Hz, 2H), 7.67 – 7.62 (m, 3H), 7.50 (s, 1H), 7.49 – 7.43 (m, 3H), 7.39 (t, *J* = 7.3 Hz, 1H), 7.33 (d, *J* = 8.4 Hz, 2H), 6.47 (s, 1H), 2.07 (s, 3H). ^{13}C NMR (101 MHz, $CDCl_3$) δ 163.6, 141.6, 140.3, 139.5, 138.3, 137.2, 132.7, 128.9, 128.8, 128.4, 128.2, 127.7, 127.3, 126.2, 125.2, 124.8, 105.6, 21.8. HRMS (ESI) calcd for $C_{22}H_{17}NNaO^+$ [M + Na^+] 334.1202, found 334.1205.

3-Methyl-2-(naphthalen-2-yl)isoquinolin-1(2*H*)-one (2s)

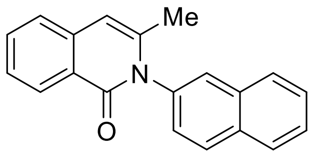

According to the procedure A, **2s** was purified by silica gel chromatography (PE/EtOAc = 10/1). A white solid (109.9 mg, yield: 77%). mp: 159 - 161 °C. ^{1}H NMR (400 MHz, $CDCl_3$) δ 8.41 (d, *J* = 7.8 Hz, 1H), 8.06 – 7.85 (m, 3H), 7.76 (s, 1H), 7.66 (t, *J* = 7.3 Hz, 1H), 7.60 – 7.52 (m, 2H), 7.53 – 7.42 (m, 2H), 7.35 (d, *J* = 8.1 Hz, 1H), 6.48 (s, 1H), 2.04 (s, 3H). ^{13}C NMR (101 MHz, $CDCl_3$) δ 163.7, 139.6, 137.2, 136.6, 133.7, 133.0, 132.7, 129.7, 128.2, 128.2, 127.9, 127.3, 126.8, 126.6, 126.2, 126.2, 125.2, 124.9, 105.7, 21.8. HRMS (ESI) calcd for $C_{20}H_{15}NNaO^+$ [M + Na^+] 308.1046, found 308.1043.

6,7-Dichloro-3-ethyl-2-phenylisoquinolin-1(2*H*)-one (2t)

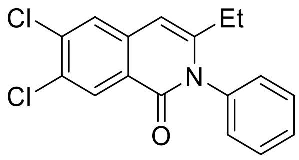

According to the procedure A, **2t** was purified by silica gel chromatography (PE/EtOAc = 10/1). A white solid (111.4 mg, yield: 70%). mp: 129 - 131 °C. ^{1}H NMR (400 MHz, $CDCl_3$) δ 8.42 (s, 1H), 7.62 (s, 1H), 7.54 (t, *J* = 7.3 Hz, 2H), 7.48 (t, *J* = 7.3 Hz, 1H), 7.23 (d, *J* = 7.2 Hz, 2H), 6.32 (s, 1H), 2.25 (q, *J* = 7.2 Hz, 2H), 1.12 (t, *J* = 7.4 Hz, 3H). ^{13}C NMR (101 MHz, $CDCl_3$) δ 162.0, 146.8, 138.2, 137.3, 136.4, 130.3, 129.8, 129.7, 128.9, 128.6, 126.8, 101.8, 26.9, 12.1. HRMS (ESI) calcd for $C_{17}H_{13}Cl_2NNaO^+$ [M + Na^+] 340.0266, found 340.0268.

3,5-Dimethyl-2-phenylisoquinolin-1(2*H*)-one (2u)

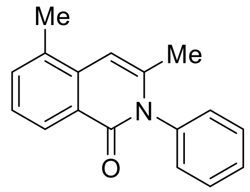

According to the procedure A, **2u** was purified by silica gel chromatography (PE/EtOAc = 10/1). Pale yellow liquid (92.2 mg, yield: 74%). ^{1}H NMR (400 MHz, $CDCl_3$) δ 8.25 (d, *J* = 8.0 Hz, 1H), 7.52 (t, *J* = 7.4 Hz, 2H), 7.48 – 7.42 (m, 2H), 7.31 (t, *J* = 7.7 Hz, 1H), 7.26 – 7.22 (m, 2H), 6.54 (s, 1H), 2.53 (s, 3H), 2.03 (s, 3H). ^{13}C NMR (101 MHz, $CDCl_3$) δ 163.8, 139.2, 139.1, 136.0, 133.4, 132.3, 129.6, 128.6, 128.4, 126.2, 125.7, 125.0, 102.2, 22.0, 19.0. HRMS (ESI) calcd for $C_{17}H_{15}NNaO^+$ [M + Na^+]

272.1046, found 272.1044.

6-Methoxy-3-methyl-2-phenylisoquinolin-1(2*H*)-one (2v)

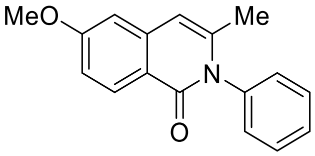

According to the procedure A, **2v** was purified by silica gel chromatography (PE/EtOAc = 4/1). A white solid (94.2 mg, yield: 71%). mp: 176 - 178 °C. ^{1}H NMR (400 MHz, $CDCl_3$) δ 8.29 (d, *J* = 8.9 Hz, 1H), 7.51 (t, *J* = 7.5 Hz, 2H), 7.44 (t, *J* = 7.1 Hz, 1H), 7.24 (d, *J* = 7.6 Hz, 2H), 7.03 – 6.95 (m, 1H), 6.82 (s, 1H), 6.35 (s, 1H), 3.90 (s, 3H), 1.98 (s, 3H). ^{13}C NMR (101 MHz, $CDCl_3$) δ 163.2, 163.0, 140.2, 139.2, 139.2, 130.2, 129.6, 128.6, 128.5, 118.7, 115.5, 106.1, 105.3, 55.5, 21.7. HRMS (ESI) calcd for $C_{17}H_{15}NNaO_2^+$ [M + Na^+] 288.0995, found 288.0998.

5. Typical Synthetic Procedure B and Spectroscopic Data of 3a-t

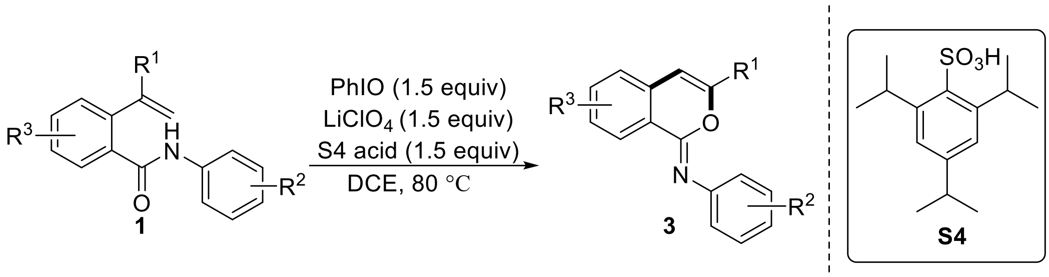

To a reaction flask filled with iodosobenzene (1.5 equiv, 0.75 mmol) in DCE (5.0 mL) was added **S4** (1.5 equiv, 0.75 mmol) and $LiClO_4$ (1.5 equiv, 0.75 mmol). The mixture was stirred at 80 °C for 5 min and then reactant **1** (0.5 mmol) was added. The resulting mixture was kept stirring until TLC indicated the total consumption of substrate **1**. Then the mixture was quenched with sat. aq. $NaHCO_3$ (5 mL), and extracted with dichloromethane (10 mL x 3). The combined organic layer was dried over anhydrous Na_2SO_4 and the solvent was removed *in vacuo*. The residue was purified by flash column chromatography on silica gel to afford target product **3**.

(*Z*)-3-Methyl-*N*-phenyl-1*H*-isochromen-1-imine (3a)

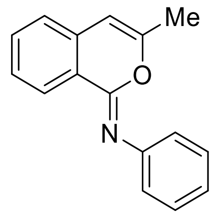

According to the procedure B, **3a** was purified by silica gel chromatography (PE/EtOAc = 50/1). Colorless liquid (105.9 mg, yield: 90%). ^{1}H NMR (400 MHz, $CDCl_3$) δ 8.33 (d, *J* = 7.9 Hz, 1H), 7.46 (td, *J* = 7.6, 1.3 Hz, 1H), 7.37 – 7.31 (m, 3H), 7.21 (dd, *J* = 8.4, 1.1 Hz, 2H), 7.13 (d, *J* = 7.7 Hz, 1H), 7.11 – 7.05 (m, 1H), 5.95 (s, 1H), 2.04 (s, 3H). ^{13}C NMR (101 MHz, $CDCl_3$) δ 152.7, 150.2, 146.6, 134.1, 132.3, 128.7, 127.5, 127.4, 124.5, 123.5, 123.2, 122.9, 102.5, 19.2. HRMS (ESI) calcd for $C_{16}H_{13}NNaO^+$ [M + Na$^+$] 258.0889, found 258.0886.

(*Z*)-3-Ethyl-*N*-phenyl-1*H*-isochromen-1-imine (3b)

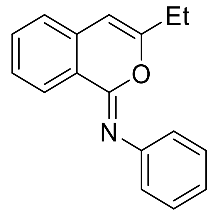

According to the procedure B, **3b** was purified by silica gel chromatography (PE/EtOAc = 50/1). Colorless liquid (114.7 mg, yield: 92%). ^{1}H NMR (600 MHz, $CDCl_3$) δ 8.36 (d, *J* = 7.5 Hz, 1H), 7.50 (t, *J* = 7.4 Hz, 1H), 7.37 (q, *J* = 8.0 Hz, 3H), 7.22 (d, *J* = 7.8 Hz, 2H), 7.19 (d, *J* = 7.7 Hz, 1H), 7.10 (t, *J* = 7.3 Hz, 1H), 5.99 (s, 1H), 2.37 (q, *J* = 7.4 Hz, 2H), 1.15 (t, *J* = 7.5 Hz, 3H). ^{13}C NMR (151 MHz, $CDCl_3$) δ 157.5, 150.4, 146.6, 134.2, 132.3, 128.6, 127.5, 127.5, 124.6, 123.5, 123.4, 122.9, 100.9, 26.4, 11.2. HRMS (ESI) calcd for $C_{17}H_{15}NNaO^+$ [M + Na$^+$] 272.1046, found 272.1048.

(*Z*)-3-Isopropyl-*N*-phenyl-1*H*-isochromen-1-imine (3c)

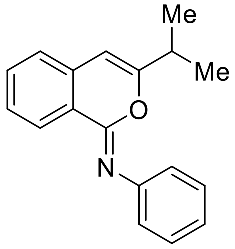

According to the procedure B, **3c** was purified by silica gel chromatography (PE/EtOAc = 50/1). Colorless liquid (115.9 mg, yield: 88%). ^{1}H NMR (400 MHz, $CDCl_3$) δ 8.38 (d, *J* = 7.5 Hz, 1H), 7.51 (td, *J* = 7.6, 1.2 Hz, 1H), 7.41 – 7.37 (m, 1H), 7.37 – 7.32 (m, 2H), 7.21 (dd, *J* = 7.0, 4.5 Hz, 3H), 7.13 – 7.05 (m, 1H), 6.00 (s, 1H), 2.58 (hept, *J* = 6.9 Hz, 1H), 1.14 (d, *J* = 6.9 Hz, 6H). ^{13}C NMR (101 MHz, $CDCl_3$) δ 161.0, 146.5, 134.2, 132.4, 128.6, 127.6, 127.4, 124.9, 123.5, 123.1, 122.8, 99.7, 32.1, 20.1. HRMS (ESI) calcd for $C_{18}H_{17}NNaO^+$ [M + Na^+] 286.1202, found 286.1205.

(*Z*)-3-Butyl-*N*-phenyl-1*H*-isochromen-1-imine (3d)

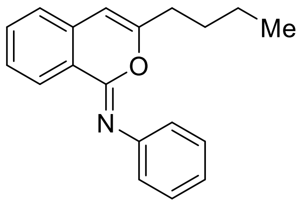

According to the procedure B, **3d** was purified by silica gel chromatography (PE/EtOAc = 50/1). Colorless liquid (113.7 mg, yield: 82%). ^{1}H NMR (400 MHz, $CDCl_3$) δ 8.36 (d, *J* = 7.9 Hz, 1H), 7.50 (td, *J* = 7.6, 1.3 Hz, 1H), 7.36 (qd, *J* = 7.4, 6.6, 1.5 Hz, 3H), 7.22 – 7.17 (m, 3H), 7.12 – 7.07 (m, 1H), 5.99 (s, 1H), 2.34 (t, *J* = 7.4 Hz, 2H), 1.54 (p, *J* = 7.4 Hz, 2H), 1.37 – 1.30 (m, 2H), 0.91 (t, *J* = 7.3 Hz, 3H). ^{13}C NMR (101 MHz, $CDCl_3$) δ 156.4, 150.5, 146.7, 134.1, 132.3, 128.6, 127.5, 127.4, 124.6, 123.4, 123.3, 122.8, 101.8, 32.7, 28.8, 22.0, 13.8. HRMS (ESI) calcd for $C_{19}H_{19}NNaO^+$ [M + Na^+] 300.1359, found 300.1357.

(*Z*)-3-Pentyl-*N*-phenyl-1*H*-isochromen-1-imine (3e)

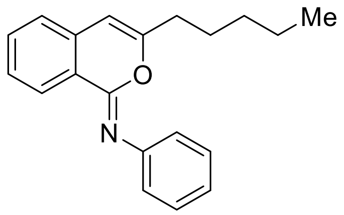

According to the procedure B, **3e** was purified by silica gel chromatography (PE/EtOAc = 50/1). Colorless liquid (123.8 mg, yield: 85%). ^{1}H NMR (400 MHz, $CDCl_3$) δ 8.36 (d, *J* = 7.9 Hz, 1H), 7.50 (td, *J* = 7.6, 1.2 Hz, 1H), 7.40 – 7.32 (m, 3H), 7.20 (t, *J* = 8.1 Hz, 3H), 7.10 (t, *J* = 7.3 Hz, 1H), 5.99 (s, 1H), 2.33 (t, *J* = 7.4 Hz, 2H), 1.56 (p, *J* = 7.4 Hz, 2H), 1.38 – 1.23 (m, 4H), 0.89 (t, *J* = 6.9 Hz, 3H). ^{13}C NMR (101 MHz, $CDCl_3$) δ 155.3, 149.4, 145.6, 133.1, 131.2, 127.5, 126.4, 126.3, 123.5, 122.4, 122.2, 121.7, 100.8, 31.9, 30.0, 25.3, 21.3, 12.9. HRMS (ESI) calcd for $C_{20}H_{21}NNaO^+$ [M + Na^+] 314.1515, found 314.1518.

(*Z*)-3-Hexyl-*N*-phenyl-1*H*-isochromen-1-imine (3f)

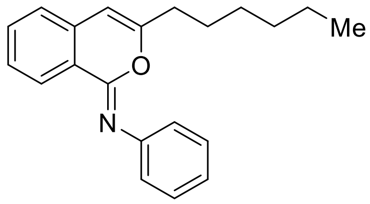

According to the procedure B, **3f** was purified by silica gel chromatography (PE/EtOAc = 50/1). Colorless liquid (131.3 mg, yield: 86%). ^{1}H NMR (400 MHz, $CDCl_3$) δ 8.36 (d, *J* = 7.9 Hz, 1H), 7.50 (td, *J* = 7.5, 1.4 Hz, 1H), 7.41 – 7.30 (m, 3H), 7.23 – 7.16 (m, 3H), 7.10 (t, *J* = 7.4 Hz, 1H), 5.99 (s, 1H), 2.33 (t, *J* = 7.4 Hz, 2H), 1.55 (p, *J* = 7.6, 7.0 Hz, 2H), 1.33 – 1.25 (m, 6H), 0.91 – 0.87 (m, 3H). ^{13}C NMR (101 MHz, $CDCl_3$) δ 156.4, 150.6, 146.6, 134.2, 132.3, 128.6, 127.5, 127.4, 124.6, 123.4, 123.2, 122.8, 101.9, 33.1, 31.5, 28.5, 26.6, 22.5, 14.1. HRMS (ESI) calcd for $C_{21}H_{23}NNaO^+$ [M + Na^+] 328.1672, found 328.1675.

(*Z*)-*N*-(2-Fluorophenyl)-3-methyl-1*H*-isochromen-1-imine (3g)

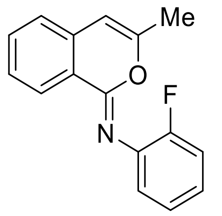

According to the procedure B, **3g** was purified by silica gel chromatography (PE/EtOAc = 50/1). A white solid (105.1 mg, yield: 83%). mp: 81 - 83 °C. ^{1}H NMR (400 MHz, $CDCl_3$) δ 8.39 (d, *J* = 7.9 Hz, 1H), 7.51 (t, *J* = 7.5 Hz, 1H), 7.38 (t, *J* = 7.6 Hz, 1H), 7.20 (t, *J* = 7.9 Hz, 2H), 7.15 – 7.01 (m, 3H), 6.01 (s, 1H), 2.05 (s, 3H). ^{13}C NMR (101 MHz, $CDCl_3$) δ 154.6, (d, $^{1}J_{C\text{-}F}$ = 246.7 Hz), 152.8, 152.1, 134.9, 134.8, 134.2, 132.6, 127.6, (d, $^{2}J_{C\text{-}F}$ = 19.3 Hz), 124.5, (d, $^{3}J_{C\text{-}F}$ = 6.2 Hz), 124.4, 124.2, (d, $^{3}J_{C\text{-}F}$ = 7.5 Hz), 124.0, (d, $^{4}J_{C\text{-}F}$ = 3.7 Hz), 122.6, 115.7, (d, $^{2}J_{C\text{-}F}$ = 20.7 Hz), 102.6, 19.1. HRMS (ESI) calcd for $C_{16}H_{12}FNNaO^+$ [M + Na^+] 276.0795, found 276.0792.

(*Z*)-*N*-(2-Ethylphenyl)-3-methyl-1*H*-isochromen-1-imine (3h)

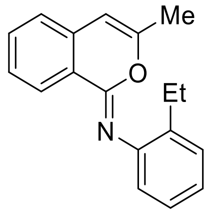

According to the procedure B, **3h** was purified by silica gel chromatography (PE/EtOAc = 50/1). A white solid (114.6 mg, yield: 87%). mp: 70 - 72 °C. ^{1}H NMR (400 MHz, $CDCl_3$) δ 8.40 (d, *J* = 7.9 Hz, 1H), 7.51 (td, *J* = 7.5, 1.4 Hz, 1H), 7.39 (t, *J* = 7.2 Hz, 1H), 7.28 (d, *J* = 7.5 Hz, 1H), 7.23 – 7.16 (m, 2H), 7.14 – 7.04 (m, 2H), 5.98 (s, 1H), 2.69 (q, *J* = 7.5 Hz, 2H), 2.04 (s, 3H), 1.22 (t, *J* = 7.5 Hz, 3H). ^{13}C NMR (101 MHz, $CDCl_3$) δ 152.9, 149.6, 144.9, 136.4, 134.1, 132.2, 128.5, 127.5, 127.5, 126.0, 124.4, 123.5, 123.2, 121.3, 102.2, 25.1, 19.3, 14.5. HRMS (ESI) calcd for $C_{18}H_{17}NNaO^+$ [M + Na^+] 286.1202, found 286.1201.

(*Z*)-3-Methyl-*N*-(*m*-tolyl)-1*H*-isochromen-1-imine (3i)

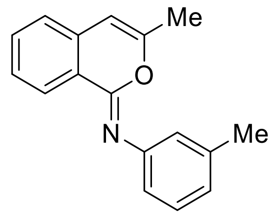

According to the procedure B, **3i** was purified by silica gel chromatography (PE/EtOAc = 50/1). Colorless liquid (103.5 mg, yield: 83%). ^{1}H NMR (400 MHz, $CDCl_3$) δ 8.39 (d, J = 7.8 Hz, 1H), 7.52 (td, J = 7.5, 1.4 Hz, 1H), 7.40 (t, J = 7.6 Hz, 1H), 7.29 (q, J = 3.9 Hz, 1H), 7.20 (d, J = 7.7 Hz, 1H), 7.11 – 7.05 (m, 2H), 6.96 (d, J = 7.5 Hz, 1H), 6.02 (s, 1H), 2.41 (s, 3H), 2.11 (s, 3H). ^{13}C NMR (101 MHz, $CDCl_3$) δ 152.7, 150.3, 146.3, 138.4, 134.1, 132.3, 128.5, 127.5, 127.5, 124.4, 124.4, 123.6, 123.1, 119.7, 102.5, 21.5, 19.2. HRMS (ESI) calcd for $C_{17}H_{15}NNaO^+$ [M + Na$^+$] 272.1046, found 272.1048.

(*Z*)-3-Methyl-*N*- (3-nitrophenyl)-1*H*-isochromen-1-imine (3j)

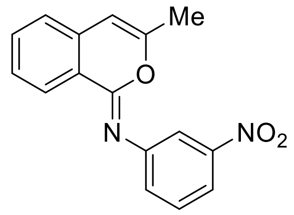

According to the procedure B, **3j** was purified by silica gel chromatography (PE/EtOAc = 20/1). A yellow solid (102.3 mg, yield: 73%). mp: 113 - 115 °C. ^{1}H NMR (400 MHz, $CDCl_3$) δ 8.34 (d, J = 8.0 Hz, 1H), 8.10 (s, 1H), 7.94 (d, J = 7.5 Hz, 1H), 7.57 – 7.52 (m, 1H), 7.52 – 7.44 (m, 2H), 7.40 (t, J = 7.6 Hz, 1H), 7.21 (d, J = 7.7 Hz, 1H), 6.08 (s, 1H), 2.10 (s, 3H). ^{13}C NMR (101 MHz, $CDCl_3$) δ 152.6, 152.0, 148.7, 148.0, 134.3, 132.9, 129.5, 129.2, 127.8, 127.5, 124.7, 122.4, 118.2, 117.9, 103.1, 19.0. HRMS (ESI) calcd for $C_{16}H_{12}N_2NaO_3^+$ [M + Na$^+$] 303.0740, found 303.0742.

(*Z*)- *N*-(3,4-Dichlorophenyl)-3-methyl-1*H*-isochromen-1-imine (3k)

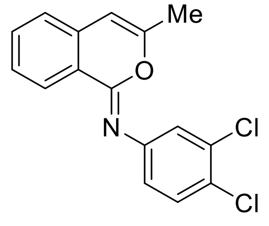

According to the procedure B, **3k** was purified by silica gel chromatography (PE/EtOAc = 50/1). A light yellow solid (109.2 mg, yield: 77%). mp: 86 - 88 °C. ^{1}H NMR (400 MHz, $CDCl_3$) δ 8.31 (d, *J* = 7.9 Hz, 1H), 7.53 (td, *J* = 7.6, 1.2 Hz, 1H), 7.41 – 7.36 (m, 2H), 7.34 (d, *J* = 2.3 Hz, 1H), 7.20 (d, *J* = 7.7 Hz, 1H), 7.06 (dd, *J* = 8.5, 2.3 Hz, 1H), 6.05 (s, 1H), 2.11 (s, 3H). ^{13}C NMR (101 MHz, $CDCl_3$) δ 152.6, 146.2, 134.2, 132.8, 132.1, 130.2, 127.7, 127.5, 126.6, 124.8, 124.6, 122.7, 122.6, 102.9, 19.1. HRMS (ESI) calcd for $C_{16}H_{11}Cl_2NNaO^+$ [M + Na^+] 326.0110, found 326.0113.

(*Z*)- *N*-(4-Fluorophenyl)-3-methyl-1*H*-isochromen-1-imine (3l)

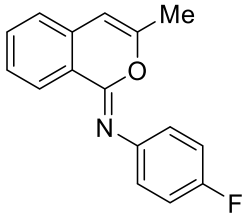

According to the procedure B, **3l** was purified by silica gel chromatography (PE/EtOAc = 50/1). Colorless liquid (108.9 mg, yield: 86%). ^{1}H NMR (600 MHz, $CDCl_3$) δ 8.33 (d, *J* = 7.9 Hz, 1H), 7.49 (td, *J* = 7.6, 1.2 Hz, 1H), 7.39 – 7.34 (m, 1H), 7.24 – 7.20 (m, 2H), 7.16 (d, *J* = 7.7 Hz, 1H), 7.07 – 7.01 (m, 2H), 6.00 (s, 1H), 2.09 (s, 3H). ^{13}C NMR (151 MHz, $CDCl_3$) δ 159.3, (d, $^{1}J_{C\text{-}F}$ = 241.6 Hz), 152.6, 150.4, (d, $^{4}J_{C\text{-}F}$ = 5.2 Hz), 142.5, 134.0, 132.3, 127.5, 127.4, 124.5, 124.4, (d, $^{3}J_{C\text{-}F}$ = 7.8 Hz), 123.2, 115.3, (d, $^{2}J_{C\text{-}F}$ = 22.2 Hz), 102.6, 19.1. HRMS (ESI) calcd for $C_{16}H_{12}FNNaO^+$ [M + Na^+] 276.0795, found 276.0797.

(*Z*)- *N*-(4-Bromophenyl)-3-methyl-1*H*-isochromen-1-imine (3m)

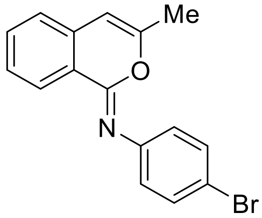

According to the procedure B, **3m** was purified by silica gel chromatography (PE/EtOAc = 50/1). Colorless liquid (138.2 mg, yield: 88%). ^{1}H NMR (400 MHz, $CDCl_3$) δ 8.32 (d, *J* = 7.9 Hz, 1H), 7.53 – 7.42 (m, 3H), 7.39 – 7.32 (m, 1H), 7.17 (d, *J*

= 7.7 Hz, 1H), 7.11 (d, *J* = 8.7 Hz, 2H), 6.00 (s, 1H), 2.07 (s, 3H). ^{13}C NMR (101 MHz, $CDCl_3$) δ 151.5, 149.7, 144.6, 133.0, 131.4, 130.5, 126.5, 126.3, 123.6, 123.5, 121.8, 115.2, 101.6, 18.0. HRMS (ESI) calcd for $C_{16}H_{12}BrNNaO^+$ [M + Na^+] 335.9994, found 335.9996.

(*Z*)- *N*-(4-Iodophenyl)-3-methyl-1*H*-isochromen-1-imine (3n)

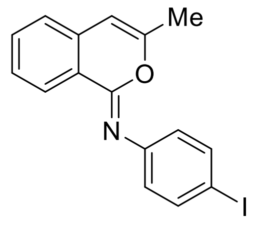

According to the procedure B, **3n** was purified by silica gel chromatography (PE/EtOAc = 50/1). A light yellow solid (142.7 mg, yield: 79%). mp: 77 - 79 °C. ^{1}H NMR (600 MHz, $CDCl_3$) δ 8.31 (d, *J* = 7.9 Hz, 1H), 7.63 (d, *J* = 8.3 Hz, 2H), 7.50 (t, *J* = 7.4 Hz, 1H), 7.36 (t, *J* = 7.6 Hz, 1H), 7.17 (d, *J* = 7.7 Hz, 1H), 6.98 (d, *J* = 8.4 Hz, 2H), 6.01 (s, 1H), 2.08 (s, 3H). ^{13}C NMR (151 MHz, $CDCl_3$) δ 152.6, 150.8, 146.4, 137.6, 134.1, 132.5, 127.6, 127.5, 125.1, 124.5, 122.9, 102.7, 87.0, 19.1. HRMS (ESI) calcd for $C_{16}H_{12}INNaO^+$ [M + Na^+] 383.9856, found 383.9854.

(*Z*)- *N*-(4-Ethylphenyl)-3-methyl-1*H*-isochromen-1-imine (3o)

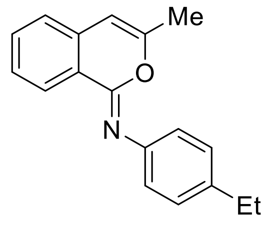

According to the procedure B, **3o** was purified by silica gel chromatography (PE/EtOAc = 50/1). Colorless liquid (110.6 mg, yield: 84%). ^{1}H NMR (400 MHz, $CDCl_3$) δ 8.27 (d, *J* = 7.8 Hz, 1H), 7.38 (td, *J* = 7.6, 1.1 Hz, 1H), 7.26 (t, *J* = 7.3 Hz, 1H), 7.14 – 7.08 (m, 4H), 7.06 (d, *J* = 7.7 Hz, 1H), 5.88 (s, 1H), 2.57 (q, *J* = 7.6 Hz, 2H), 2.00 (s, 3H), 1.18 (t, *J* = 7.6 Hz, 3H). ^{13}C NMR (101 MHz, $CDCl_3$) δ 151.6, 142.7, 138.5, 133.0, 131.1, 126.9, 126.4, 123.3, 122.3, 122.0, 101.4, 27.4, 18.1, 14.6. HRMS (ESI) calcd for $C_{18}H_{17}NNaO^+$ [M + Na^+] 286.1202, found 286.1205.

(*Z*)- *N*-(4-(*tert*-Butyl)phenyl)-3-methyl-1*H*-isochromen-1-imine (3p)

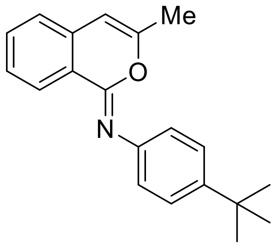

According to the procedure B, **3p** was purified by silica gel chromatography (PE/EtOAc = 50/1). A white solid (126.8 mg, yield: 87%). mp: 85 - 87 °C. ^{1}H NMR (400 MHz, $CDCl_3$) δ 8.29 (d, *J* = 7.9 Hz, 1H), 7.40 (td, *J* = 7.6, 1.1 Hz, 1H), 7.30 (t, *J* = 8.7 Hz, 3H), 7.19 (s, 1H), 7.17 (d, *J* = 3.0 Hz, 1H), 7.07 (d, *J* = 7.7 Hz, 1H), 5.90 (s, 1H), 2.04 (s, 3H), 1.29 (s, 9H). ^{13}C NMR (101 MHz, $CDCl_3$) δ 152.7, 149.8, 146.4, 143.5, 137.0, 134.0, 132.1, 127.4, 125.5, 124.4, 123.5, 122.9, 102.4, 34.4, 31.5, 19.3. HRMS (ESI) calcd for $C_{20}H_{21}NNaO^+$ [M + Na^+] 314.1515, found 314.1517.

(*Z*)- *N*-(4-Methoxyphenyl)-3-methyl-1*H*-isochromen-1-imine (3q)

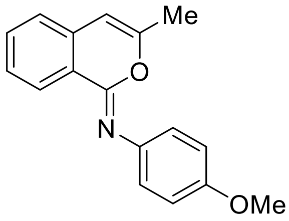

According to the procedure B, **3q** was purified by silica gel chromatography (PE/EtOAc = 50/1). Colorless liquid (118.1 mg, yield: 89%). ^{1}H NMR (400 MHz, $CDCl_3$) δ 8.36 (d, *J* = 7.3 Hz, 1H), 7.47 (t, *J* = 7.4 Hz, 1H), 7.35 (t, *J* = 7.6 Hz, 1H), 7.29 (d, *J* = 8.6 Hz, 2H), 7.15 (d, *J* = 7.6 Hz, 1H), 6.90 (d, *J* = 8.5 Hz, 2H), 5.99 (s, 1H), 3.83 (s, 3H), 2.12 (s, 3H). ^{13}C NMR (101 MHz, $CDCl_3$) δ 156.1, 152.6, 134.0, 132.1, 127.5, 127.3, 124.6, 124.4, 123.4, 123.4, 123.4, 113.9, 102.5, 55.4, 19.2. HRMS (ESI) calcd for $C_{17}H_{15}NNaO^+$ [M + Na^+] 288.0995, found 288.0998.

(*Z*)- *N*-([1,1'-Biphenyl]-4-yl)-3-methyl-1*H*-isochromen-1-imine (3r)

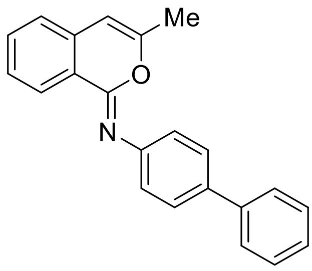

According to the procedure B, **3r** was purified by silica gel chromatography (PE/EtOAc = 50/1). A brown solid (133.9 mg, yield: 86%). mp: 96 - 98 °C. ^{1}H NMR (400 MHz, $CDCl_3$) δ 8.39 (d, J = 7.9 Hz, 1H), 7.68 – 7.62 (m, 3H), 7.61 (d, J = 1.8 Hz, 1H), 7.51 (td, J = 7.6, 1.1 Hz, 1H), 7.45 (t, J = 7.6 Hz, 2H), 7.40 (d, J = 7.4 Hz, 1H), 7.37 – 7.30 (m, 3H), 7.18 (d, J = 7.7 Hz, 1H), 6.02 (s, 1H), 2.12 (s, 3H). ^{13}C NMR (101 MHz, $CDCl_3$) δ 152.8, 150.5, 145.8, 141.1, 136.3, 134.2, 132.4, 128.8, 127.6, 127.5, 127.3, 126.8, 126.8, 124.5, 123.5, 123.2, 102.6, 19.2. HRMS (ESI) calcd for $C_{22}H_{17}NNaO^+$ [M + Na^+] 334.1202, found 334.1203.

(*Z*)-3-Methyl-*N*-(naphthalen-2-yl)-1*H*-isochromen-1-imine (3s)

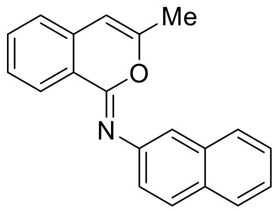

According to the procedure B, **3s** was purified by silica gel chromatography (PE/EtOAc = 50/1). Colorless liquid (109.9 mg, yield: 77%). ^{1}H NMR (400 MHz, $CDCl_3$) δ 8.44 (d, J = 7.9 Hz, 1H), 7.84 (d, J = 8.8 Hz, 2H), 7.80 (d, J = 8.3 Hz, 1H), 7.65 (s, 1H), 7.53 (td, J = 7.5, 1.4 Hz, 1H), 7.48 – 7.37 (m, 4H), 7.19 (d, J = 7.7 Hz, 1H), 6.02 (s, 1H), 2.06 (s, 3H). ^{13}C NMR (101 MHz, $CDCl_3$) δ 151.7, 149.8, 143.2, 133.2, 133.1, 131.4, 129.6, 128.4, 127.1, 126.5, 126.5, 126.4, 126.4, 124.8, 123.4, 123.3, 122.5, 118.1, 101.6, 18.1. HRMS (ESI) calcd for $C_{20}H_{15}NNaO^+$ [M + Na^+] 308.1046, found 308.1044.

(*Z*)-6,7-Dichloro-3-ethyl-*N*-phenyl-1*H*-isochromen-1-imine (3t)

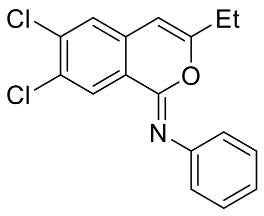

According to the procedure B, **3t** was purified by silica gel chromatography (PE/EtOAc = 50/1). A yellow solid (116.1 mg, yield: 73%). mp: 111 - 113 °C. ^{1}H NMR (400 MHz, $CDCl_3$) δ 8.42 (s, 1H), 7.35 (t, *J* = 7.8 Hz, 2H), 7.27 (s, 1H), 7.21 (d, *J* = 7.4 Hz, 2H), 7.12 (t, *J* = 7.4 Hz, 1H), 5.90 (s, 1H), 2.37 (q, *J* = 7.4 Hz, 2H), 1.14 (t, *J* = 7.5 Hz, 3H). ^{13}C NMR (101 MHz, $CDCl_3$) δ 159.1, 136.7, 133.7, 131.3, 129.6, 129.2, 128.7, 126.0, 124.0, 122.9, 121.3, 113.1, 99.4, 26.4, 11.0. HRMS (ESI) calcd for $C_{17}H_{13}Cl_2NNaO^+$ [M + Na^+] 340.0266, found 340.0268.

(*Z*)-3,5-Dimethyl-*N*-phenyl-1*H*-isochromen-1-imine (3u)

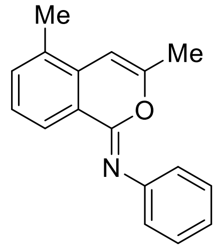

According to the procedure B, **3u** was purified by silica gel chromatography (PE/EtOAc = 50/1). Pale yellow liquid (110.9 mg, yield: 89%).^{1}H NMR (400 MHz, $CDCl_3$) δ 8.20 (d, *J* = 7.9 Hz, 1H), 7.37 – 7.28 (m, 3H), 7.25 – 7.17 (m, 3H), 7.07 (t, *J* = 7.3 Hz, 1H), 6.07 (s, 1H), 2.35 (s, 3H), 2.06 (s, 3H). ^{13}C NMR (101 MHz, $CDCl_3$) δ 152.4, 150.7, 146.8, 133.4, 132.7, 131.7, 128.7, 126.9, 125.3, 123.4, 123.2, 122.8, 99.1, 19.5, 18.7. HRMS (ESI) calcd for $C_{17}H_{15}NNaO^+$ [M + Na^+] 272.1046, found 272.1043.

(*Z*)-6-Methoxy-3-methyl-*N*-phenyl-1*H*-isochromen-1-imine (3v)

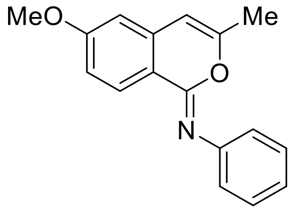

According to the procedure B, **3v** was purified by silica gel chromatography (PE/EtOAc = 50/1). Pale yellow liquid (103.5 mg, yield: 78%). ^{1}H NMR (400 MHz, $CDCl_3$) δ 8.27 (d, *J* = 8.6 Hz, 1H), 7.34 (t, *J* = 7.7 Hz, 2H), 7.20 (d, *J* = 7.6 Hz, 2H), 7.07 (t, *J* = 7.3 Hz, 1H), 6.92 (dd, *J* = 8.8, 2.2 Hz, 1H), 6.60 (d, *J* = 2.2 Hz, 1H), 5.94 (s, 1H), 3.87 (s, 3H), 2.06 (s, 3H). ^{13}C NMR (101 MHz, $CDCl_3$) δ 162.9, 153.3, 136.0, 129.5, 128.6, 123.2, 122.9, 116.2, 115.0, 107.4, 102.5, 55.5, 19.2. HRMS (ESI) calcd for $C_{17}H_{15}NNaO_2^+$ [M + Na^+] 288.0995, found 288.0997.

III. X-ray Crystal Structure and Data

Product 2p:

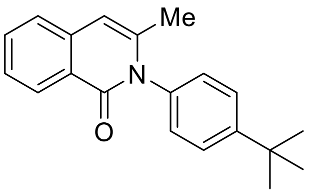

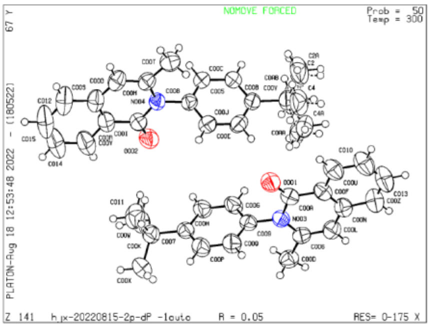

Table S3 Crystal data and structure refinement for 2p.

Identification code	2p
Empirical formula	$C_{40}H_{42}N_2O_2$
Formula weight	582.75
Temperature/K	300.26(10)
Crystal system	triclinic
Space group	P-1
a/Å	10.92450(10)
b/Å	12.56750(10)
c/Å	14.2956(2)
α/°	110.0180(10)
β/°	111.4170(10)
γ/°	93.5060(10)
Volume/Å^3	1677.45(3)
Z	2
ρ_{calc}g/cm^3	1.154
μ/mm^{-1}	0.546
F(000)	624.0
Crystal size/mm^3	0.1 × 0.1 × 0.1
Radiation	Cu Kα (λ = 1.54184)

2Θ range for data collection/°	7.662 to 152.674
Index ranges	-13 ≤ h ≤ 13, -15 ≤ k ≤ 13, -17 ≤ l ≤ 17
Reflections collected	22880
Independent reflections	6648 [R_{int} = 0.0311, R_{sigma} = 0.0305]
Data/restraints/parameters	6648/4/437
Goodness-of-fit on F^2	1.070
Final R indexes [I>=2σ (I)]	R_1 = 0.0462, wR_2 = 0.1357
Final R indexes [all data]	R_1 = 0.0536, wR_2 = 0.1430
Largest diff. peak/hole / e Å^{-3}	0.16/-0.14

Table S4 Fractional Atomic Coordinates (×10^4) and Equivalent Isotropic Displacement Parameters (Å^2×10^3) for 2p. U_{eq} is defined as 1/3 of of the trace of the orthogonalised U_{IJ} tensor.

**Atom**	***x***	***y***	***z***	**U(eq)**
O001	7177.6(10)	4409.1(9)	2785.2(8)	74.3(3)
O002	7383.4(12)	9513.1(10)	6405.8(8)	80.3(3)
N003	6469.0(10)	3494.6(9)	3677.5(8)	55.9(3)
N004	7570.5(10)	9855.4(9)	4997.5(8)	56.7(3)
C005	5200.0(13)	9022.8(11)	3747.0(11)	59.0(3)
C006	5652.9(13)	2586.1(11)	3672.2(11)	58.2(3)
C007	8839.6(13)	6311.0(11)	6689.8(10)	56.9(3)
C008	6475.5(12)	8868.4(10)	4236.6(10)	53.7(3)
C009	7293.9(12)	4456.0(11)	4698.3(10)	55.3(3)
C00A	6464.4(13)	3578.6(12)	2732.0(11)	59.0(3)
C00B	4421.1(15)	6963.3(11)	2662.1(10)	62.5(3)
C00C	4184.2(14)	8076.8(11)	2965.6(11)	63.6(3)
C00D	5788.1(16)	2591.0(13)	4751.5(12)	69.7(4)
C00E	6735.4(14)	7772.6(11)	3976.8(12)	63.5(3)

Table S4 Fractional Atomic Coordinates (×10^4) and Equivalent Isotropic Displacement Parameters (Å^2×10^3) for 2p. U_{eq} is defined as 1/3 of of the trace of the orthogonalised U_{IJ} tensor.

Atom	*x*	*y*	*z*	U(eq)
C00F	5557.8(13)	2638.3(12)	1703.8(11)	62.9(3)
C00G	6762.5(14)	5387.2(12)	5078.0(11)	66.4(3)
C00H	7527.2(14)	6301.6(12)	6059.2(12)	68.2(4)
C00I	7978.1(14)	10095.2(12)	6105.2(11)	63.5(3)
C00J	5719.1(16)	6833.3(11)	3189.3(12)	67.0(4)
C00K	9705.1(14)	7333.6(12)	7767.4(12)	66.3(3)
C00L	4802.6(14)	1748.4(12)	2716.7(12)	67.3(3)
C00M	8178.7(13)	10490.5(11)	4588.2(12)	61.1(3)
C00N	4721.6(14)	1748.3(13)	1697.9(12)	66.9(3)
C00O	9195.6(15)	11404.9(12)	5286.1(14)	71.6(4)
C00P	9360.3(14)	5372.2(13)	6273.2(13)	73.9(4)
C00Q	8604.8(14)	4456.9(13)	5292.4(13)	72.5(4)
C00R	9127.7(15)	11062.2(13)	6843.3(12)	72.8(4)
C00S	9705.0(14)	11728.6(13)	6438.7(14)	75.9(4)
C00T	7659.4(17)	10096.2(14)	3374.2(13)	76.1(4)
C00U	5510.2(17)	2655.3(17)	717.8(13)	82.1(4)
C00V	3300(2)	5929.1(13)	1771.0(12)	91.4(6)
C00W	8848.6(19)	8060.1(15)	8249.4(14)	87.1(5)
C00X	10601(2)	6904.0(18)	8608.0(15)	98.2(6)
C00Y	9650(2)	11308.0(18)	7965.3(15)	100.2(6)
C00Z	3831.5(18)	894.8(17)	679.9(15)	90.5(5)
C010	4635(2)	1810(2)	-259.6(14)	99.7(6)
C011	10592(2)	8094.1(17)	7522.4(18)	103.1(6)
C012	10784.5(18)	12672.2(17)	7186.9(19)	104.7(7)

Table S4 Fractional Atomic Coordinates (×10^4) and Equivalent Isotropic Displacement Parameters (Å^2×10^3) for 2p. U_{eq} is defined as 1/3 of of the trace of the orthogonalised U_{IJ} tensor.

Atom	*x*	*y*	*z*	U(eq)
C013	3805(2)	938(2)	-269.2(15)	105.0(6)
C014	10727(3)	12220(2)	8668.9(19)	130.7(9)
C015	11278(2)	12891(2)	8273(2)	132.1(10)
C2	2221(7)	6116(5)	1149(7)	166(6)
C4	4189(6)	5315(4)	931(4)	118(2)
C0AA	3349(8)	4969(5)	2205(5)	102(2)
C0AB	1976(4)	5993(4)	2118(5)	128(2)
C4A	3493(7)	4784(4)	1672(5)	122(2)
C2A	2802(9)	6097(7)	722(4)	184(5)

Table S5 Anisotropic Displacement Parameters (Å^2×10^3) for 2p. The Anisotropic displacement factor exponent takes the form: $-2\pi^2[h^2a^{*2}U_{11}+2hka^*b^*U_{12}+\ldots]$.

Atom	U_{11}	U_{22}	U_{33}	U_{23}	U_{13}	U_{12}
O001	75.3(6)	83.3(7)	67.2(6)	35.0(5)	29.4(5)	4.6(5)
O002	88.3(7)	88.2(7)	62.7(6)	35.1(5)	26.5(5)	4.0(6)
N003	55.1(5)	61.0(6)	54.2(6)	24.2(5)	23.5(5)	13.6(4)
N004	55.6(6)	54.4(5)	55.4(6)	20.3(5)	20.1(5)	4.8(4)
C005	64.1(7)	50.7(6)	59.0(7)	22.4(5)	21.5(6)	9.9(5)
C006	58.0(7)	59.9(7)	64.7(7)	28.8(6)	28.9(6)	17.8(5)
C007	56.6(7)	61.4(7)	56.8(7)	29.7(6)	22.4(5)	7.1(5)
C008	58.0(7)	51.4(6)	51.5(6)	20.5(5)	22.9(5)	5.8(5)
C009	54.9(6)	60.9(7)	54.2(7)	26.8(6)	22.9(5)	12.8(5)
C00A	54.1(6)	71.2(8)	58.7(7)	30.3(6)	25.7(6)	17.7(6)

Table S5 Anisotropic Displacement Parameters ($Å^2 \times 10^3$) for 2p. The Anisotropic displacement factor exponent takes the form: $-2\pi^2[h^2a^{*2}U_{11}+2hka^*b^*U_{12}+…]$.

Atom	U_{11}	U_{22}	U_{33}	U_{23}	U_{13}	U_{12}
C00B	79.1(9)	55.5(7)	46.4(6)	21.0(5)	19.9(6)	1.5(6)
C00C	63.0(7)	60.8(7)	58.8(7)	26.3(6)	14.7(6)	6.8(6)
C00D	78.2(9)	71.7(8)	70.5(9)	35.5(7)	35.4(7)	16.1(7)
C00E	64.0(7)	59.2(7)	69.9(8)	26.9(6)	28.7(6)	14.8(6)
C00F	56.9(7)	75.8(8)	55.5(7)	24.7(6)	22.9(6)	19.1(6)
C00G	57.9(7)	74.7(8)	59.3(7)	24.7(6)	16.2(6)	24.2(6)
C00H	67.4(8)	67.1(8)	63.2(8)	22.5(6)	20.8(6)	24.0(6)
C00I	62.5(7)	64.1(7)	58.7(7)	22.4(6)	20.9(6)	12.3(6)
C00J	86.3(9)	49.6(6)	67.7(8)	22.2(6)	35.5(7)	13.9(6)
C00K	63.1(7)	67.1(8)	62.7(8)	27.0(6)	20.1(6)	1.5(6)
C00L	65.5(8)	64.1(8)	71.6(9)	25.8(7)	28.8(7)	10.1(6)
C00M	59.7(7)	56.9(7)	71.1(8)	26.4(6)	30.5(6)	10.5(5)
C00N	59.8(7)	70.4(8)	65.1(8)	21.2(6)	24.8(6)	16.0(6)
C00O	65.6(8)	58.7(7)	88.5(10)	22.9(7)	36.5(8)	5.2(6)
C00P	51.3(7)	77.9(9)	77.5(9)	26.2(7)	14.1(6)	16.4(6)
C00Q	59.1(7)	71.1(8)	77.9(9)	23.1(7)	22.5(7)	23.2(6)
C00R	61.8(8)	70.4(8)	64.3(8)	11.1(7)	17.2(7)	12.1(6)
C00S	56.7(7)	62.0(8)	87.2(11)	7.4(7)	27.6(7)	6.9(6)
C00T	82.3(10)	81.8(9)	75.3(9)	38.4(8)	38.9(8)	7.6(7)
C00U	78.4(10)	104.9(12)	61.8(9)	32.5(8)	28.4(7)	14.7(9)
C00V	123.4(14)	62.4(8)	53.7(8)	18.9(7)	9.0(9)	-17.3(9)
C00W	89.7(11)	81.3(10)	69.0(9)	13.2(8)	26.9(8)	6.2(8)
C00X	92.3(12)	101.9(13)	68.7(10)	31.5(9)	3.0(9)	8.5(10)
C00Y	90.7(12)	106.1(13)	65.6(10)	9.3(9)	15.4(9)	12.0(10)
C00Z	81.1(10)	90.6(11)	74.3(10)	15.4(9)	23.5(8)	-0.2(9)

Table S5 Anisotropic Displacement Parameters (Å^2×10^3) for 2p. The Anisotropic displacement factor exponent takes the form: -2π^2[h^{2}a*^{2}U$_{11}$+2hka*b*U$_{12}$+…].

Atom	U11	U22	U33	U23	U13	U12
C010	98.3(13)	127.5(16)	56.9(9)	26.9(10)	24.9(9)	11.2(12)
C011	103.2(13)	89.8(12)	101.9(13)	29.1(10)	41.4(11)	-22.7(10)
C012	74.6(10)	79.9(11)	112.8(16)	-10.9(10)	37.6(10)	-7.0(8)
C013	97.4(13)	117.7(15)	59.8(10)	9.2(9)	17.4(9)	-2.5(11)
C014	100.1(15)	142(2)	72.7(12)	-12.9(13)	7.8(11)	2.2(15)
C015	83.2(13)	116.3(17)	109.5(18)	-30.2(14)	21.8(12)	-17.2(12)
C2	118(5)	84(3)	151(8)	35(4)	-83(6)	-19(4)
C4	161(5)	95(3)	55(2)	2(2)	30(3)	-10(3)
C0AA	110(4)	69(3)	82(4)	17(3)	12(3)	-39(3)
C0AB	89(2)	101(3)	146(4)	-1(3)	52(3)	-31(2)
C4A	118(4)	57.6(19)	116(4)	12(3)	-8(3)	5.0(19)
C2A	257(10)	143(5)	58(2)	42(3)	-18(3)	-92(6)

Table S6 Bond Lengths for 2p.

Atom	Atom	Length/Å	Atom	Atom	Length/Å
O001	C00A	1.2269(16)	C00I	C00R	1.461(2)
O002	C00I	1.2259(17)	C00K	C00W	1.526(2)
N003	C006	1.4011(16)	C00K	C00X	1.531(2)
N003	C009	1.4494(16)	C00K	C011	1.529(2)
N003	C00A	1.3896(17)	C00L	C00N	1.427(2)
N004	C008	1.4482(15)	C00M	C00O	1.3415(19)
N004	C00I	1.3923(17)	C00M	C00T	1.496(2)
N004	C00M	1.3999(17)	C00N	C00Z	1.409(2)
C005	C008	1.3722(18)	C00O	C00S	1.426(2)

Table S6 Bond Lengths for 2p.

Atom	Atom	Length/Å	Atom	Atom	Length/Å
C005	C00C	1.3845(18)	C00P	C00Q	1.380(2)
C006	C00D	1.4931(19)	C00R	C00S	1.403(2)
C006	C00L	1.3440(19)	C00R	C00Y	1.402(2)
C007	C00H	1.3838(19)	C00S	C012	1.407(2)
C007	C00K	1.5333(19)	C00U	C010	1.370(2)
C007	C00P	1.385(2)	C00V	C2	1.283(5)
C008	C00E	1.3747(18)	C00V	C4	1.813(6)
C009	C00G	1.3710(18)	C00V	C0AA	1.530(6)
C009	C00Q	1.3722(19)	C00V	C0AB	1.690(5)
C00A	C00F	1.4637(19)	C00V	C4A	1.434(5)
C00B	C00C	1.387(2)	C00V	C2A	1.491(5)
C00B	C00J	1.392(2)	C00Y	C014	1.376(3)
C00B	C00V	1.5311(19)	C00Z	C013	1.366(3)
C00E	C00J	1.3822(19)	C010	C013	1.372(3)
C00F	C00N	1.396(2)	C012	C015	1.363(4)
C00F	C00U	1.399(2)	C014	C015	1.380(4)
C00G	C00H	1.382(2)			

Table S7 Bond Angles for 2p.

Atom	Atom	Atom	Angle/°	Atom	Atom	Atom	Angle/°
C006	N003	C009	119.96(10)	C00X	C00K	C007	110.65(12)
C00A	N003	C006	123.28(11)	C011	C00K	C007	107.35(13)
C00A	N003	C009	116.58(10)	C011	C00K	C00X	109.26(15)
C00I	N004	C008	117.15(10)	C006	C00L	C00N	121.54(13)

Table S7 Bond Angles for 2p.

Atom	Atom	Atom	Angle/°	Atom	Atom	Atom	Angle/°
C00I	N004	C00M	123.84(11)	N004	C00M	C00T	117.46(11)
C00M	N004	C008	118.99(10)	C00O	C00M	N004	119.46(13)
C008	C005	C00C	119.88(12)	C00O	C00M	C00T	123.08(13)
N003	C006	C00D	117.50(12)	C00F	C00N	C00L	118.71(13)
C00L	C006	N003	119.76(12)	C00F	C00N	C00Z	118.07(15)
C00L	C006	C00D	122.75(13)	C00Z	C00N	C00L	123.22(15)
C00H	C007	C00K	122.04(12)	C00M	C00O	C00S	121.68(14)
C00H	C007	C00P	116.62(12)	C00Q	C00P	C007	122.04(12)
C00P	C007	C00K	121.27(12)	C009	C00Q	C00P	120.08(13)
C005	C008	N004	120.61(11)	C00S	C00R	C00I	120.78(14)
C005	C008	C00E	120.08(11)	C00Y	C00R	C00I	118.72(17)
C00E	C008	N004	119.27(11)	C00Y	C00R	C00S	120.50(16)
C00G	C009	N003	119.64(11)	C00R	C00S	C00O	118.66(13)
C00G	C009	C00Q	119.13(12)	C00R	C00S	C012	118.54(18)
C00Q	C009	N003	121.22(11)	C012	C00S	C00O	122.78(18)
O001	C00A	N003	120.44(12)	C010	C00U	C00F	120.53(17)
O001	C00A	C00F	123.67(12)	C00B	C00V	C4	98.4(2)
N003	C00A	C00F	115.88(12)	C00B	C00V	C0AB	106.40(18)
C00C	C00B	C00J	117.21(12)	C2	C00V	C00B	119.1(3)
C00C	C00B	C00V	120.99(14)	C2	C00V	C4	106.7(6)
C00J	C00B	C00V	121.80(13)	C2	C00V	C0AA	124.7(5)
C005	C00C	C00B	121.49(13)	C0AA	C00V	C00B	106.8(2)
C008	C00E	C00J	119.68(13)	C0AA	C00V	C4	94.5(4)
C00N	C00F	C00A	120.78(12)	C4A	C00V	C00B	118.5(3)
C00N	C00F	C00U	120.01(14)	C4A	C00V	C0AB	101.5(4)
C00U	C00F	C00A	119.19(14)	C4A	C00V	C2A	114.8(4)

Table S7 Bond Angles for 2p.

Atom	Atom	Atom	Angle/°	Atom	Atom	Atom	Angle/°
C009	C00G	C00H	120.40(12)	C2A	C00V	C00B	109.9(3)
C00G	C00H	C007	121.70(13)	C2A	C00V	C0AB	103.9(5)
O002	C00I	N004	120.28(12)	C014	C00Y	C00R	119.3(2)
O002	C00I	C00R	124.26(13)	C013	C00Z	C00N	120.51(18)
N004	C00I	C00R	115.47(13)	C00U	C010	C013	119.64(18)
C00E	C00J	C00B	121.61(12)	C015	C012	C00S	119.8(2)
C00W	C00K	C007	111.95(12)	C00Z	C013	C010	121.24(17)
C00W	C00K	C00X	108.73(15)	C00Y	C014	C015	120.2(2)
C00W	C00K	C011	108.85(15)	C012	C015	C014	121.62(19)

Table S8 Hydrogen Atom Coordinates (Å×10^4) and Isotropic Displacement Parameters (Å^2×10^3) for 2p.

Atom	*x*	*y*	*z*	U(eq)
H005	5018.14	9761.64	3939.83	71
H00C	3323.59	8190.41	2636.78	76
H00F	6662.86	2471.07	5125.71	105
H00G	5110.2	1981.37	4639.92	105
H00H	5678.19	3323.83	5182.83	105
H00E	7590.93	7663.78	4329.12	76
H00I	5882.03	5403.57	4672.66	80
H00K	7150.15	6926.78	6302.17	82
H00J	5907.17	6096.35	3007.22	80
H00L	4254.08	1154.88	2719.78	81
H00O	9578.18	11838.74	5011.36	86
H00P	10246.4	5358.26	6666.64	89

Table S8 Hydrogen Atom Coordinates (Å×10^4) and Isotropic Displacement Parameters (Å^2×10^3) for 2p.

Atom	*x*	*y*	*z*	U(eq)
H00Q	8984.59	3840.06	5033.79	87
H00A	7699.2	9297.55	3061.13	114
H00B	8201.48	10559.22	3197.17	114
H00D	6744.05	10178.65	3085.13	114
H00U	6076.77	3244.61	725.67	99
H00M	8340.1	8390.34	7755.55	131
H00N	9422.85	8669.92	8936.55	131
H00R	8243.85	7577.12	8359.31	131
H00S	10047.6	6402.93	8743.45	147
H00T	11133.19	7554.85	9276.7	147
H00V	11183.37	6484.7	8331.09	147
H00Y	9272.86	10859.29	8231.04	120
H00Z	3257.4	297.03	653.57	109
H010	4604.16	1828.17	-912.85	120
H01A	11132.05	7643.31	7215.43	155
H01B	11166.87	8733.05	8186.49	155
H01C	10033.26	8383.36	7011.34	155
H012	11160.07	13145.3	6940.56	126
H013	3213.44	366.2	-934.1	126
H014	11086.18	12385.74	9414.02	157
H015	12002.93	13506.69	8760	159
H2A	1642.45	6311.02	1523.65	249
H2B	1765.69	5430.93	493.64	249
H2C	2446.43	6745.42	965.48	249
H4A	4383.54	5848.36	632.39	177

Table S8 Hydrogen Atom Coordinates (Å×10^4) and Isotropic Displacement Parameters (Å^2×10^3) for 2p.

Atom	*x*	*y*	*z*	U(eq)
H4B	3638.21	4598.94	347.13	177
H4C	5014.52	5173.36	1369.31	177
H0AA	4249.84	4843.21	2455.52	153
H0AB	2750	4266.44	1632.26	153
H0AC	3078.55	5194.63	2801.01	153
H0AD	2257.9	6032.12	2850.62	192
H0AE	1279.48	5313.67	1625.38	192
H0AF	1638.08	6670.31	2075.17	192
H4AA	4183.66	4616.14	1412.36	183
H4AB	2667.52	4232.02	1163.82	183
H4AC	3756.21	4736.33	2372.03	183
H2AA	2559.07	6837.99	840.08	277
H2AB	2028.57	5495.91	191.84	277
H2AC	3497.88	6065.22	460.12	277

Table S9 Atomic Occupancy for 2p.

Atom	*Occupancy*	Atom	*Occupancy*	Atom	*Occupancy*
C2	0.438(5)	H2A	0.438(5)	H2B	0.438(5)
H2C	0.438(5)	C4	0.438(5)	H4A	0.438(5)
H4B	0.438(5)	H4C	0.438(5)	C0AA	0.438(5)
H0AA	0.438(5)	H0AB	0.438(5)	H0AC	0.438(5)
C0AB	0.562(5)	H0AD	0.562(5)	H0AE	0.562(5)
H0AF	0.562(5)	C4A	0.562(5)	H4AA	0.562(5)
H4AB	0.562(5)	H4AC	0.562(5)	C2A	0.562(5)

Table S9 Atomic Occupancy for 2p.

Atom	*Occupancy*	Atom	*Occupancy*	Atom	*Occupancy*
H2AA	0.562(5)	H2AB	0.562(5)	H2AC	0.562(5)

Product 3t:

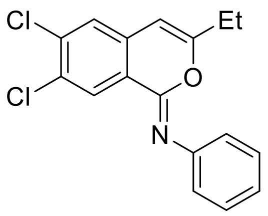

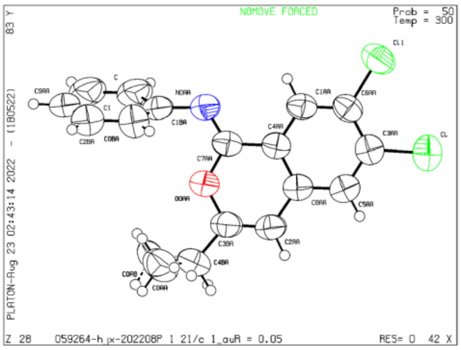

Table S10 Crystal data and structure refinement for 3t.

Identification code	3t
Empirical formula	$C_{17}H_{13}Cl_2NO$
Formula weight	318.18
Temperature/K	300.33(10)
Crystal system	monoclinic
Space group	$P2_1/c$
a/Å	6.9460(2)
b/Å	12.2329(4)
c/Å	18.1948(5)
α/°	90
β/°	98.067(3)
γ/°	90
Volume/Å^3	1530.72(8)
Z	4
ρ_{calc}g/cm^3	1.381
μ/mm^{-1}	3.787
F(000)	656.0
Crystal size/mm^3	0.3 × 0.2 × 0.2
Radiation	Cu Kα (λ = 1.54184)
2Θ range for data collection/°	8.738 to 133.192

Index ranges	$-8 \le h \le 8$, $-12 \le k \le 14$, $-21 \le l \le 21$
Reflections collected	8110
Independent reflections	2604 [R_{int} = 0.0332, R_{sigma} = 0.0303]
Data/restraints/parameters	2604/1/203
Goodness-of-fit on F^2	1.090
Final R indexes [I>=2σ (I)]	R_1 = 0.0489, wR_2 = 0.1293
Final R indexes [all data]	R_1 = 0.0629, wR_2 = 0.1460
Largest diff. peak/hole / e $Å^{-3}$	0.17/-0.26

Table S11 Fractional Atomic Coordinates (×10^4) and Equivalent Isotropic Displacement Parameters ($Å^2$×10^3) for 3t. U_{eq} is defined as 1/3 of of the trace of the orthogonalised U_{IJ} tensor.

Atom	*x*	*y*	*z*	U(eq)
Cl	2902.7(13)	2513.8(7)	4680.1(5)	100.3(4)
Cl1	3331.8(15)	2162.7(7)	6415.3(5)	105.3(4)
O0AA	2093(3)	-2804.1(17)	5421.7(10)	81.5(6)
N0AA	2472(4)	-2159(2)	6606.3(13)	81.9(7)
C1AA	2859(4)	35(2)	6093.6(15)	71.9(7)
C2AA	2035(4)	-1651(3)	4380.7(15)	76.9(8)
C3AA	2775(4)	1227(2)	5050.0(17)	73.2(7)
C4AA	2539(4)	-862(2)	5622.0(14)	65.6(7)
C	755(7)	-3573(4)	7162.9(18)	113.6(13)
C5AA	2475(4)	348(3)	4577.2(15)	73.3(7)
C6AA	2968(4)	1066(2)	5813.9(16)	74.1(7)
C7AA	2384(4)	-1962(2)	5921.4(15)	70.0(7)
C8AA	2351(4)	-709(2)	4853.2(14)	67.4(7)
C9AA	2212(11)	-5345(4)	7413(2)	137(2)
C0BA	3864(7)	-3975(3)	6837(2)	108.8(12)

Table S11 Fractional Atomic Coordinates (×10⁴) and Equivalent Isotropic Displacement Parameters (Å²×10³) for 3t. U_{eq} is defined as 1/3 of of the trace of the orthogonalised U_{IJ} tensor.

Atom	*x*	*y*	*z*	U(eq)
C1BA	2353(5)	-3248(3)	6856.1(14)	84.5(9)
C2BA	3787(9)	-5021(4)	7110(2)	125.8(15)
C3BA	1915(5)	-2636(3)	4661.8(17)	83.3(9)
C4BA	1544(8)	-3682(3)	4253(2)	125.7(15)
C1	692(9)	-4645(5)	7439(2)	141(2)
C0AA	2844(11)	-4526(5)	4439(3)	126(3)
C0AB	810(30)	-4564(11)	4549(9)	105(7)

Table S12 Anisotropic Displacement Parameters (Å²×10³) for 3t. The Anisotropic displacement factor exponent takes the form: $-2\pi^2[h^2a^{*2}U_{11}+2hka^*b^*U_{12}+\ldots]$.

Atom	U_{11}	U_{22}	U_{33}	U_{23}	U_{13}	U_{12}
Cl	100.1(7)	85.3(6)	116.2(7)	14.2(4)	17.4(5)	3.5(4)
Cl1	126.6(8)	86.2(6)	103.8(6)	-26.0(4)	19.0(5)	-10.0(5)
O0AA	101.1(15)	77.3(12)	65.7(11)	-9.7(9)	10.7(10)	-5.5(10)
N0AA	101.1(18)	81.9(16)	64.7(13)	-6.2(11)	19.4(12)	-8.8(13)
C1AA	67.5(16)	82(2)	67.7(15)	-10.6(13)	14.1(12)	-2.1(13)
C2AA	81.5(19)	88(2)	59.9(14)	-6.4(14)	5.9(13)	8.0(15)
C3AA	58.4(15)	75.1(18)	87.1(18)	0.2(14)	13.3(13)	5.5(13)
C4AA	55.1(14)	73.9(17)	68.5(15)	-5.6(12)	11.8(11)	2.1(12)
C	153(3)	124(3)	72.6(19)	-10(2)	46(2)	-27(3)
C5AA	62.5(16)	88(2)	69.8(15)	1.4(14)	10.1(12)	7.0(14)
C6AA	67.7(17)	72.6(18)	83.3(18)	-13.4(14)	14.9(13)	1.2(13)

Table S12 Anisotropic Displacement Parameters (Å^2×10^3) for 3t. The Anisotropic displacement factor exponent takes the form: -2π^2[h^{2}a*^{2}U$_{11}$+2hka*b*U$_{12}$+…].

Atom	U_{11}	U_{22}	U_{33}	U_{23}	U_{13}	U_{12}
C7AA	64.6(16)	79.5(18)	66.4(15)	-11.8(13)	11.4(12)	-1.7(13)
C8AA	55.2(14)	80.8(18)	66.5(14)	-4.8(13)	9.5(11)	5.4(12)
C9AA	264(7)	92(3)	56.2(18)	-10.2(18)	24(3)	-34(4)
C0BA	137(3)	86(3)	103(3)	3.2(19)	16(2)	-10(2)
C1BA	112(2)	89(2)	53.1(14)	-8.9(14)	12.6(15)	-13.6(19)
C2BA	185(5)	99(3)	89(3)	-2(2)	7(3)	-4(3)
C3BA	94(2)	89(2)	66.4(16)	-13.4(15)	8.7(14)	3.6(17)
C4BA	203(5)	86(3)	84(2)	-23(2)	8(3)	1(3)
C1	214(6)	142(4)	82(2)	-14(3)	68(3)	-63(4)
C0AA	162(7)	96(4)	117(4)	-21(3)	6(4)	24(4)
C0AB	137(16)	63(9)	113(11)	-16(7)	9(10)	-13(8)

Table S13 Bond Lengths for 3t.

Atom	Atom	Length/Å	Atom	Atom	Length/Å
Cl	C3AA	1.719(3)	C4AA	C7AA	1.462(4)
Cl1	C6AA	1.727(3)	C4AA	C8AA	1.399(4)
O0AA	C7AA	1.370(3)	C	C1BA	1.370(5)
O0AA	C3BA	1.386(4)	C	C1	1.406(7)
N0AA	C7AA	1.262(3)	C5AA	C8AA	1.394(4)
N0AA	C1BA	1.414(4)	C9AA	C2BA	1.351(7)
C1AA	C4AA	1.391(4)	C9AA	C1	1.365(7)
C1AA	C6AA	1.366(4)	C0BA	C1BA	1.379(5)
C2AA	C8AA	1.436(4)	C0BA	C2BA	1.377(5)

Table S13 Bond Lengths for 3t.

Atom	Atom	Length/Å	Atom	Atom	Length/Å
C2AA	C3BA	1.316(4)	C3BA	C4BA	1.485(5)
C3AA	C5AA	1.374(4)	C4BA	C0AA	1.382(7)
C3AA	C6AA	1.392(4)	C4BA	C0AB	1.338(13)

Table S14 Bond Angles for 3t.

Atom	Atom	Atom	Angle/°	Atom	Atom	Atom	Angle/°
C7AA	O0AA	C3BA	122.3(2)	N0AA	C7AA	C4AA	123.2(2)
C7AA	N0AA	C1BA	120.0(2)	C4AA	C8AA	C2AA	118.4(3)
C6AA	C1AA	C4AA	120.7(3)	C5AA	C8AA	C2AA	122.7(2)
C3BA	C2AA	C8AA	121.0(3)	C5AA	C8AA	C4AA	118.8(2)
C5AA	C3AA	Cl	118.9(2)	C2BA	C9AA	C1	120.3(4)
C5AA	C3AA	C6AA	120.0(3)	C2BA	C0BA	C1BA	121.5(4)
C6AA	C3AA	Cl	121.2(2)	C	C1BA	N0AA	119.5(4)
C1AA	C4AA	C7AA	120.7(2)	C	C1BA	C0BA	119.1(4)
C1AA	C4AA	C8AA	119.7(3)	C0BA	C1BA	N0AA	121.4(3)
C8AA	C4AA	C7AA	119.6(2)	C9AA	C2BA	C0BA	119.6(5)
C1BA	C	C1	118.9(4)	O0AA	C3BA	C4BA	110.9(3)
C3AA	C5AA	C8AA	120.8(3)	C2AA	C3BA	O0AA	121.5(3)
C1AA	C6AA	Cl1	119.5(2)	C2AA	C3BA	C4BA	127.6(3)
C1AA	C6AA	C3AA	120.0(3)	C0AA	C4BA	C3BA	117.4(4)
C3AA	C6AA	Cl1	120.5(2)	C0AB	C4BA	C3BA	122.8(7)
O0AA	C7AA	C4AA	117.2(2)	C9AA	C1	C	120.6(4)
N0AA	C7AA	O0AA	119.6(3)				

Table S15 Torsion Angles for 3t.

A	B	C	D	Angle/°	A	B	C	D	Angle/°
Cl	C3AA	C5AA	C8AA	-179.6(2)	C7AA	N0AA	C1BA	C	-112.0(3)
Cl	C3AA	C6AA	Cl1	0.3(3)	C7AA	N0AA	C1BA	C0BA	71.8(4)
Cl	C3AA	C6AA	C1AA	179.9(2)	C7AA	C4AA	C8AA	C2AA	-0.8(4)
O0AA	C3BA	C4BA	C0AA	-54.4(6)	C7AA	C4AA	C8AA	C5AA	179.1(2)
O0AA	C3BA	C4BA	C0AB	21.7(12)	C8AA	C2AA	C3BA	O0AA	-0.1(5)
C1AA	C4AA	C7AA	O0AA	-179.2(2)	C8AA	C2AA	C3BA	C4BA	178.3(4)
C1AA	C4AA	C7AA	N0AA	2.1(4)	C8AA	C4AA	C7AA	O0AA	1.2(4)
C1AA	C4AA	C8AA	C2AA	179.6(2)	C8AA	C4AA	C7AA	N0AA	-177.5(3)
C1AA	C4AA	C8AA	C5AA	-0.5(4)	C1BA	N0AA	C7AA	O0AA	2.7(4)
C2AA	C3BA	C4BA	C0AA	127.1(5)	C1BA	N0AA	C7AA	C4AA	-178.6(3)
C2AA	C3BA	C4BA	C0AB	-156.8(11)	C1BA	C	C1	C9AA	1.1(6)
C3AA	C5AA	C8AA	C2AA	179.9(2)	C1BA	C0BA	C2BA	C9AA	-0.7(6)
C3AA	C5AA	C8AA	C4AA	0.0(4)	C2BA	C9AA	C1	C	-1.0(7)
C4AA	C1AA	C6AA	Cl1	179.1(2)	C2BA	C0BA	C1BA	N0AA	177.1(3)
C4AA	C1AA	C6AA	C3AA	-0.5(4)	C2BA	C0BA	C1BA	C	0.8(5)
C5AA	C3AA	C6AA	Cl1	-179.6(2)	C3BA	O0AA	C7AA	N0AA	177.7(3)
C5AA	C3AA	C6AA	C1AA	0.0(4)	C3BA	O0AA	C7AA	C4AA	-1.0(4)
C6AA	C1AA	C4AA	C7AA	-178.8(2)	C3BA	C2AA	C8AA	C4AA	0.3(4)
C6AA	C1AA	C4AA	C8AA	0.8(4)	C3BA	C2AA	C8AA	C5AA	-179.6(3)
C6AA	C3AA	C5AA	C8AA	0.3(4)	C1	C	C1BA	N0AA	-177.3(3)
C7AA	O0AA	C3BA	C2AA	0.5(5)	C1	C	C1BA	C0BA	-1.0(5)
C7AA	O0AA	C3BA	C4BA	-178.1(3)	C1	C9AA	C2BA	C0BA	0.8(6)

Table S16 Hydrogen Atom Coordinates (Å×10^4) and Isotropic Displacement Parameters (Å^2×10^3) for 3t.

Atom	*x*	*y*	*z*	U(eq)
H1AA	2999.53	-68.86	6604.68	86
H2AA	1912.28	-1563.24	3868.54	92
H	-271.67	-3093.39	7188.55	136
H5AA	2353.25	460.22	4067.25	88
H9AA	2162.55	-6048.12	7603.56	165
H0BA	4957.92	-3753.33	6634.84	131
H2BA	4814.19	-5501.7	7087.36	151
H4BA	260.88	-3936.73	4324.12	151
H4BB	1509.34	-3531.35	3727.85	151
H4BC	678.22	-3514.59	3801.11	151
H4BD	2771.08	-3904.94	4102.55	151
H1	-394.63	-4878.75	7640.73	170
H0AA	4126.54	-4294.49	4369.26	190
H0AB	2472.15	-5146.24	4127.71	190
H0AC	2836.6	-4724.39	4949.07	190
H0AD	1598.56	-4742.09	5010.41	158
H0AE	803.21	-5168.84	4213.05	158
H0AF	-493.13	-4412.51	4637.25	158

Table S17 Atomic Occupancy for 3t.

Atom	*Occupancy*	Atom	*Occupancy*	Atom	*Occupancy*
H4BA	0.757(9)	H4BB	0.757(9)	H4BC	0.243(9)
H4BD	0.243(9)	C0AA	0.757(9)	H0AA	0.757(9)
H0AB	0.757(9)	H0AC	0.757(9)	C0AB	0.243(9)

Table S17 Atomic Occupancy for 3t.

Atom	*Occupancy*	Atom	*Occupancy*	Atom	*Occupancy*
H0AD	0.243(9)	H0AE	0.243(9)	H0AF	0.243(9)

IV. Supplementary References

1. Yang, G., Shen, C. & Zhang, W. An Asymmetric Aerobic Aza-Wacker-Type Cyclization: Synthesis of Isoindolinones Bearing Tetrasubstituted Carbon Stereocenters. *Angew. Chem., Int. Ed.* **51**, 9141-9145, (2012).
2. Xu, C. & Shen, Q. Lewis Acid Mediated Trifluoromethylthio Lactonization/Lactamization. *Org. Lett.* **17**, 4561-4563, (2015).
3. Zhang, W., Chen, P. & Liu, G. Enantioselective Palladium(II)-Catalyzed Intramolecular Aminoarylation of Alkenes by Dual N−H and Aryl C−H Bond Cleavage. *Angew. Chem., Int. Ed.* **56**, 5336-5340, (2017).
4. Liu, H., Deng, X., Huang, X., Ji, N. & He, W. Study on the ArI-catalyzed intramolecular oxy-cyclization of 2-alkenylbenzamides to benzoiminolactones. *Org. Biomol. Chem.* **18**, 3654-3658, (2020).
5. Naves, Y. R. Etudes sur les matières végétales volatiles XXIV. Composition de l'huile essentielle et du résinoïde de livèche (Levisticum officinale Koch). *Helv. Chim. Acta*. **26**, 1281-1295, (1943).
6. Kerins, F. & O'Shea, D. F. Generation of Substituted Styrenes via Suzuki Cross-Coupling of Aryl Halides with 2,4,6-Trivinylcyclotriboroxane. *J. Org. Chem.* **67**, 4968-4971, (2002).
7. Yang, S., Zhu, S.-F., Guo, N., Song, S. & Zhou, Q.-L. Carboxy-directed asymmetric hydrogenation of α-alkyl-α-aryl terminal olefins: highly enantioselective and chemoselective access to a chiral benzylmethyl center. *Org. Biomol. Chem.* **12**, 2049-2052, (2014).
8. Hemric, B. N., Shen, K. & Wang, Q. Copper-Catalyzed Amino Lactonization and Amino Oxygenation of Alkenes Using O-Benzoylhydroxylamines. *J. Am. Chem. Soc.* **138**, 5813-5816, (2016).
