# Supplementary material for: Chemoselective cycloisomerization of O-alkenylbenzamides via concomitant 1,2-aryl migration/elimination mediated by hypervalent iodine reagents
Source: Commun Chem. 2023 Jun 17;6:126. doi: 10.1038/s42004-023-00930-5 (PMC10276869; doi:10.1038/s42004-023-00930-5)
Supplement: Supplementary file 3 — Description of Additional Supplementary File [file 42004_2023_930_MOESM3_ESM.pdf]

Description of Additional Supplementary Files

File name: Supplementary Data 1

Description: ^{1}H and ^{13}C NMR Spectra of Substrates and Products

File name: Supplementary Data 2

Description: X-ray data file of compound **2p** (CCDC 2201660)

File name: Supplementary Data 3

Description: X-ray data file of compound **3t** (CCDC 2202945)

File name: Supplementary Data 4

Description: DFT calculation data
