# Supplementary figures and images for: Chemoselective cycloisomerization of O-alkenylbenzamides via concomitant 1,2-aryl migration/elimination mediated by hypervalent iodine reagents
Source: Commun Chem. 2023 Jun 17;6:126. doi: 10.1038/s42004-023-00930-5 (PMC10276869; doi:10.1038/s42004-023-00930-5)

**1H and 13C NMR Spectra of Substrates and Products**


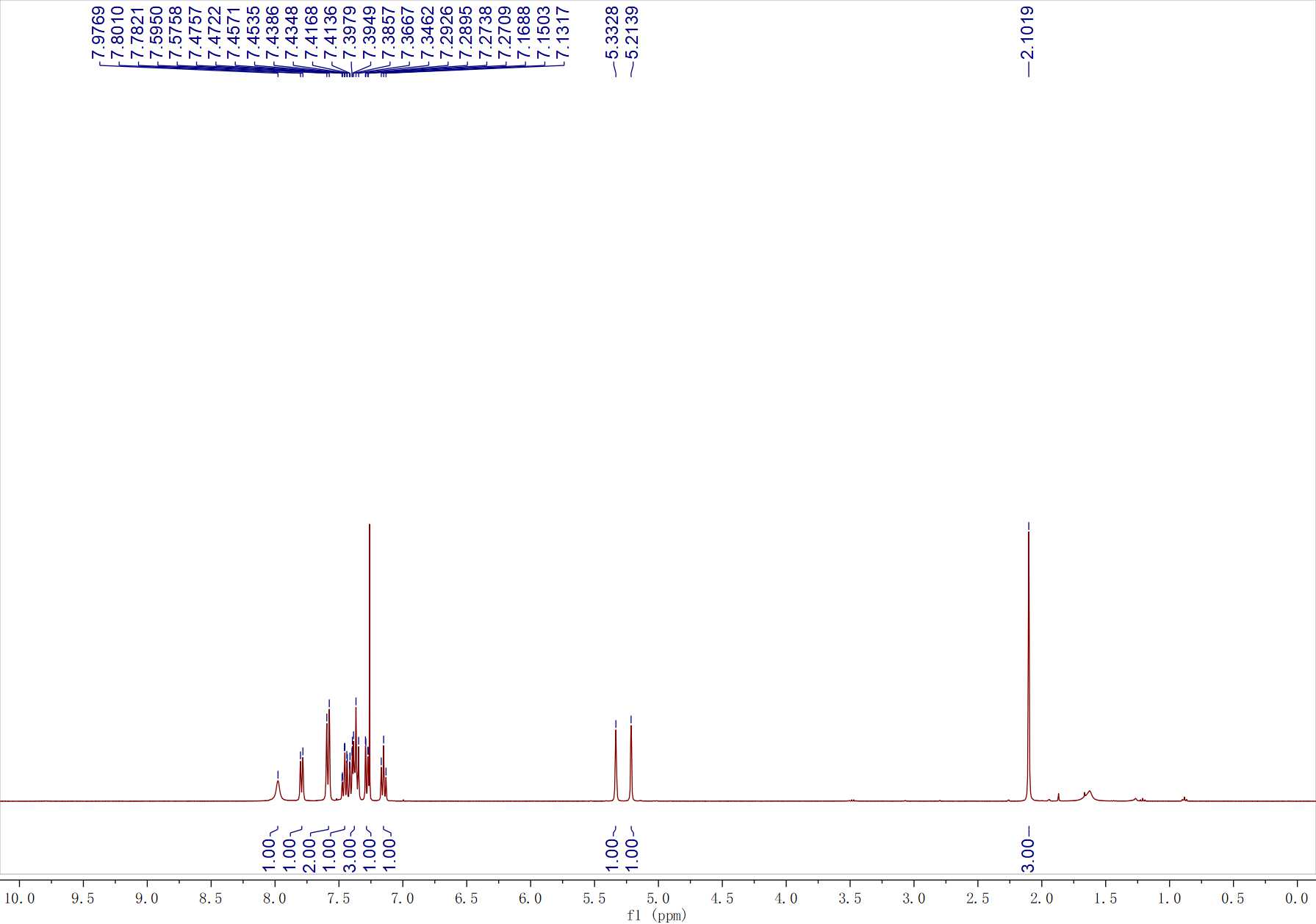


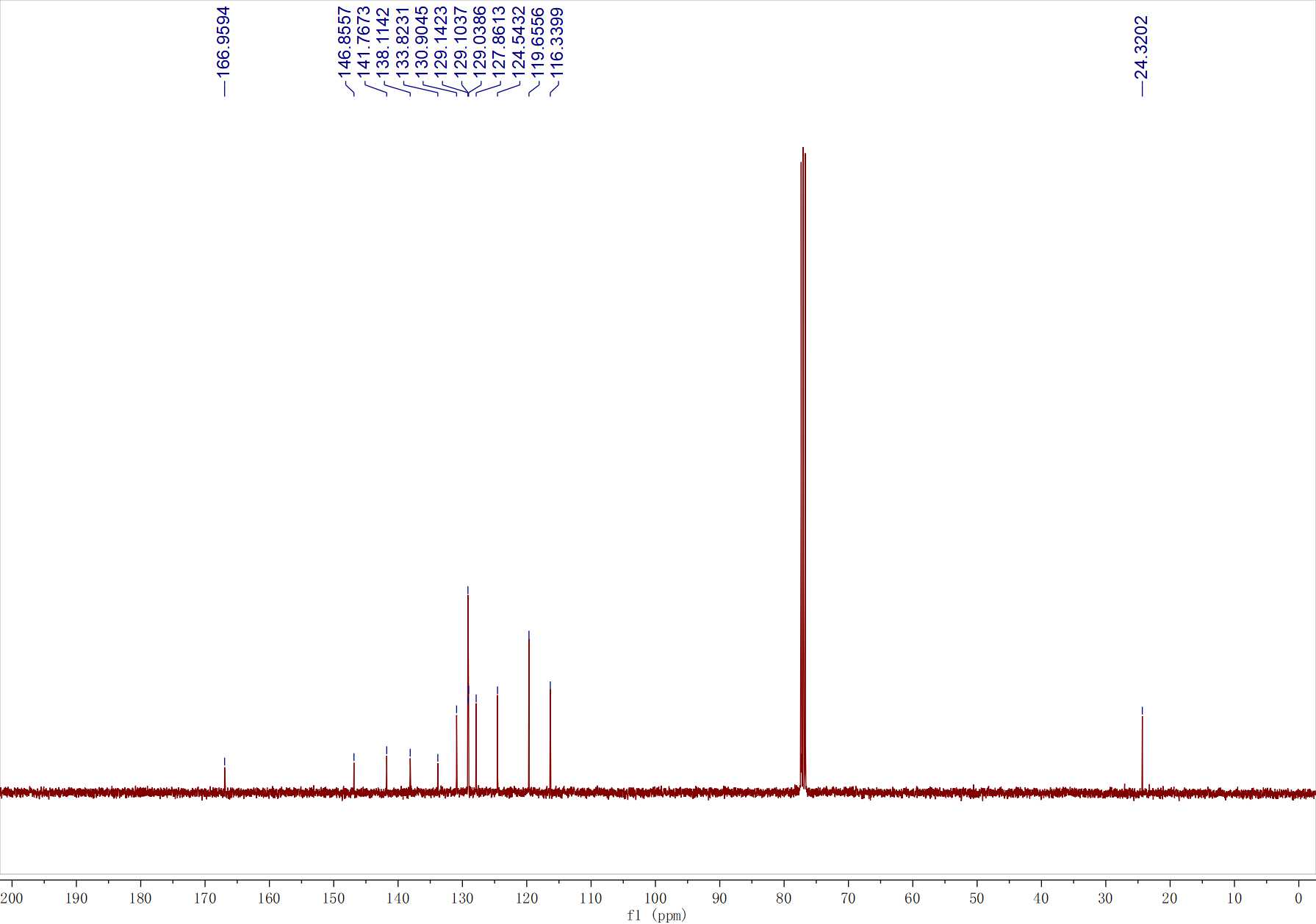


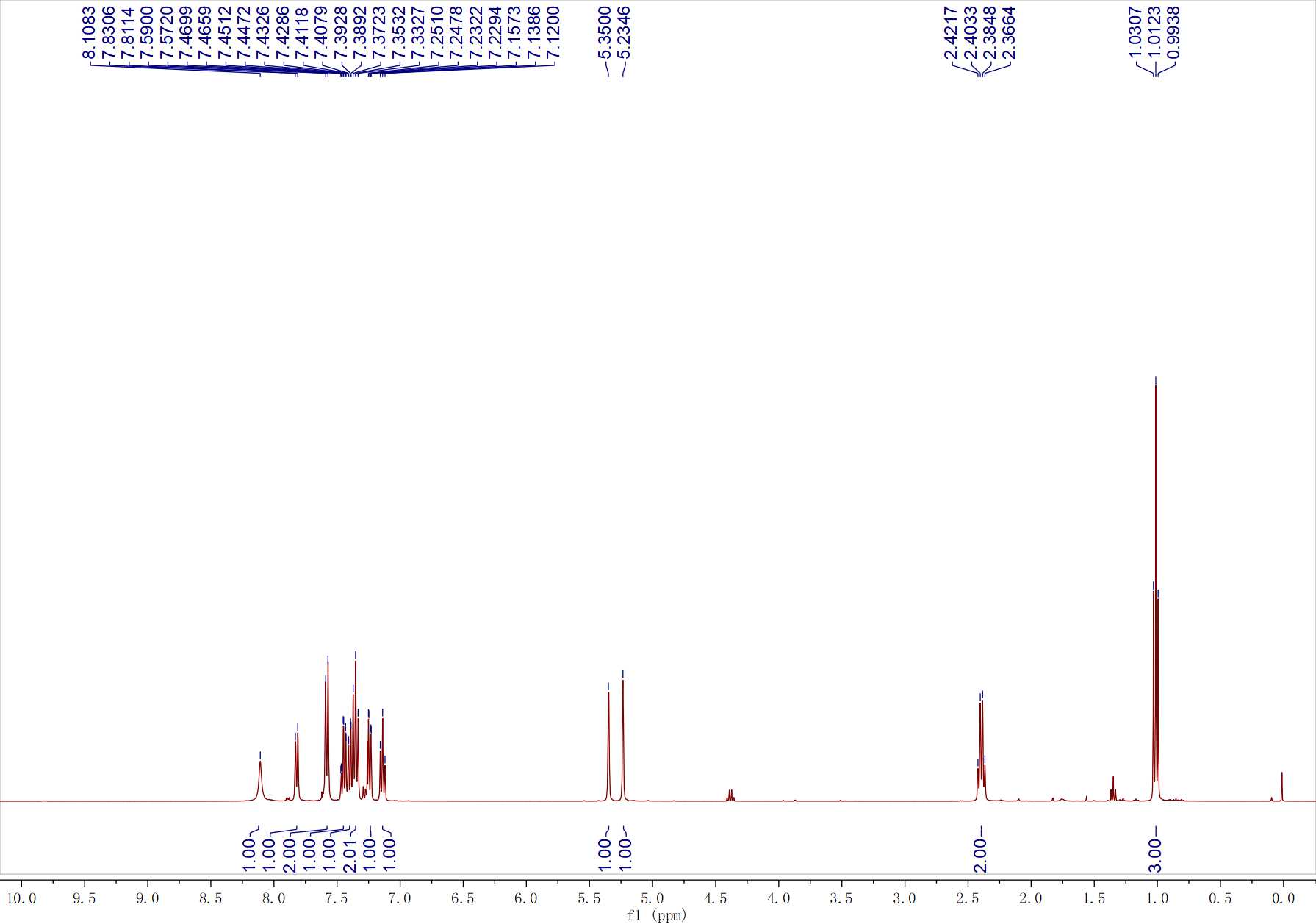

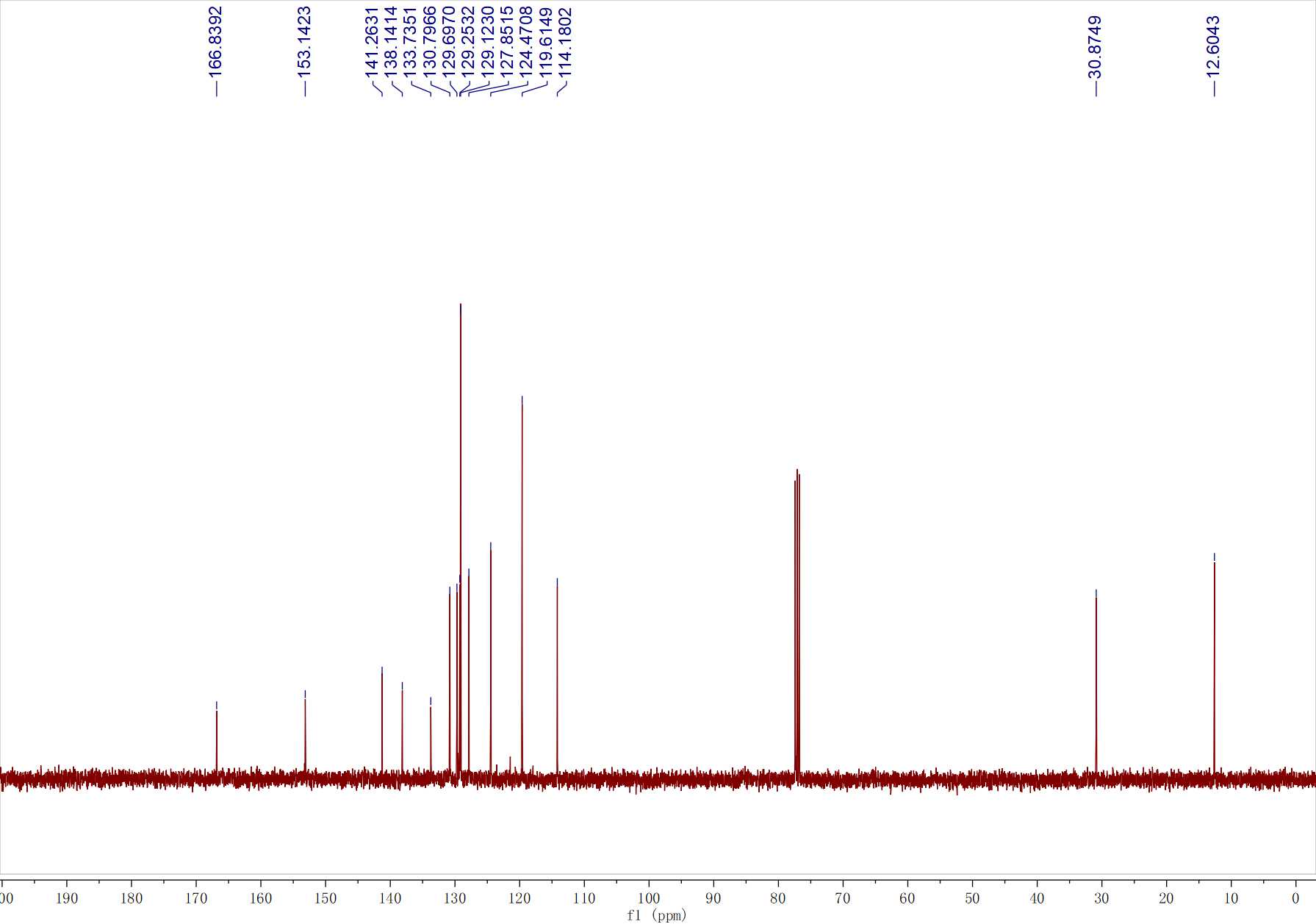


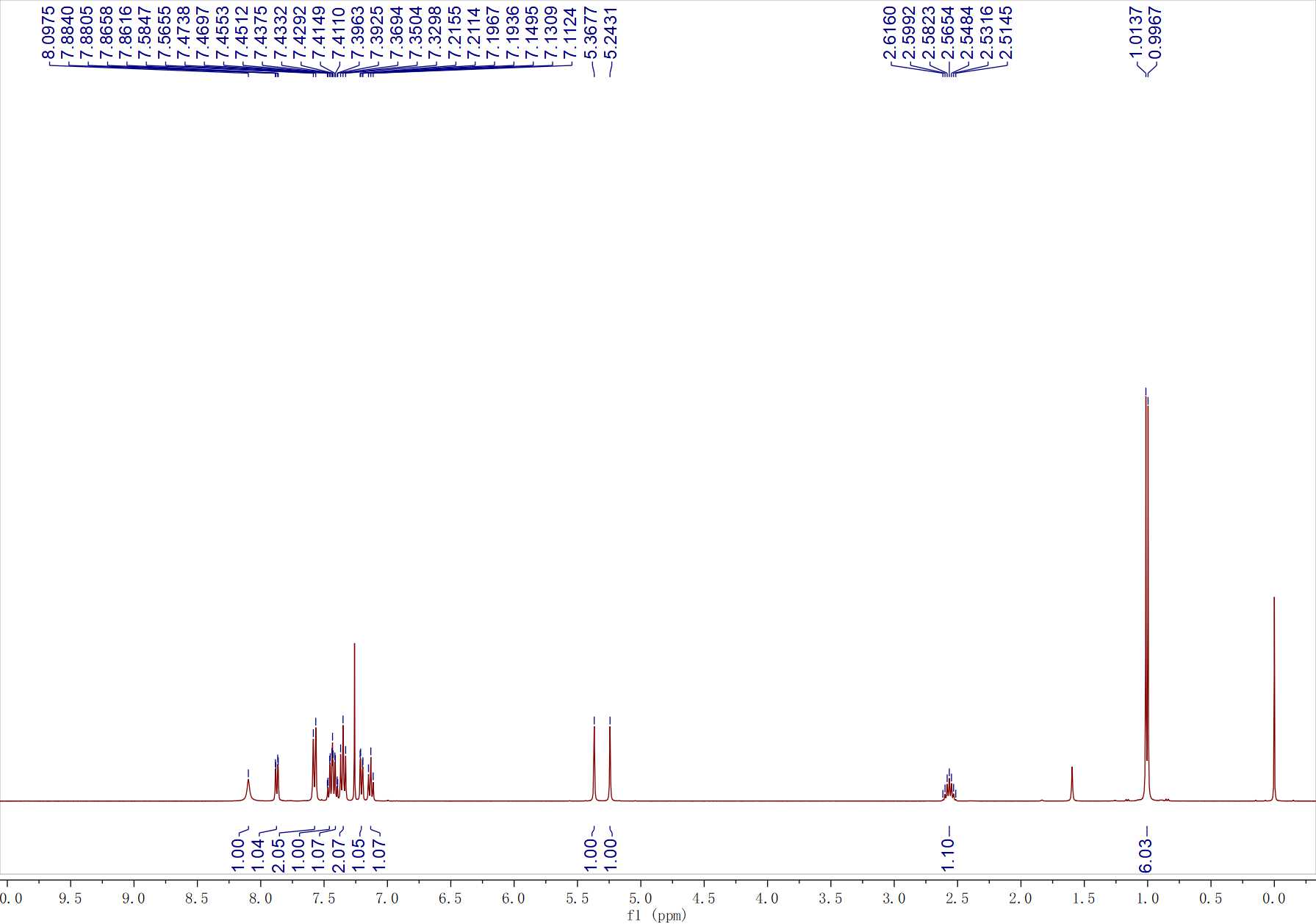


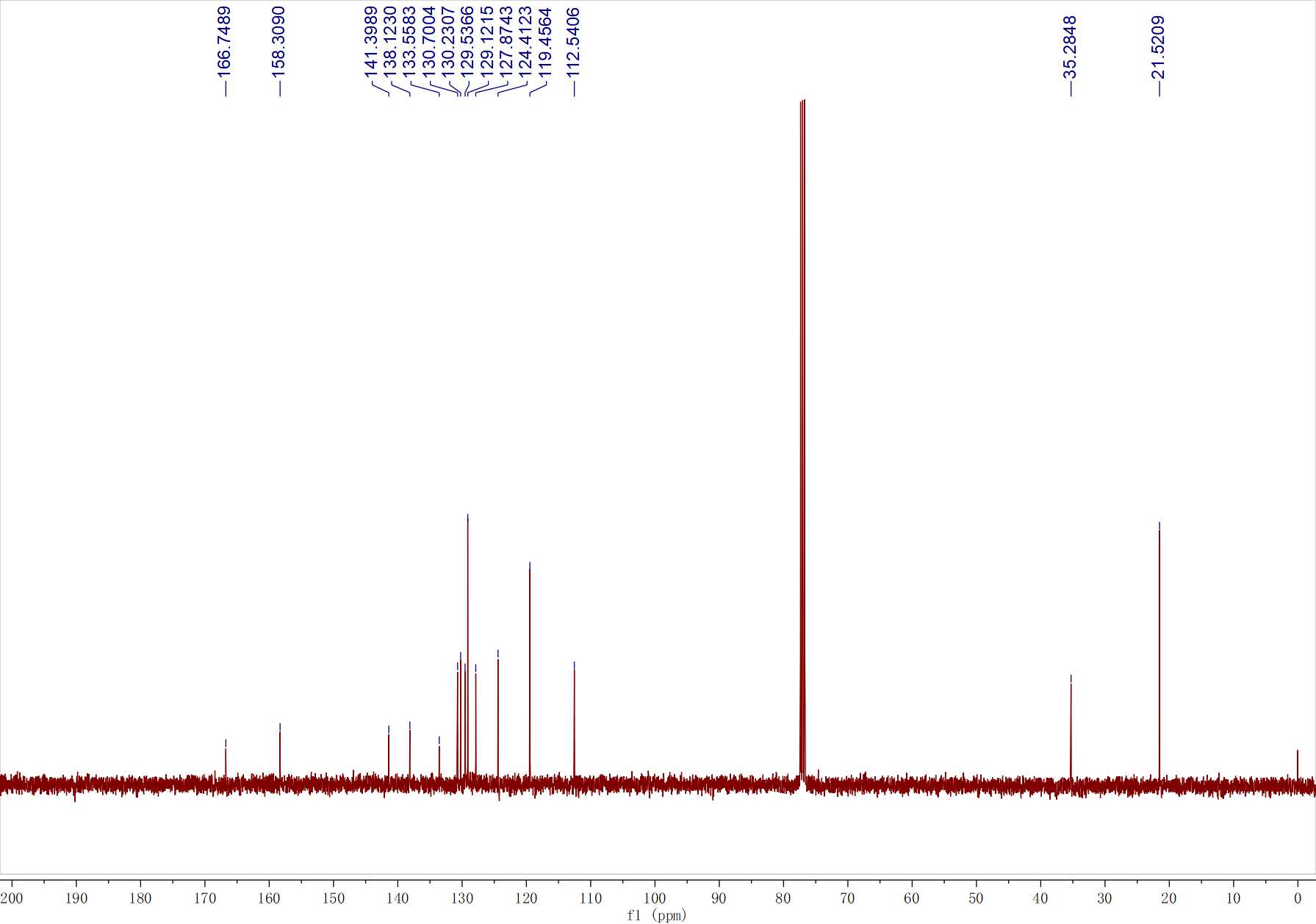


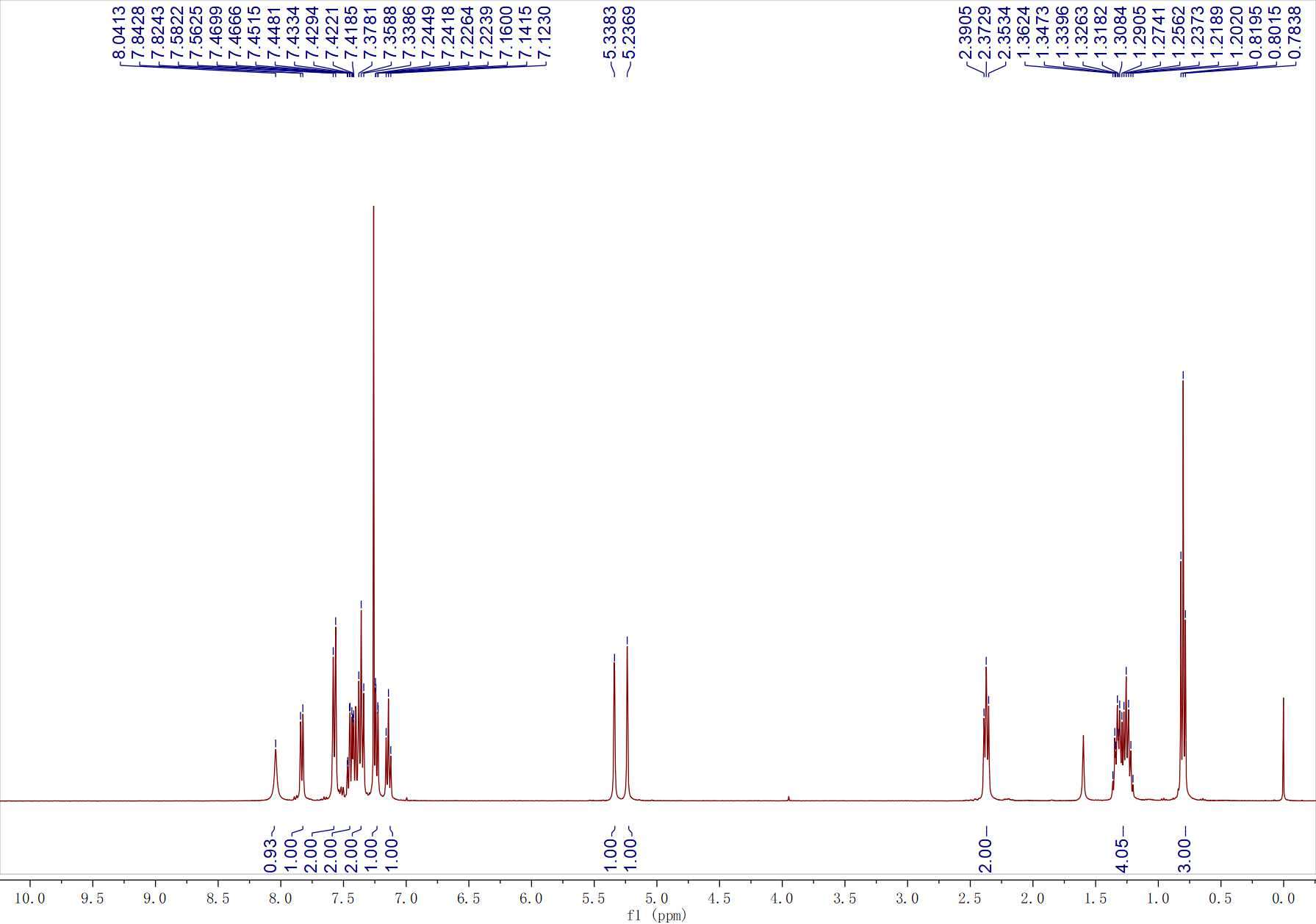


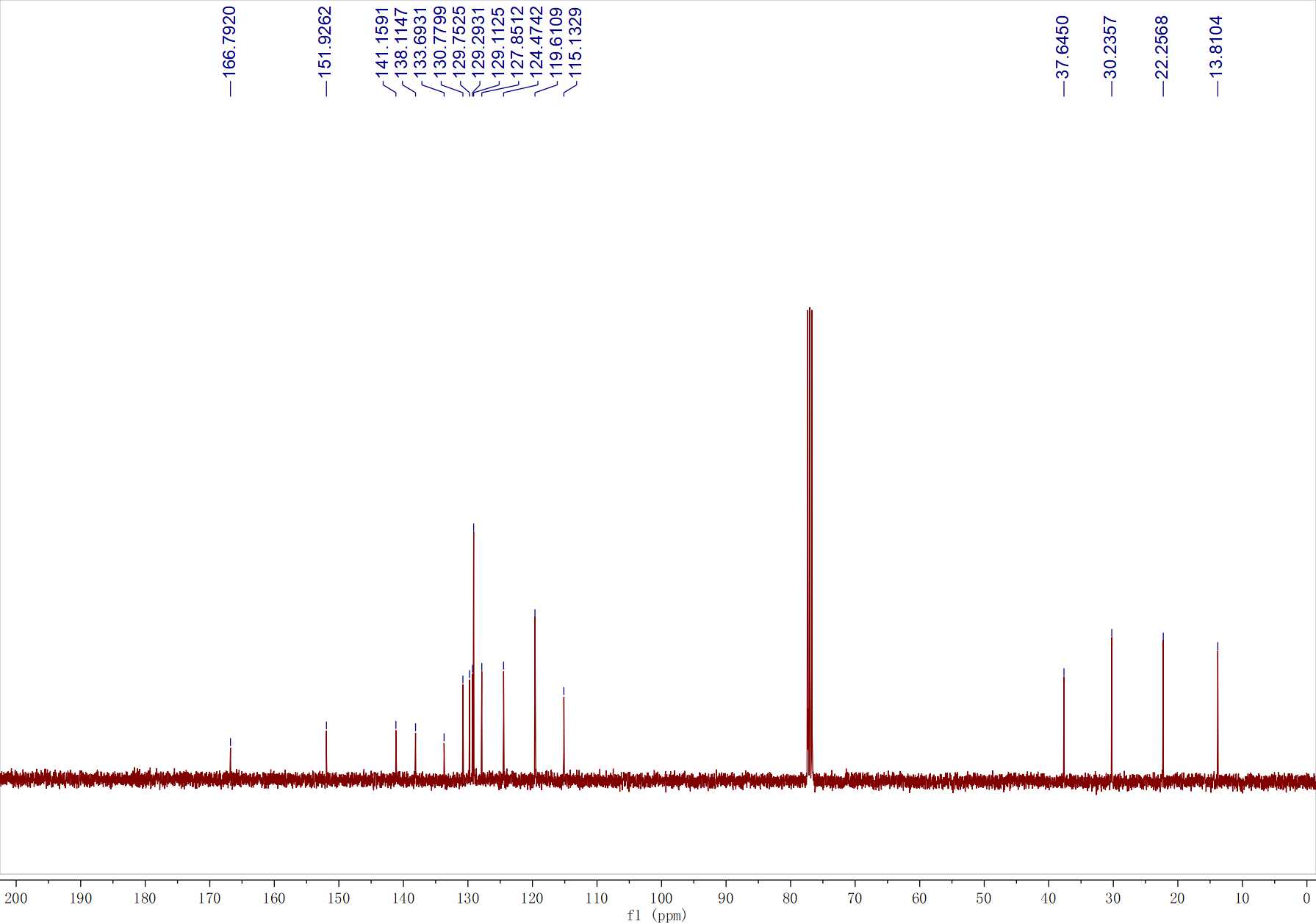


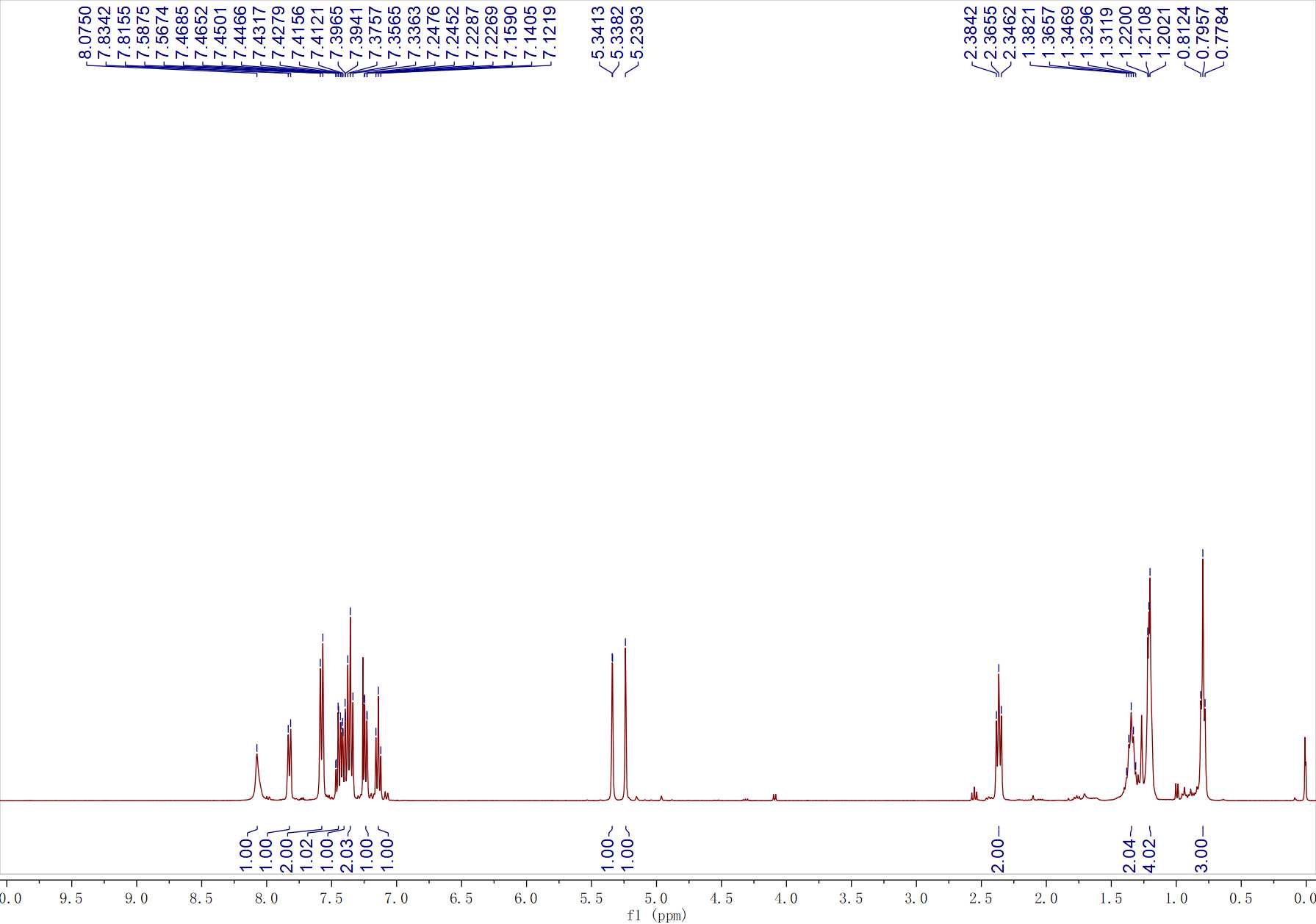


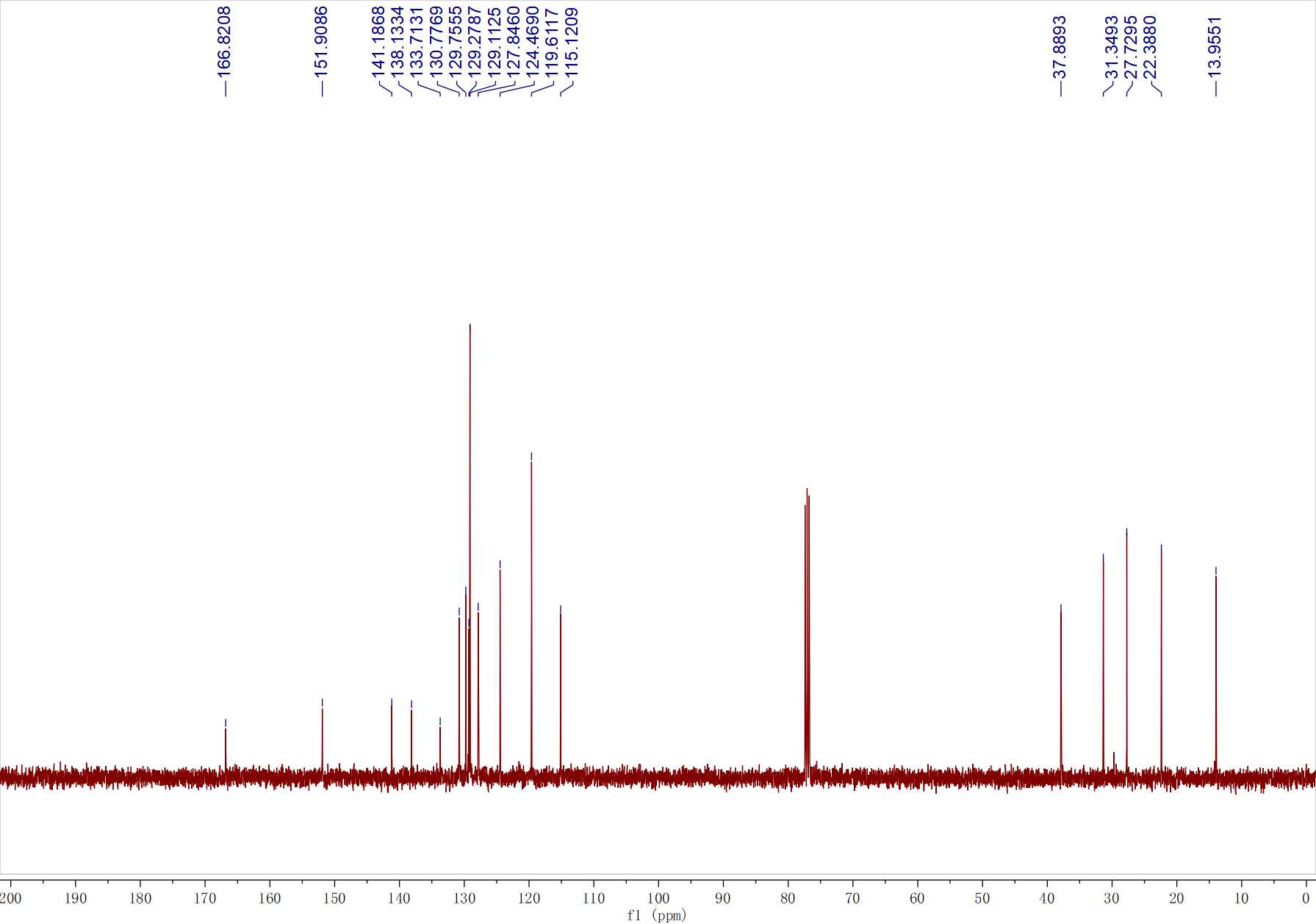


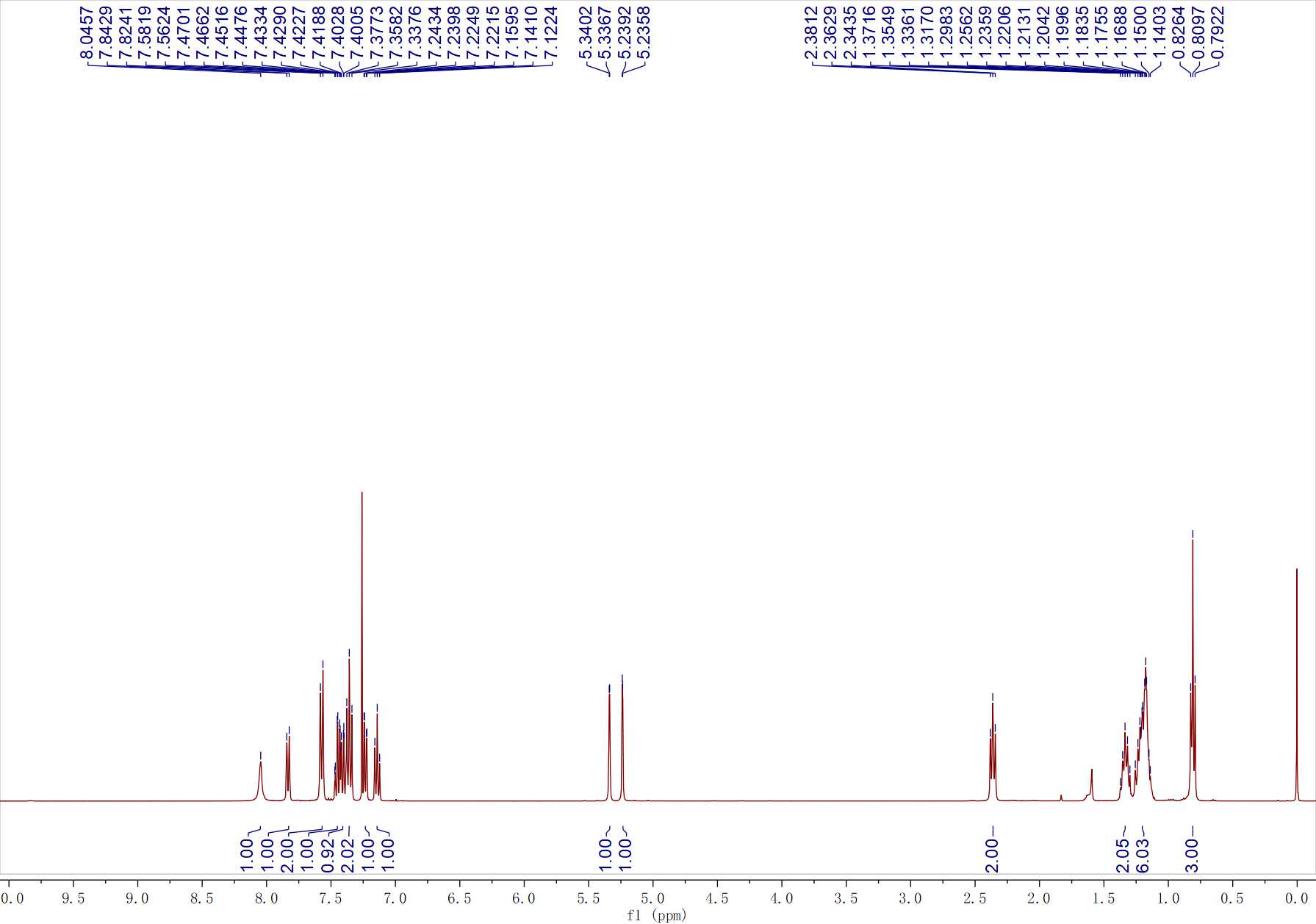


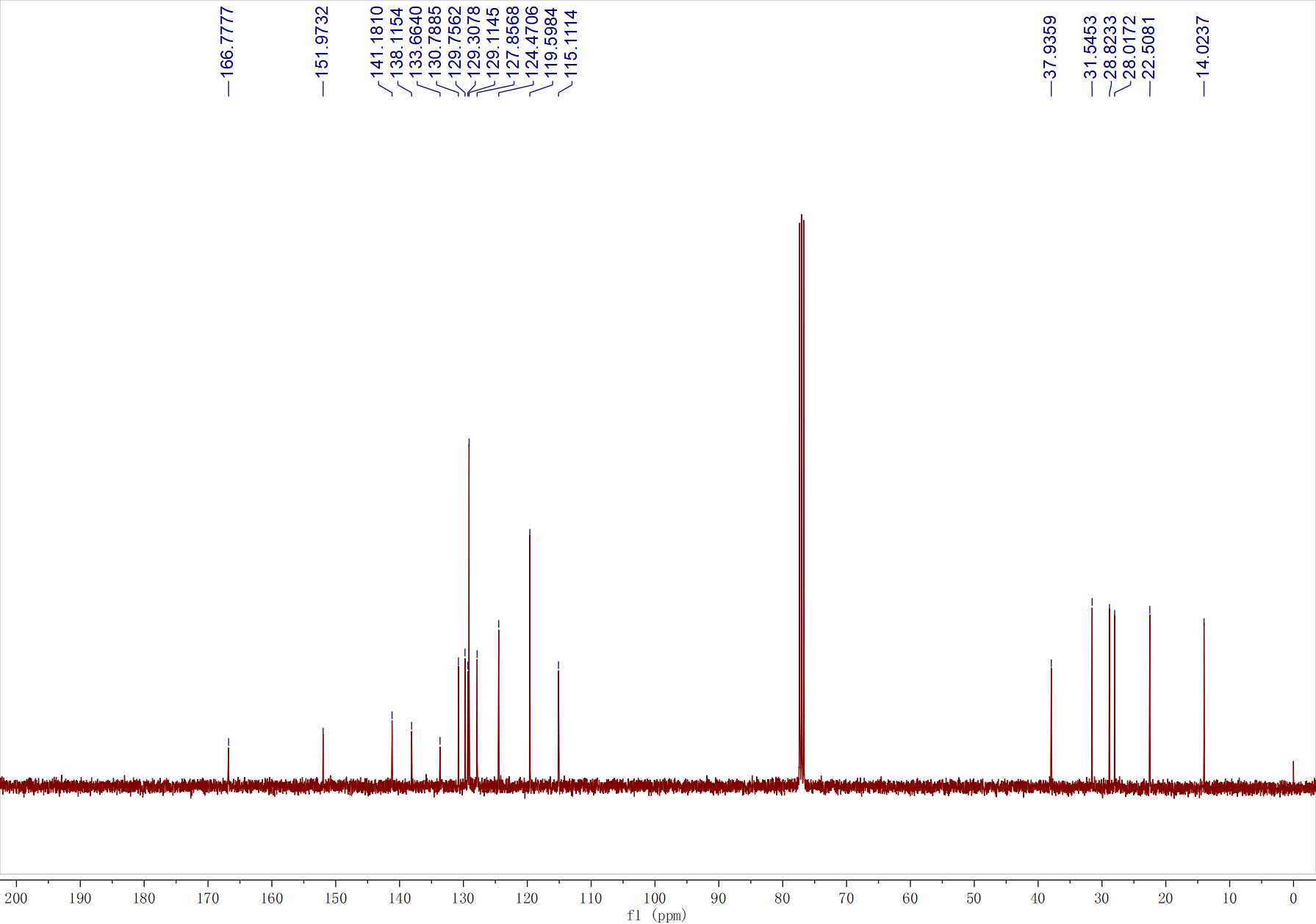


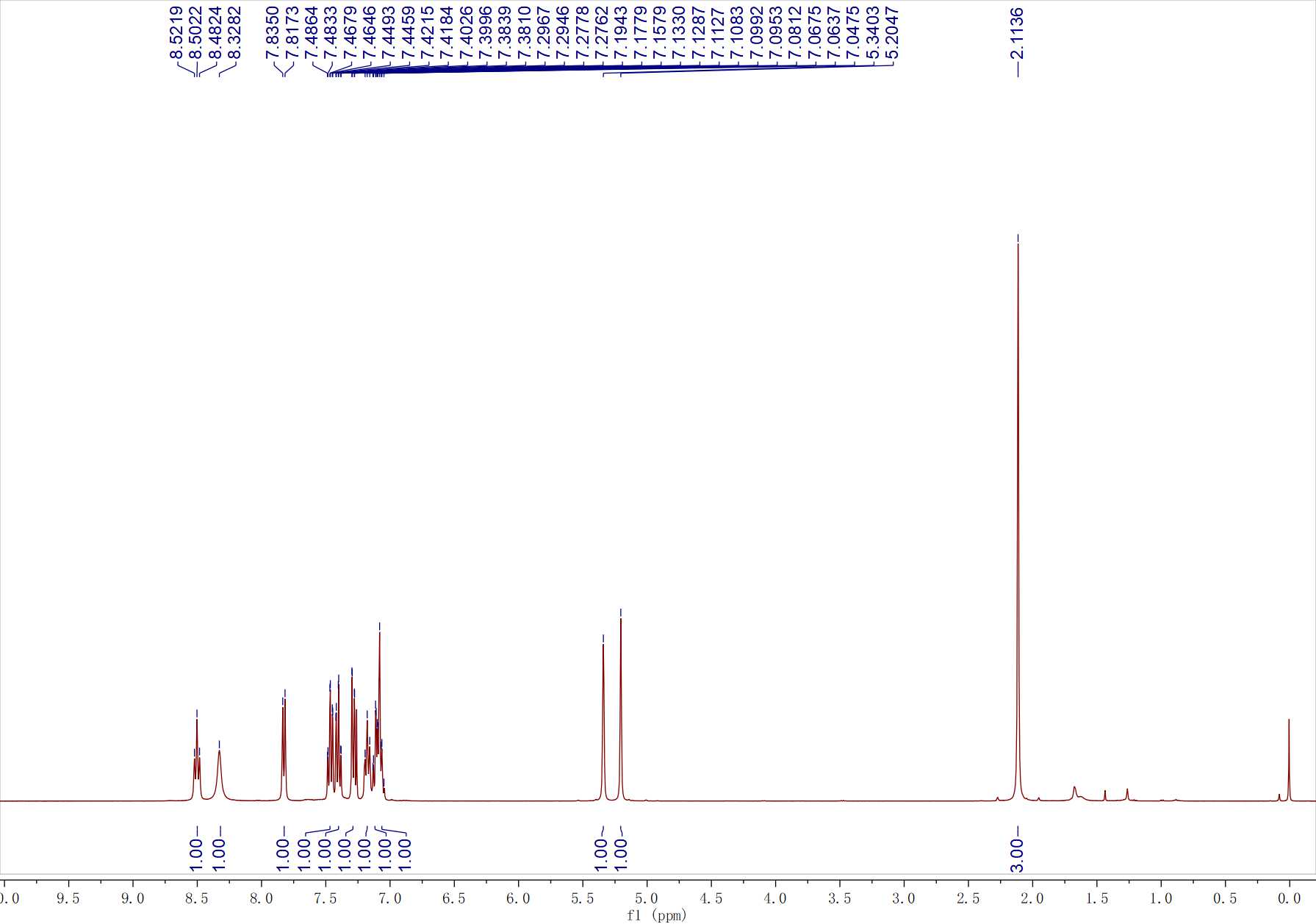


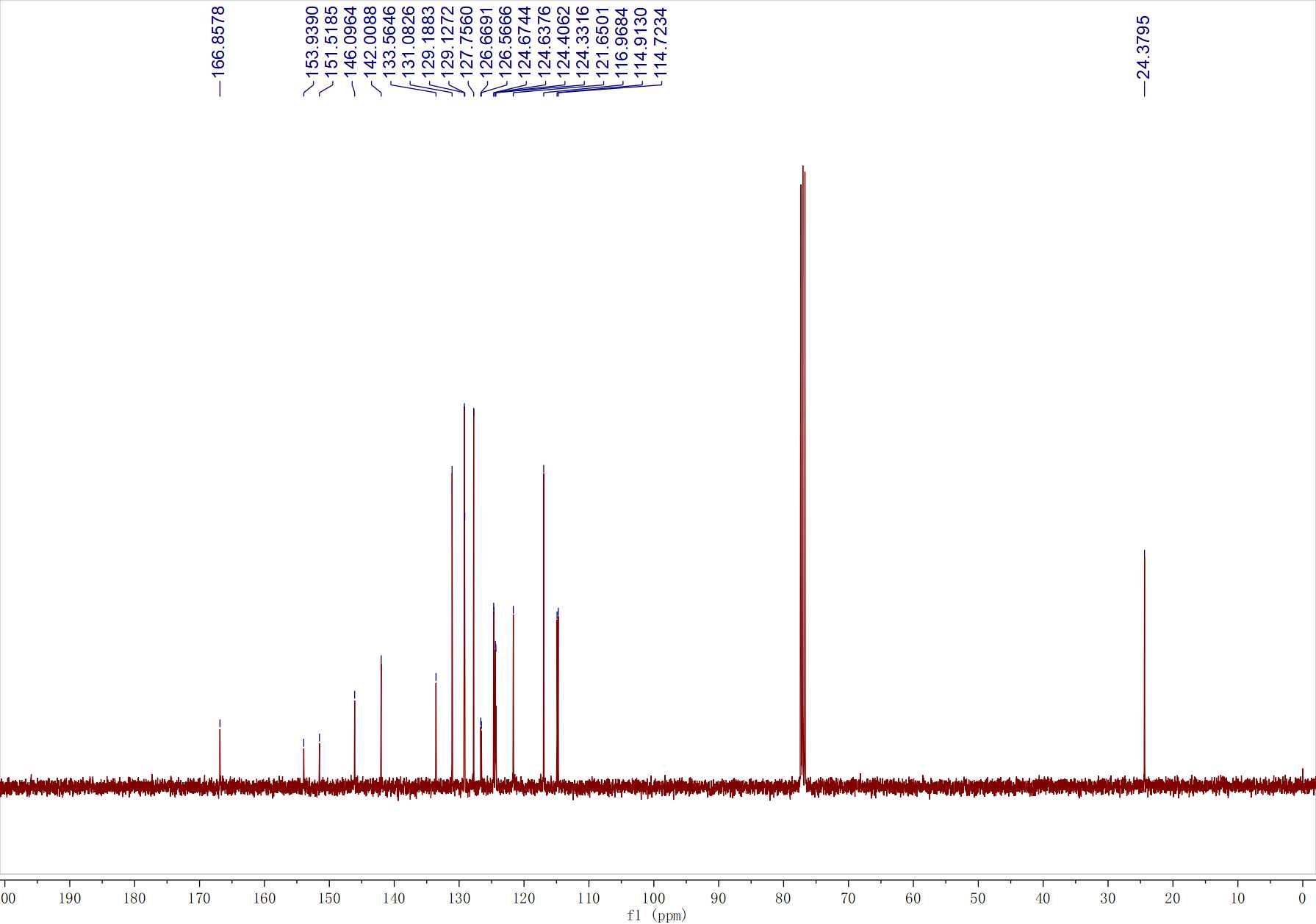


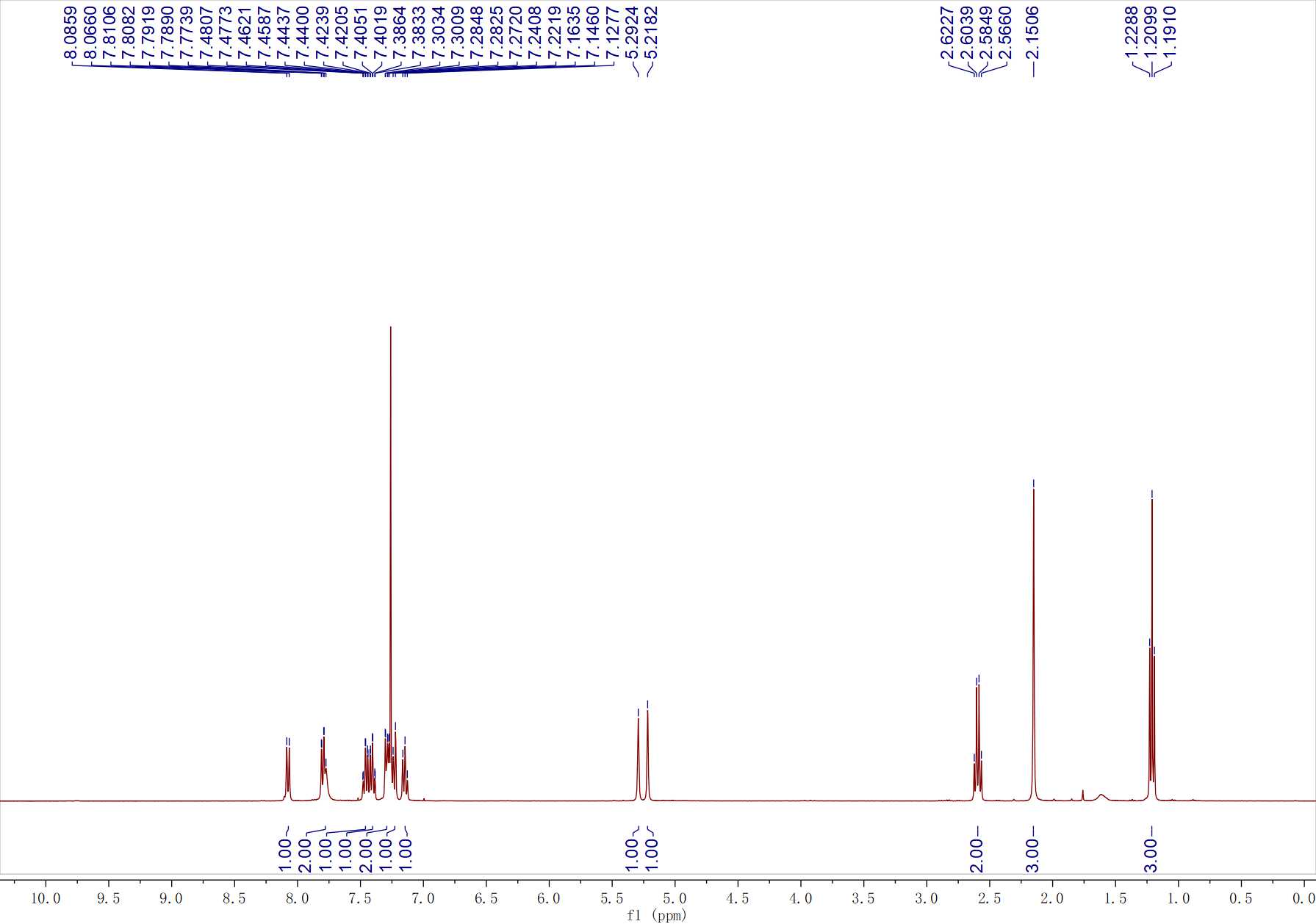


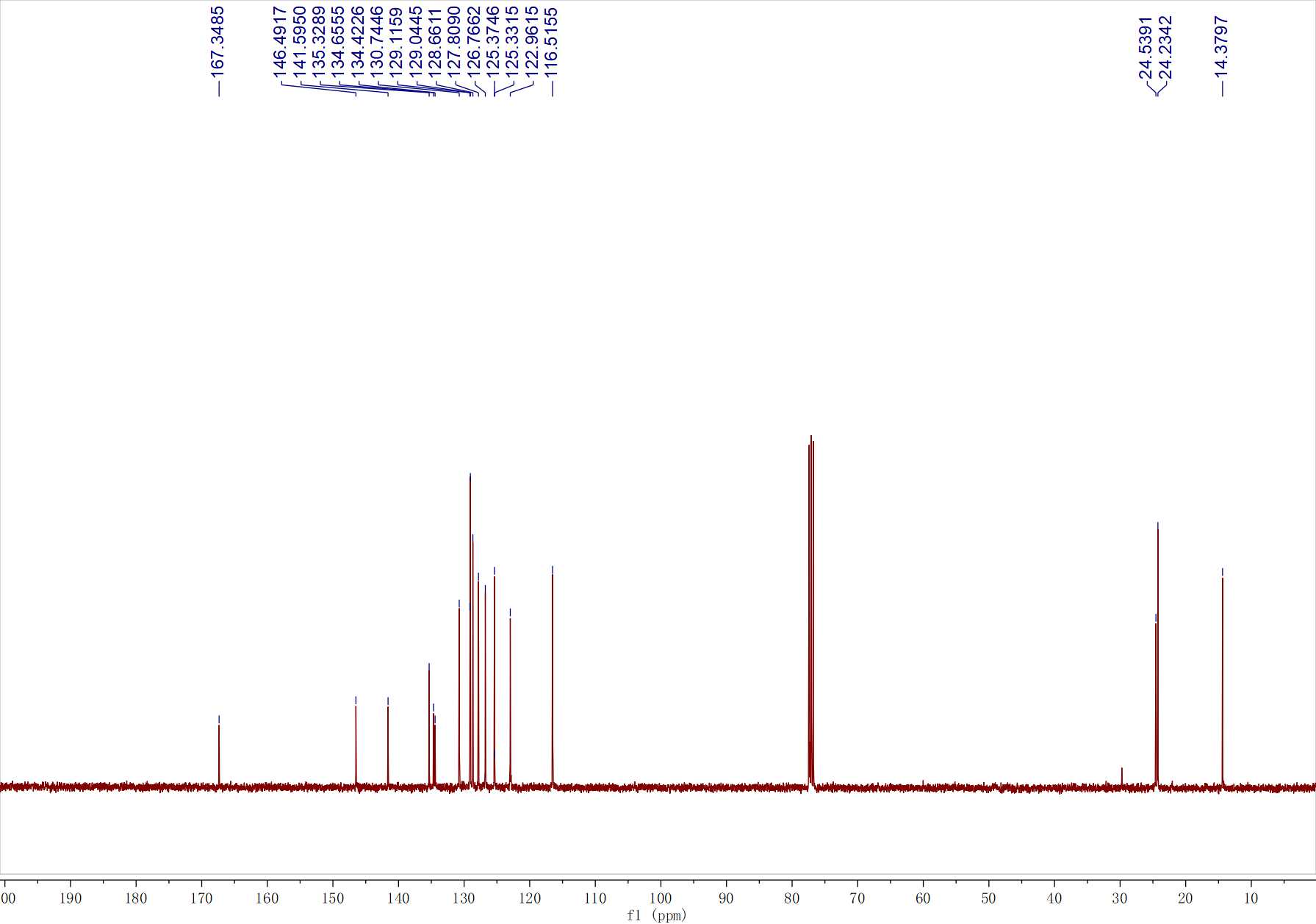


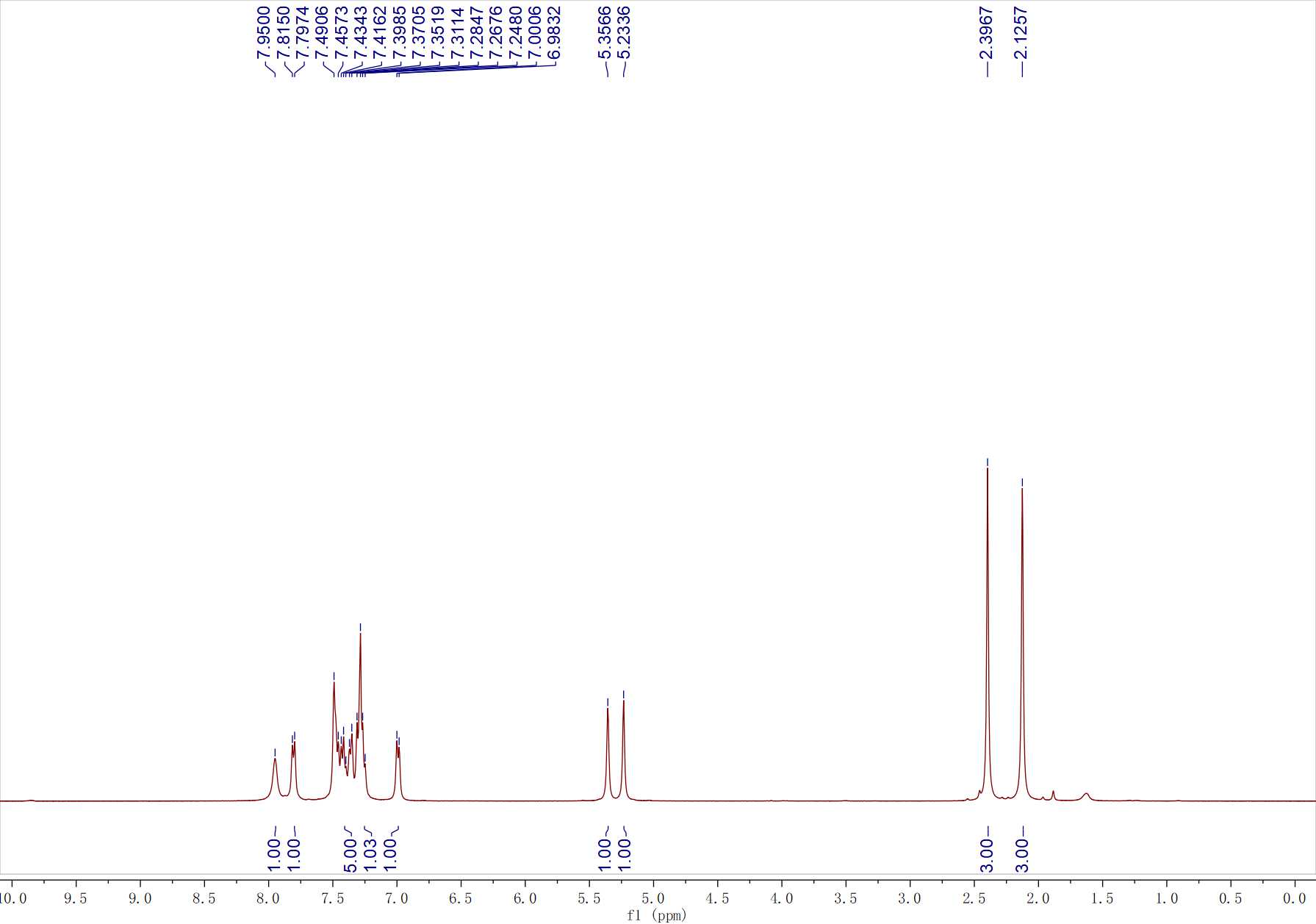


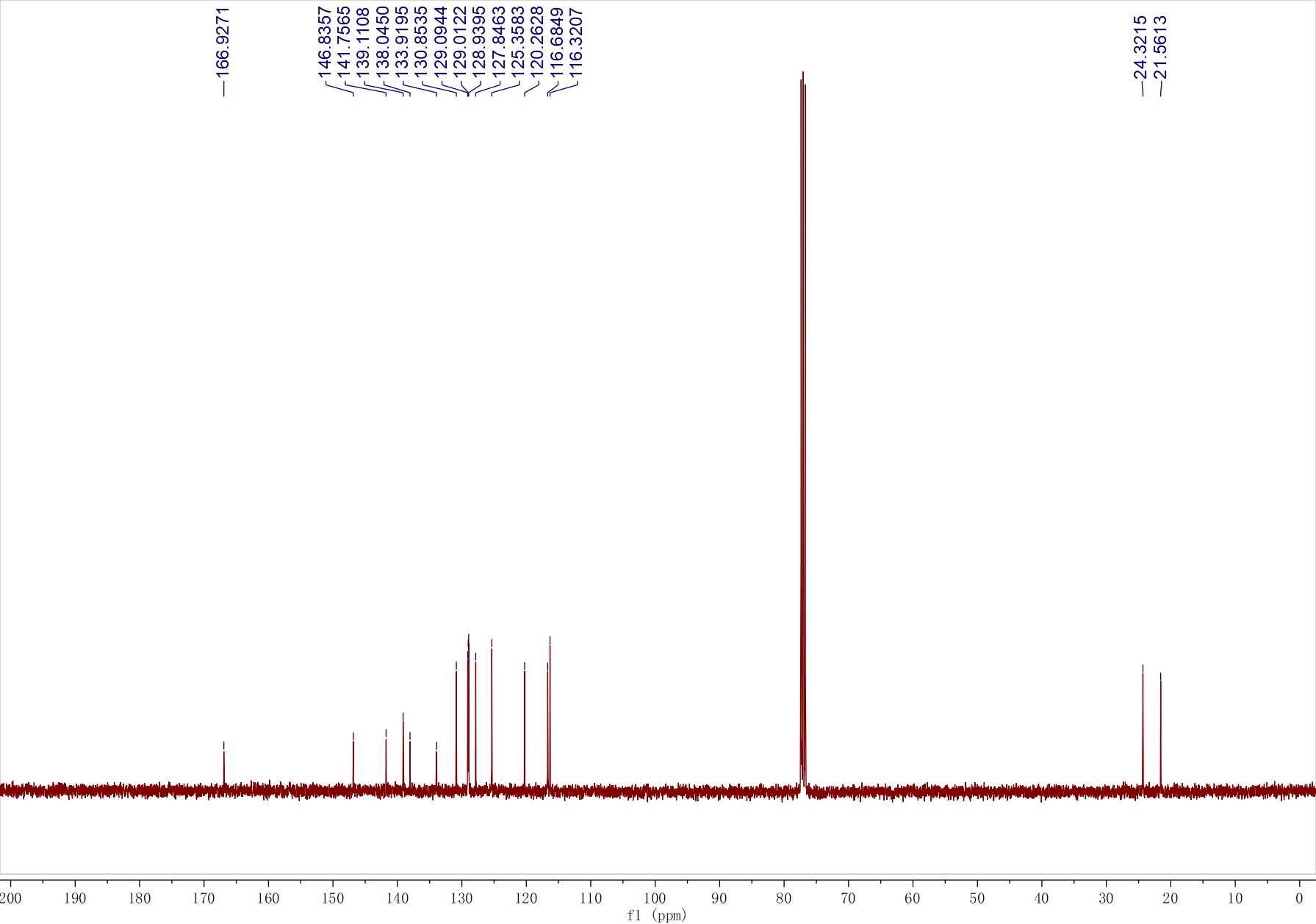


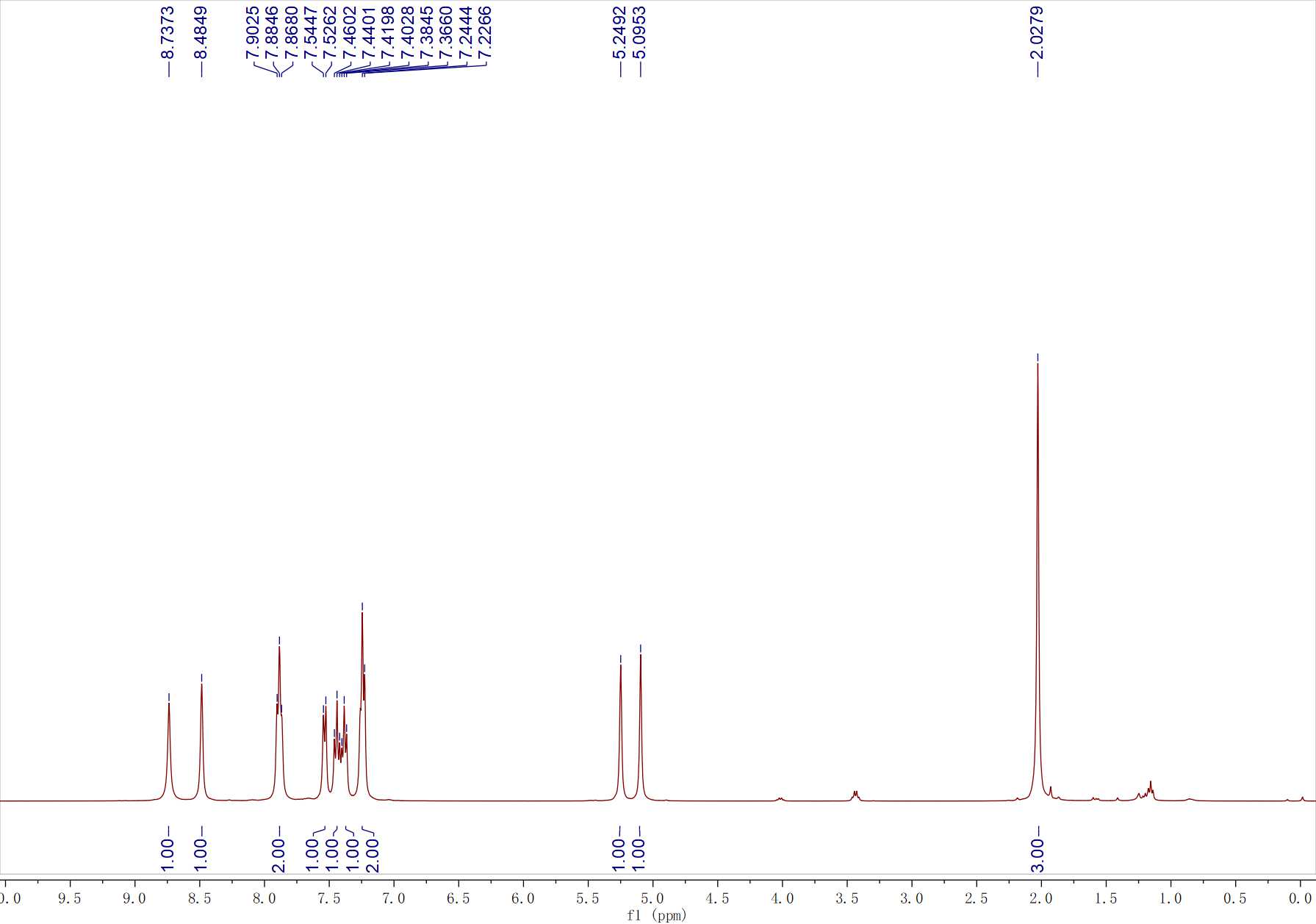


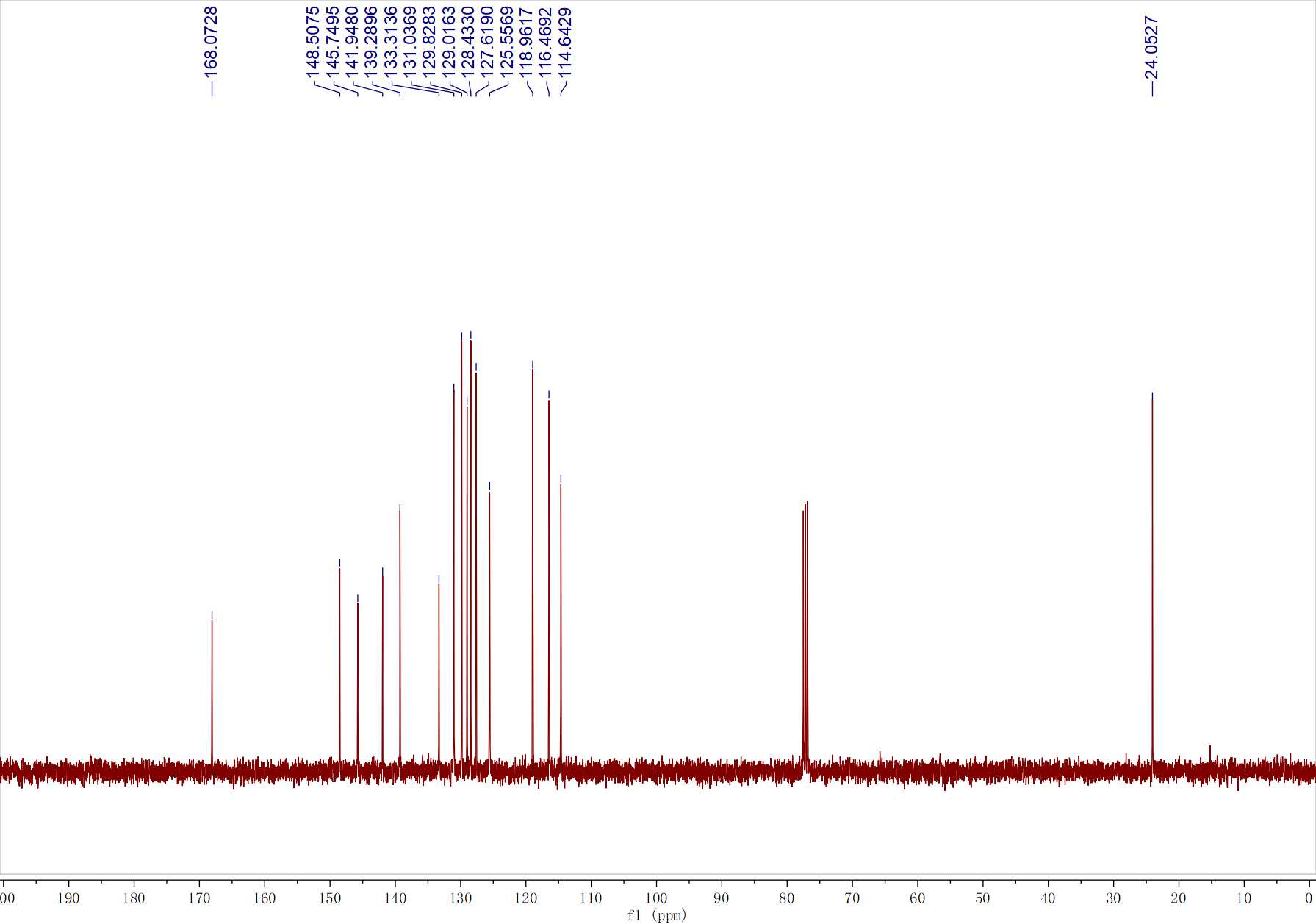


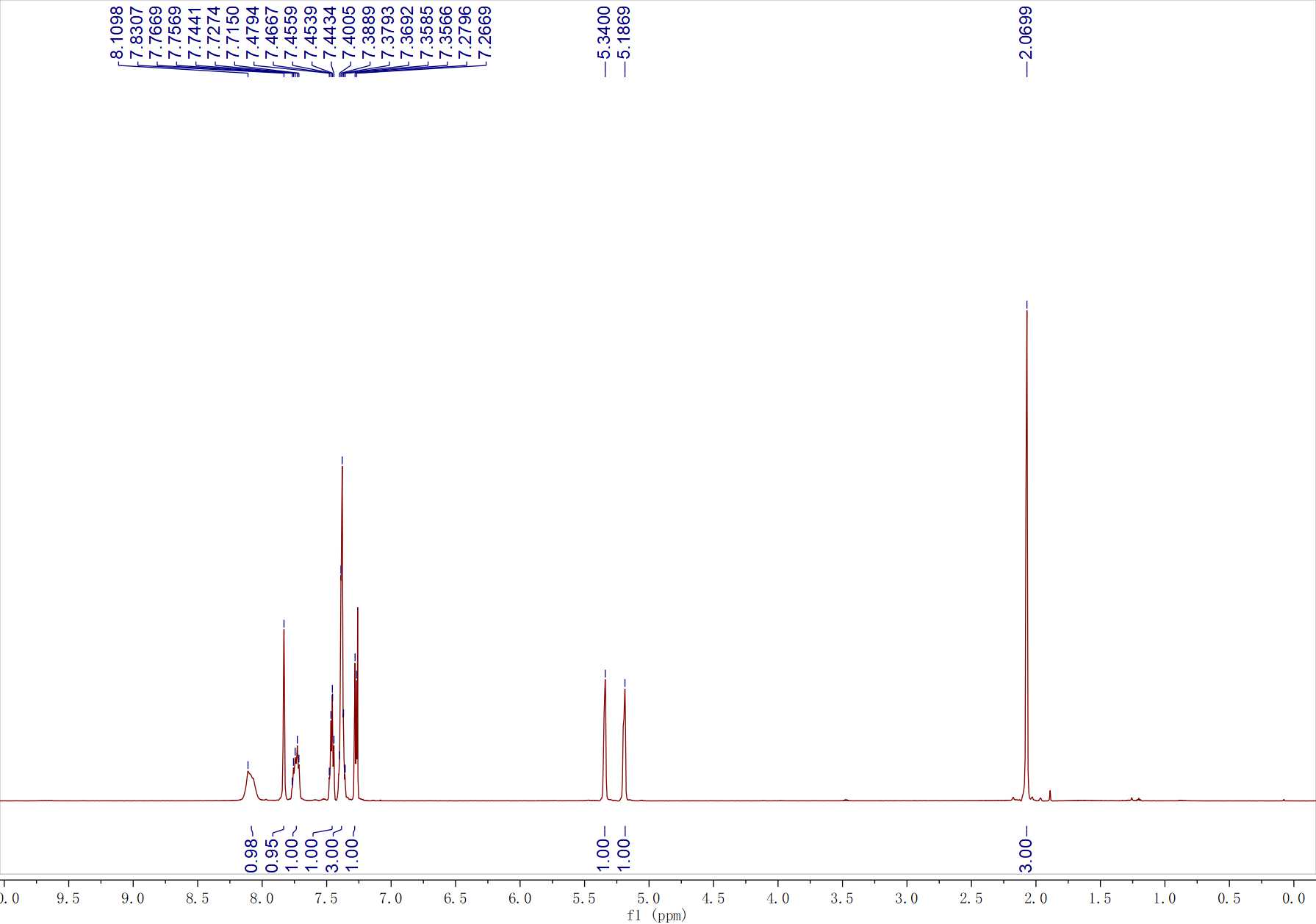


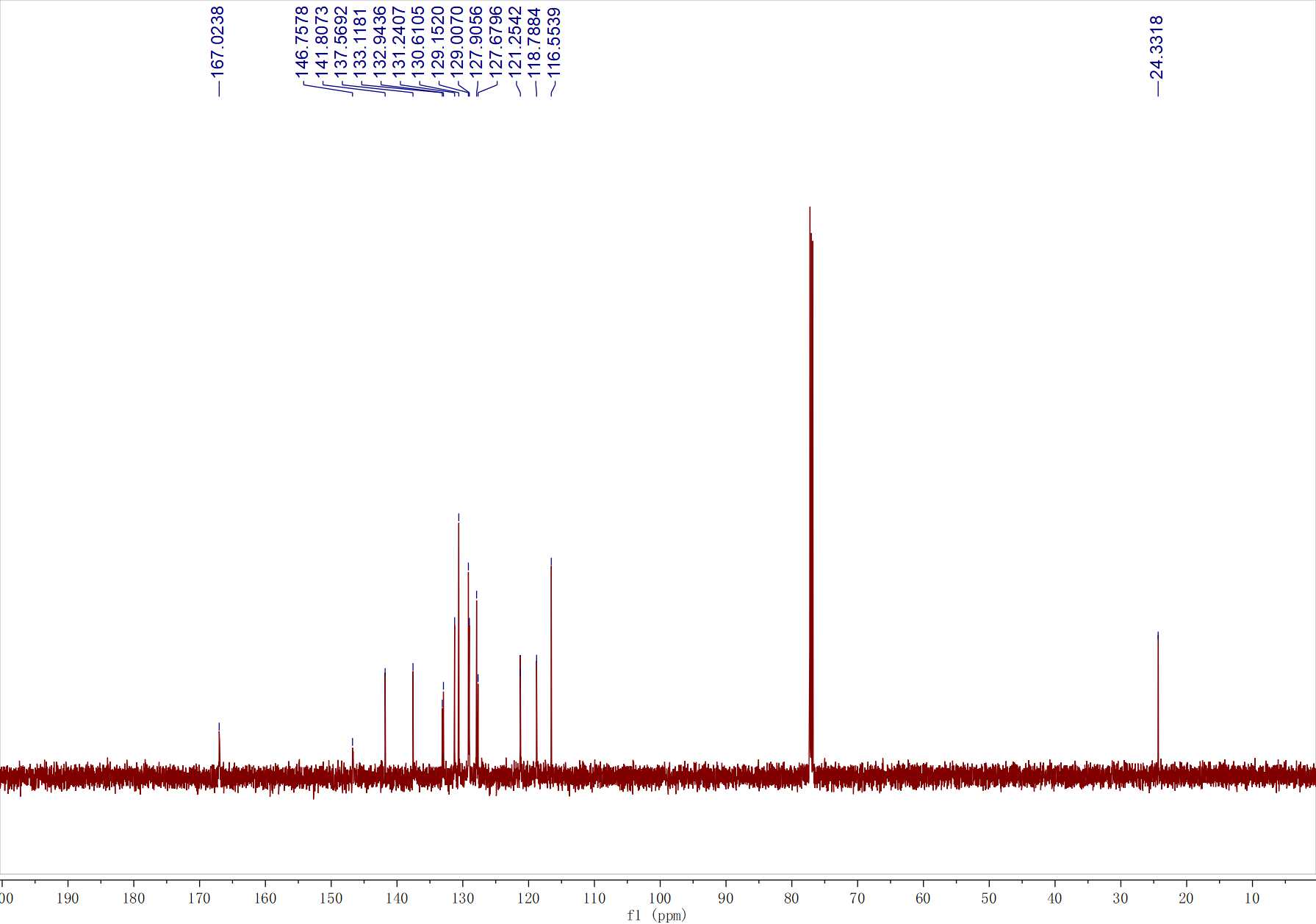


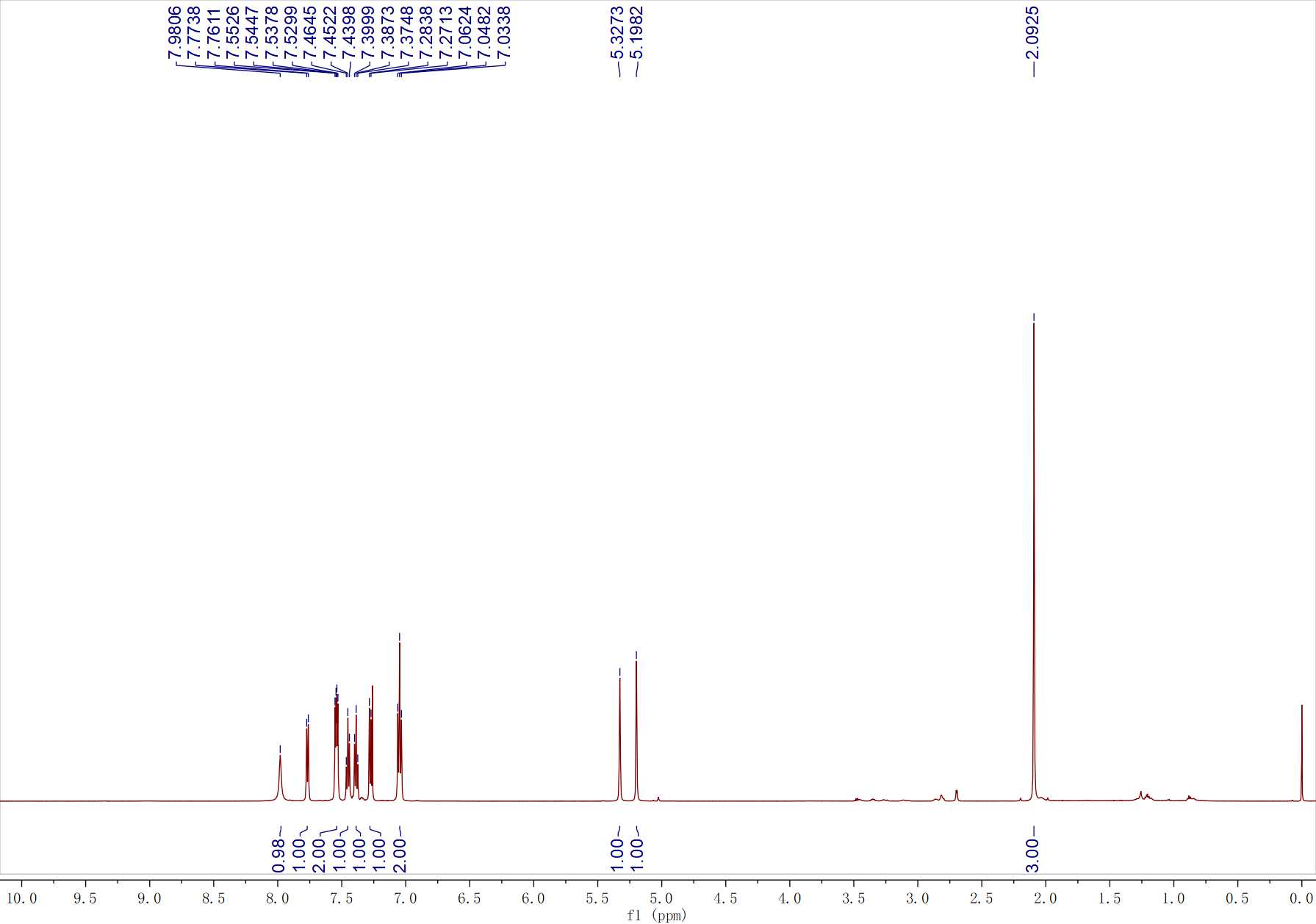


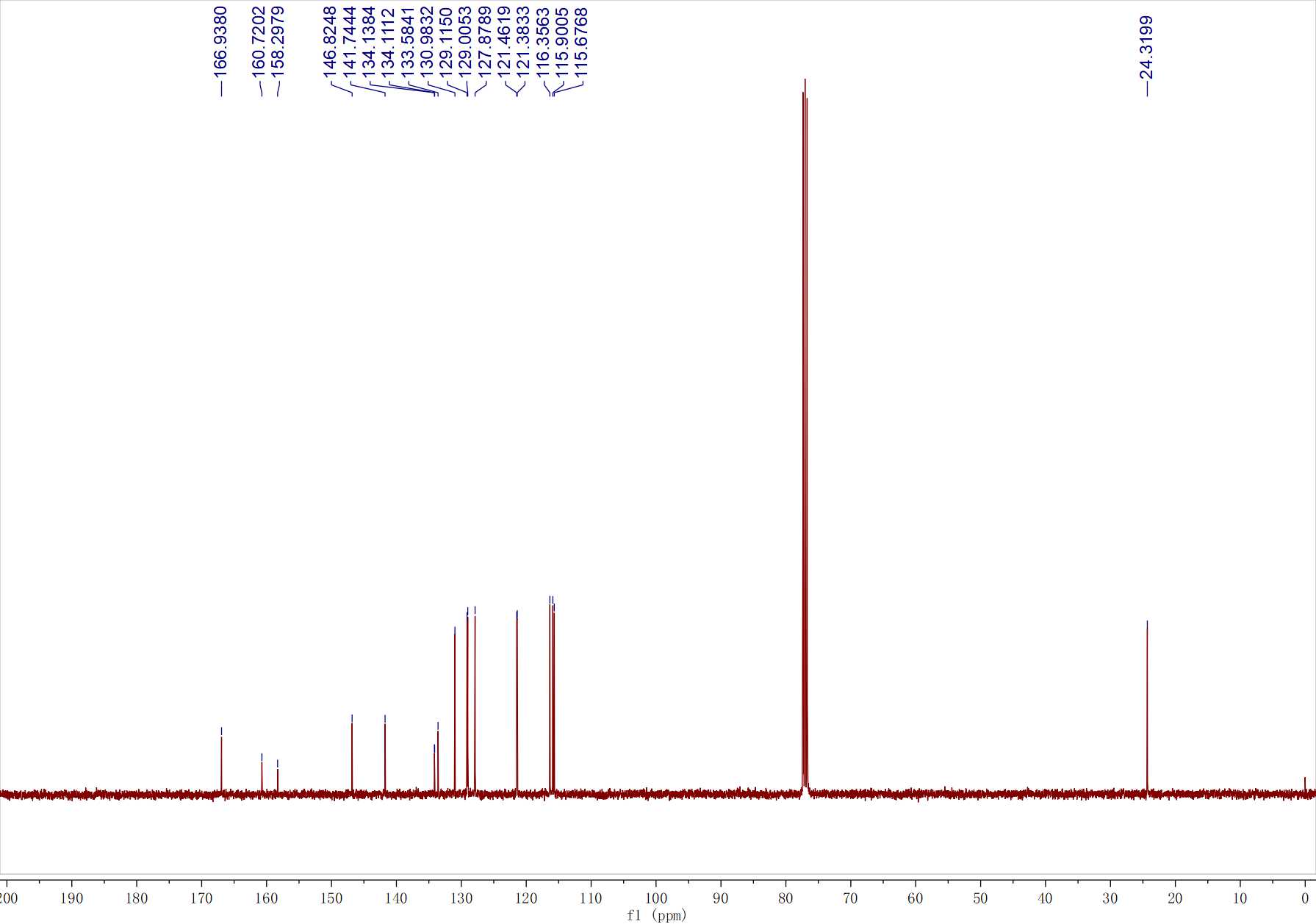


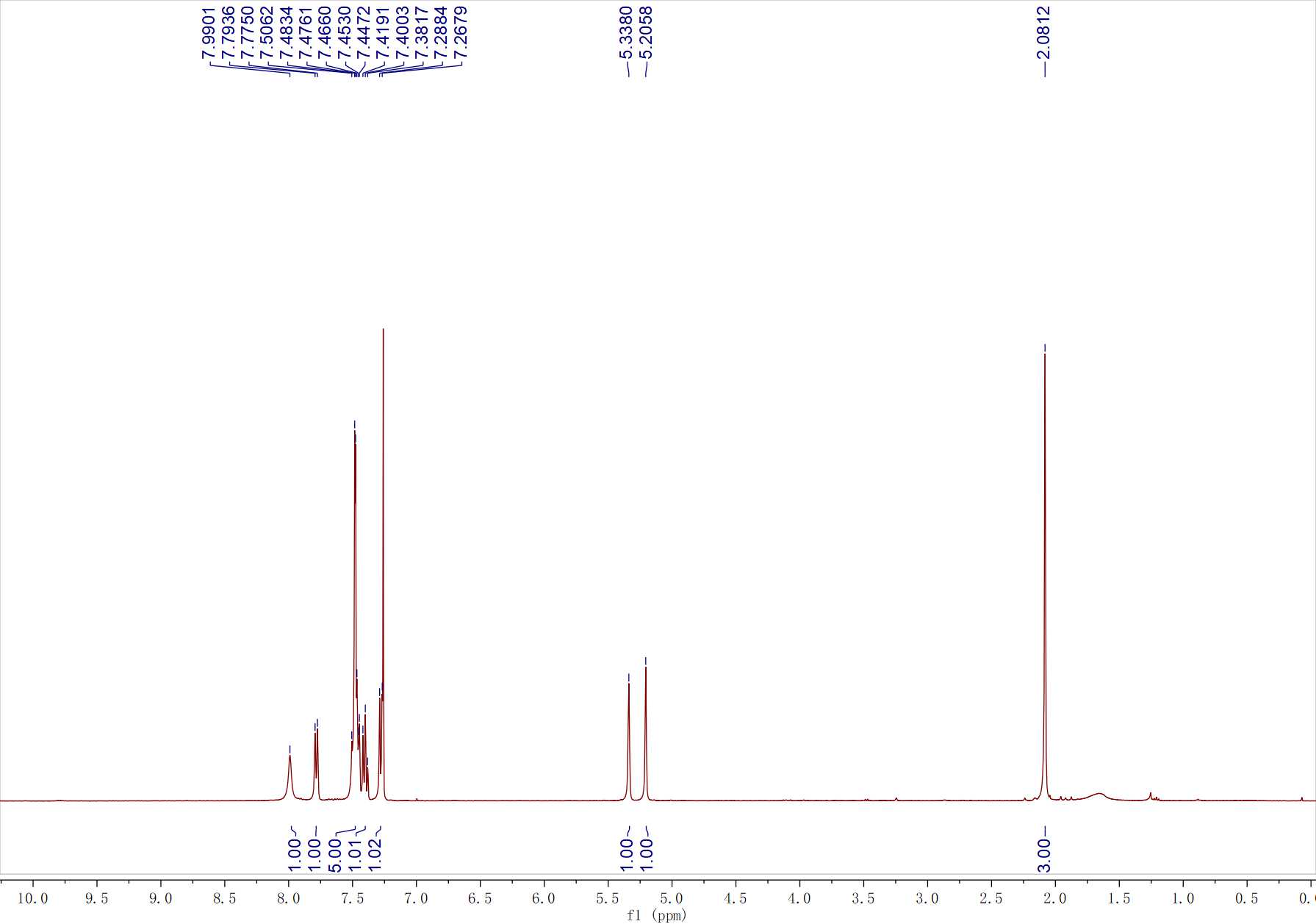


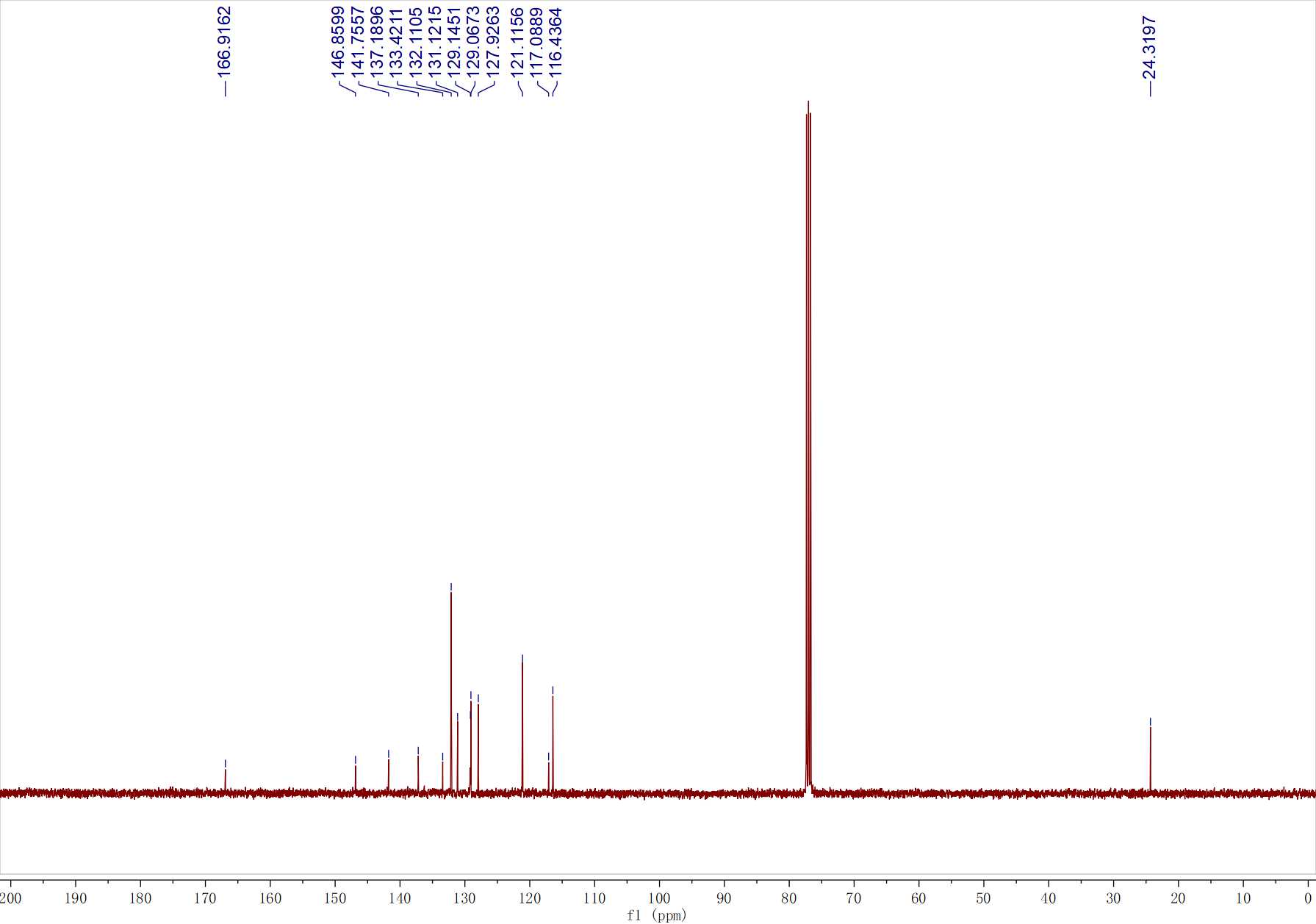


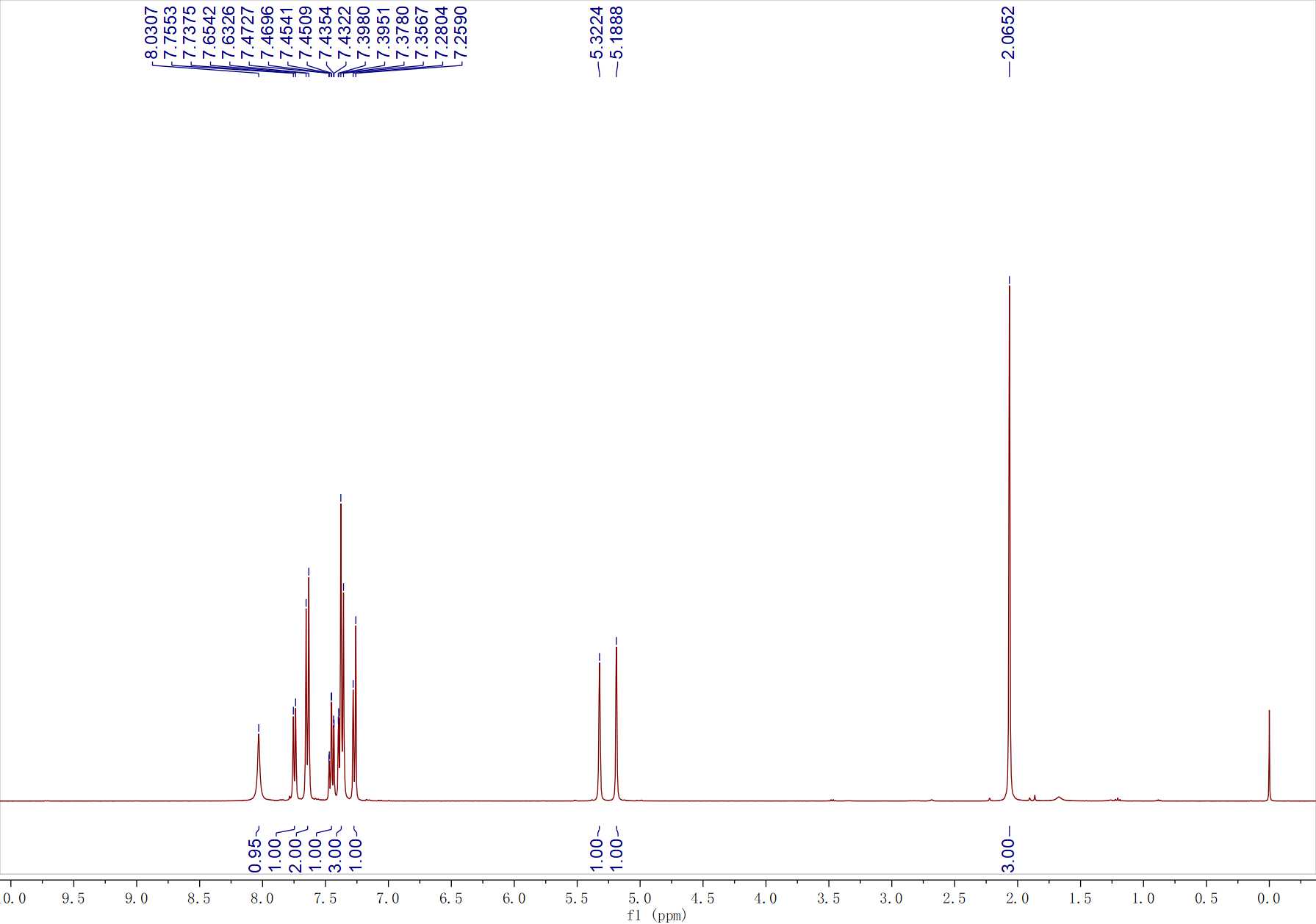


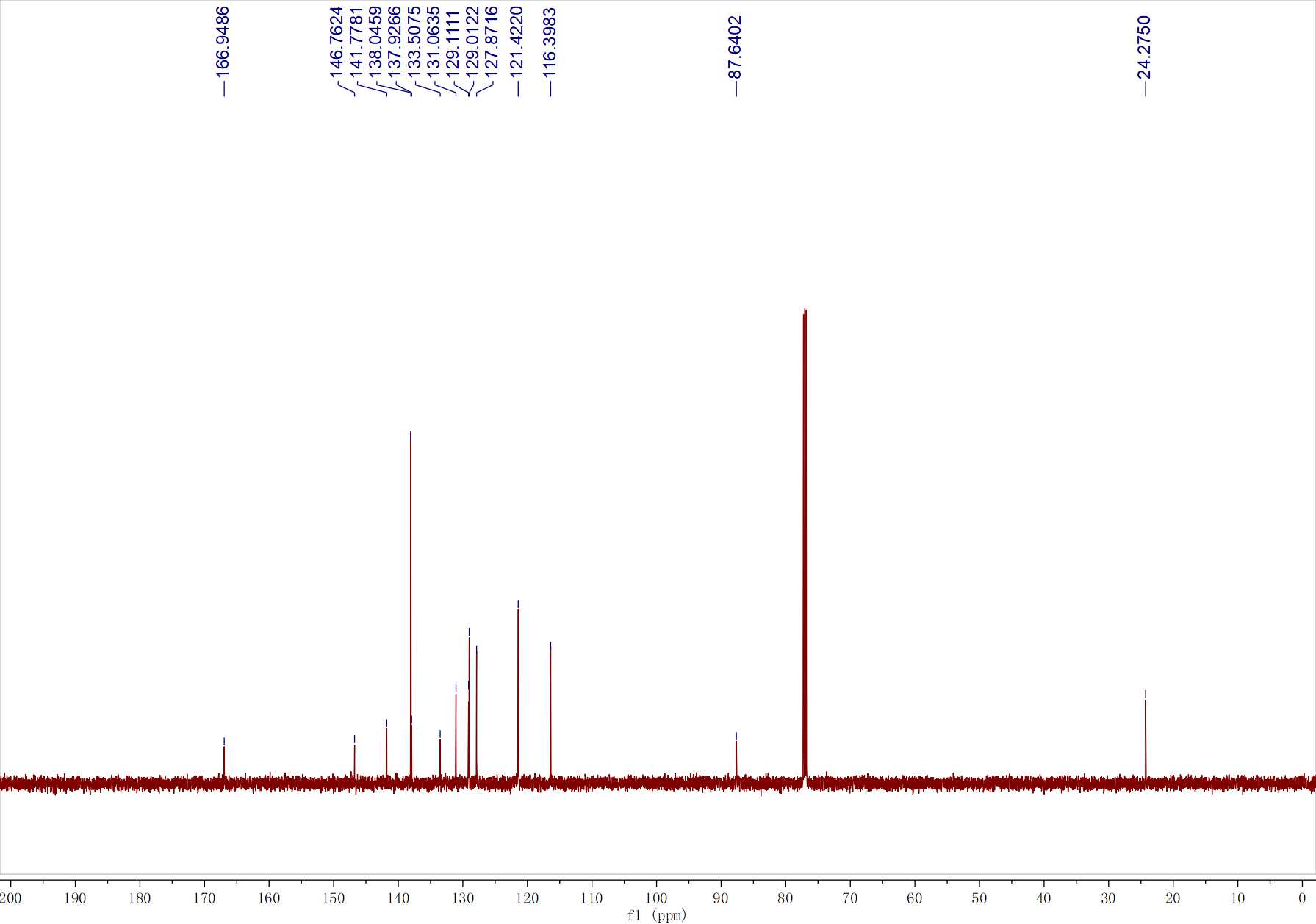


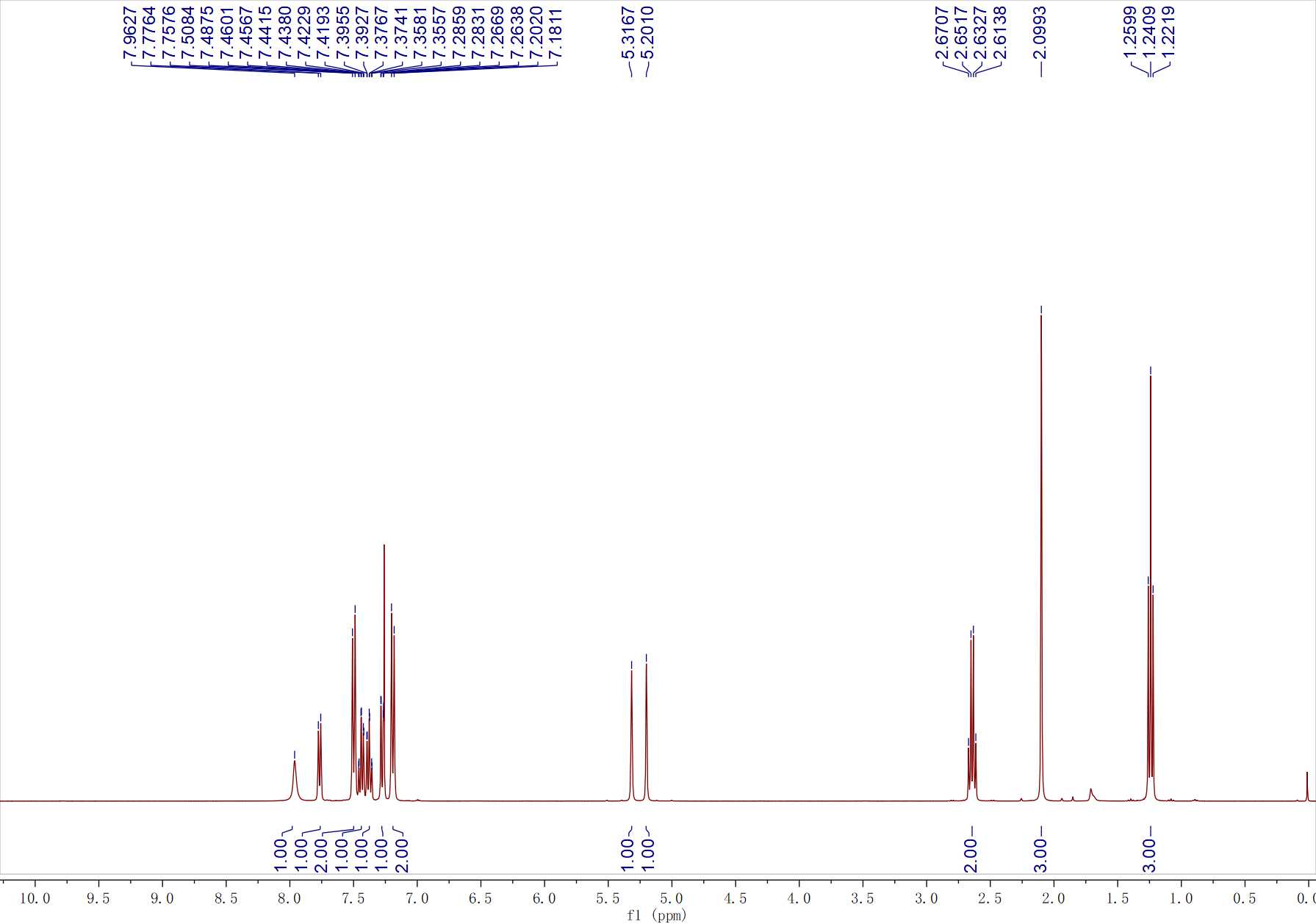


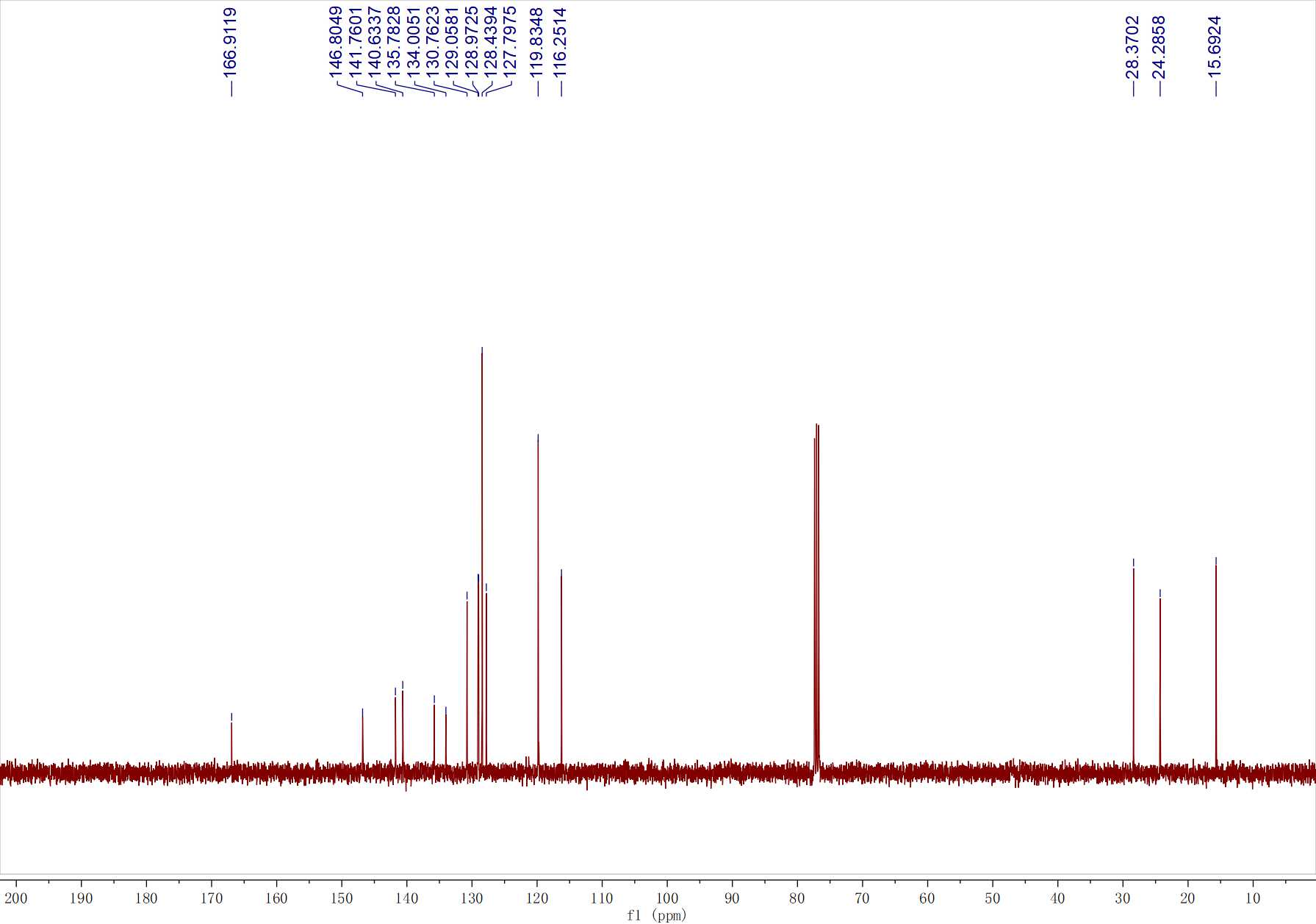


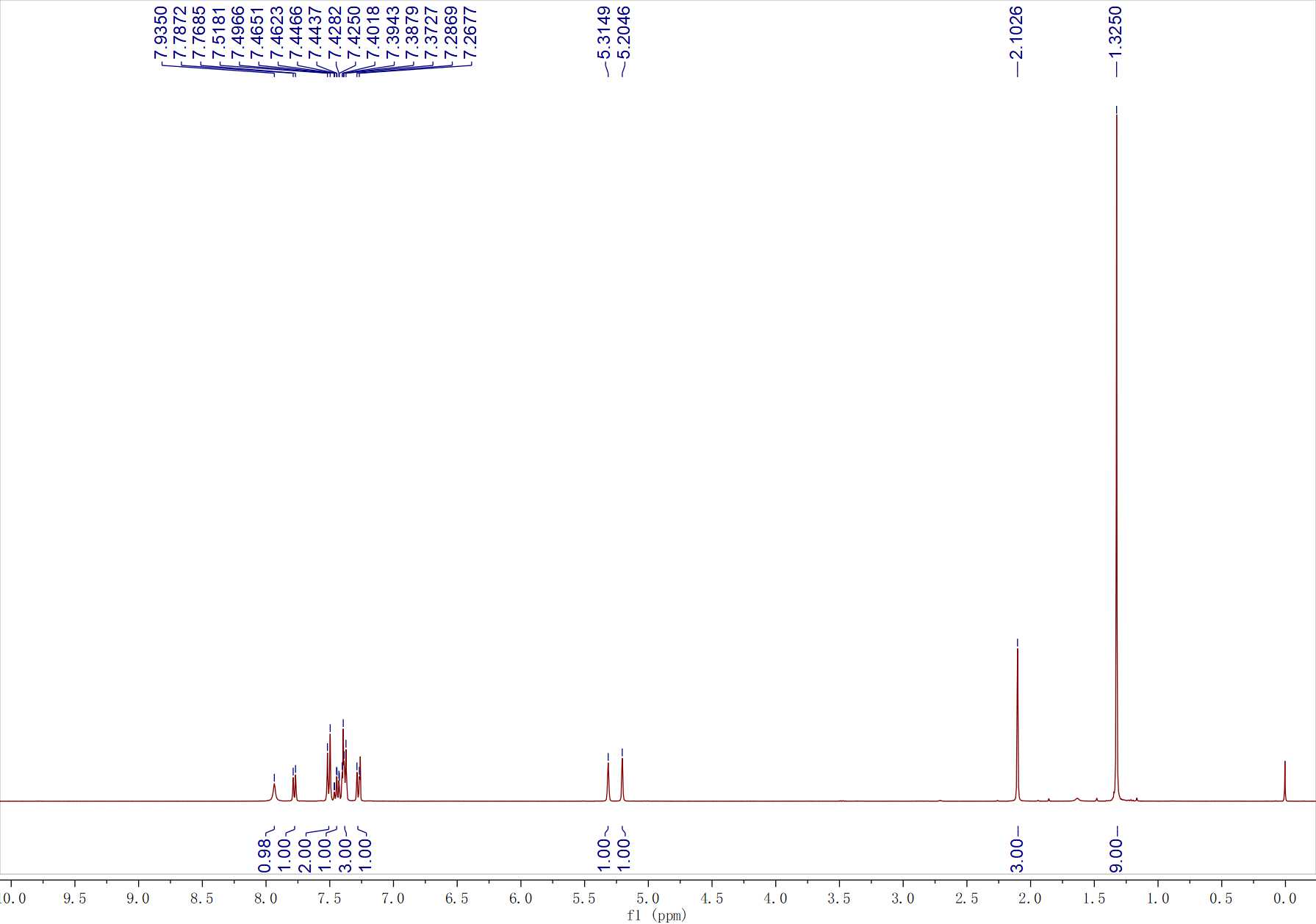


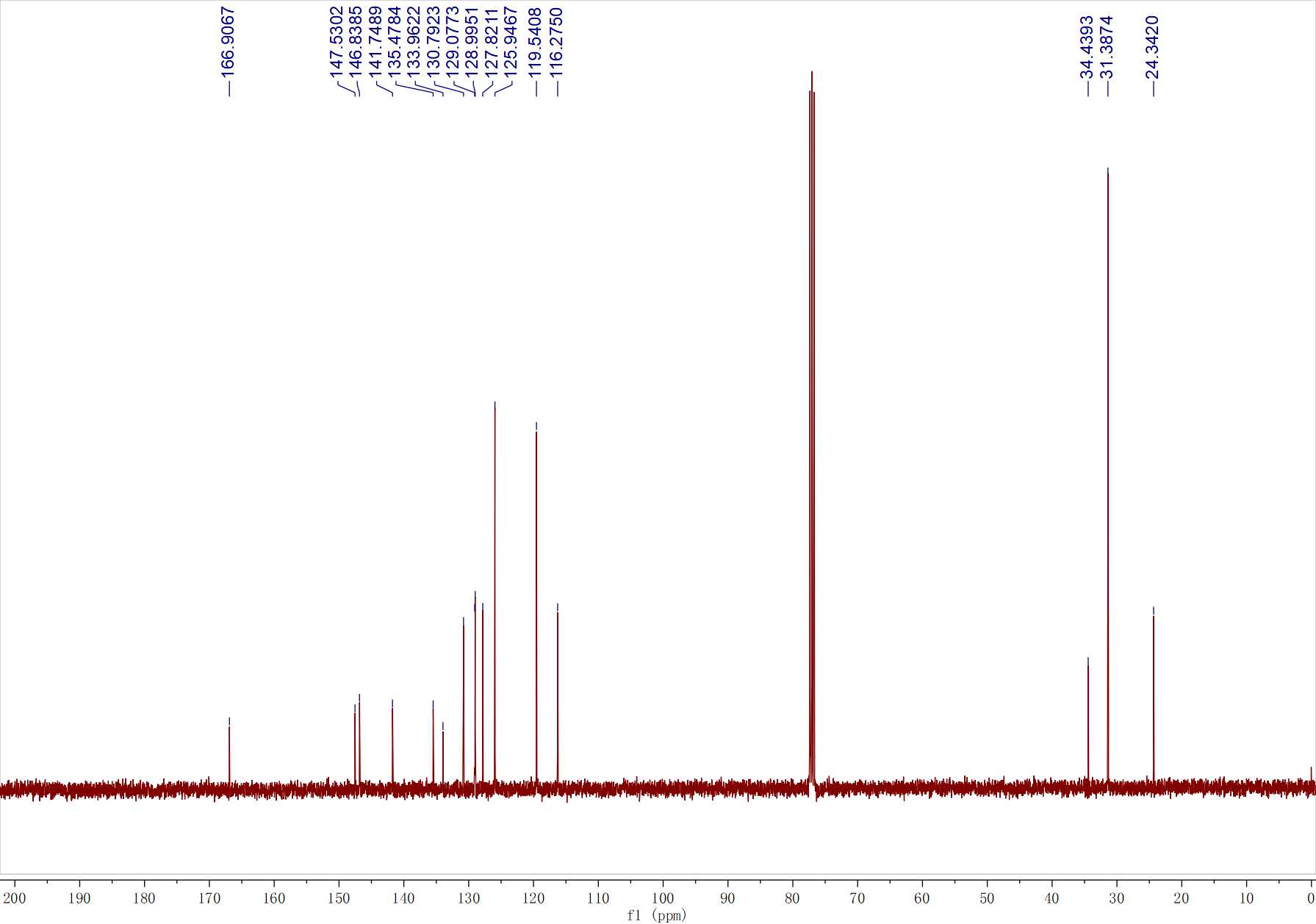


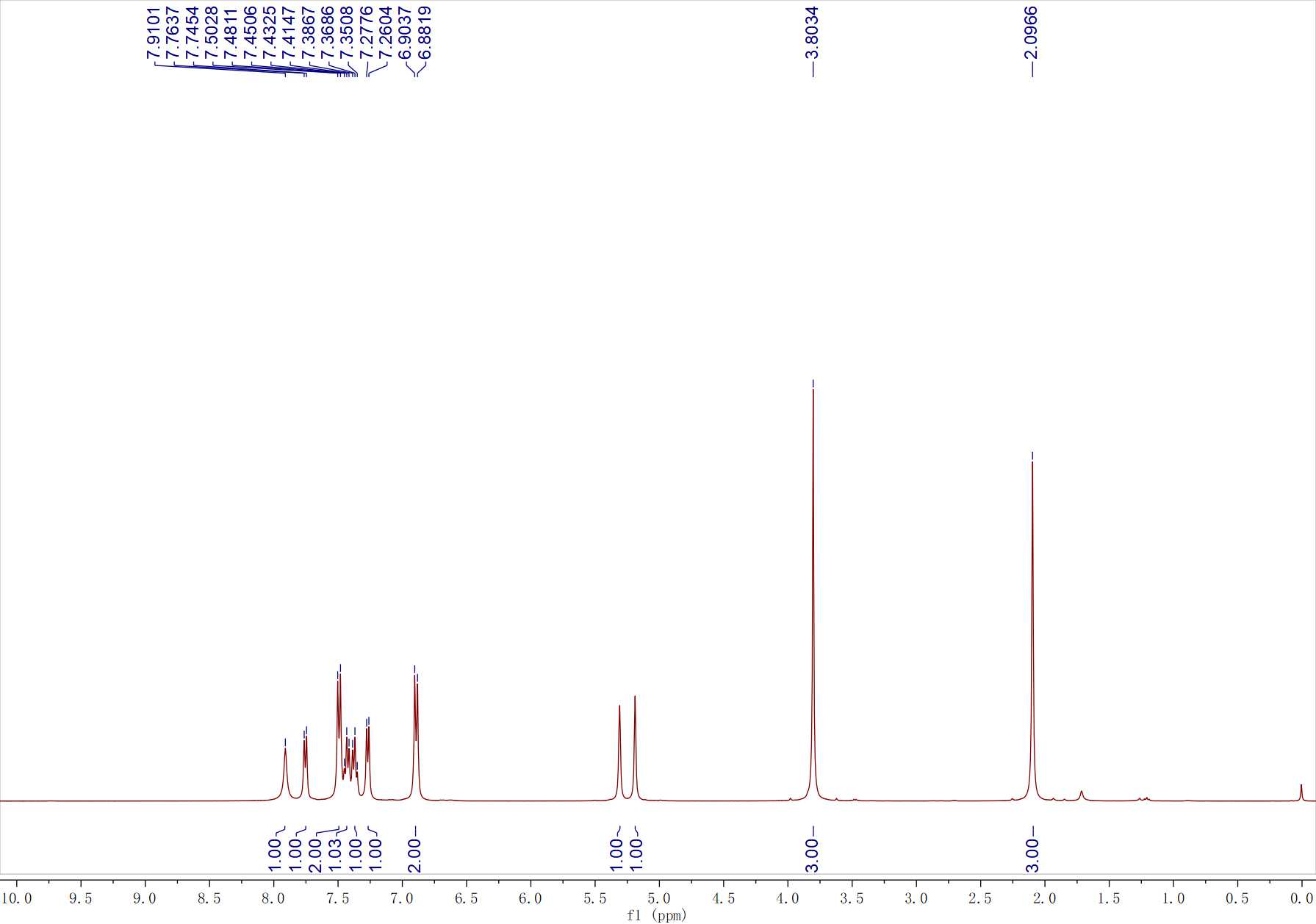


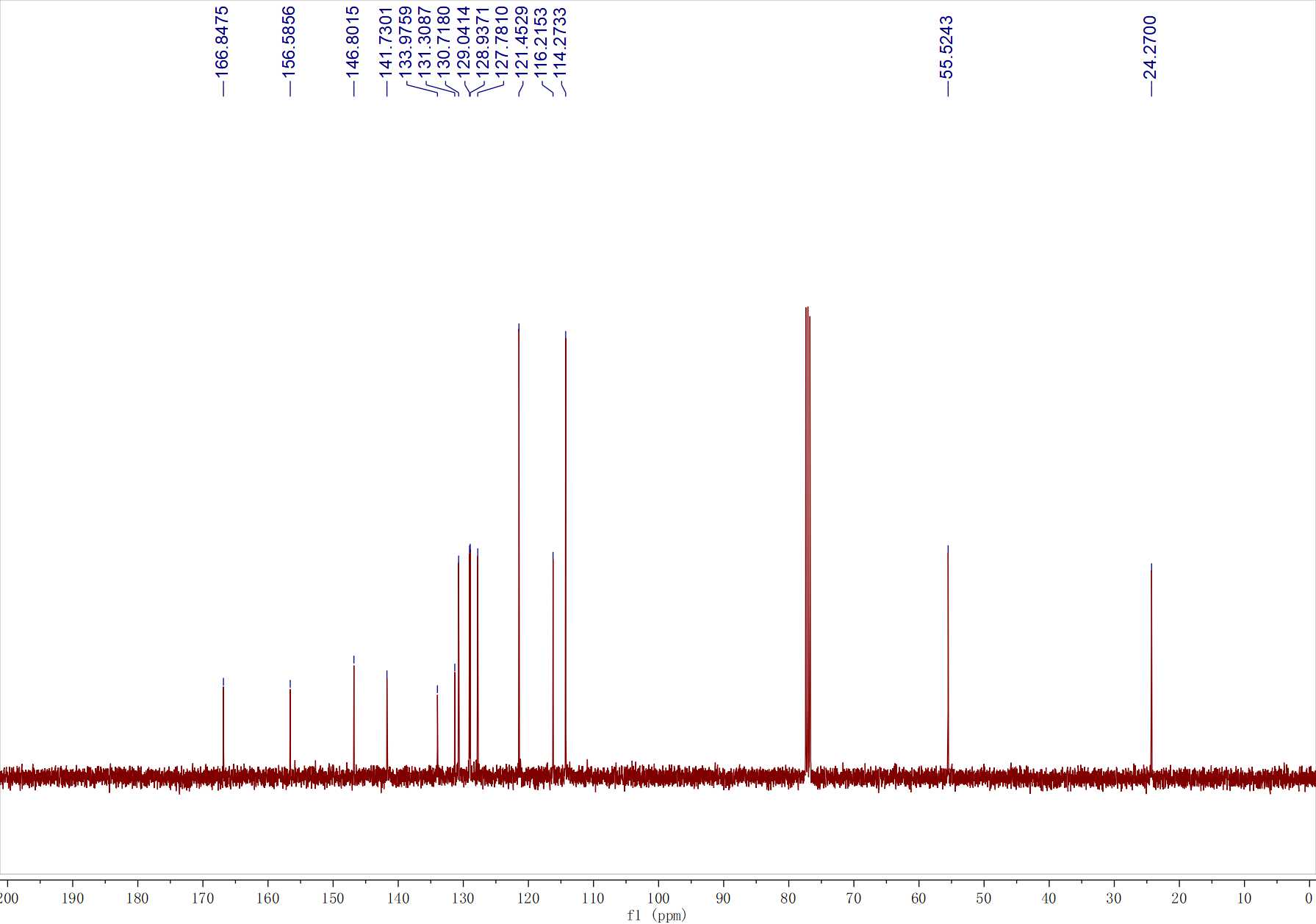


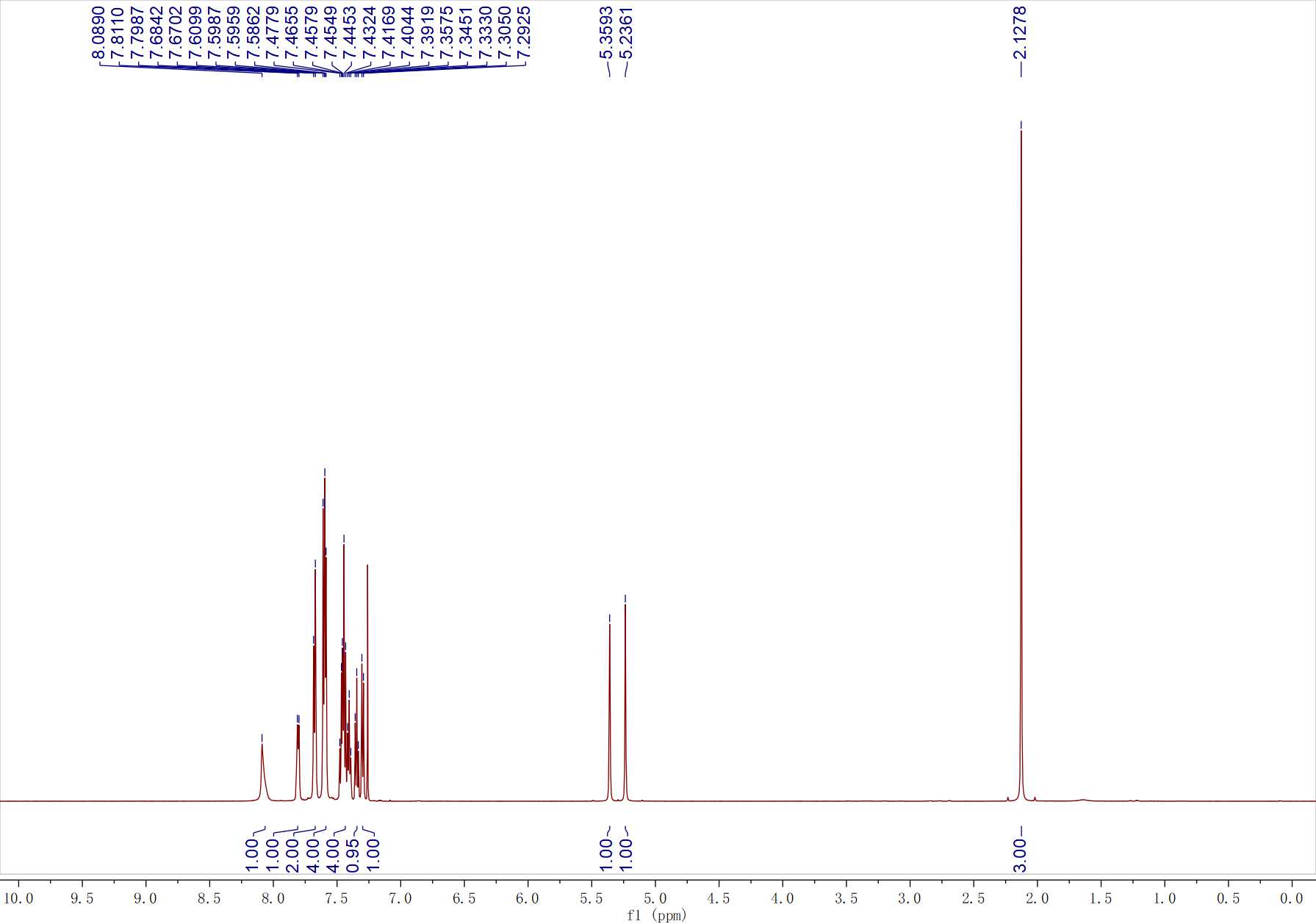


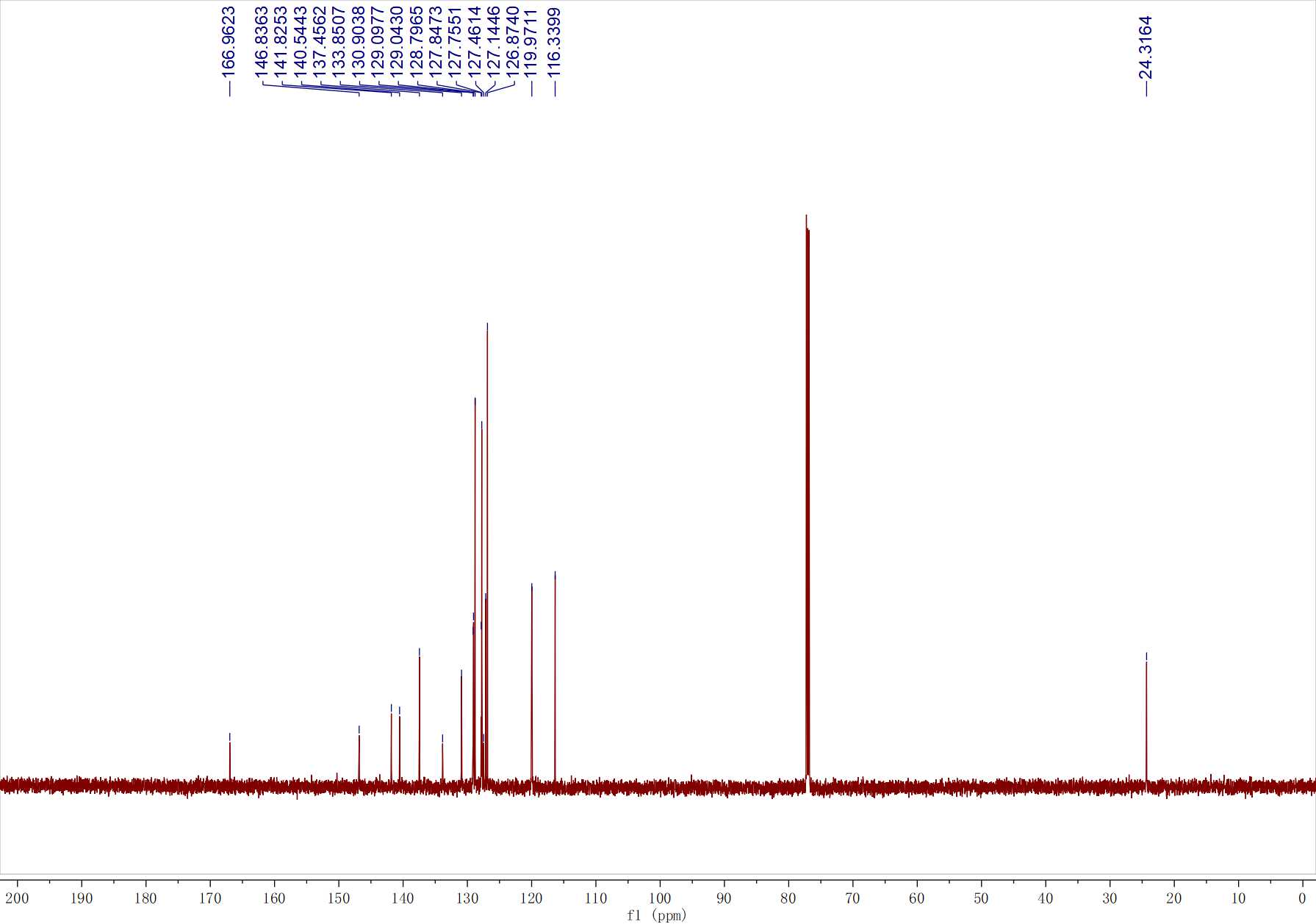


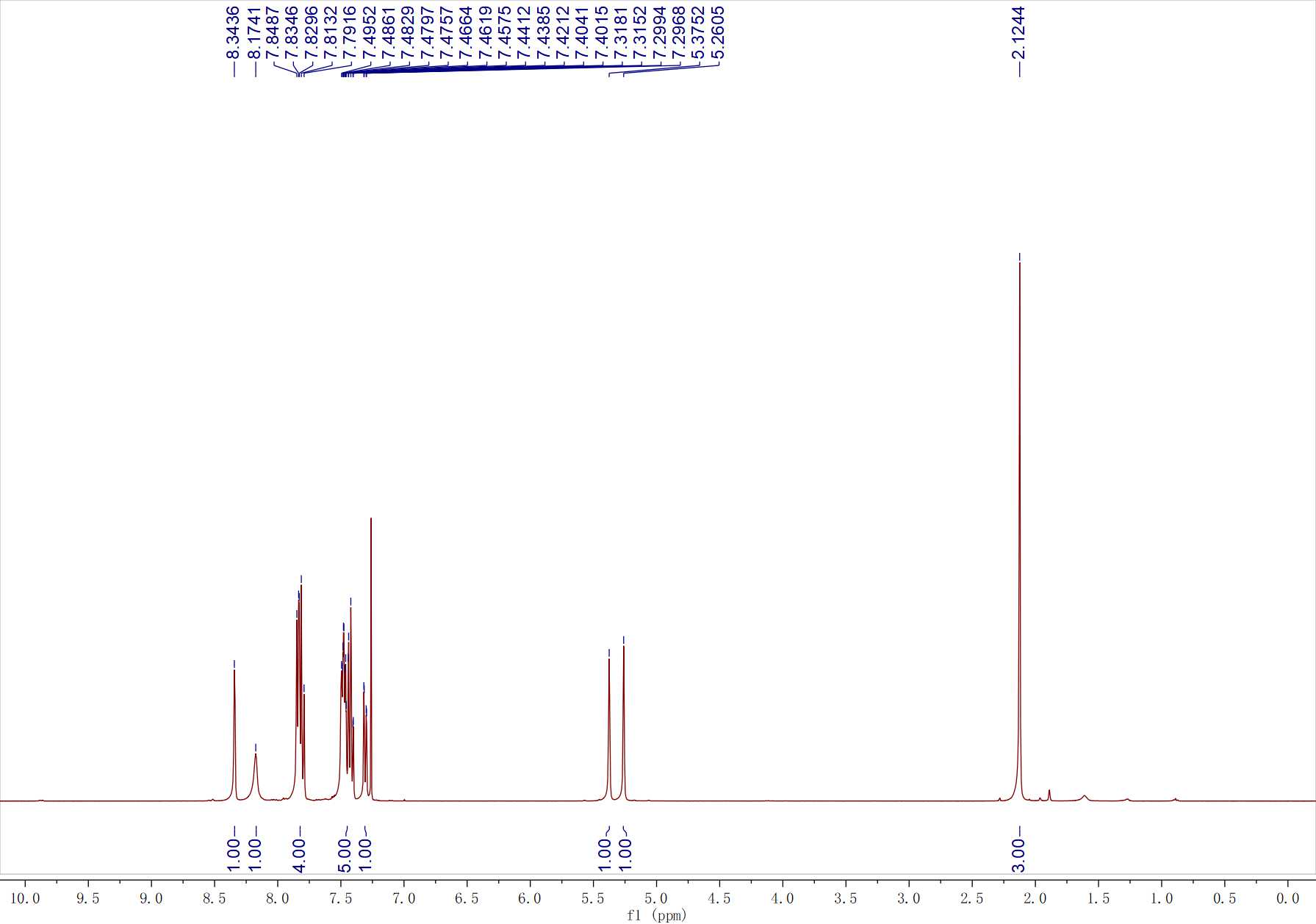


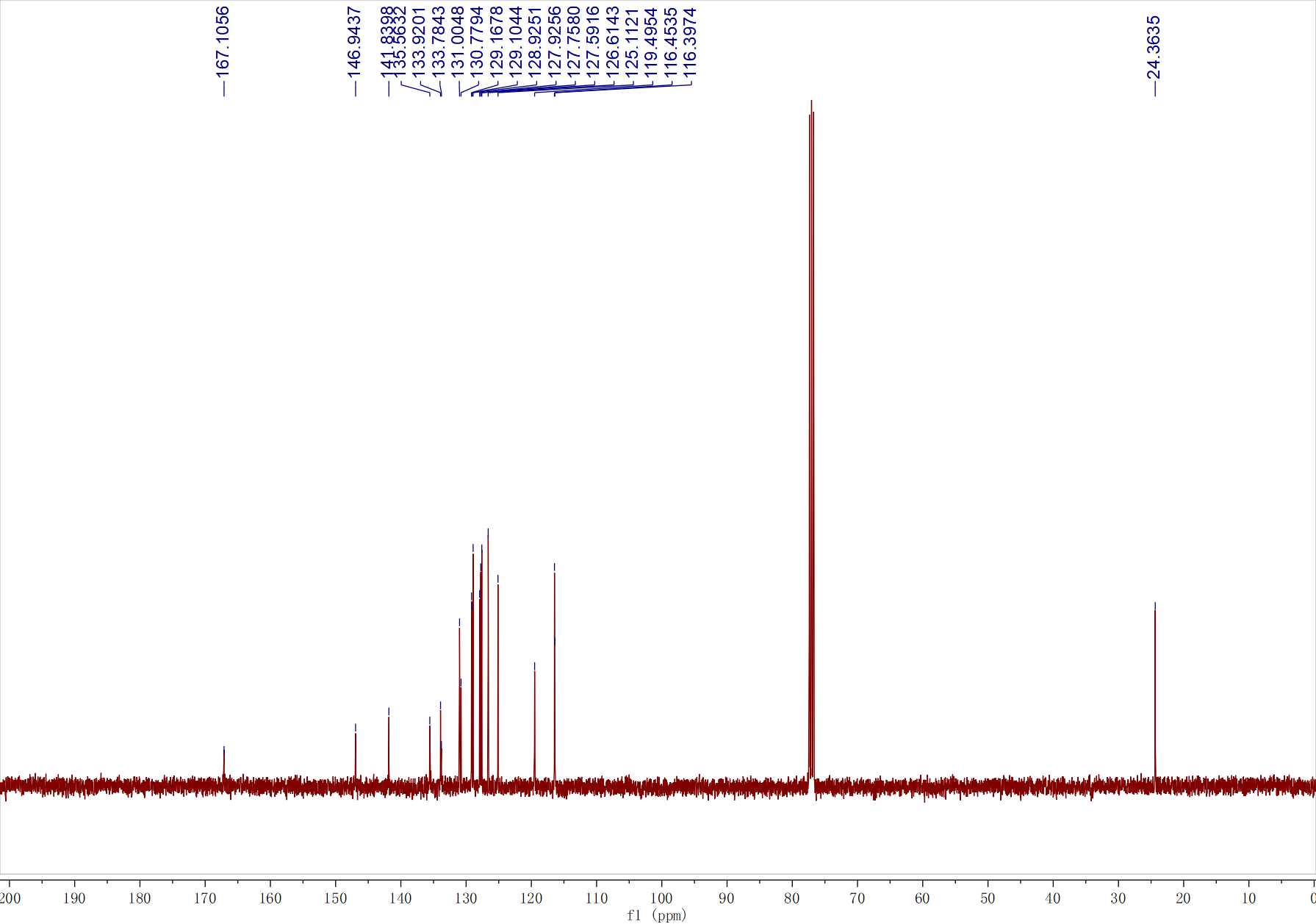


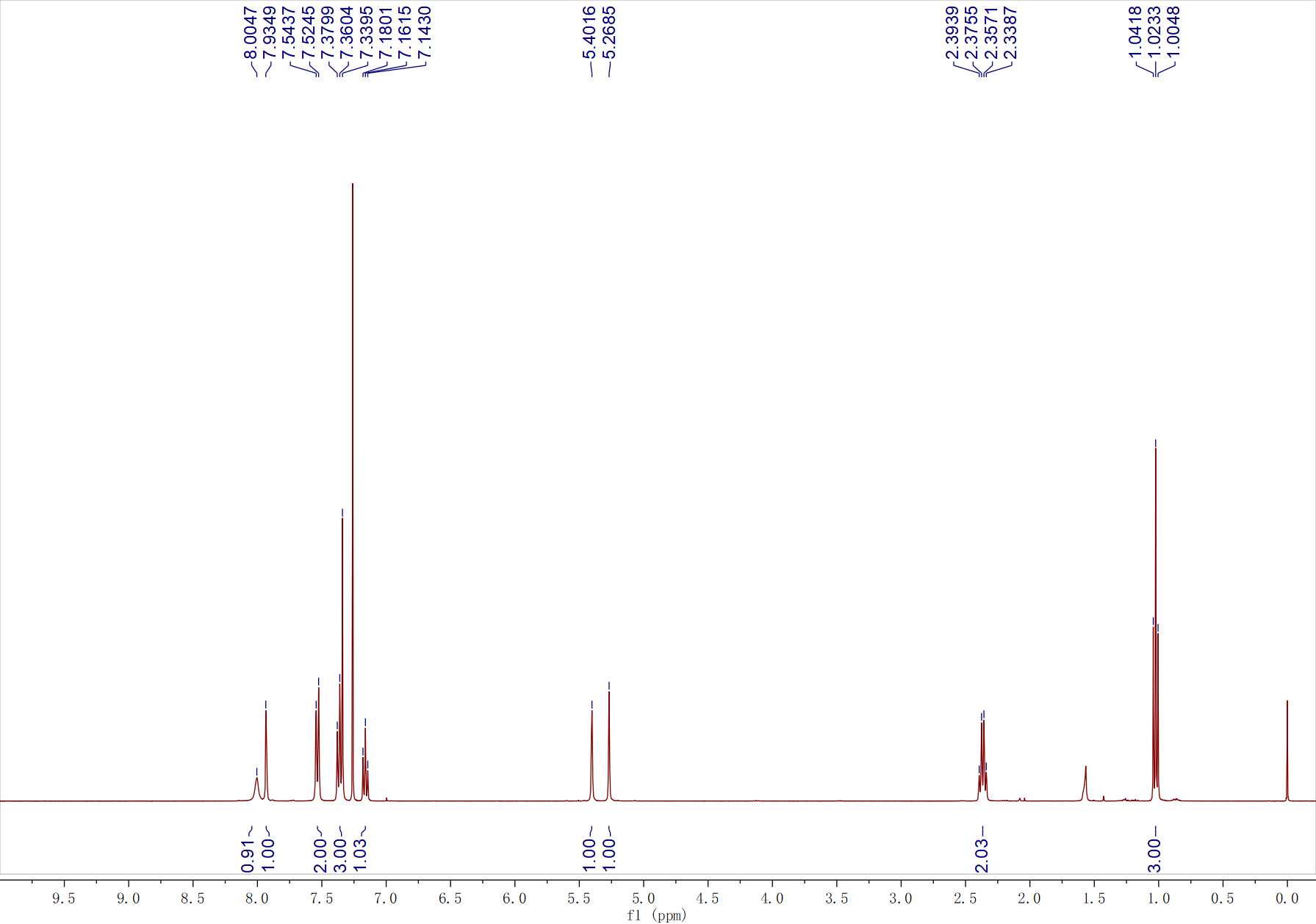


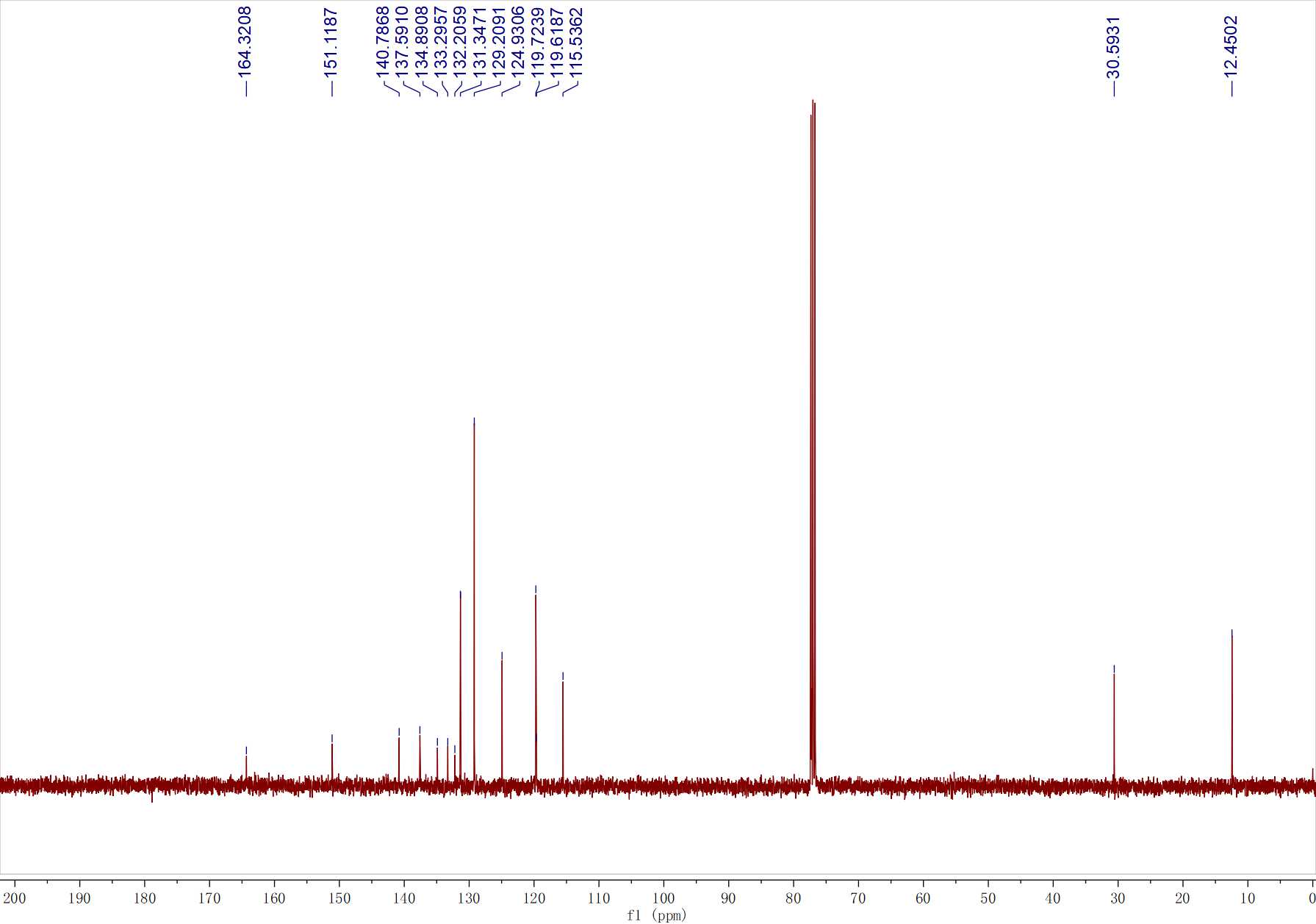


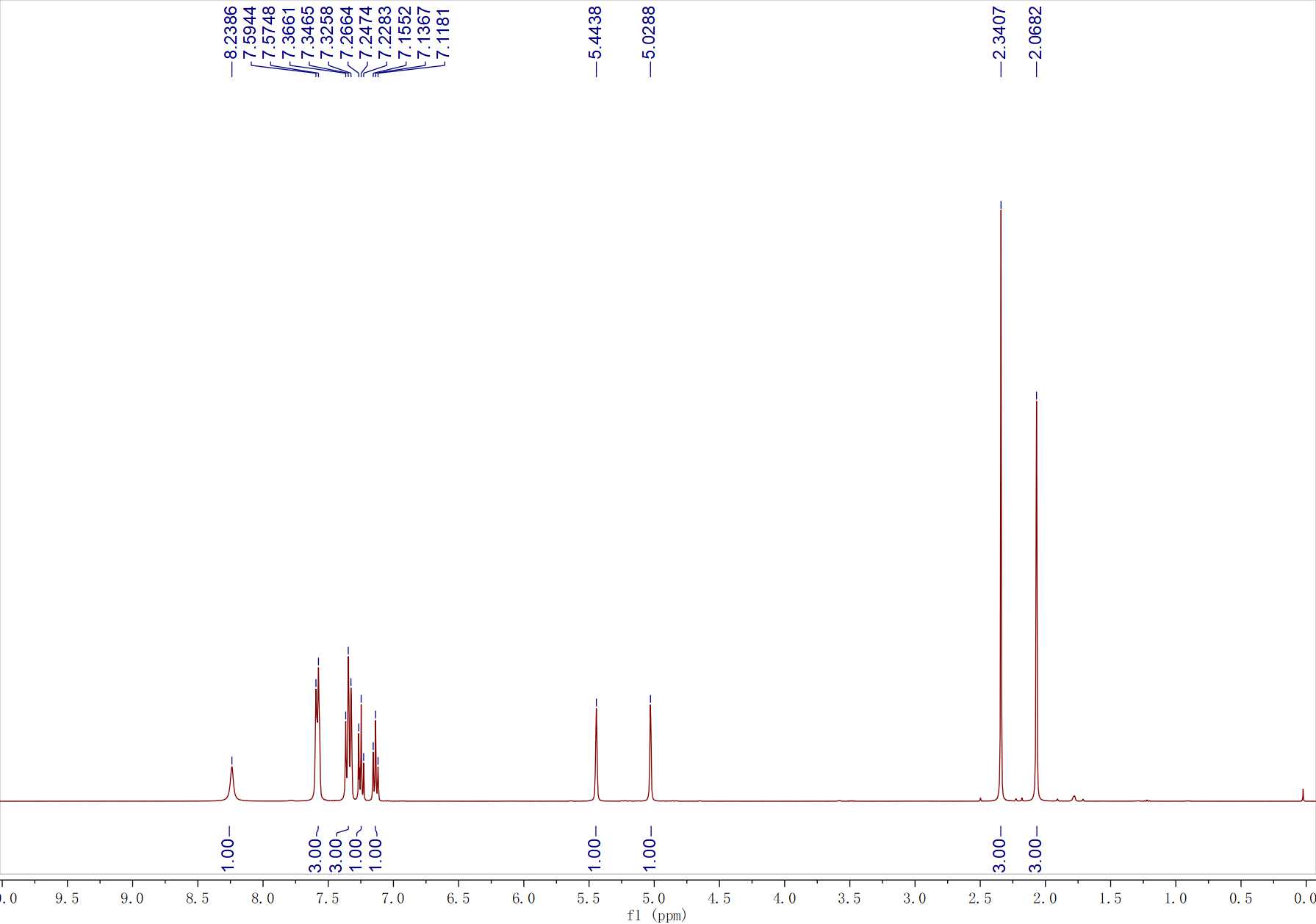


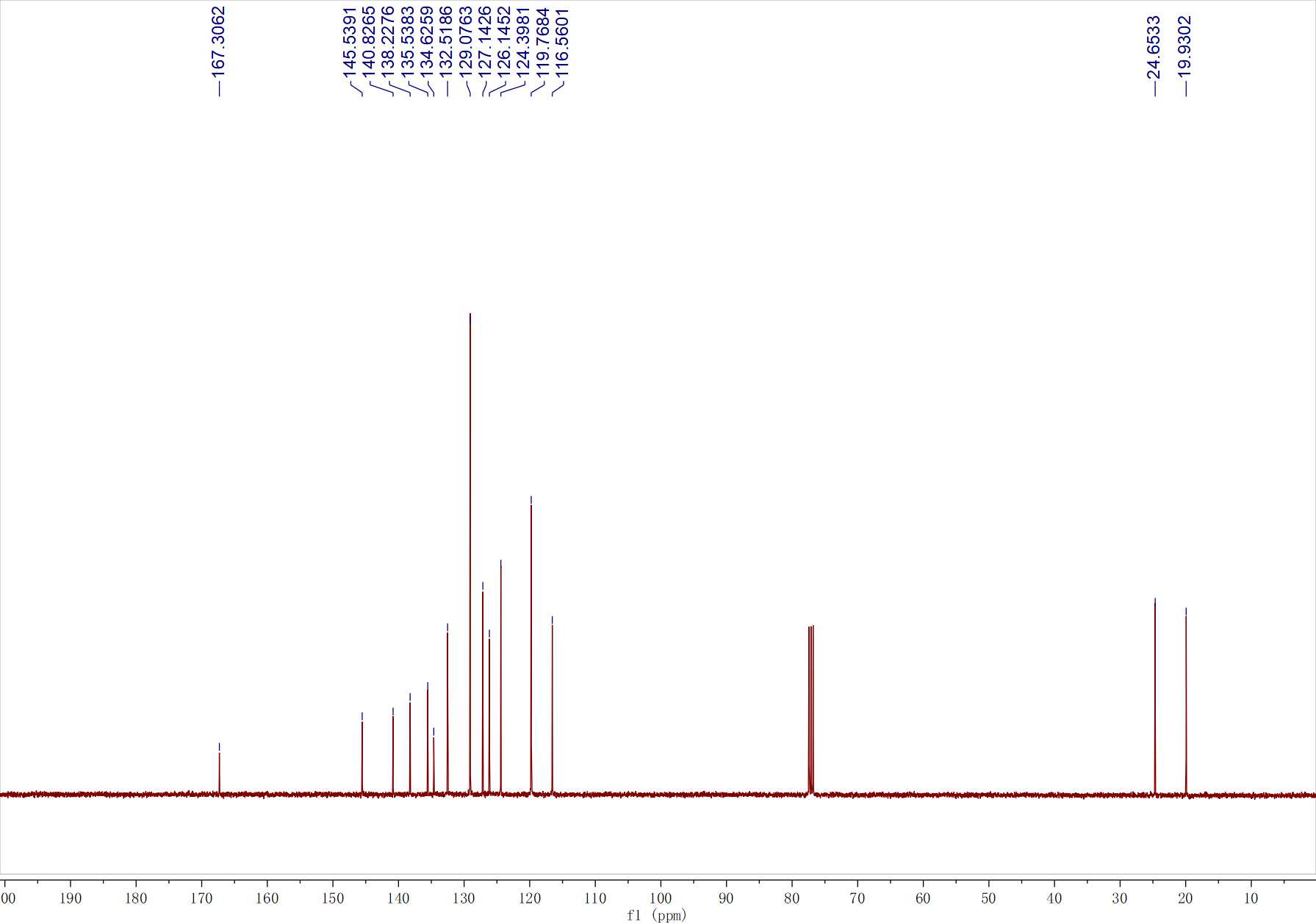


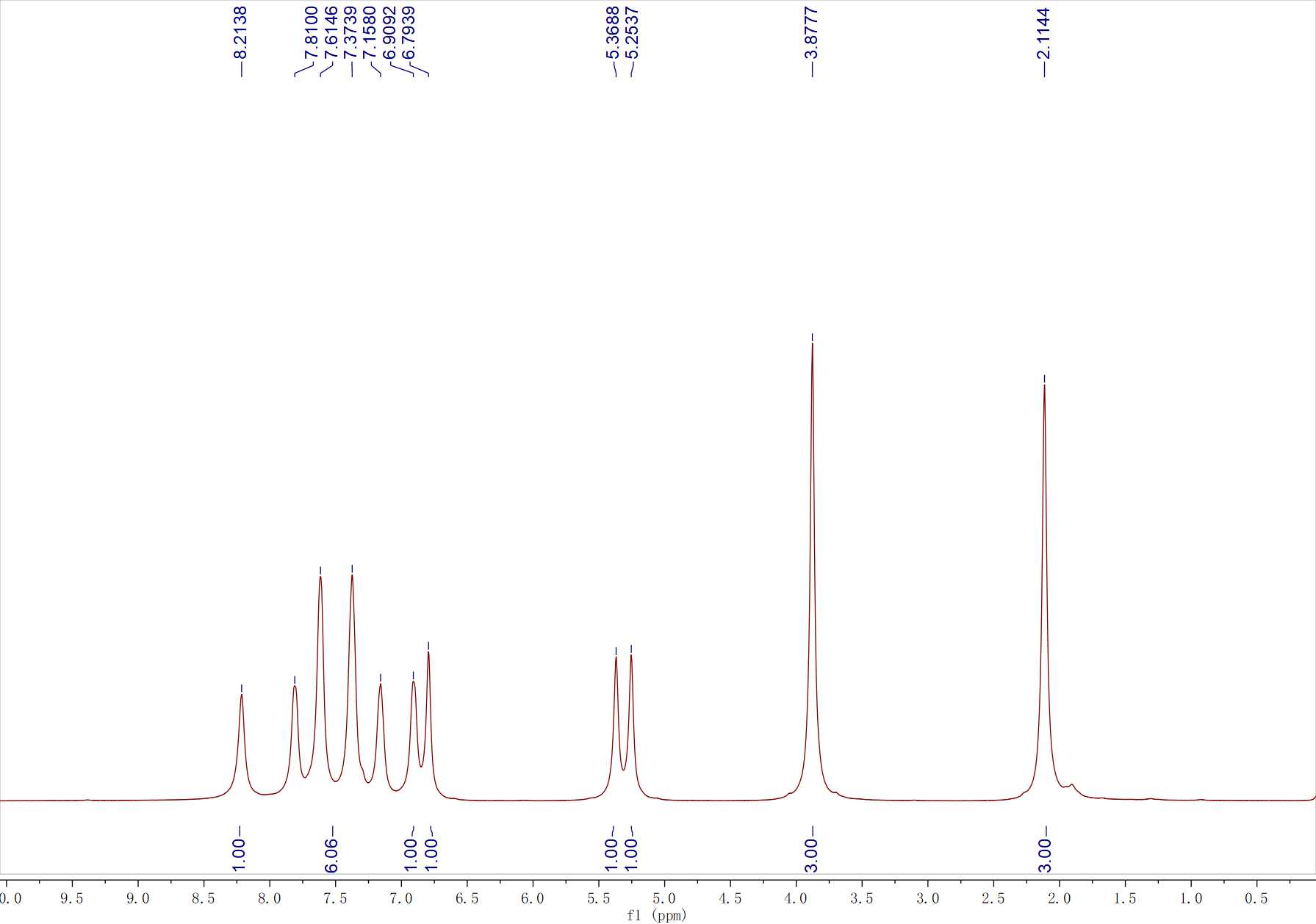


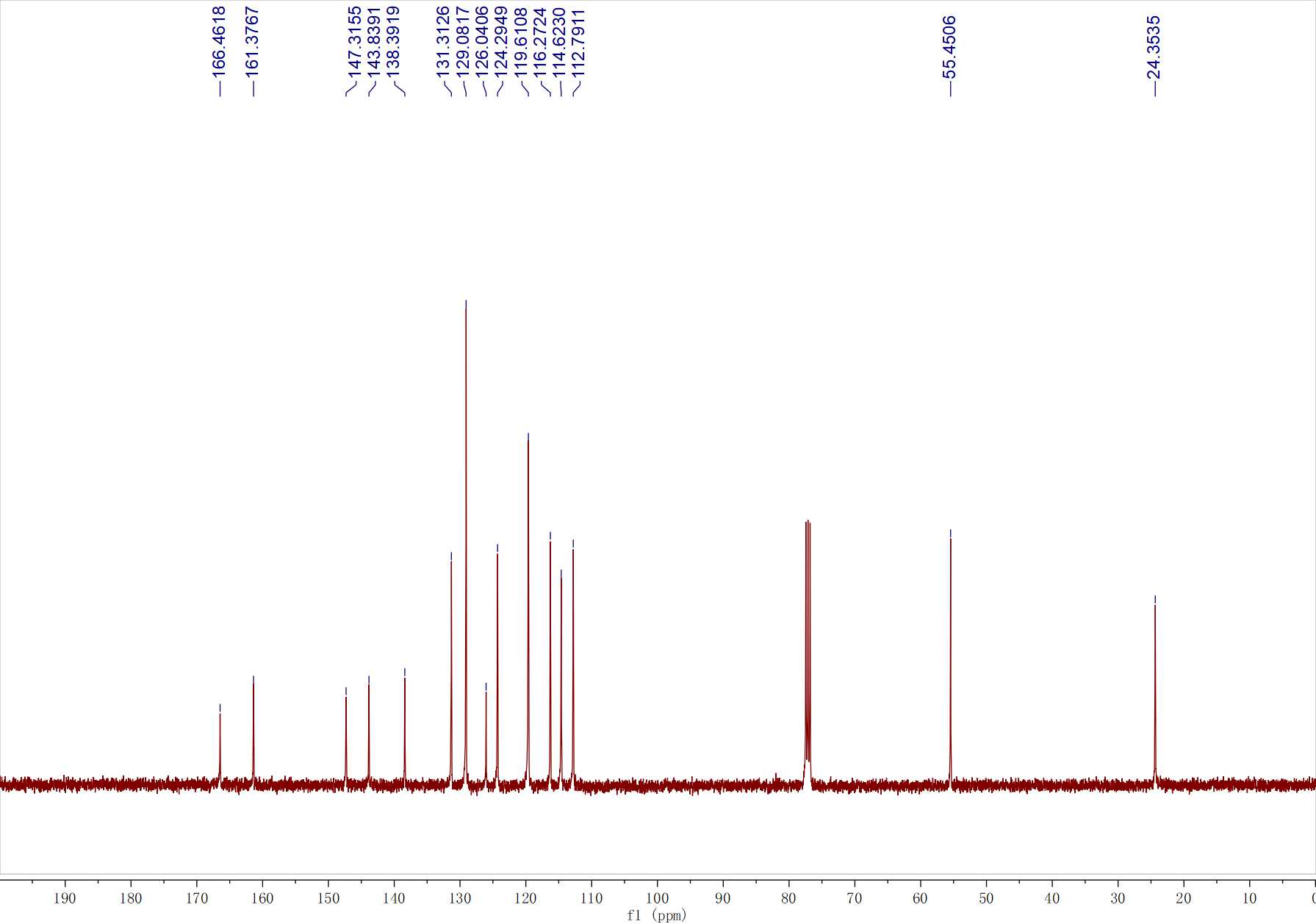


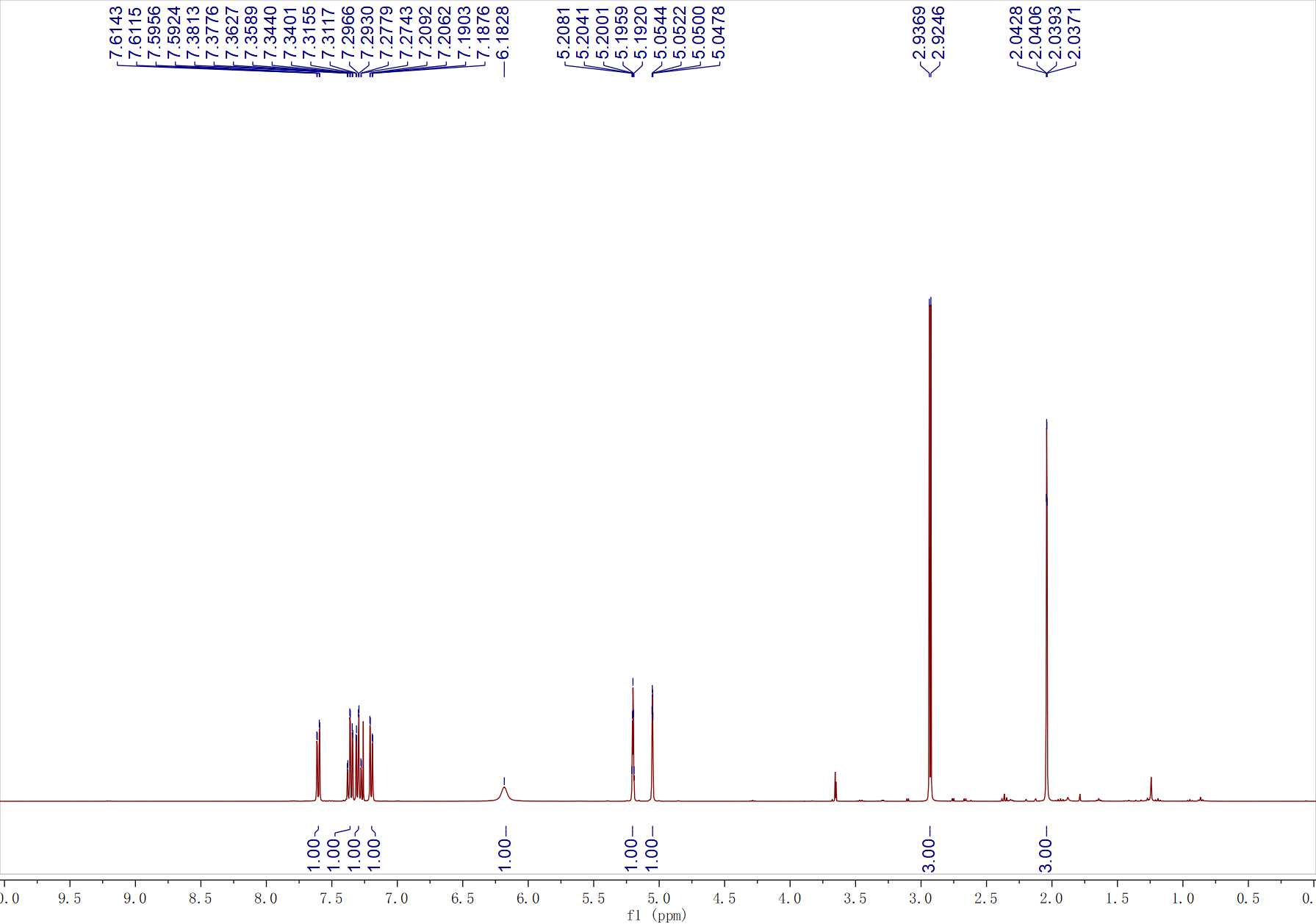


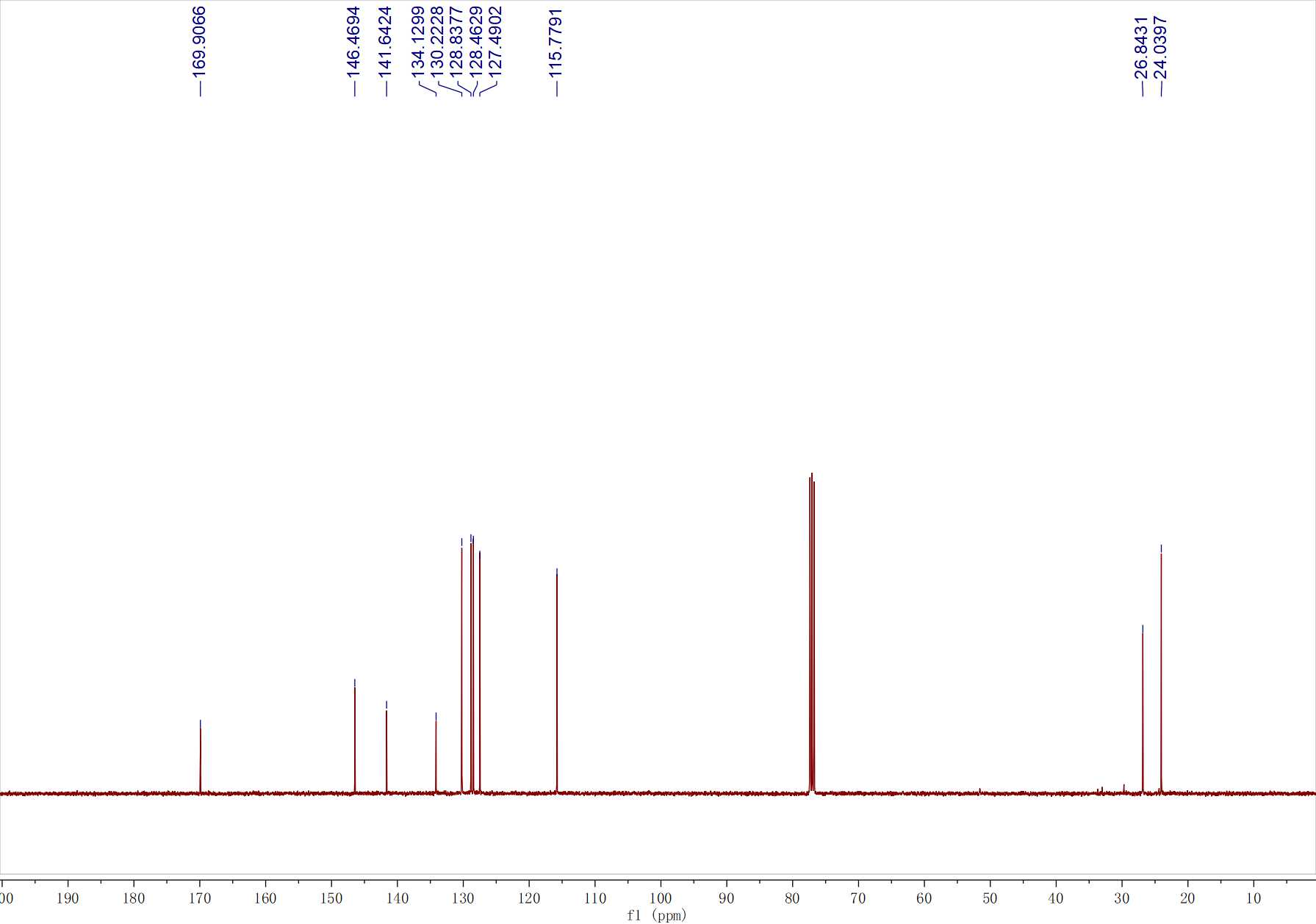


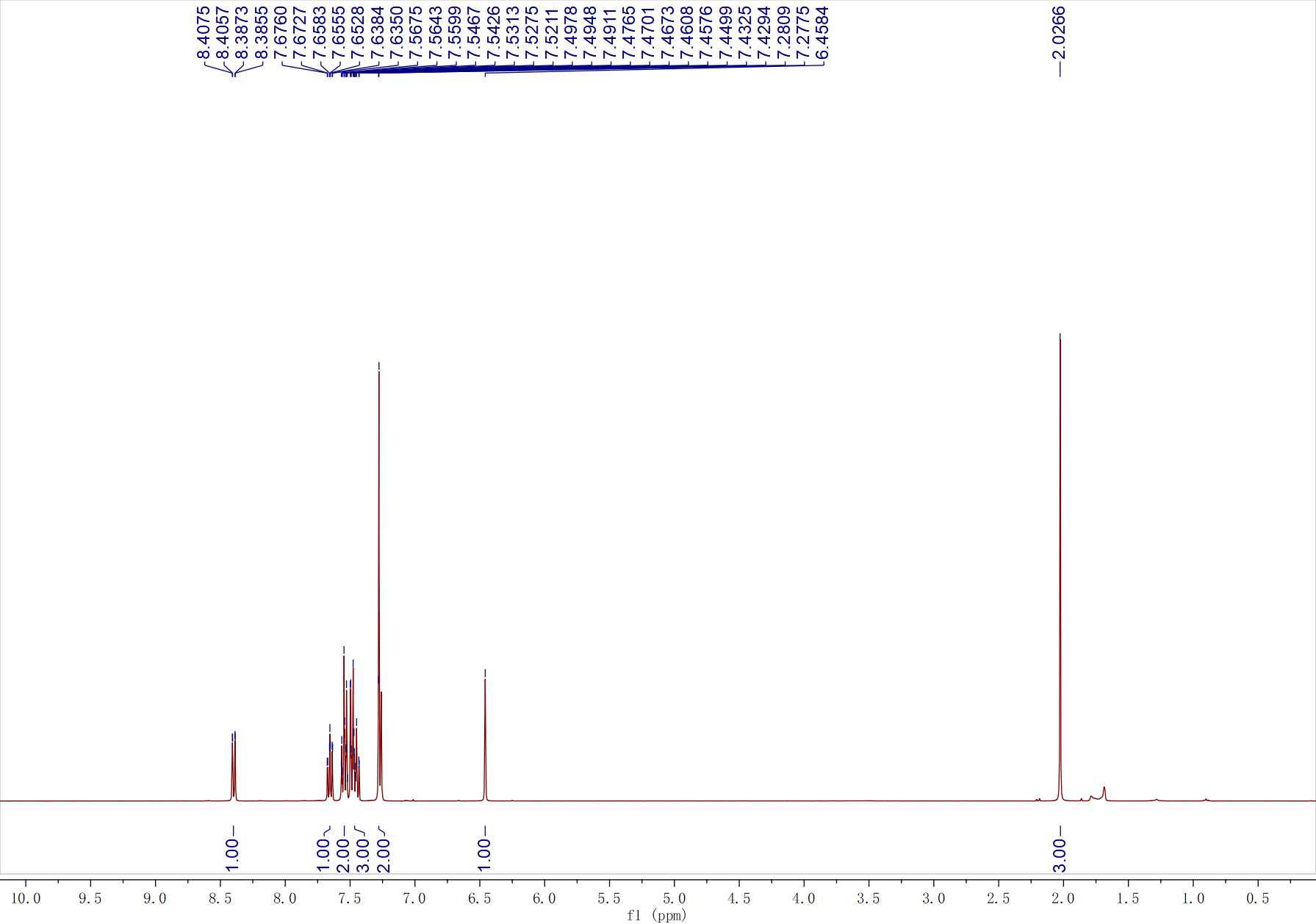


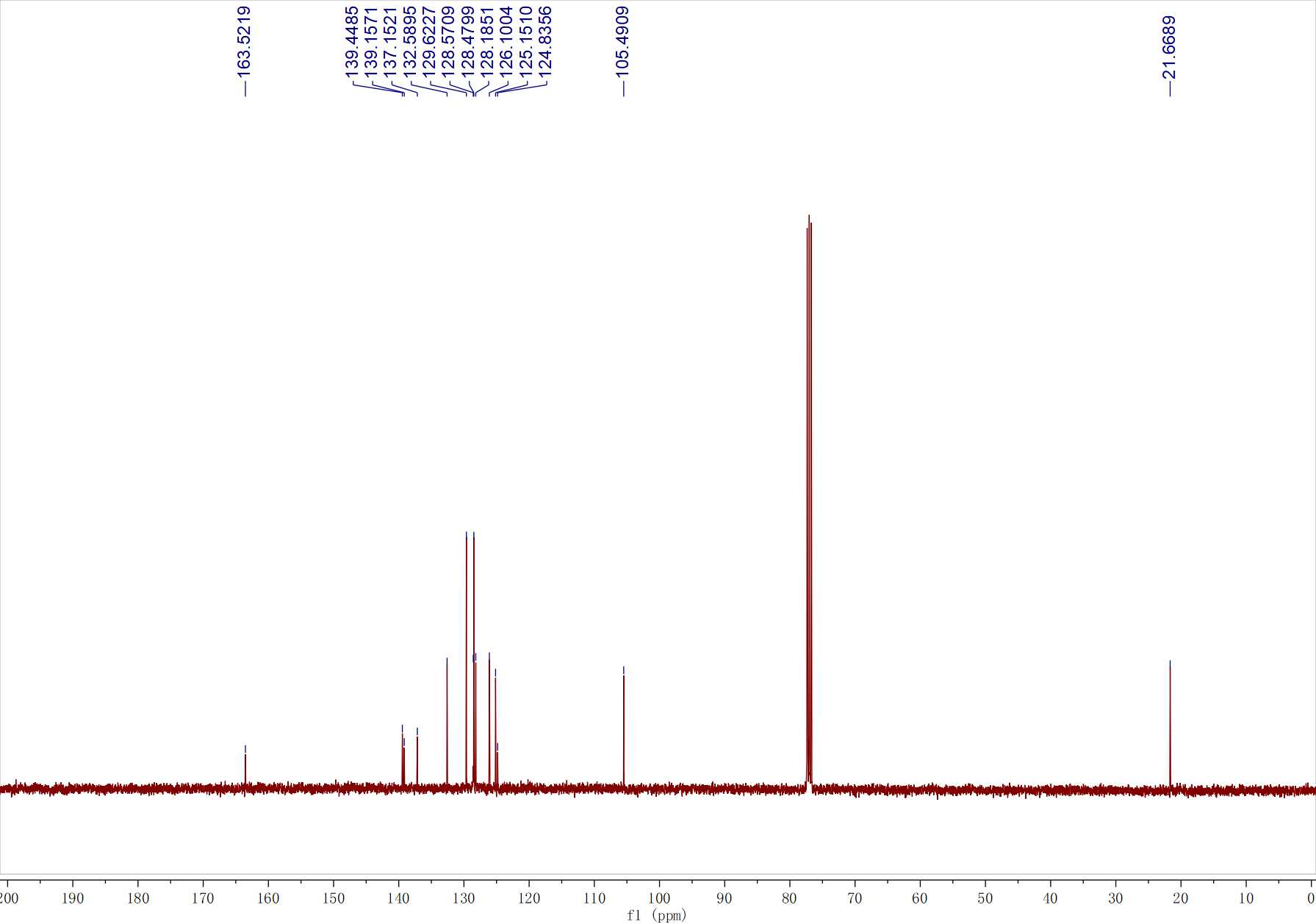


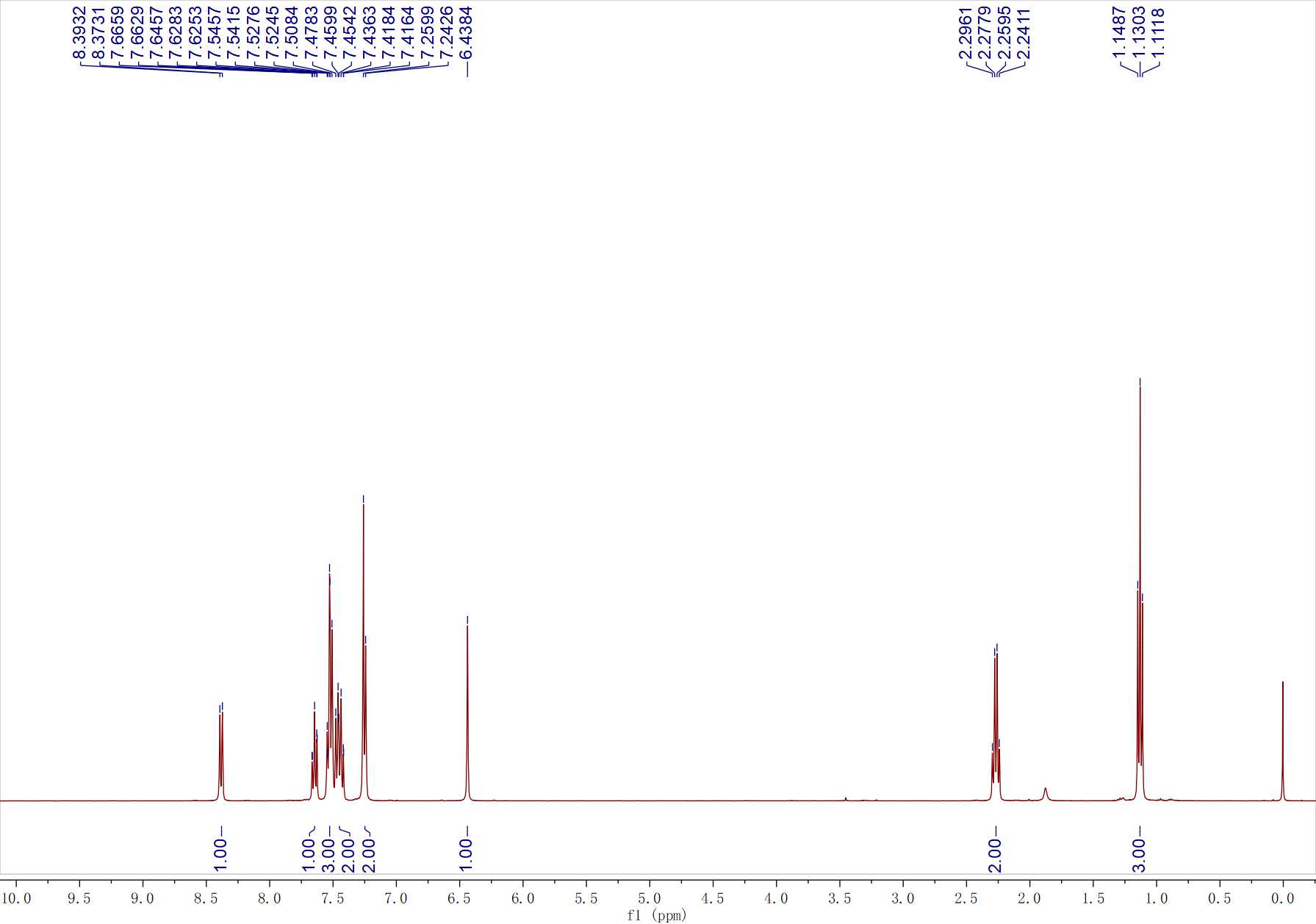


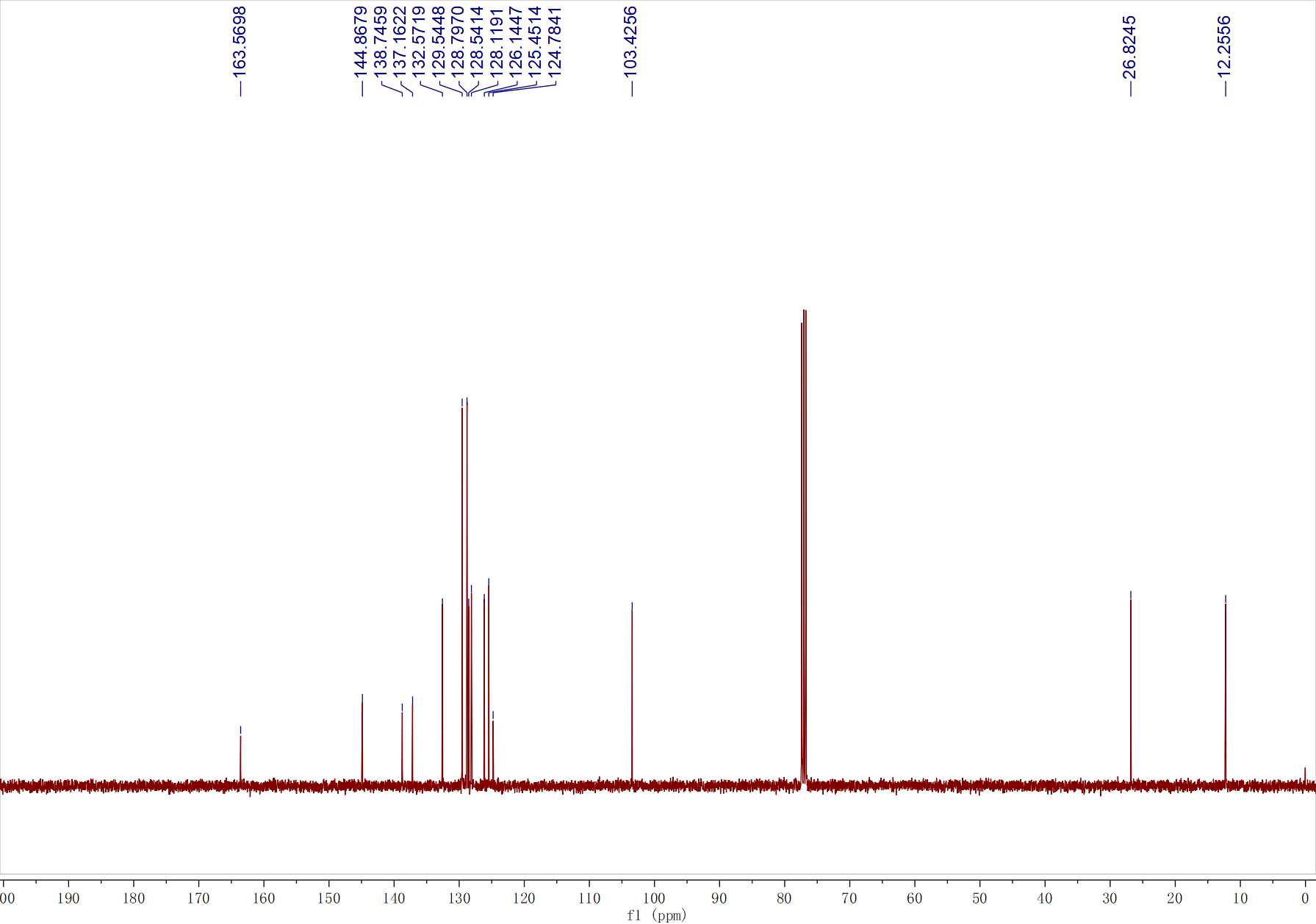


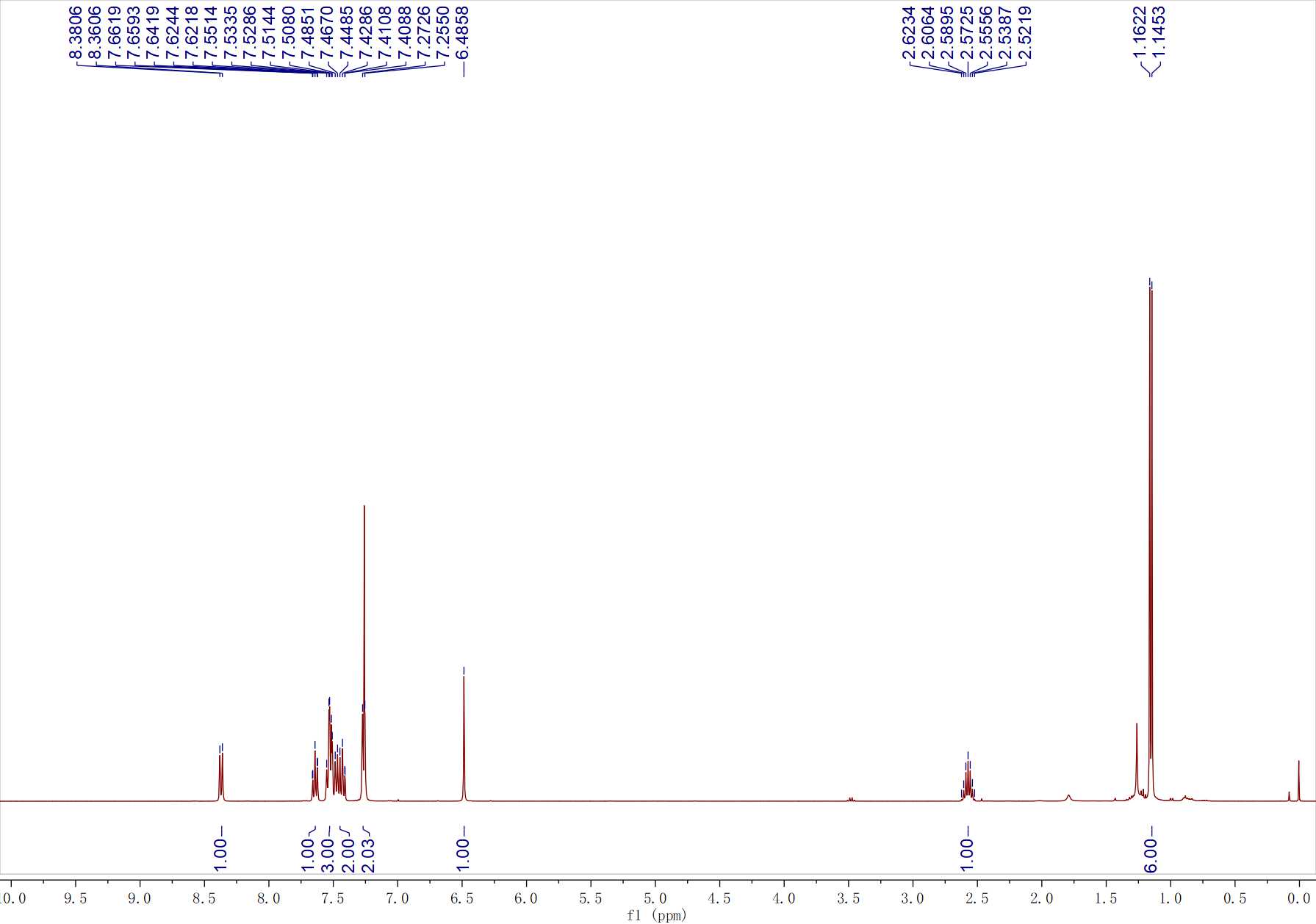


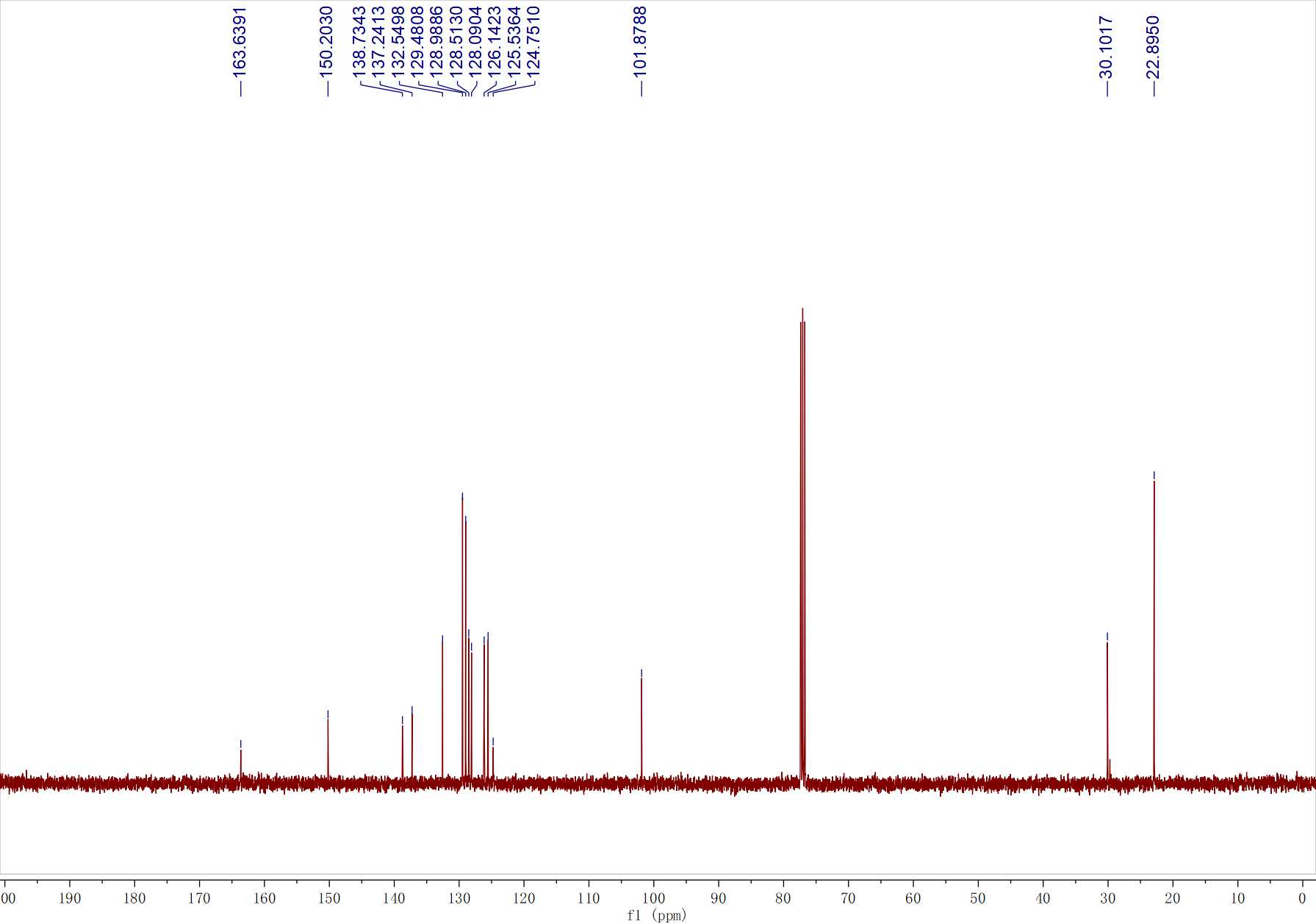


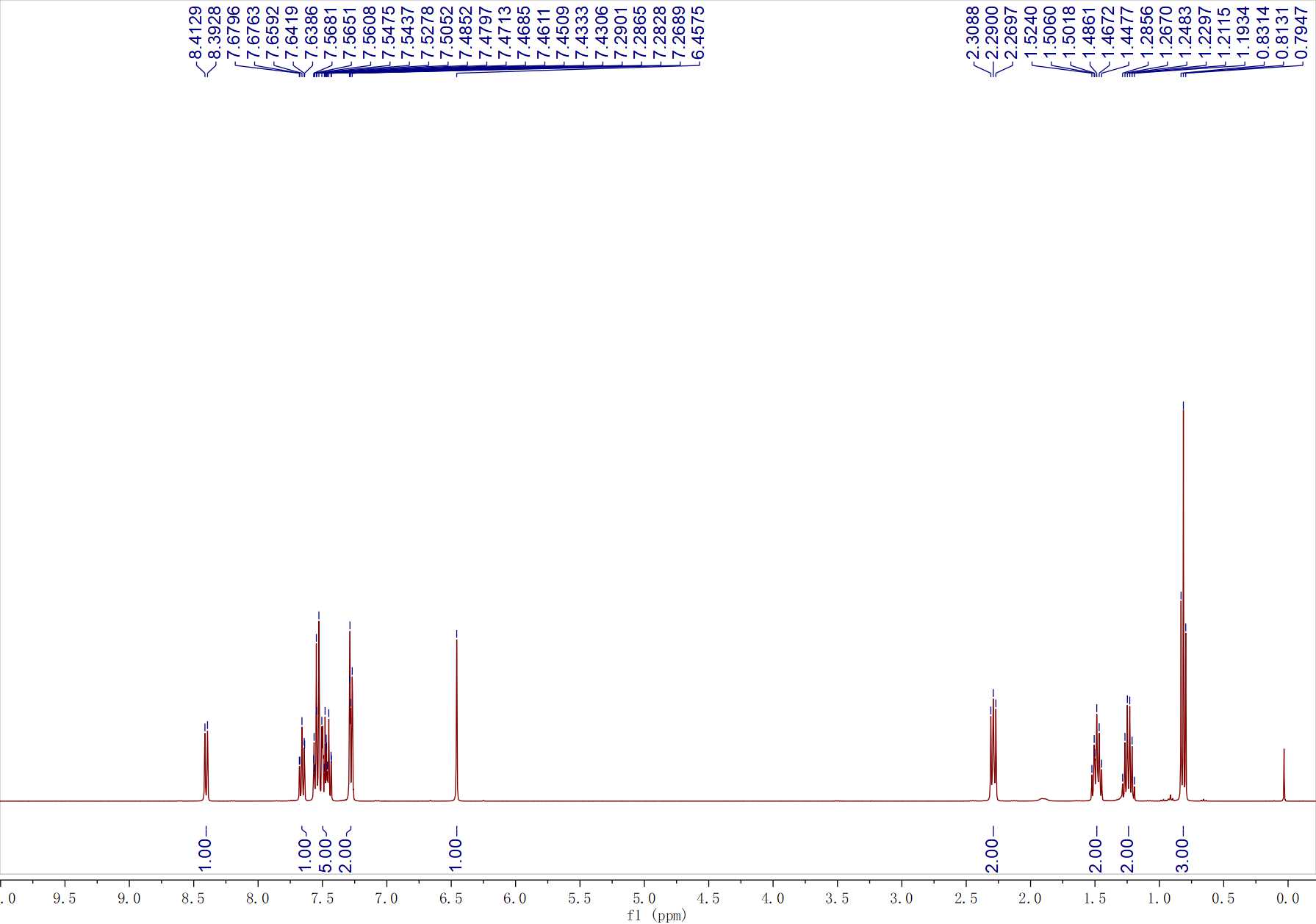


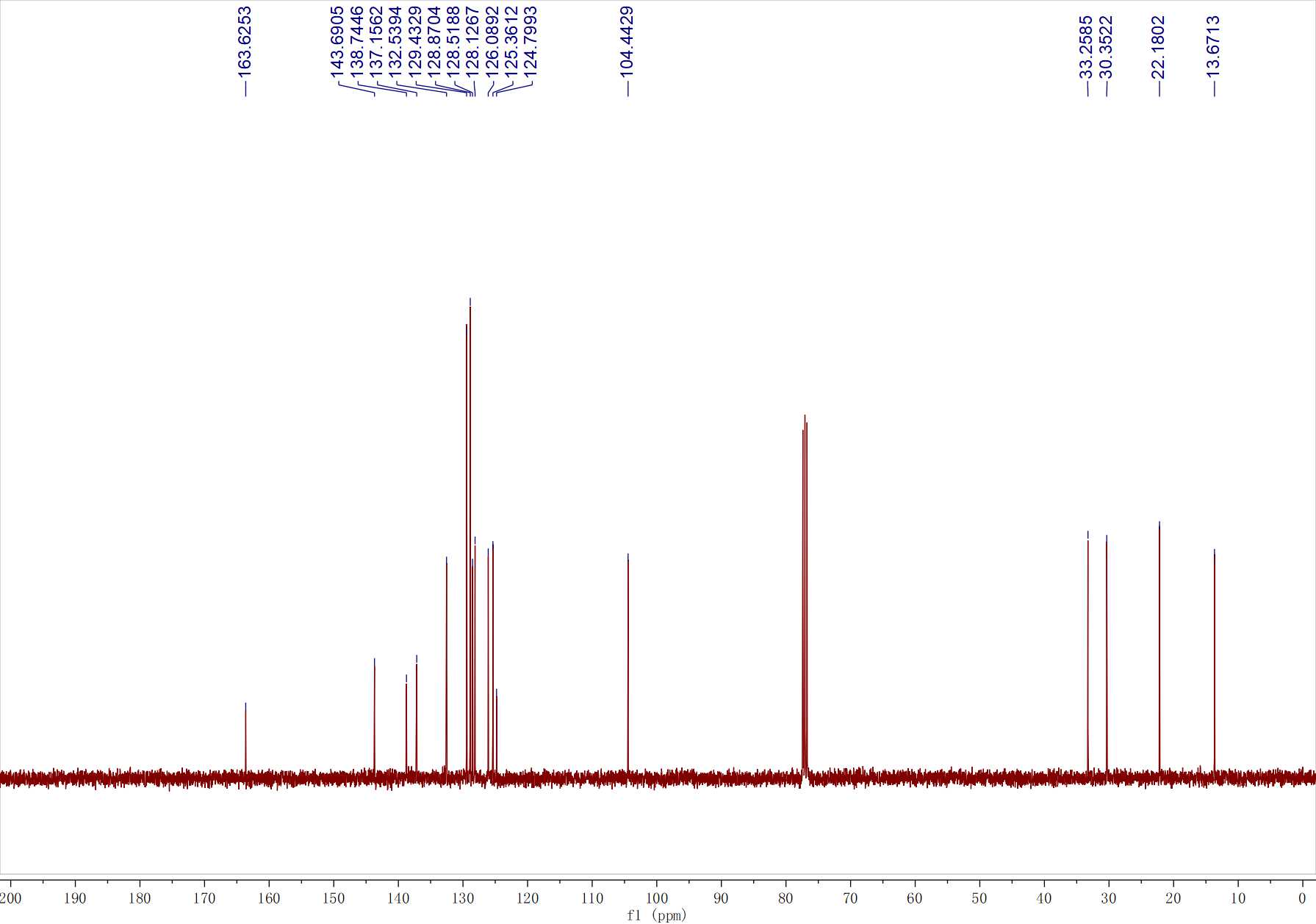


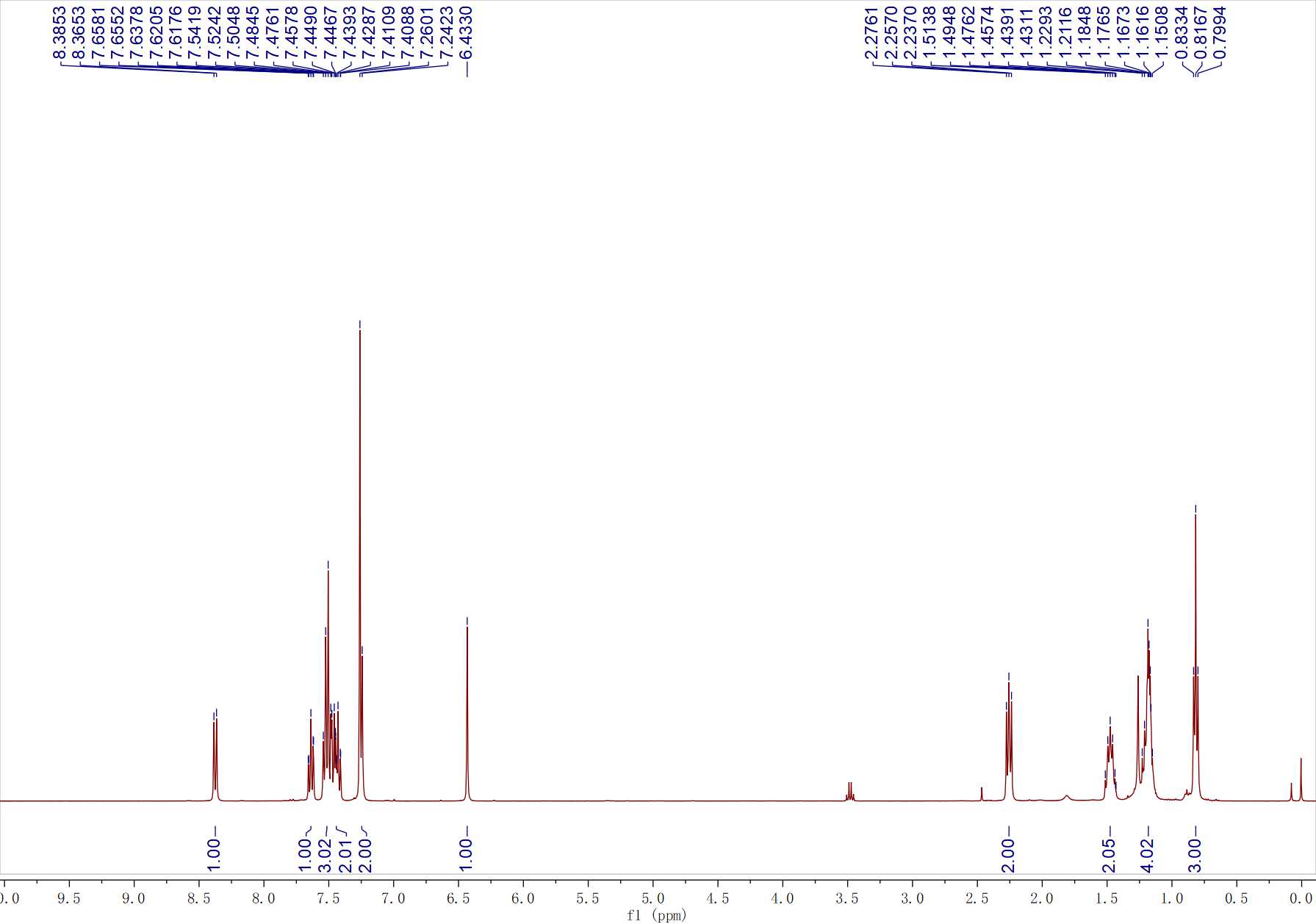


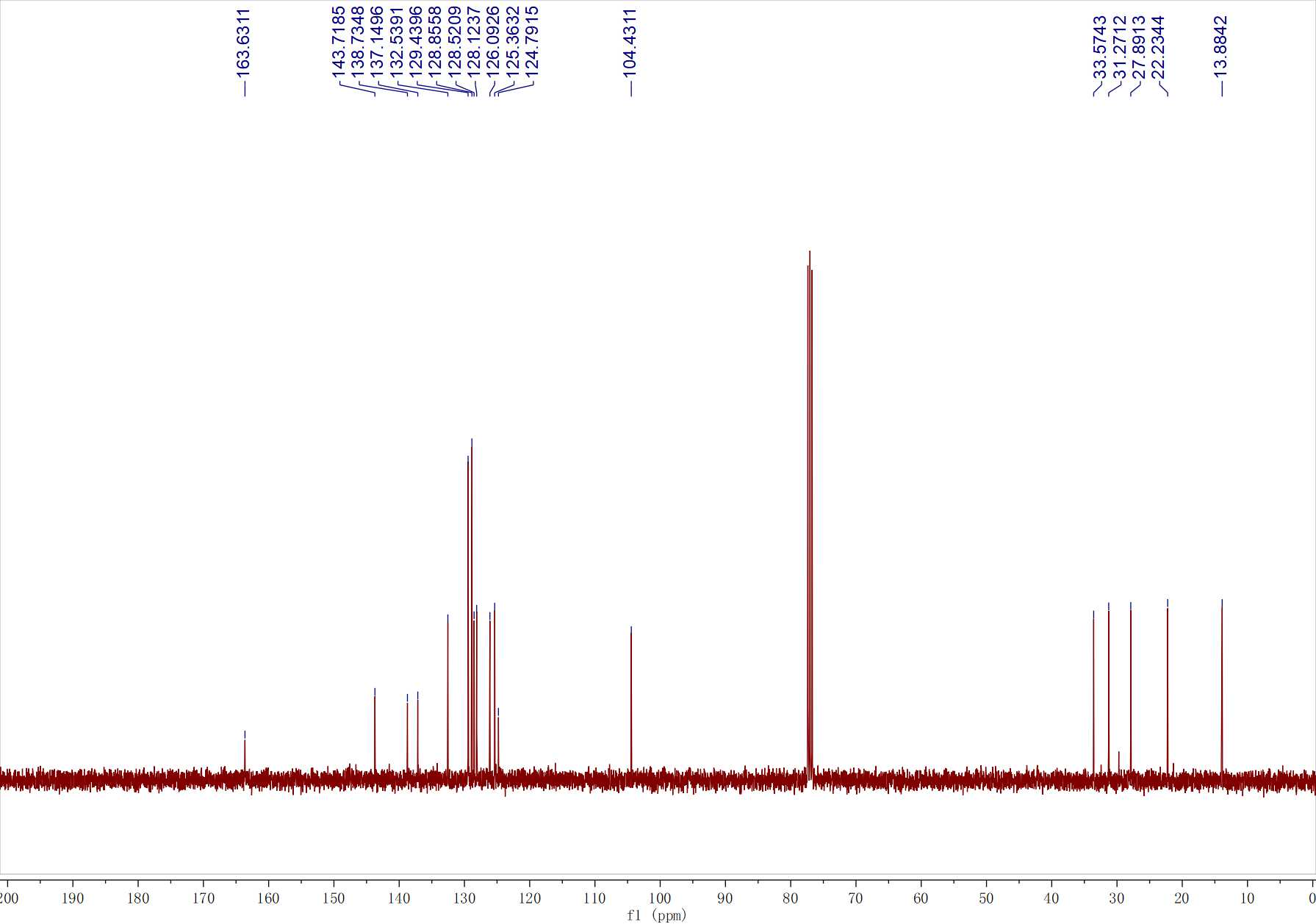


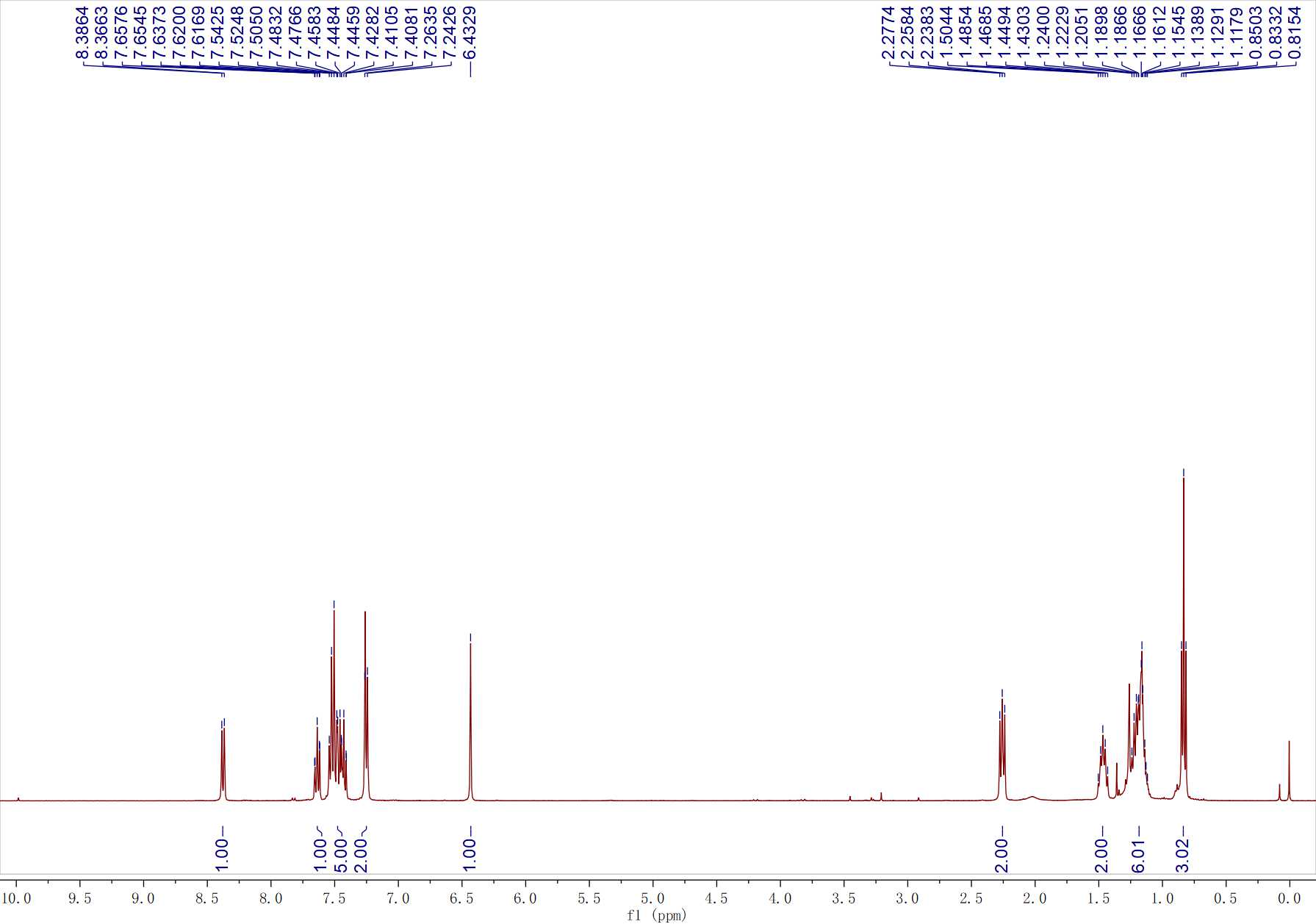


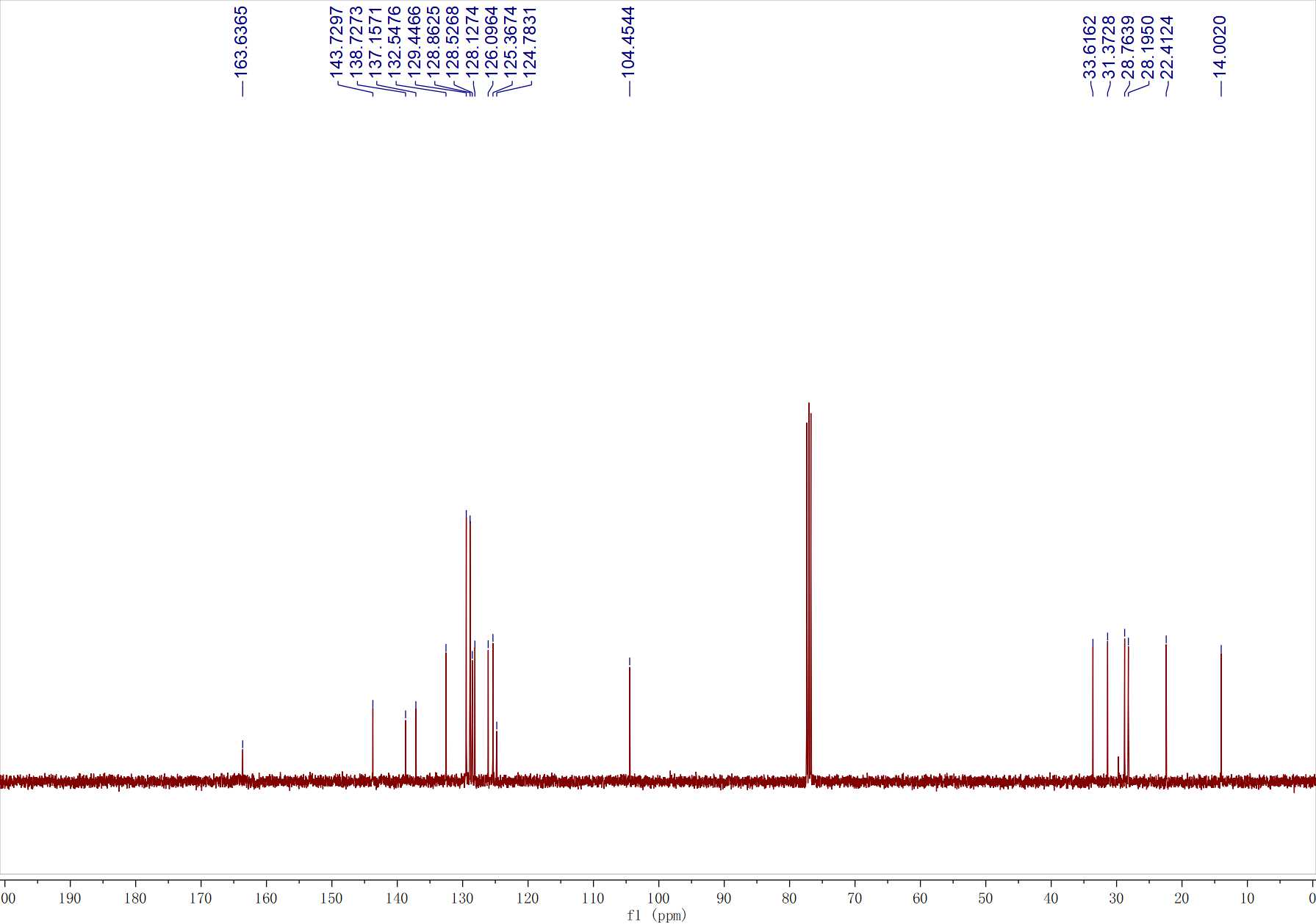


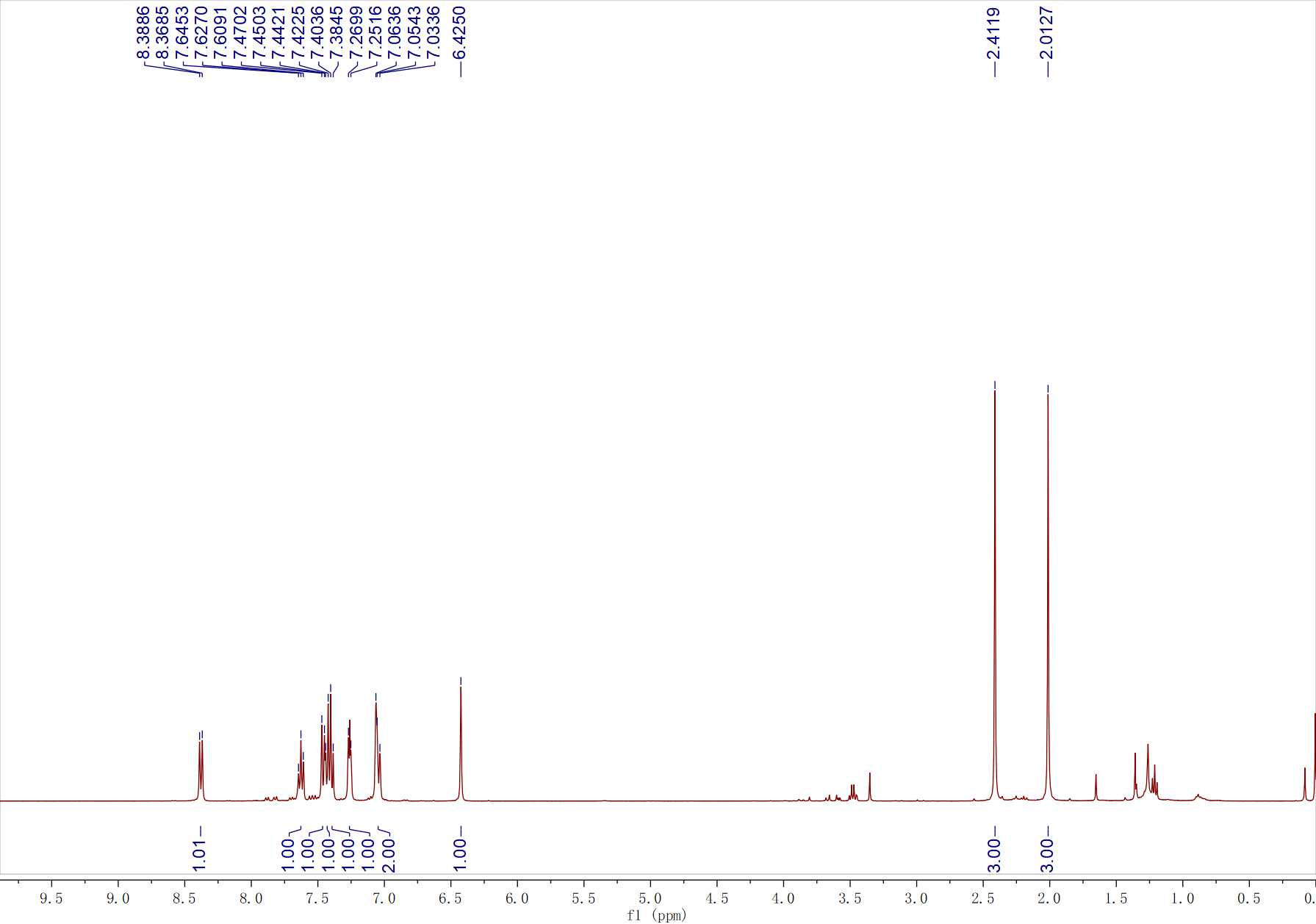


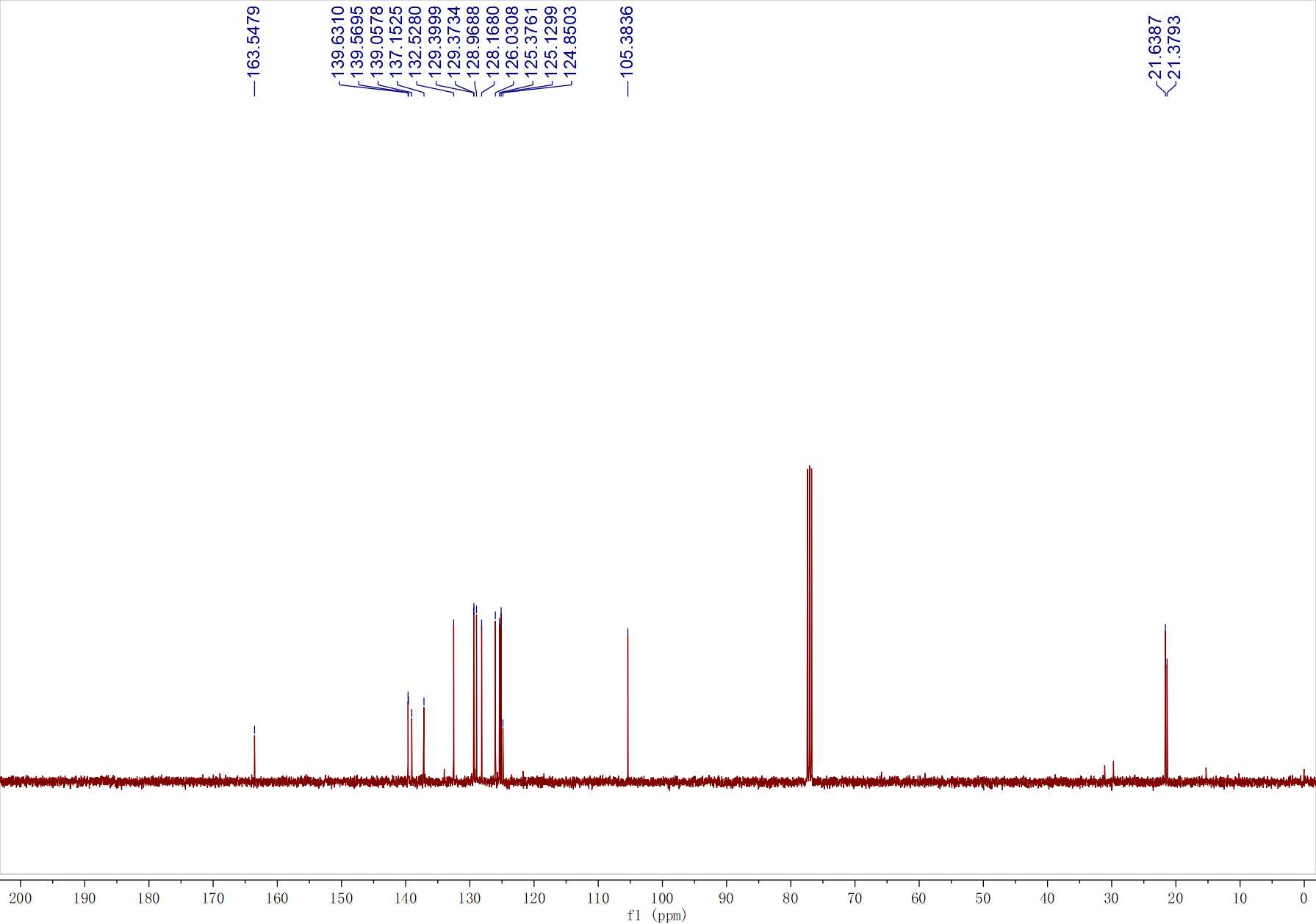


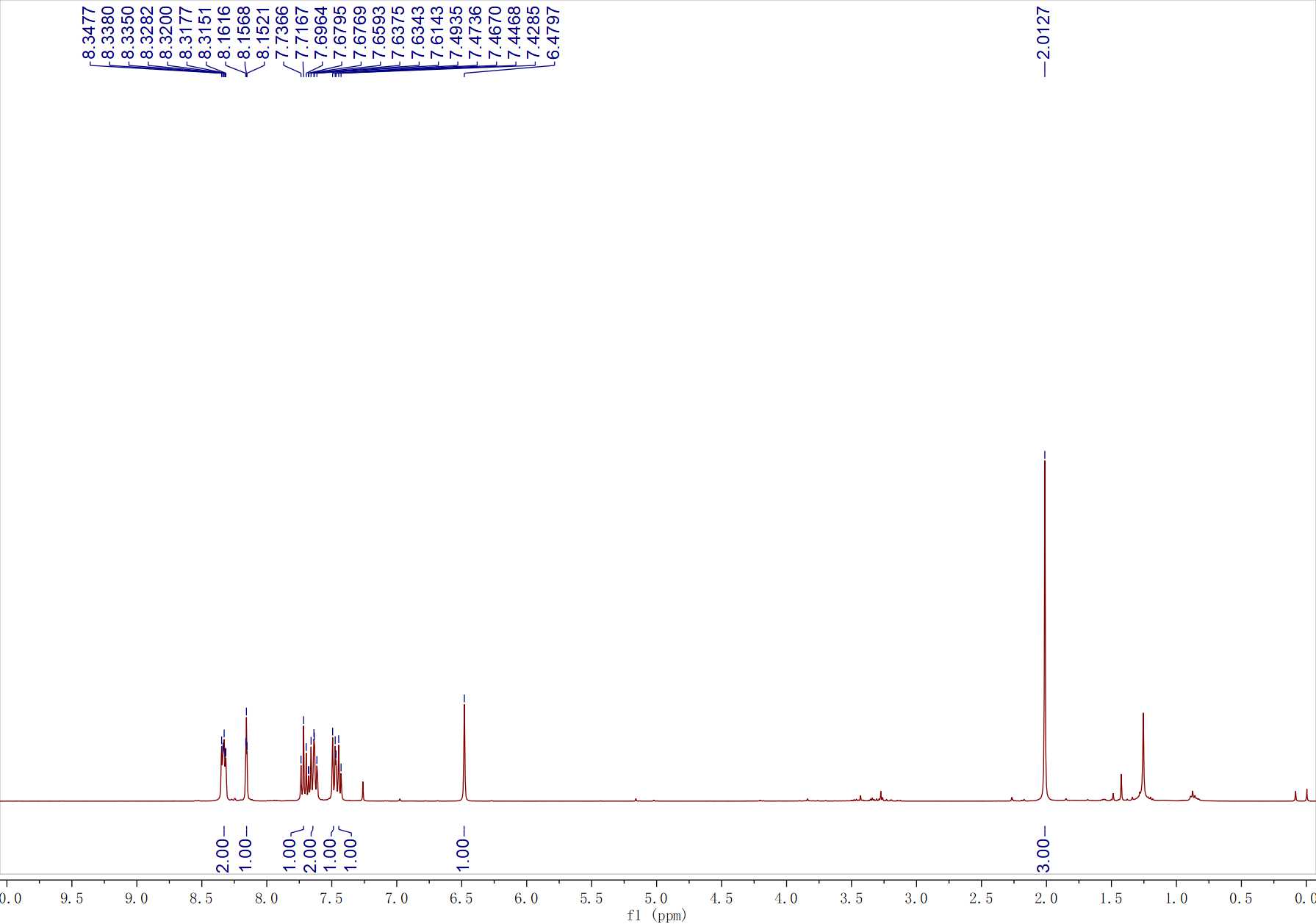


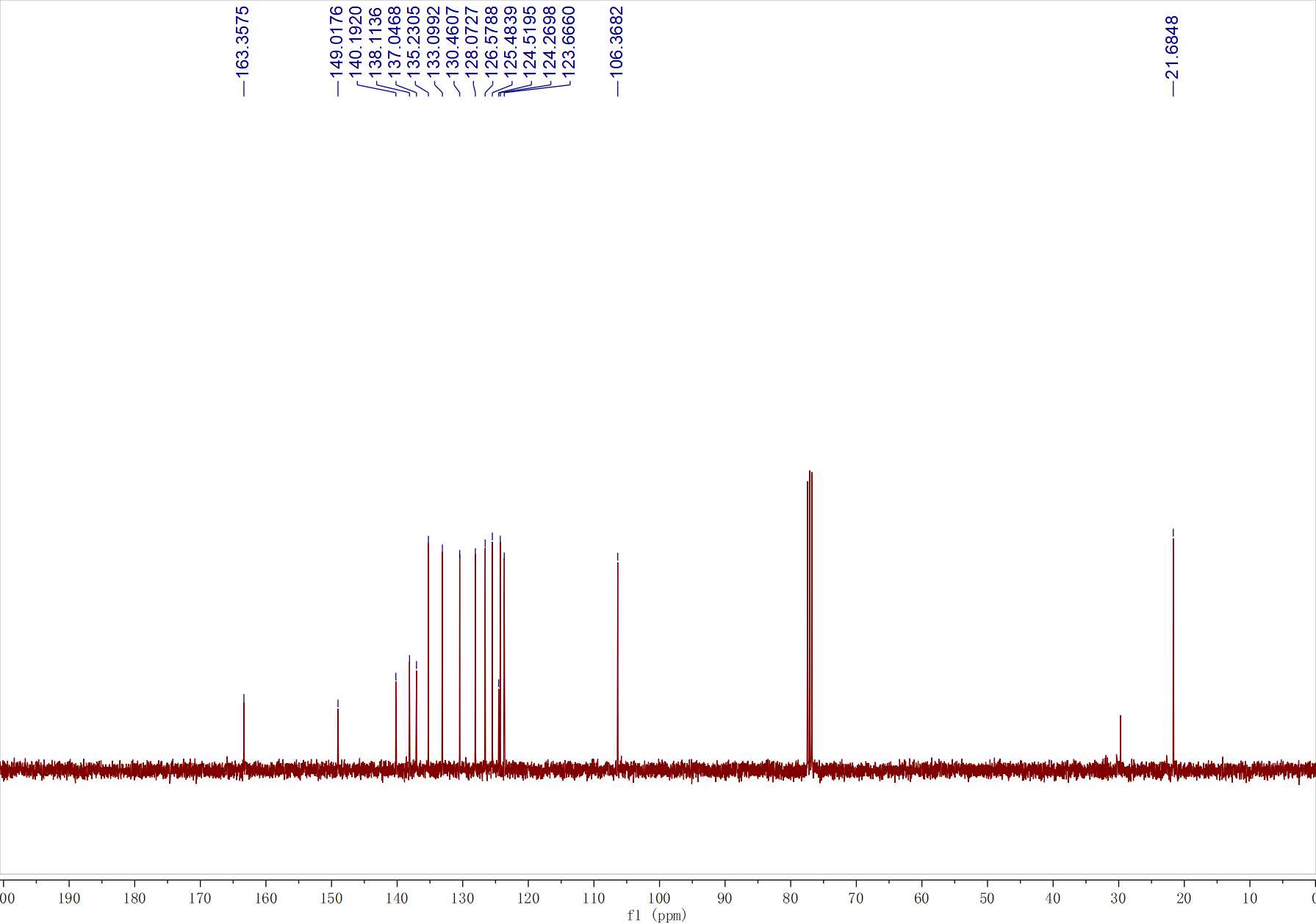


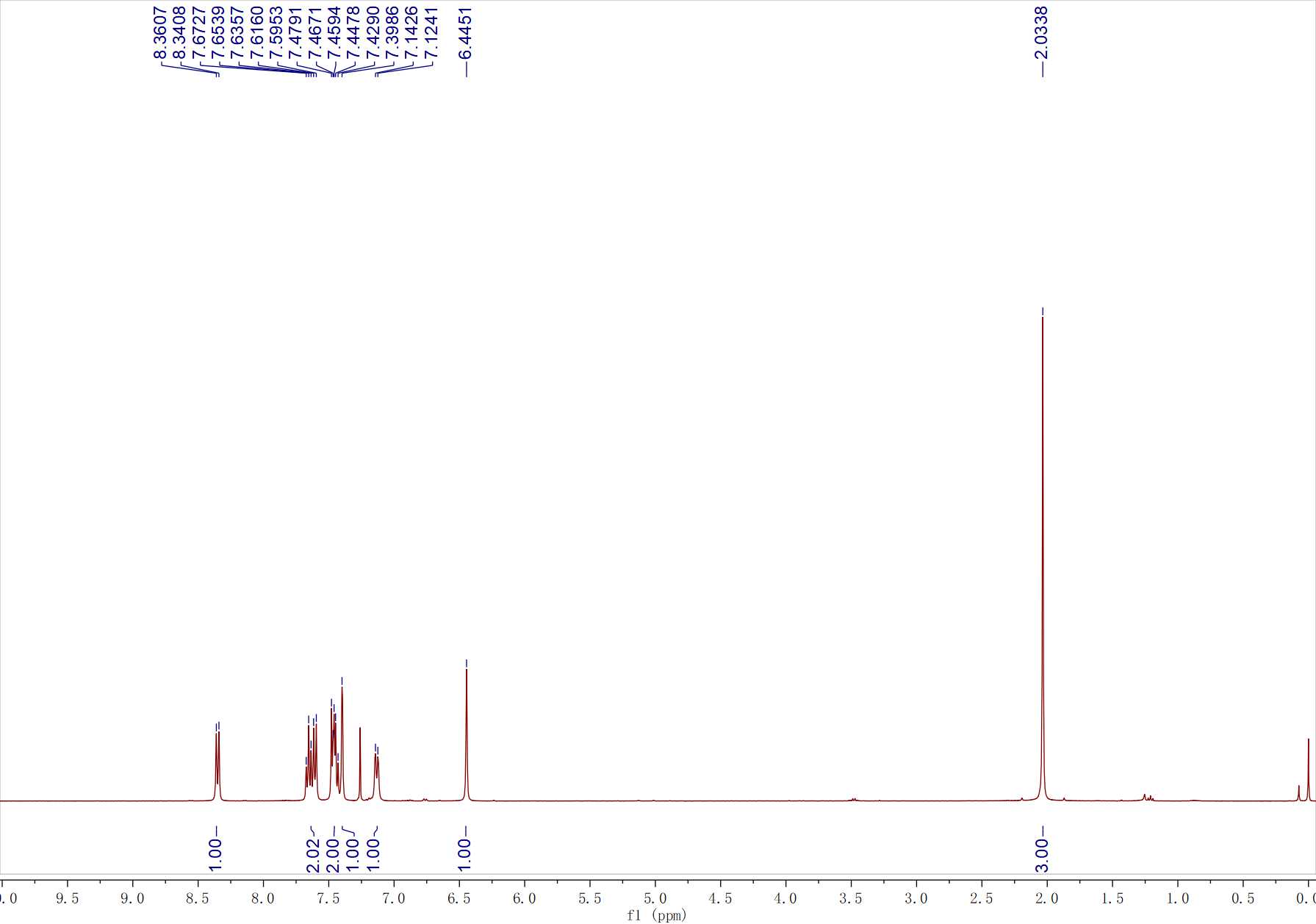


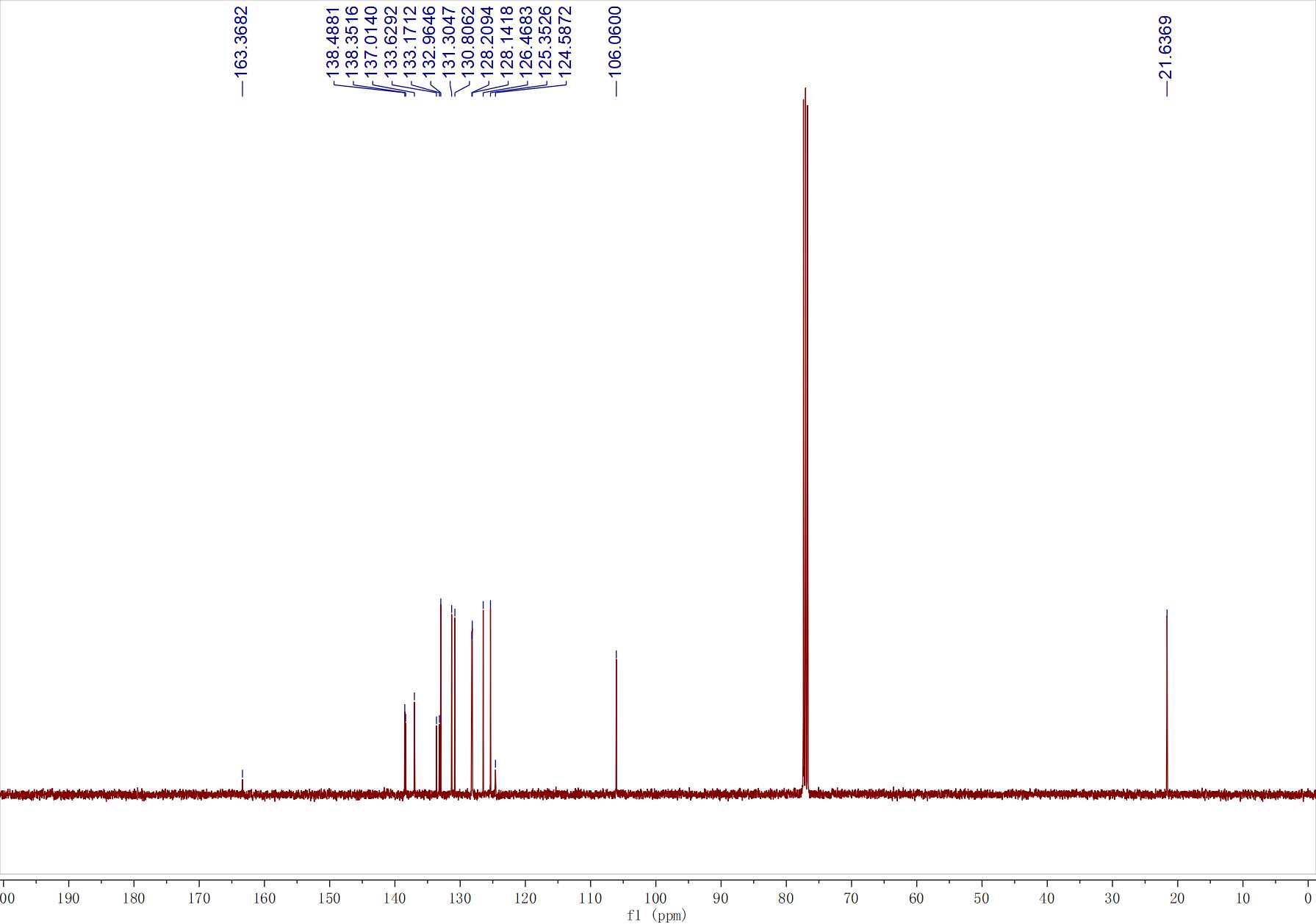


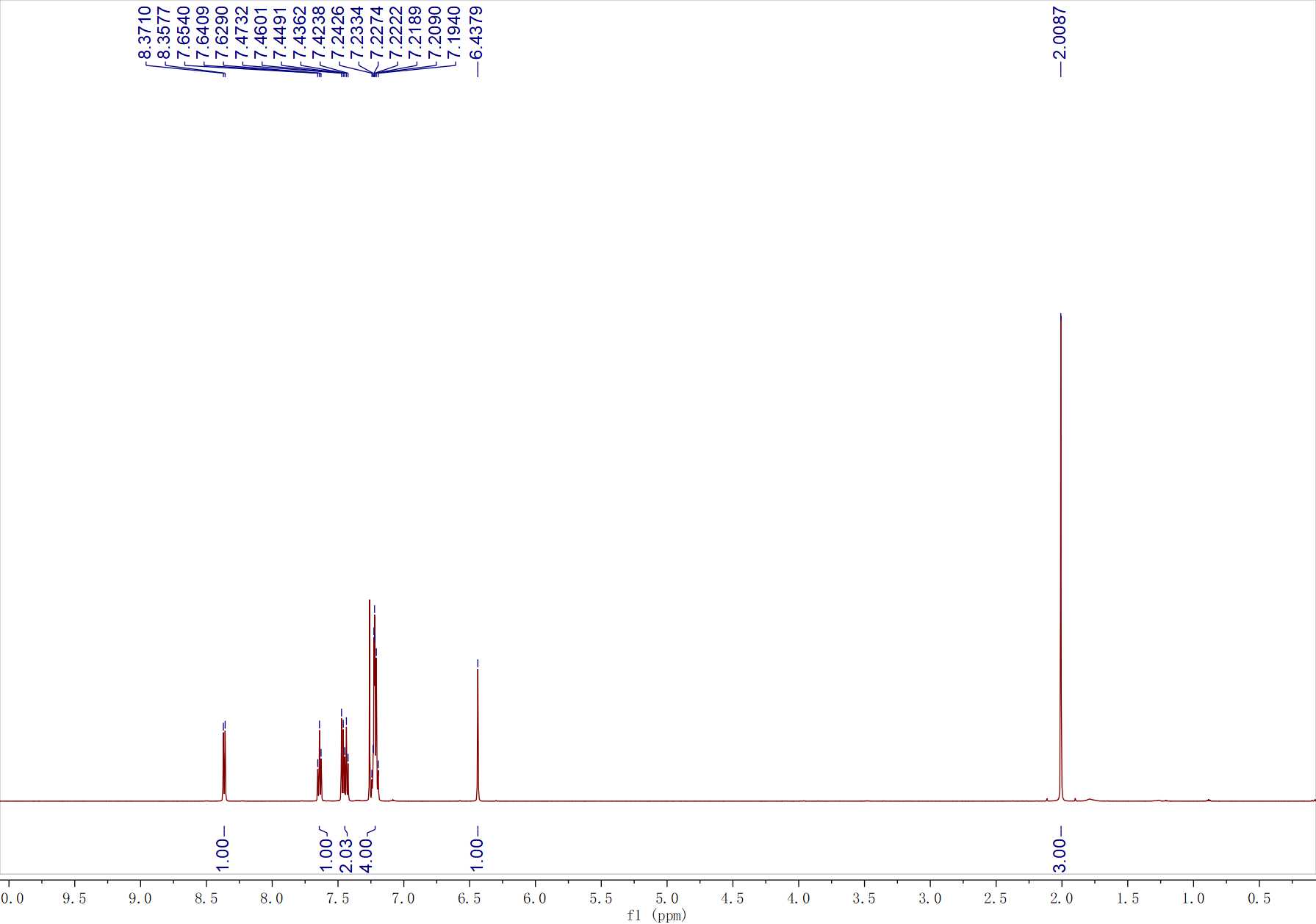


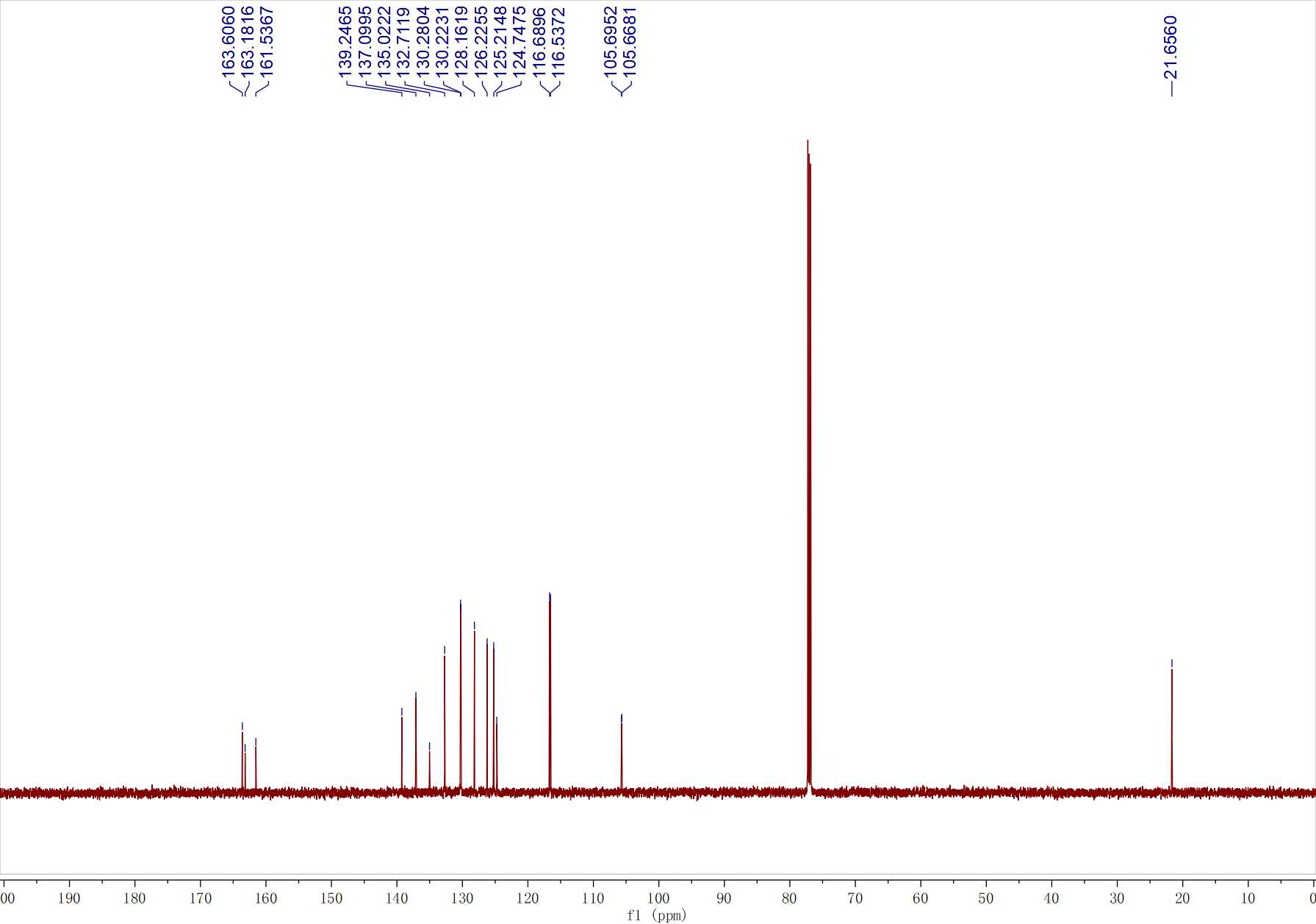


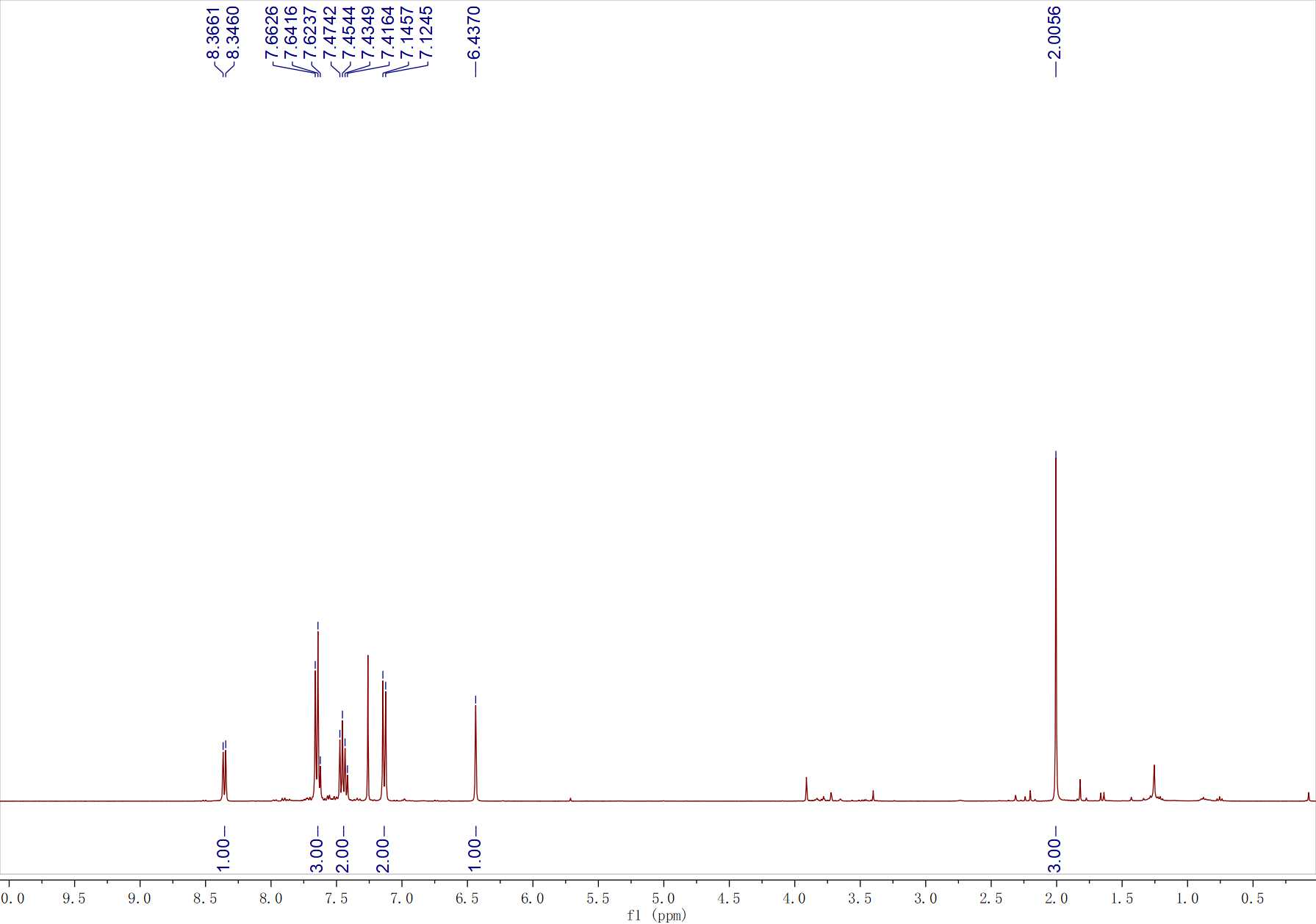


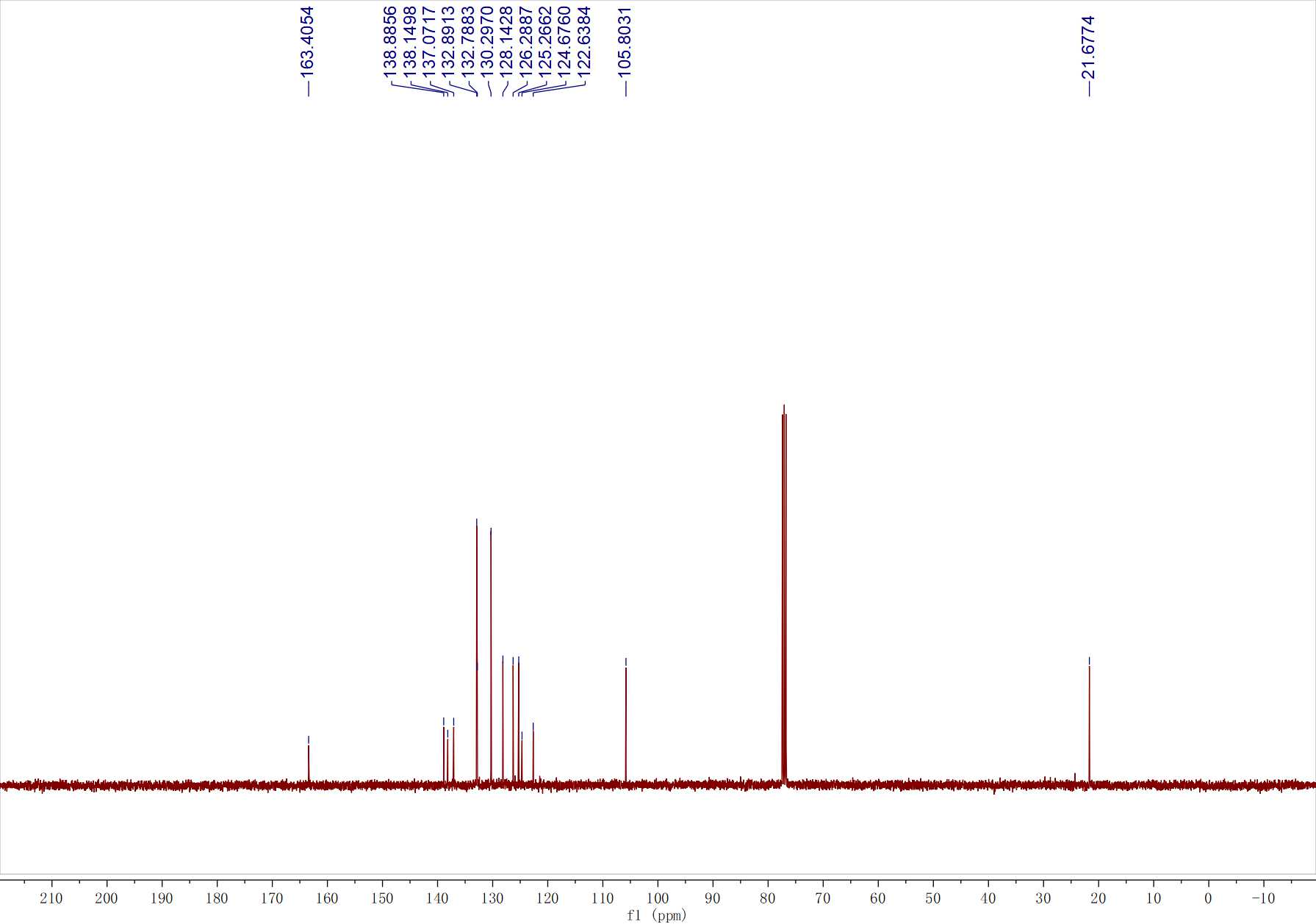


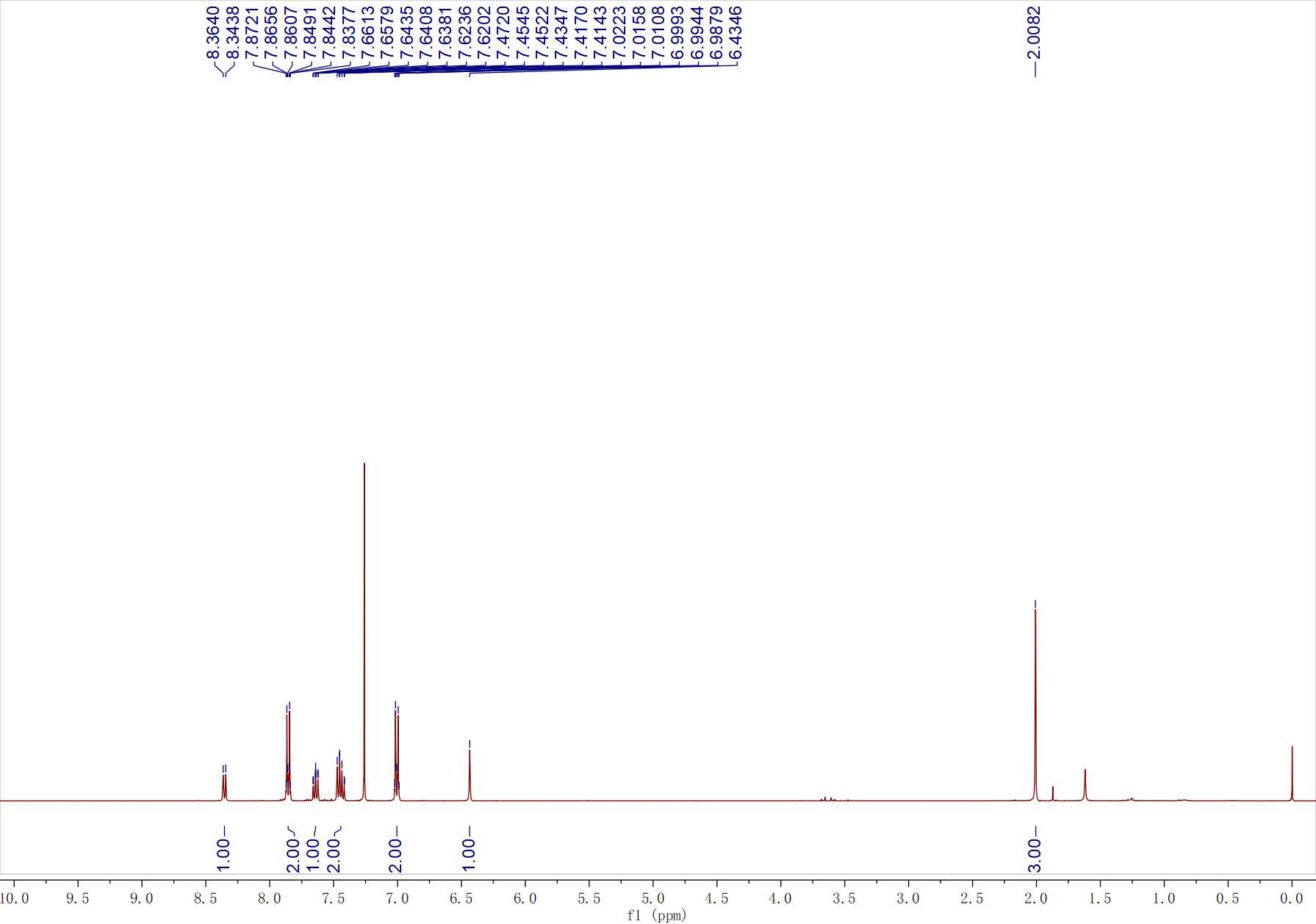


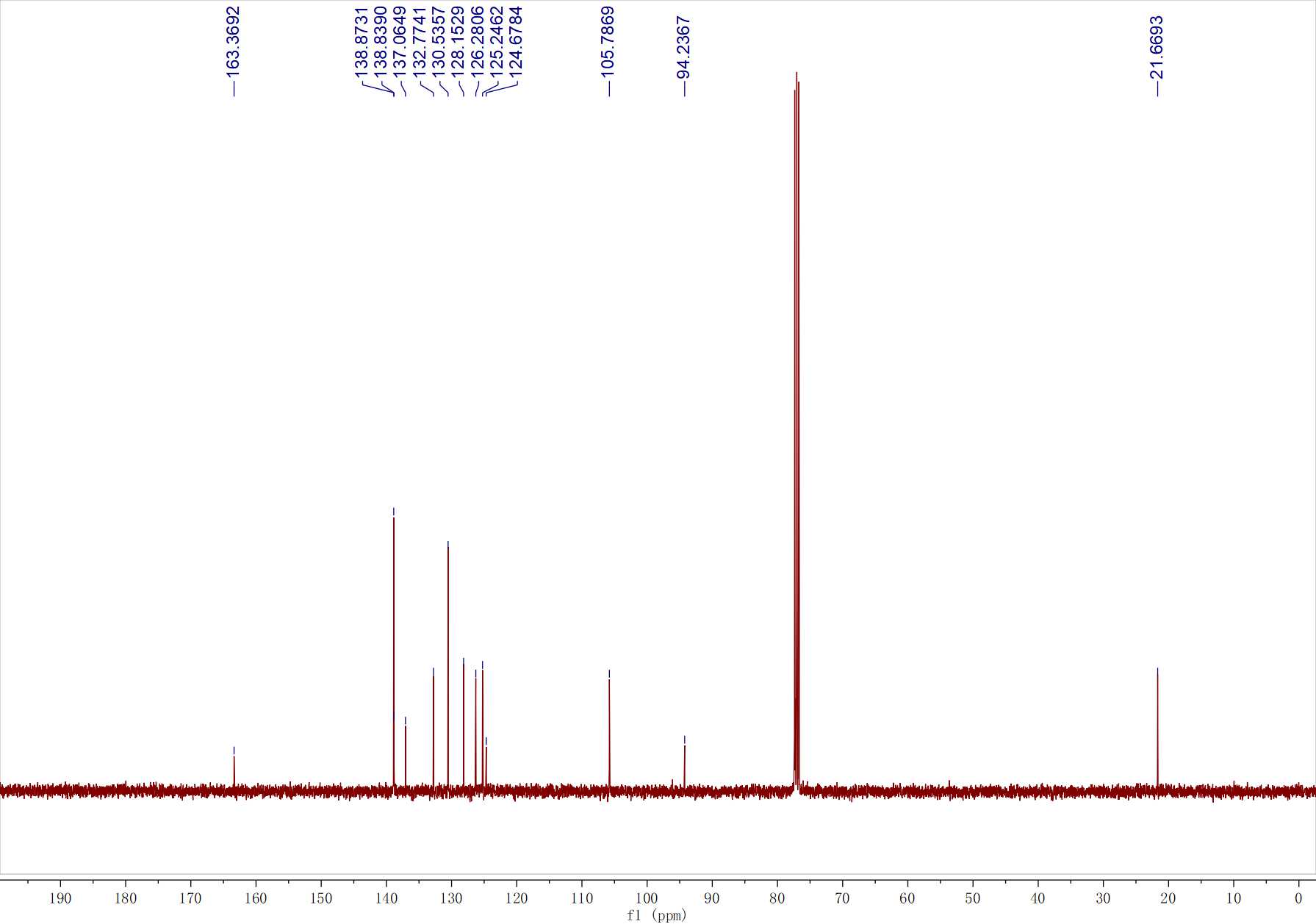


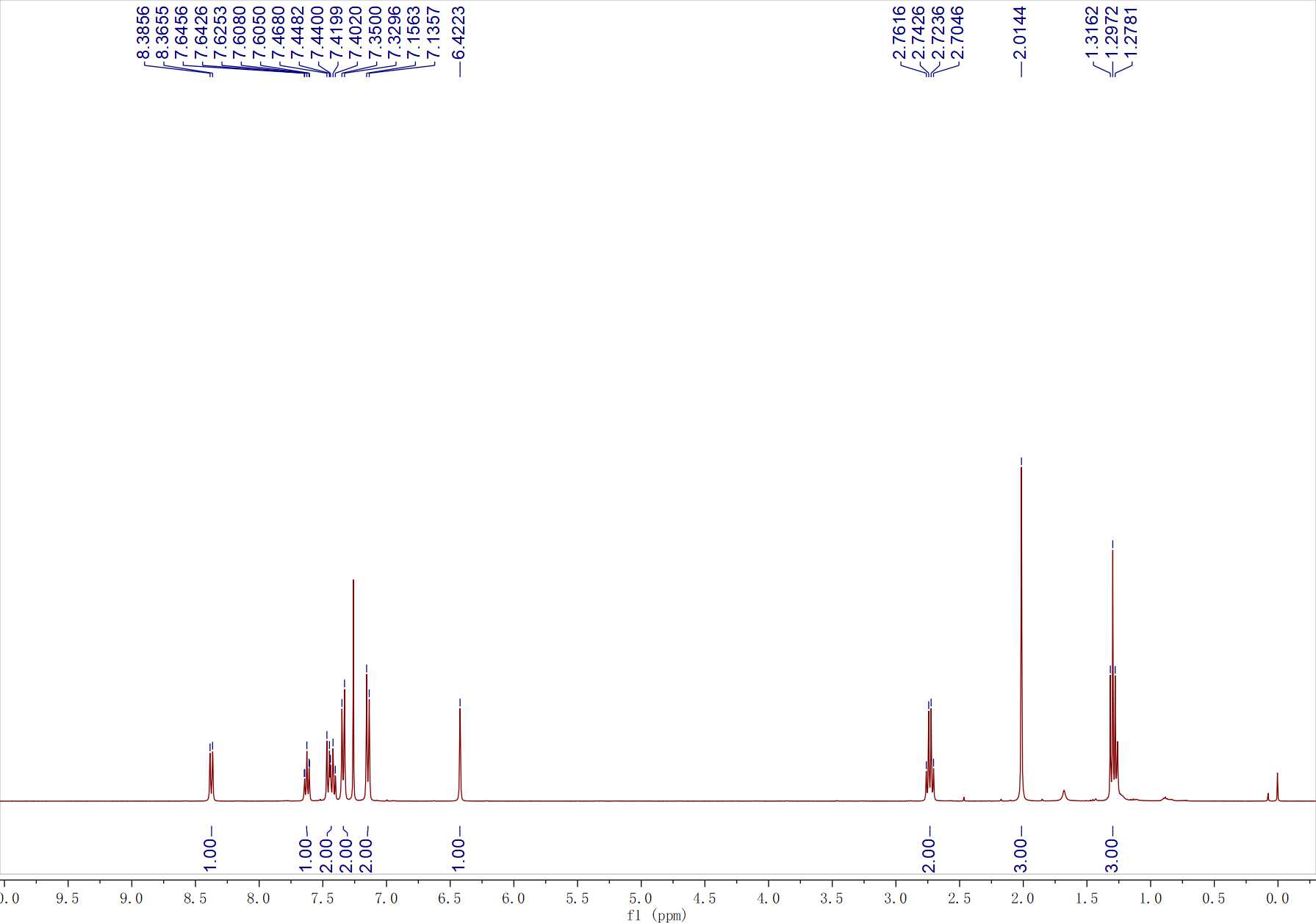


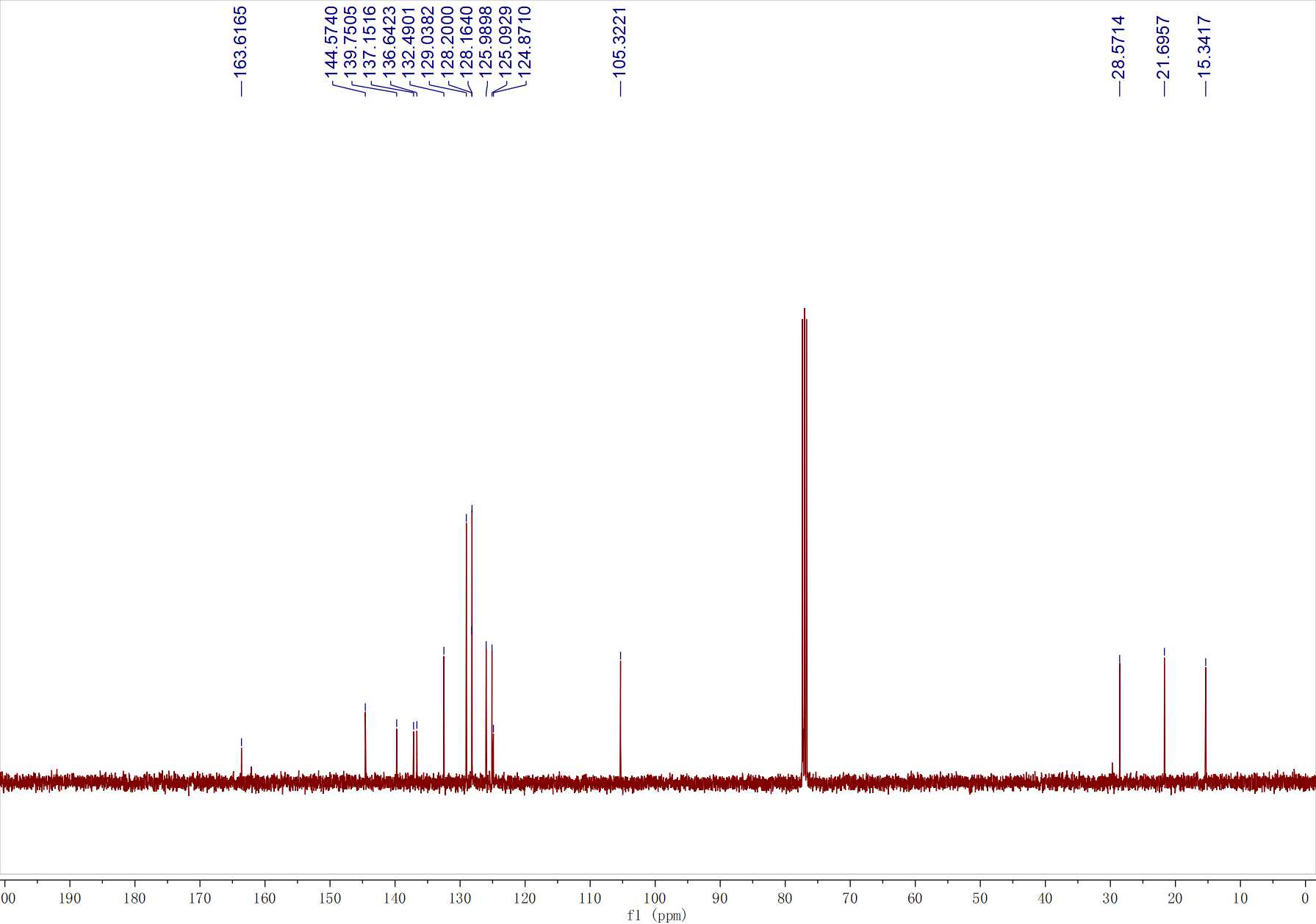


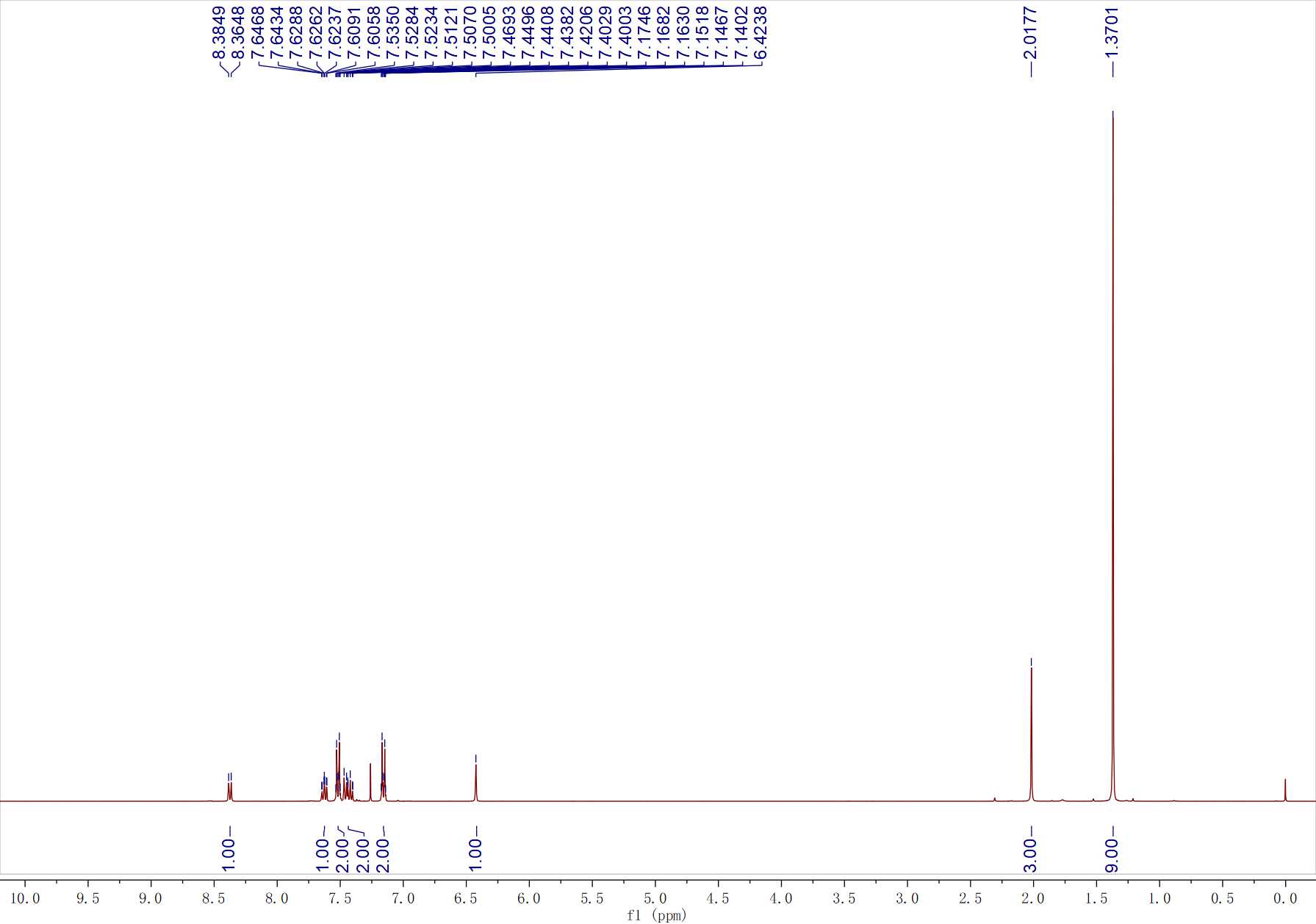


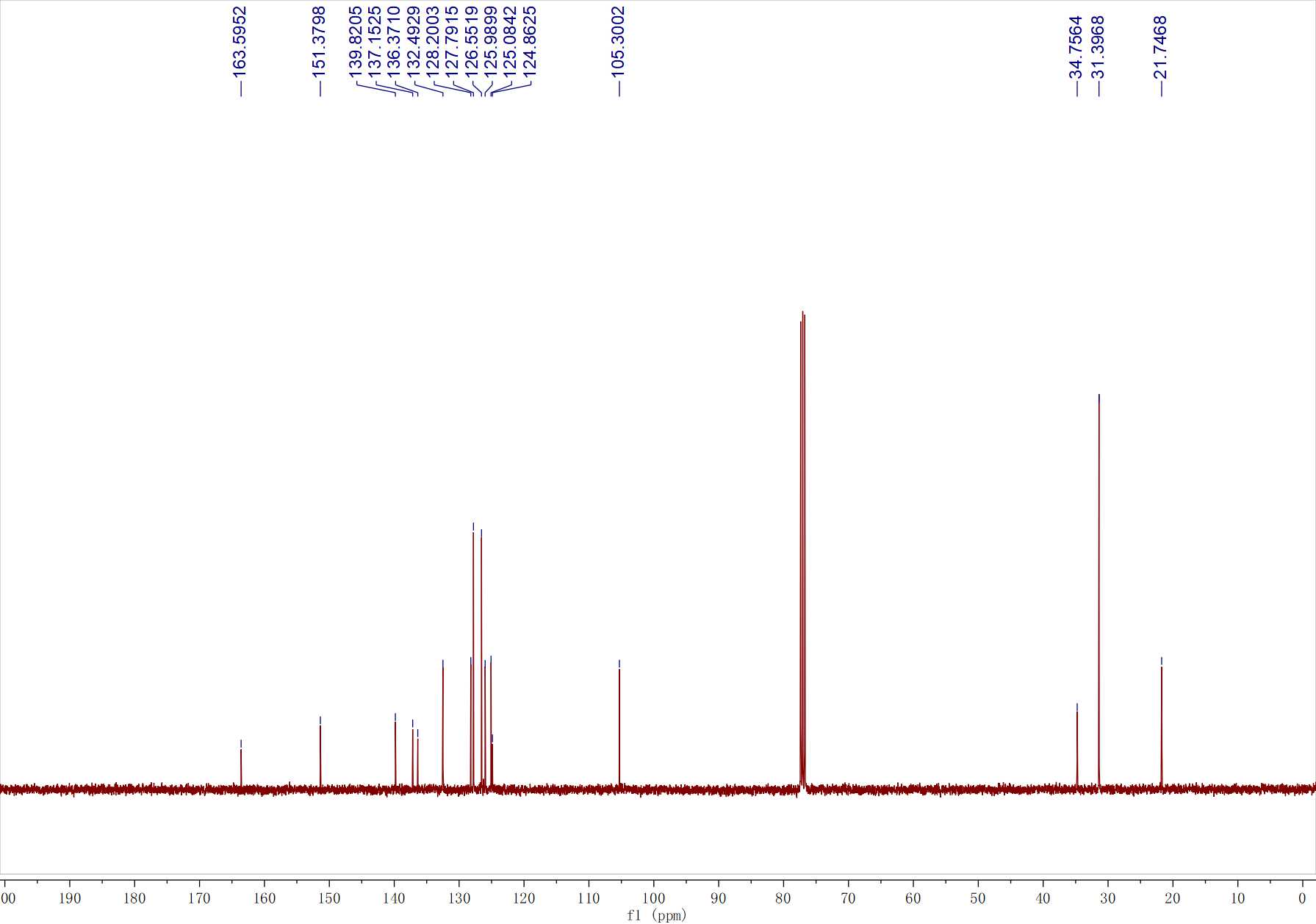


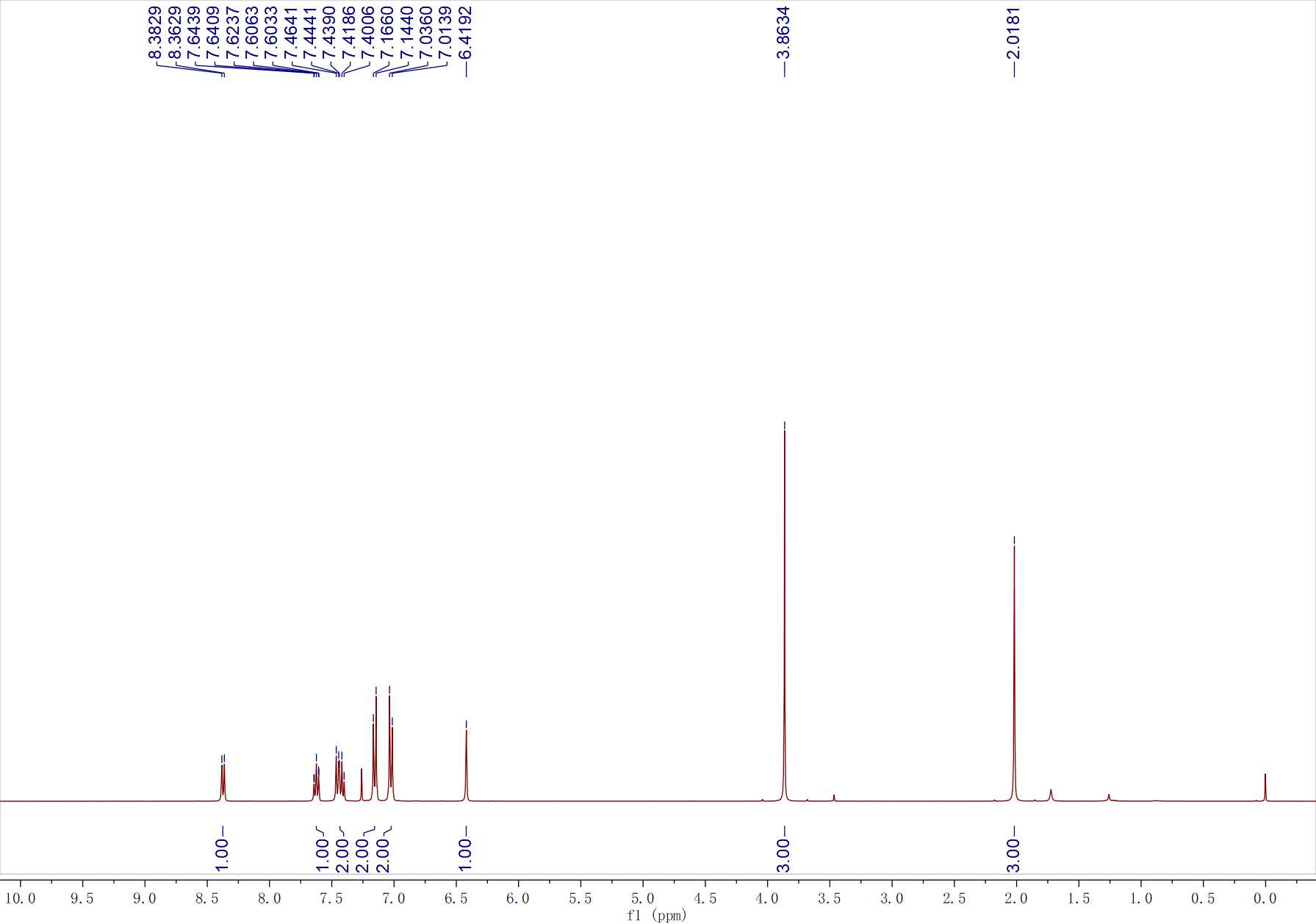


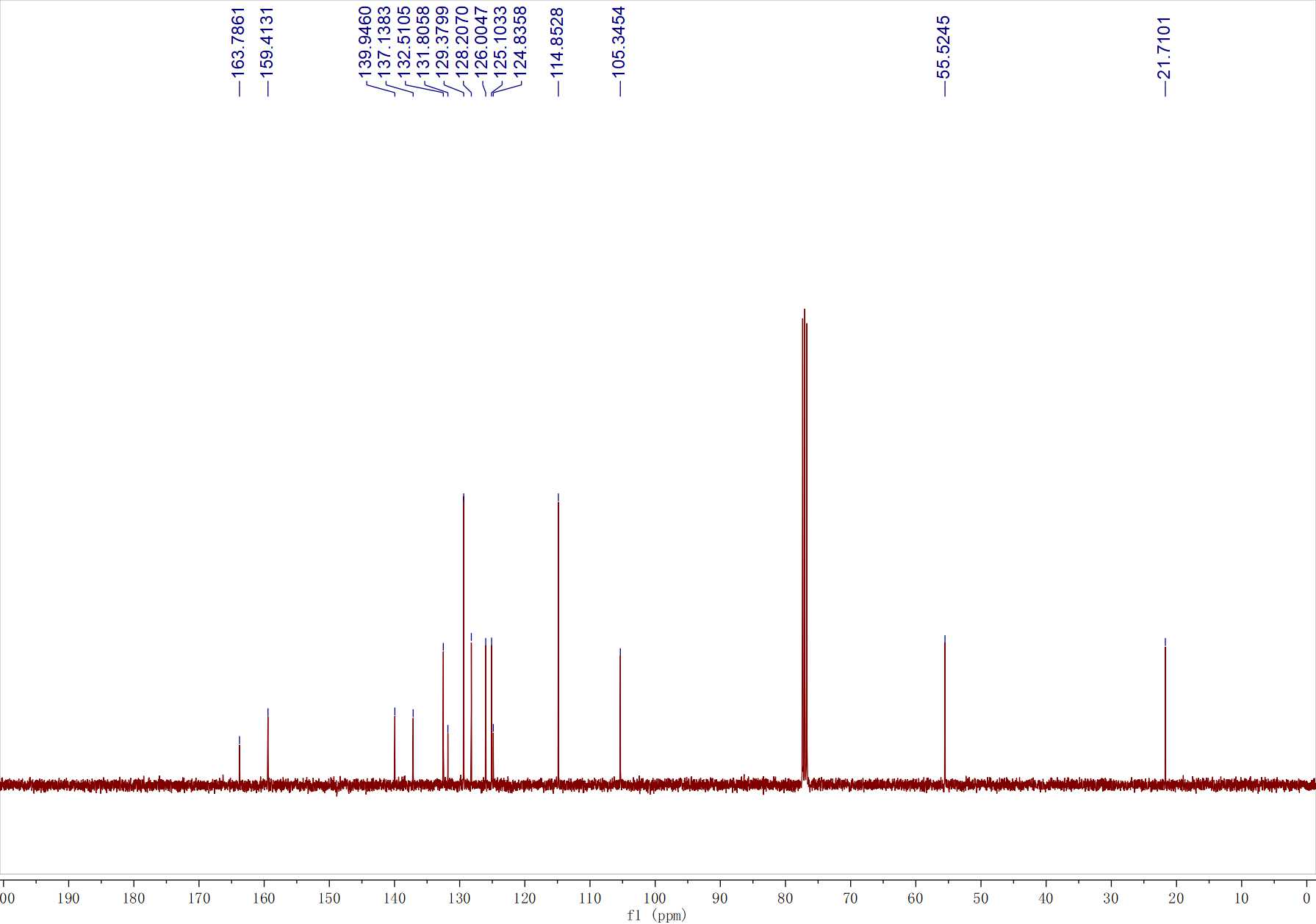


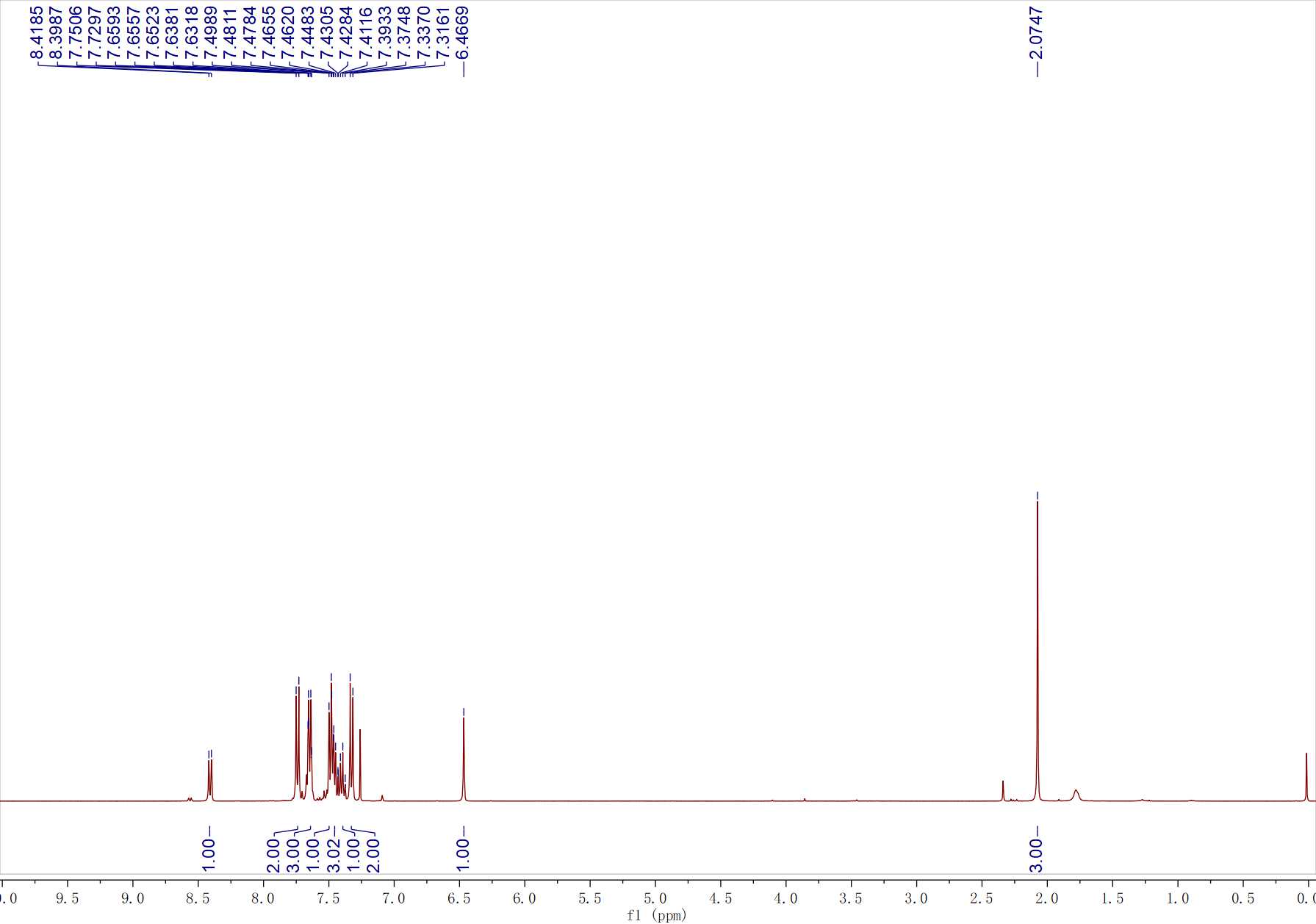


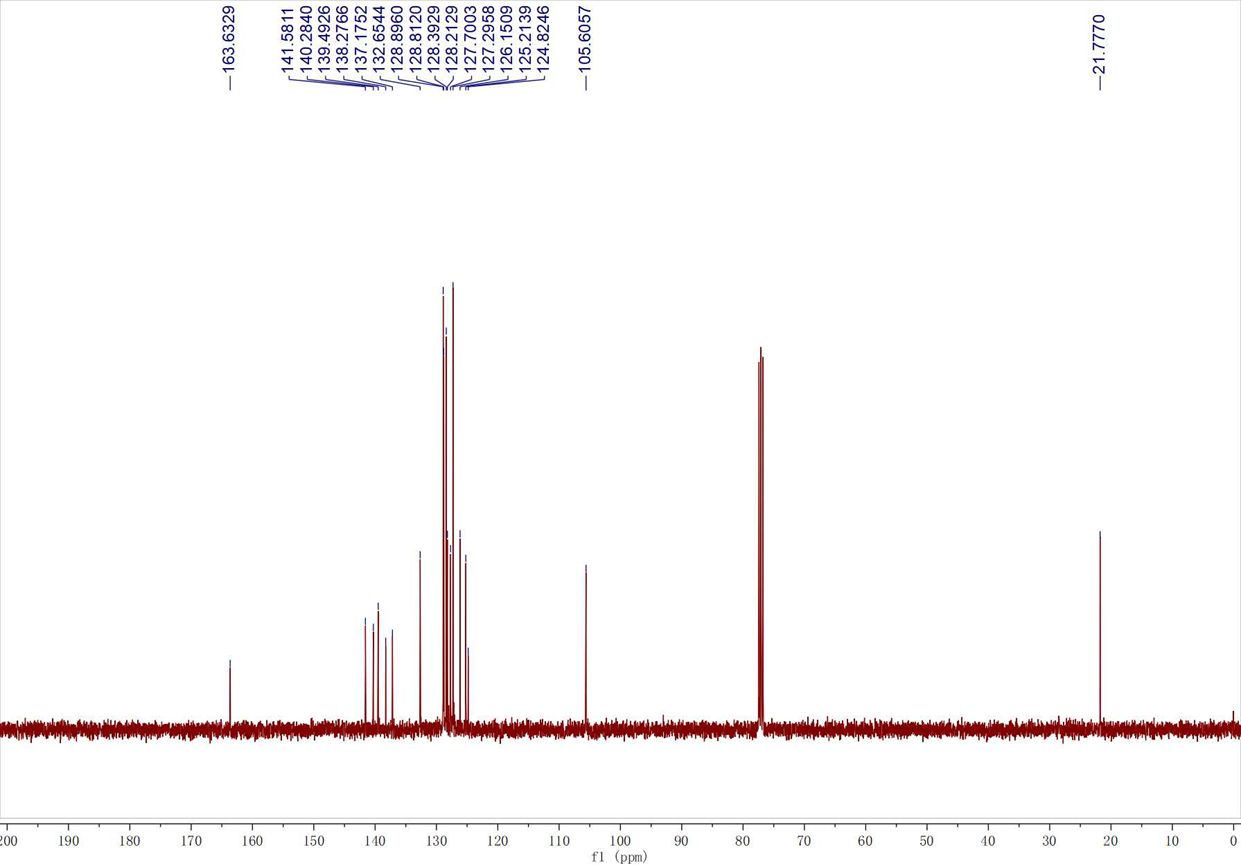


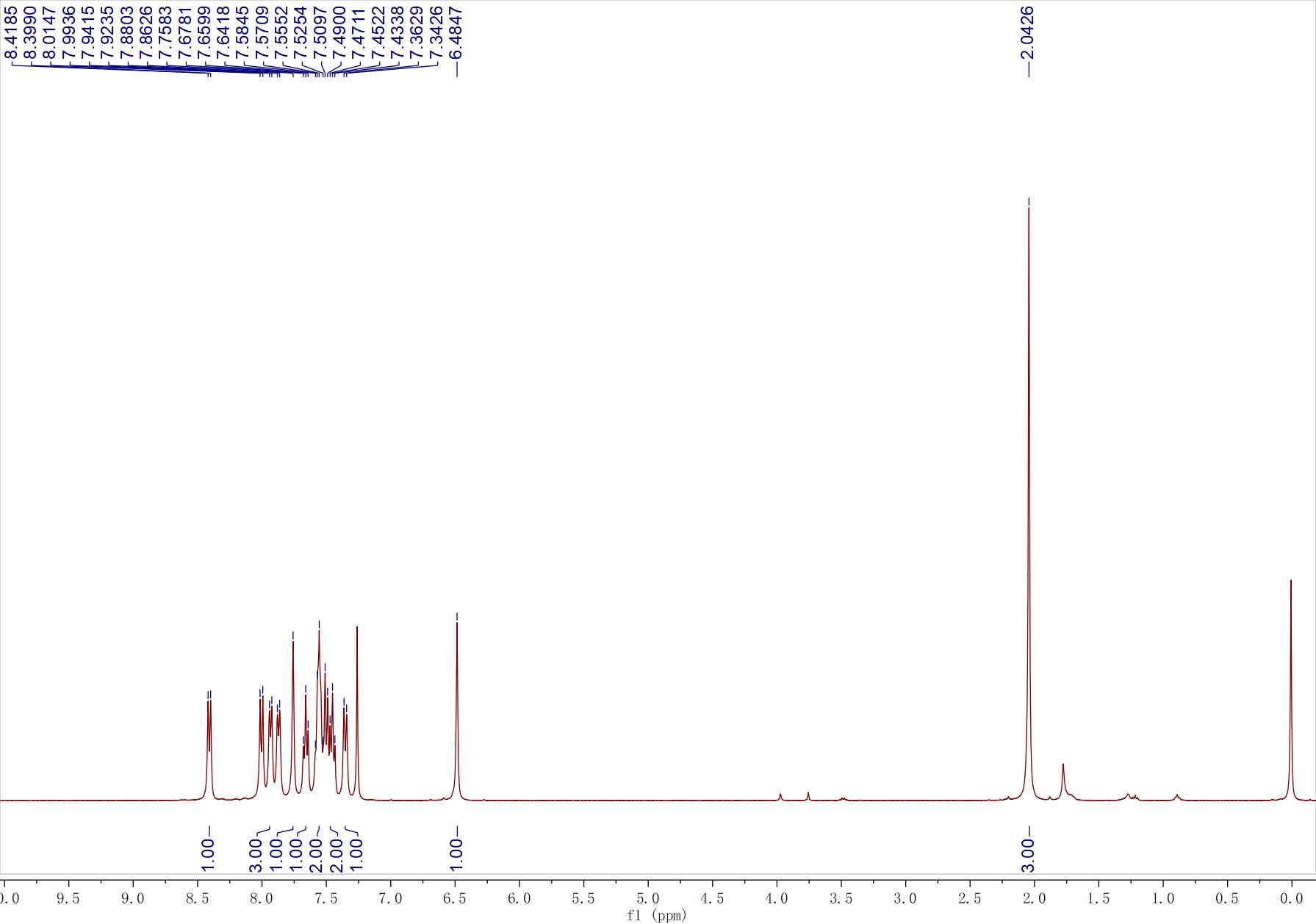


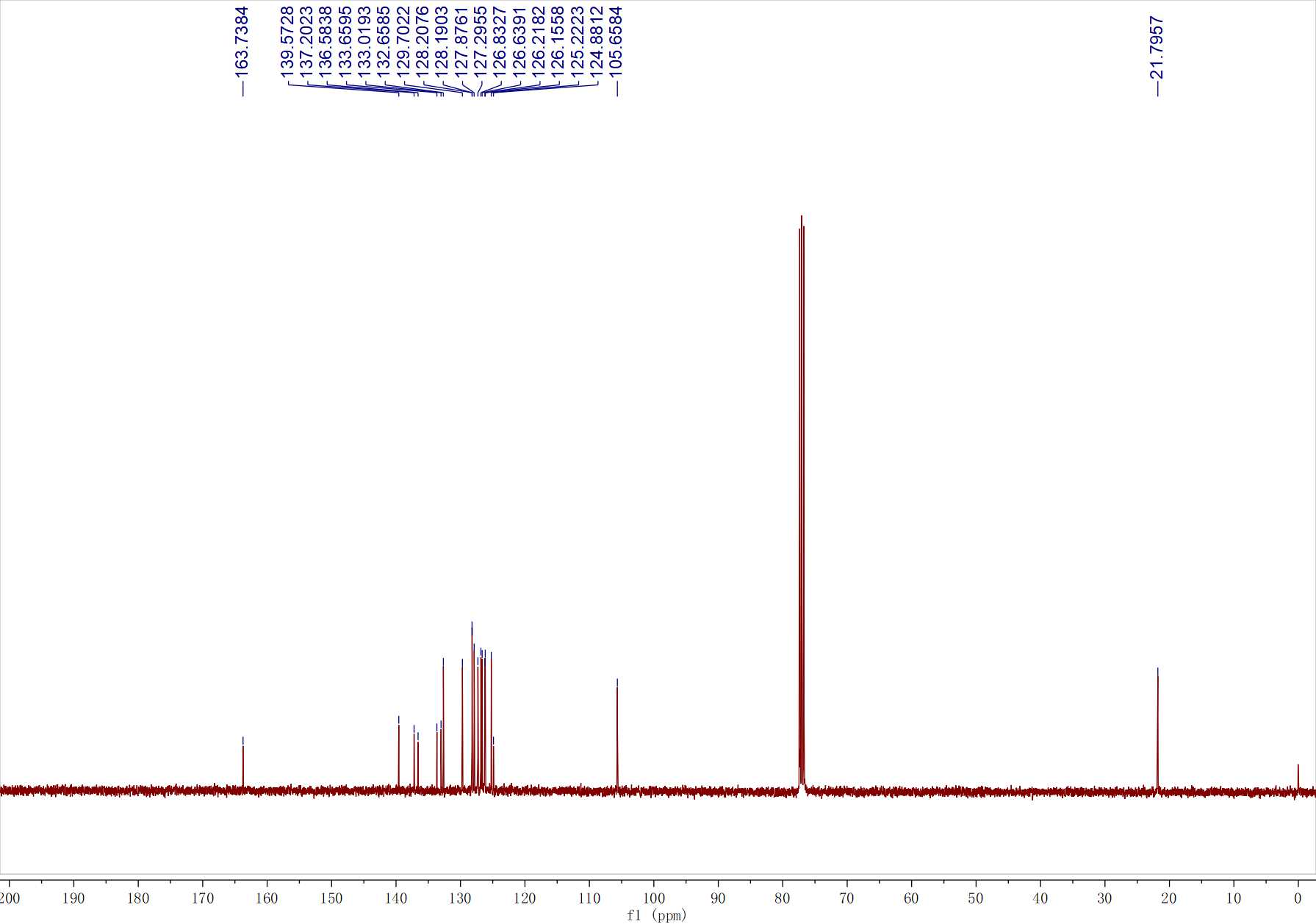


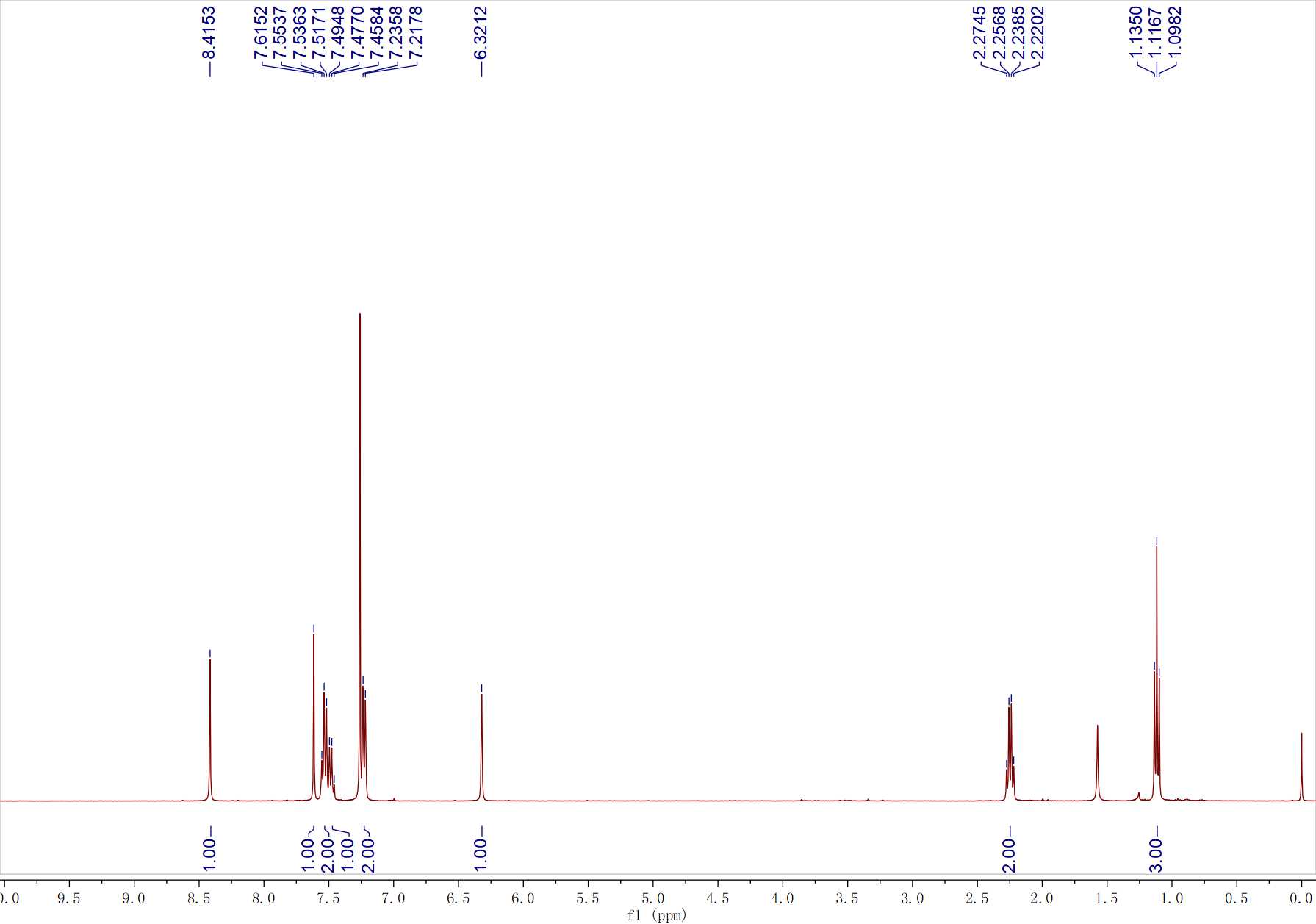


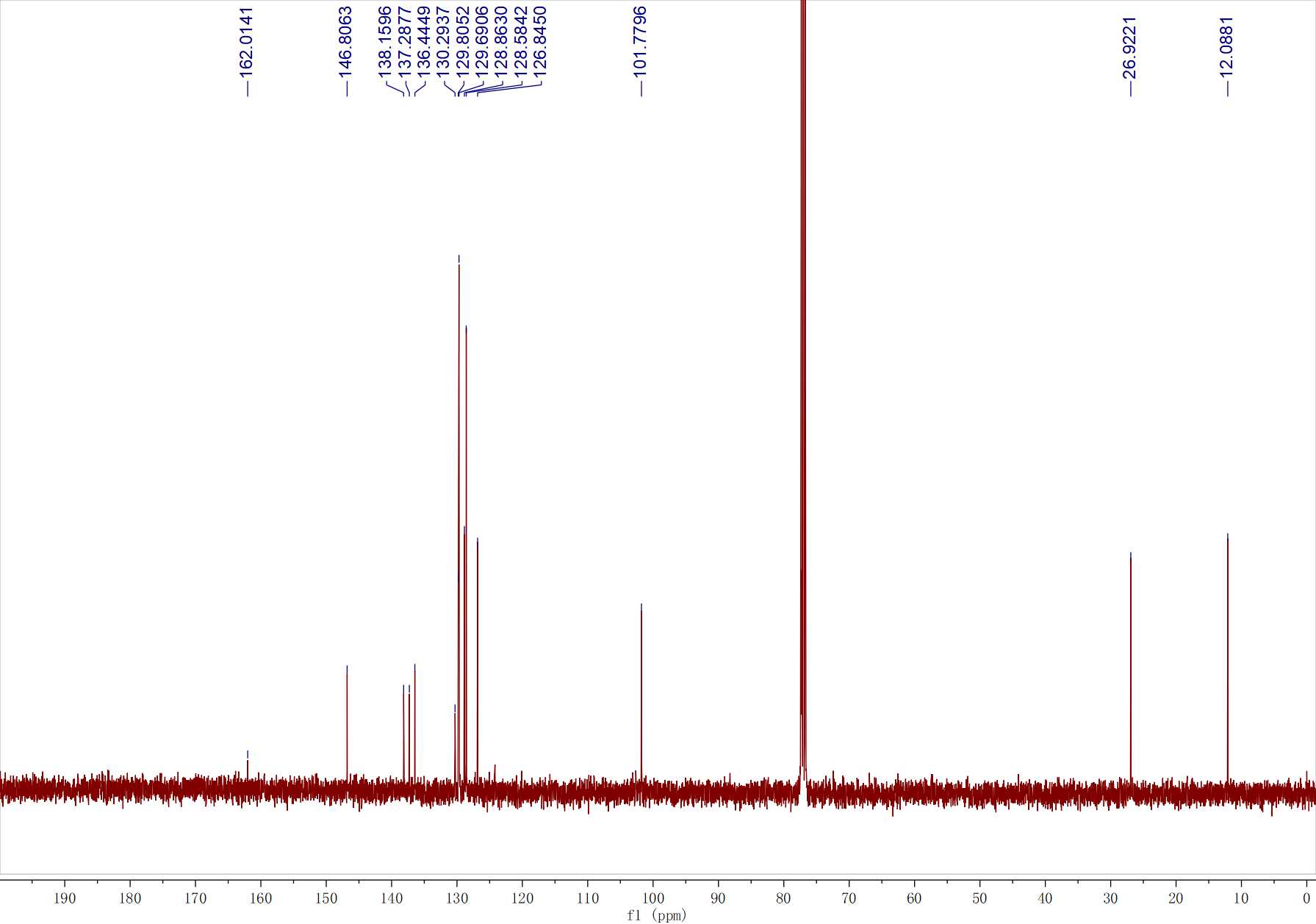


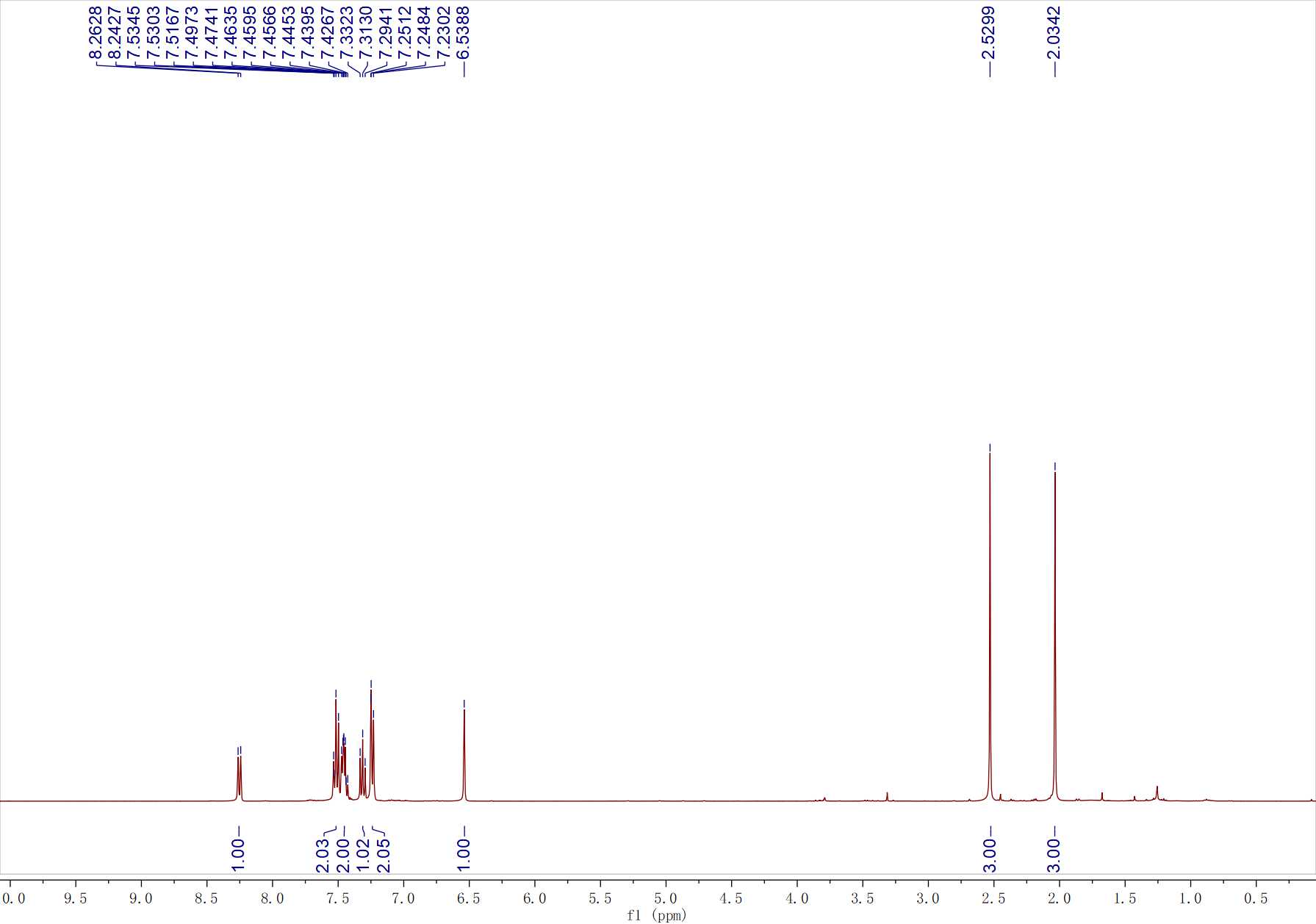


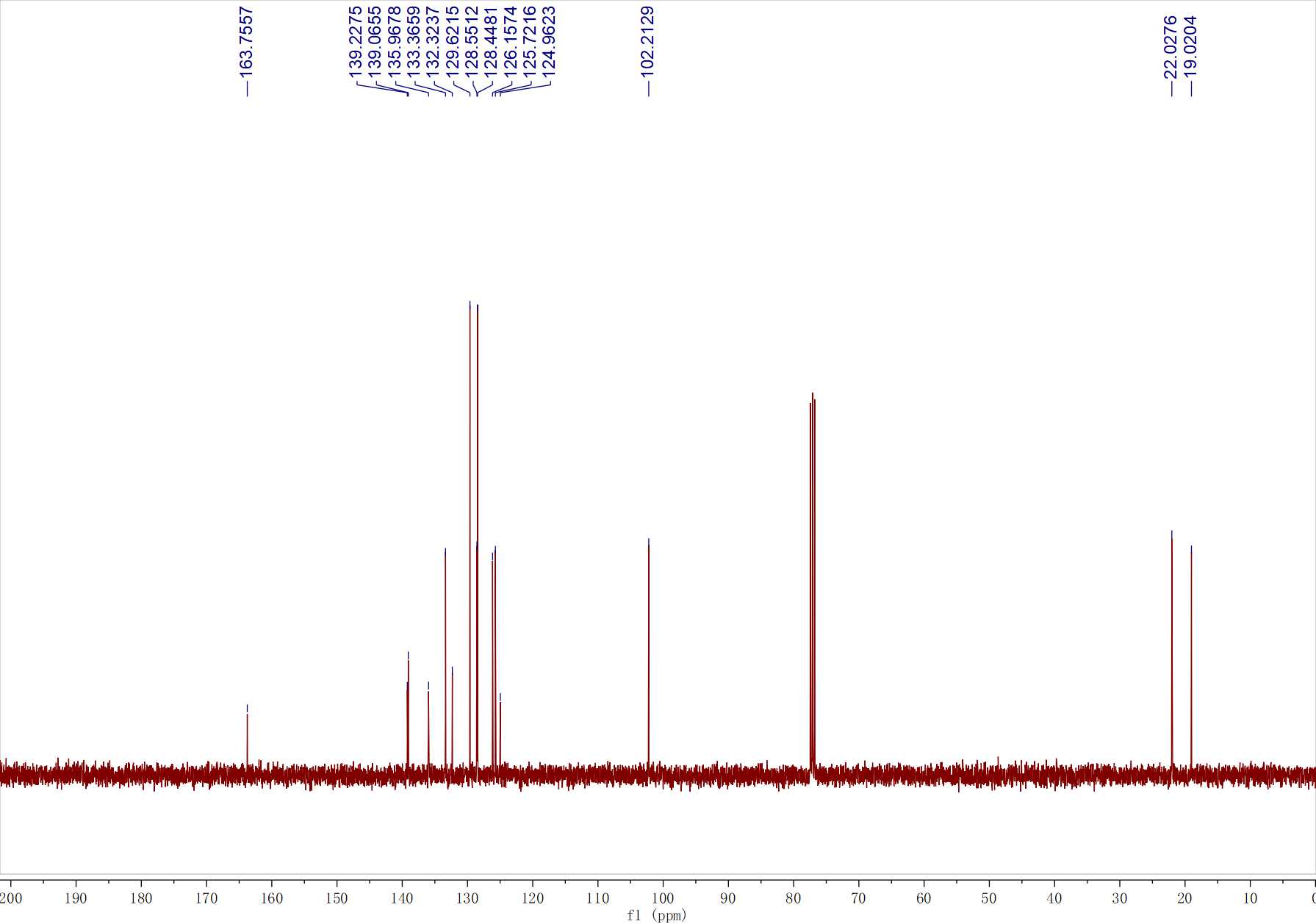


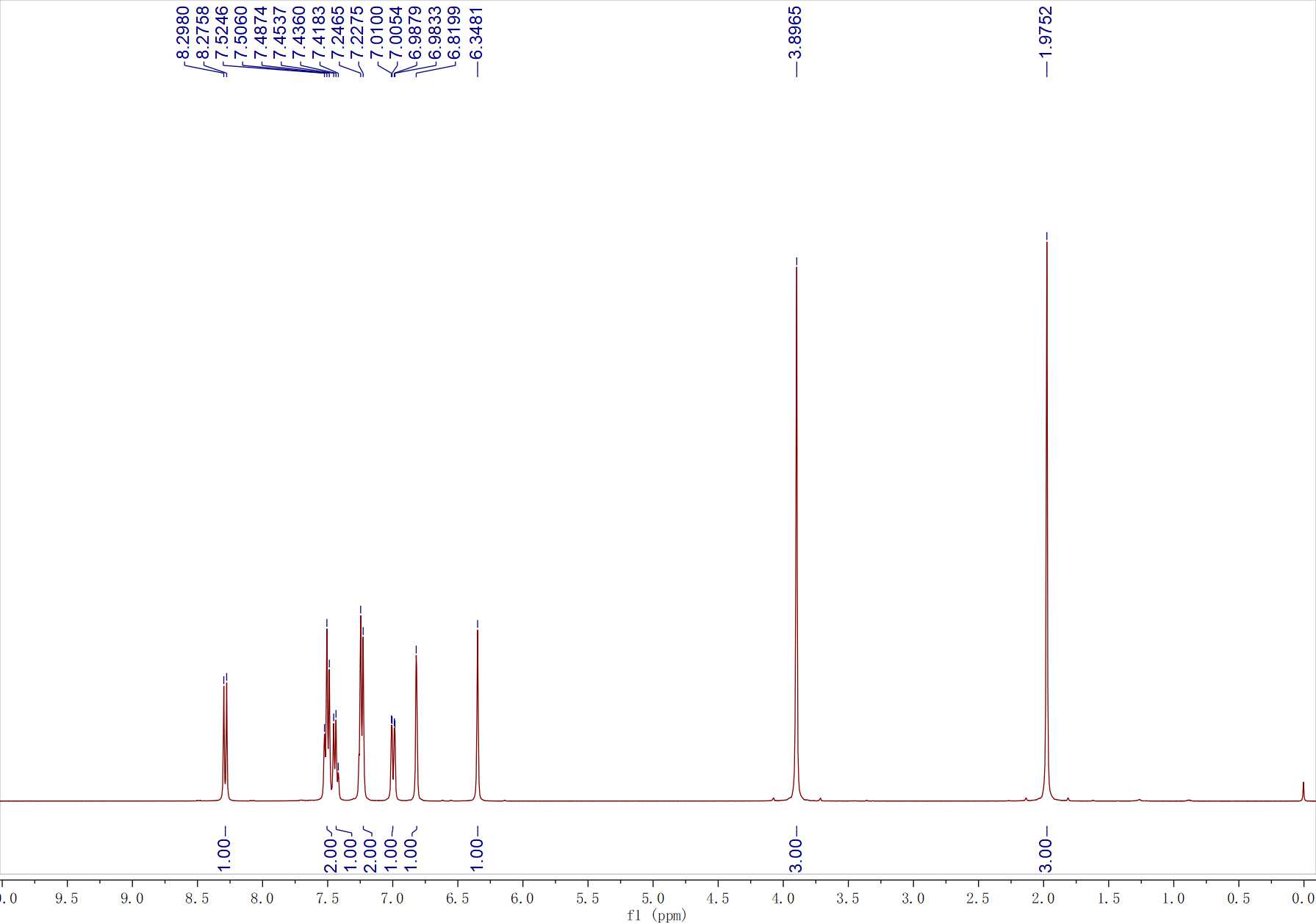


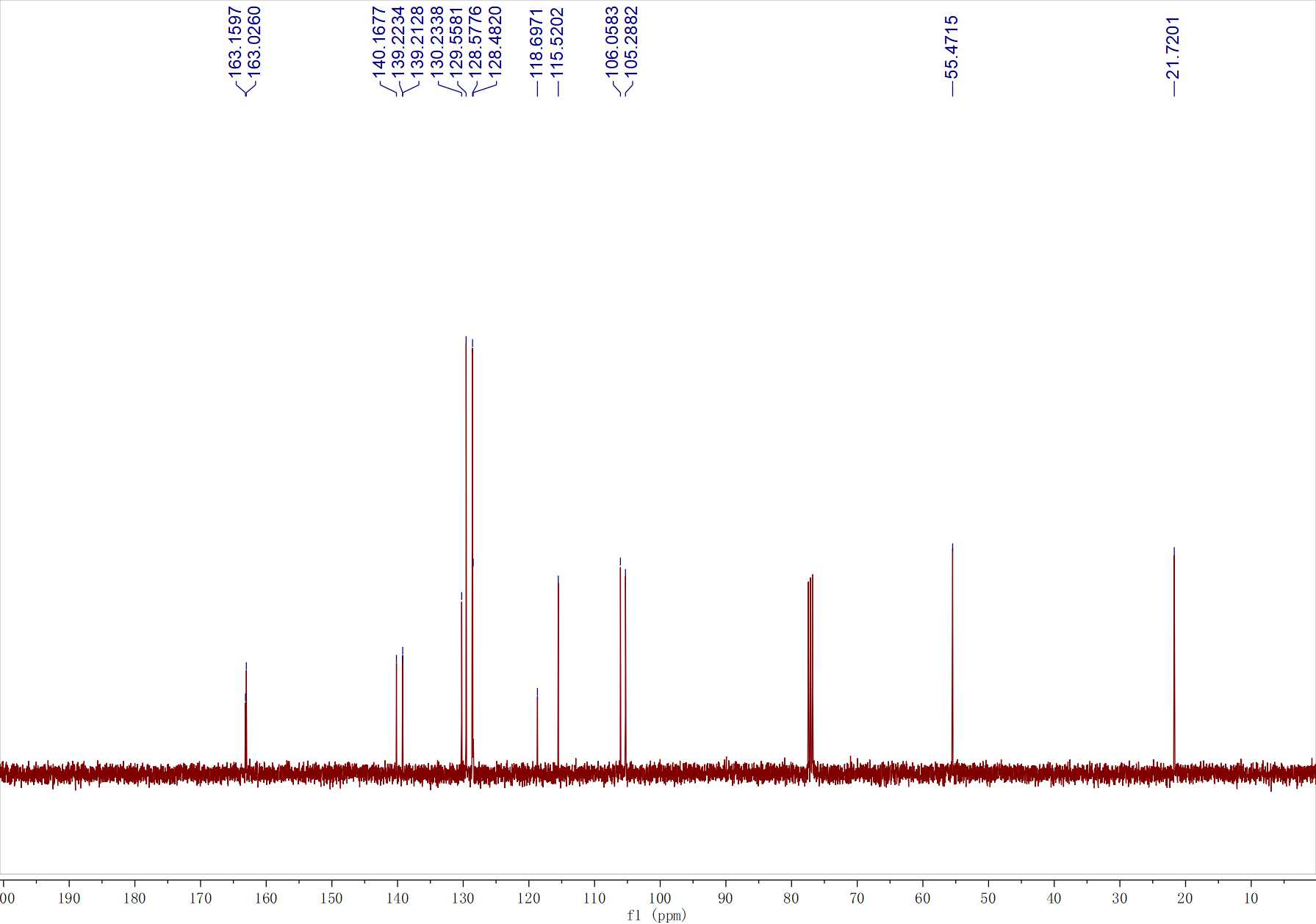


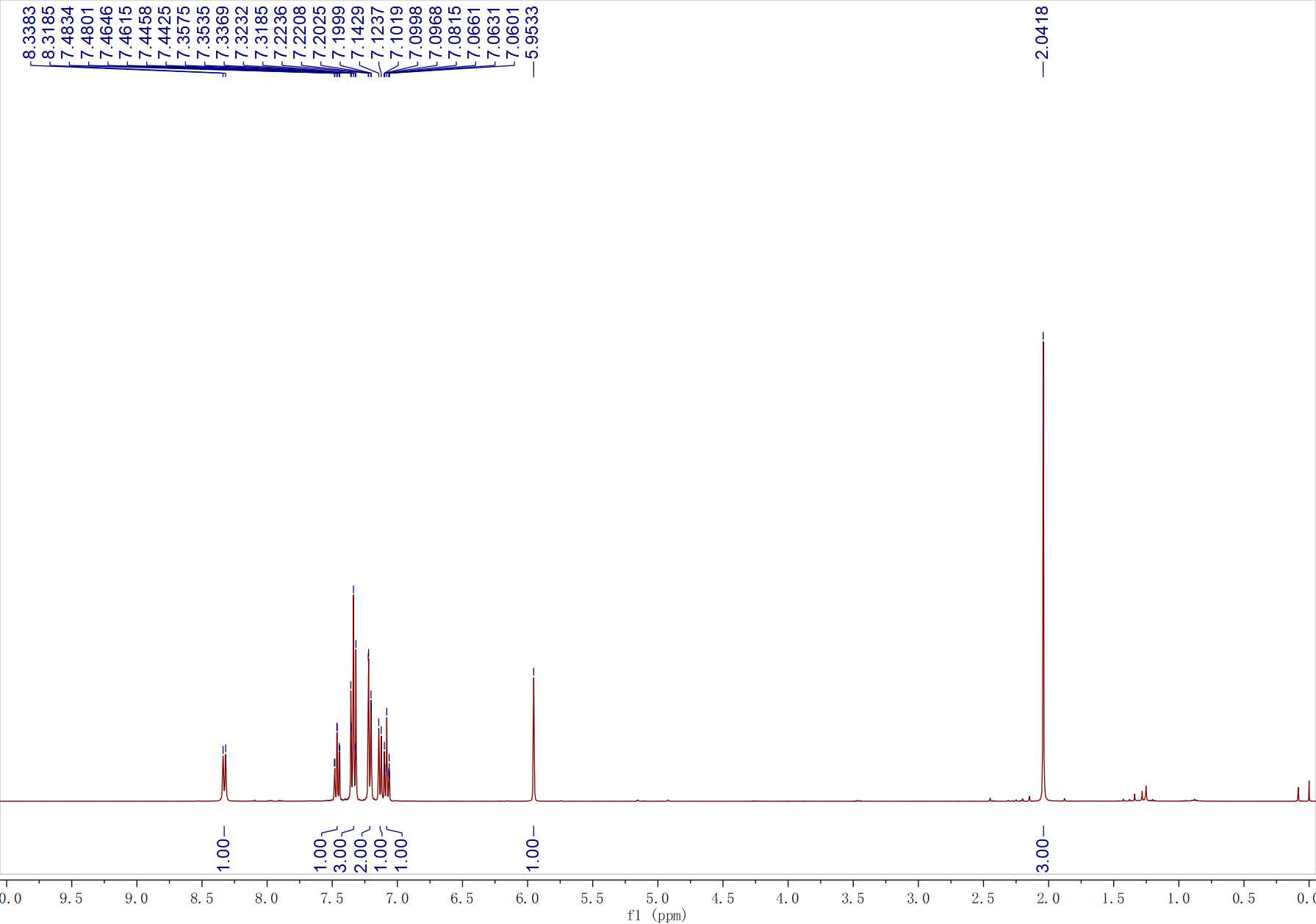


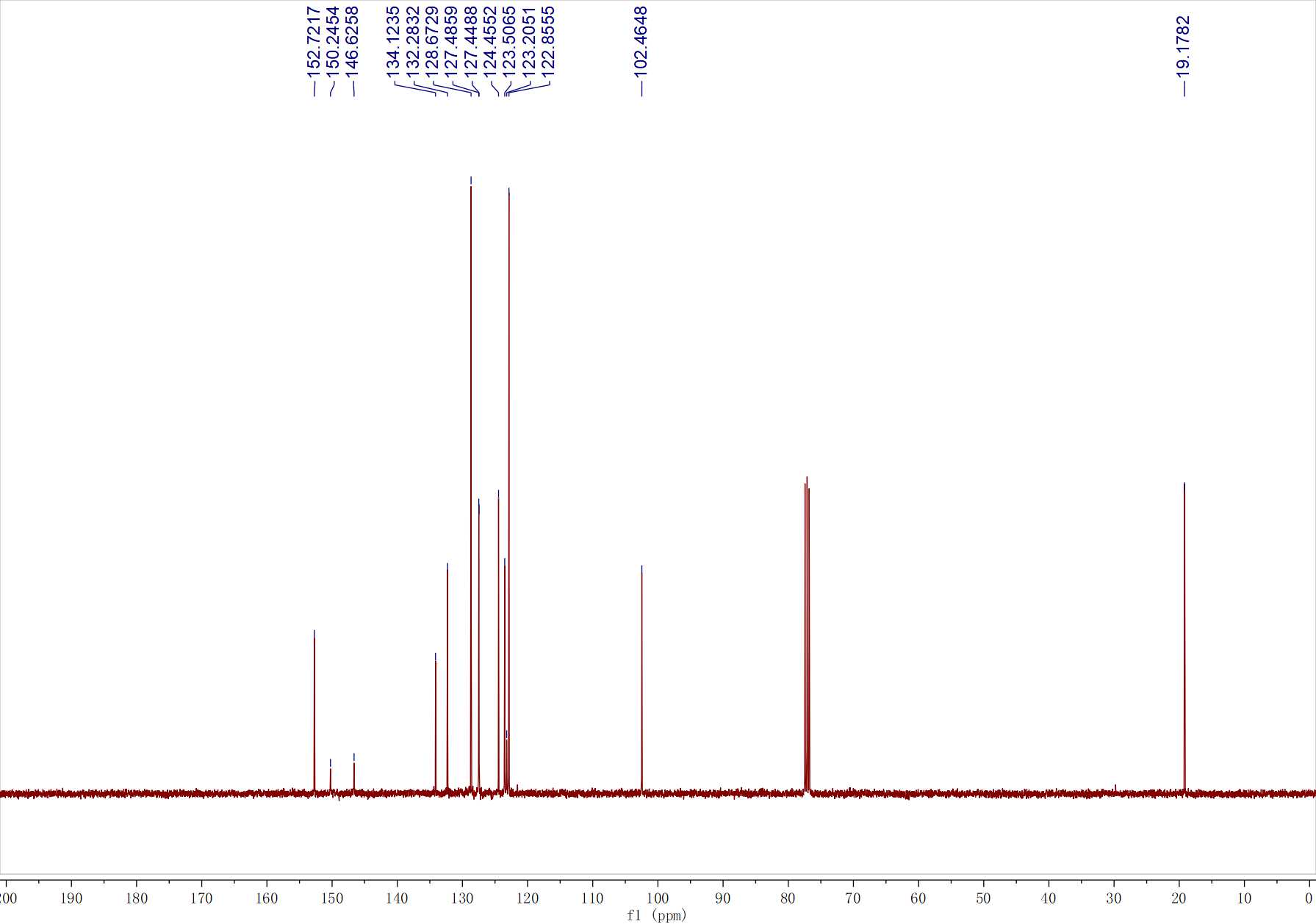


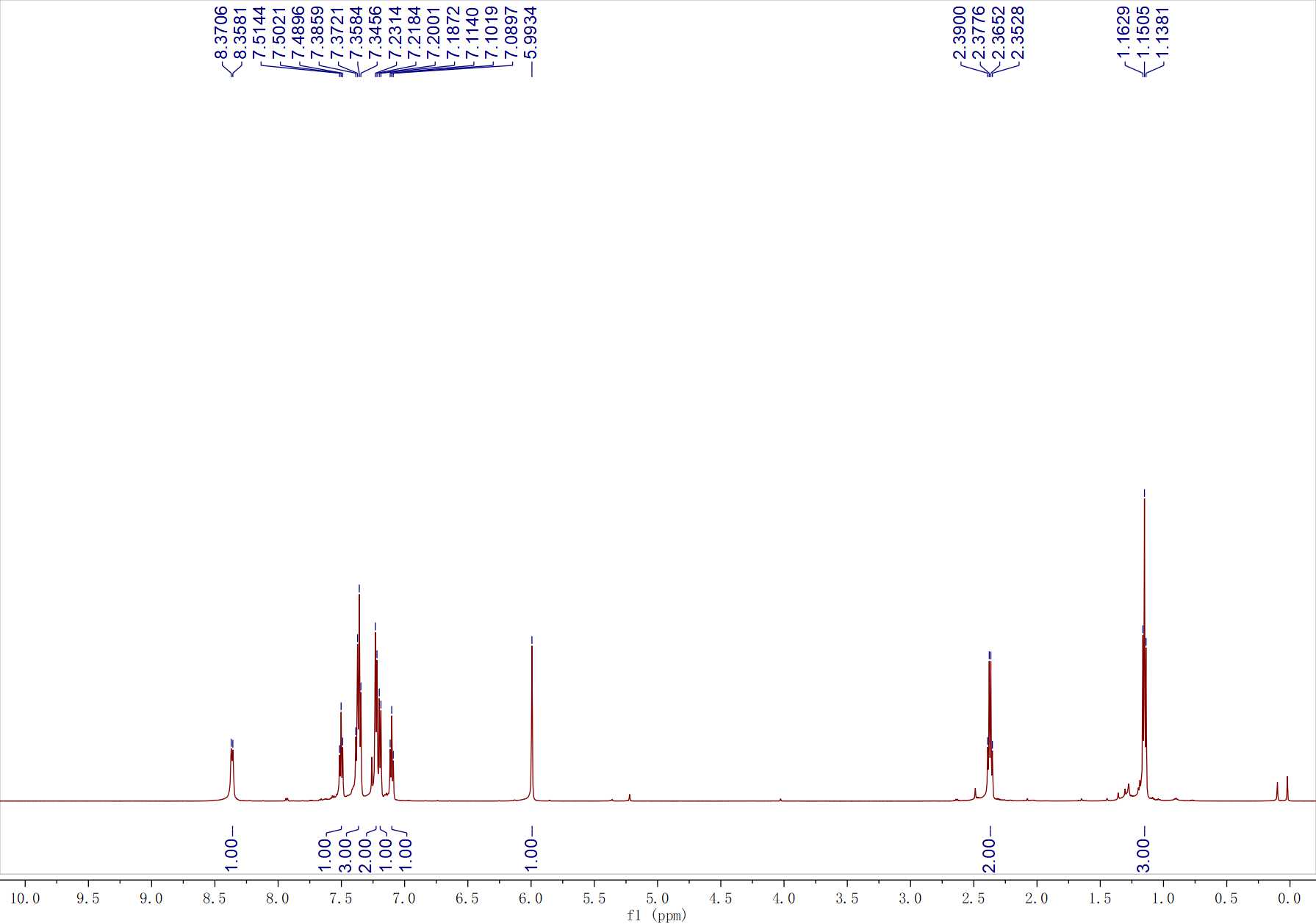


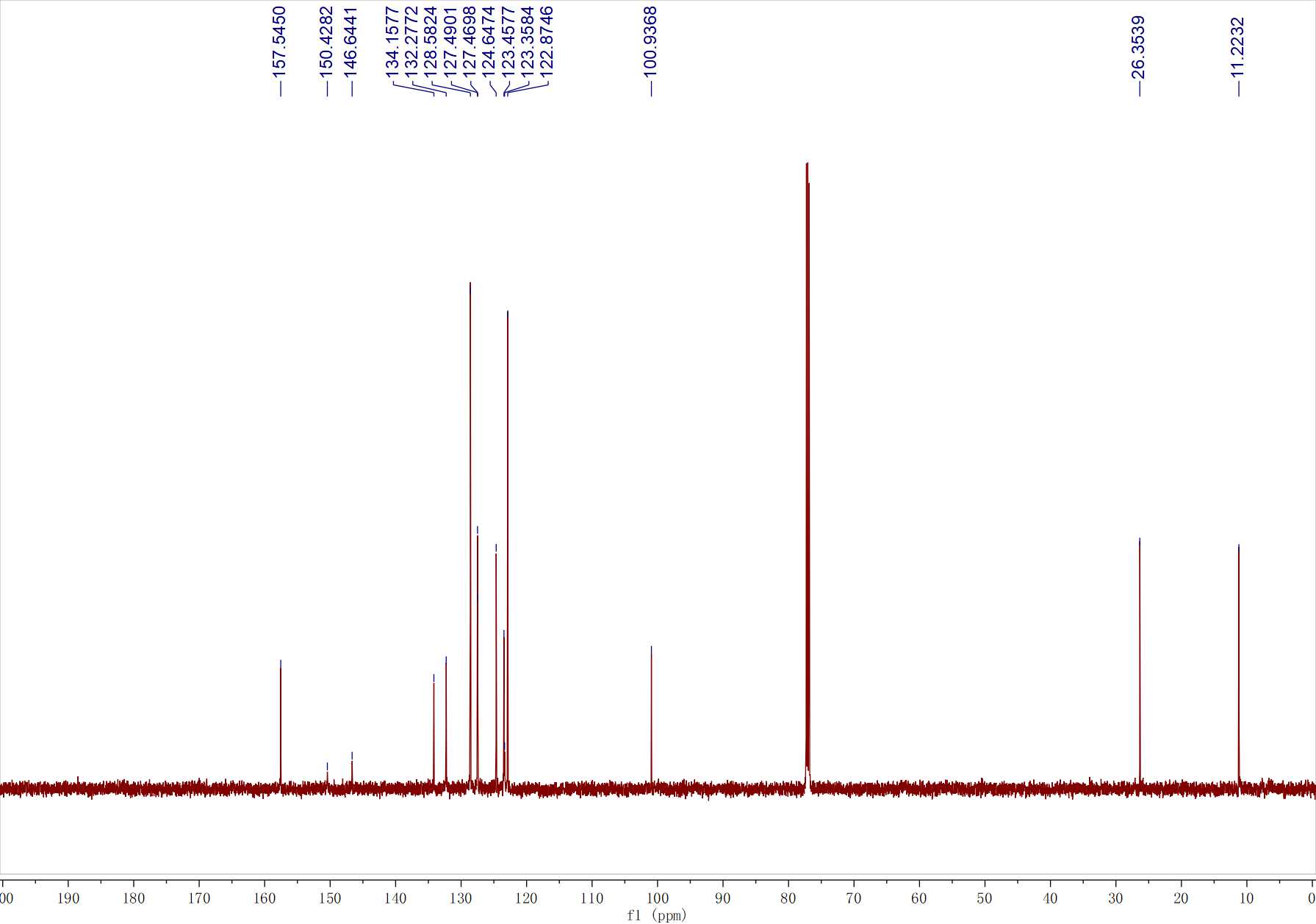


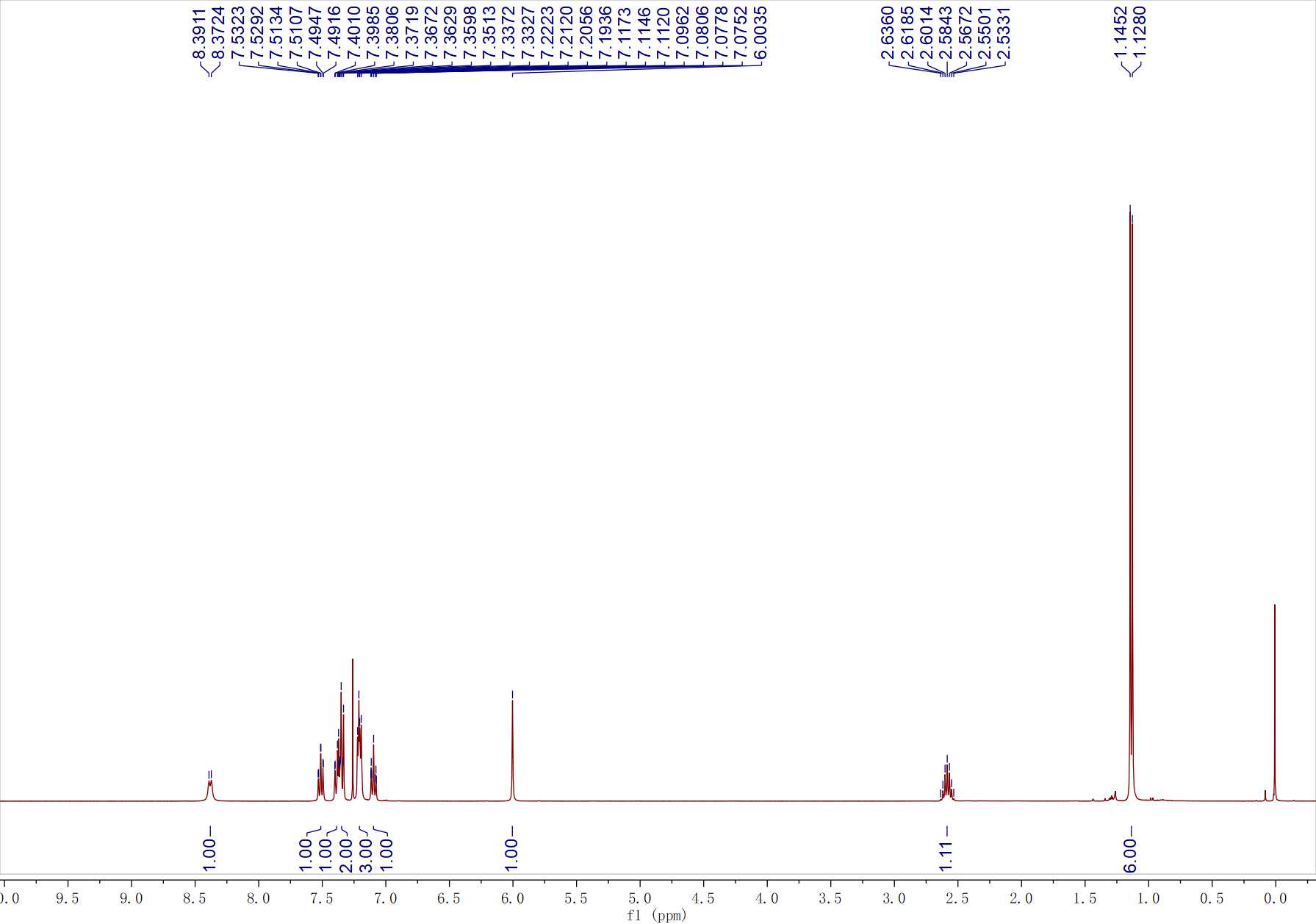


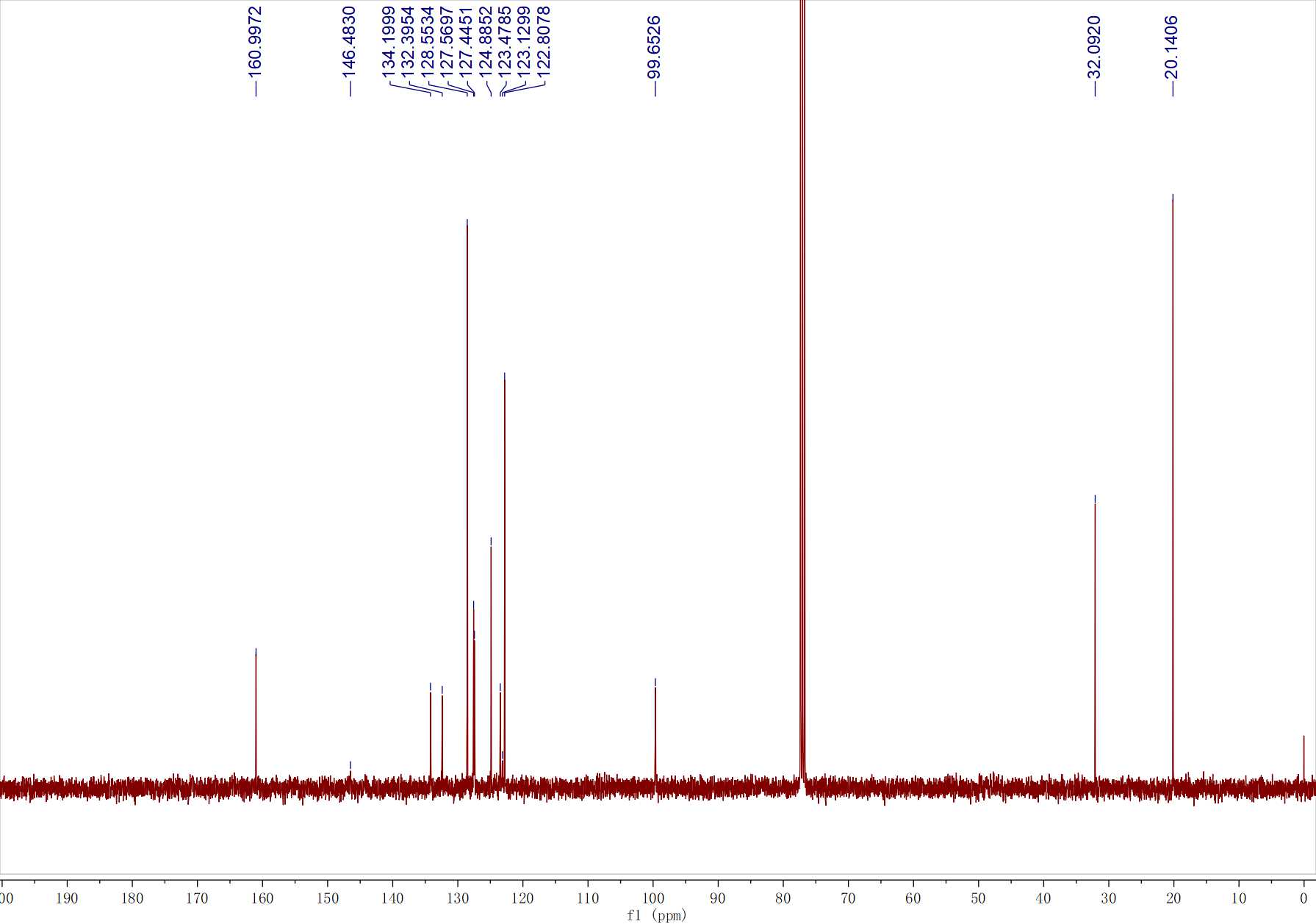


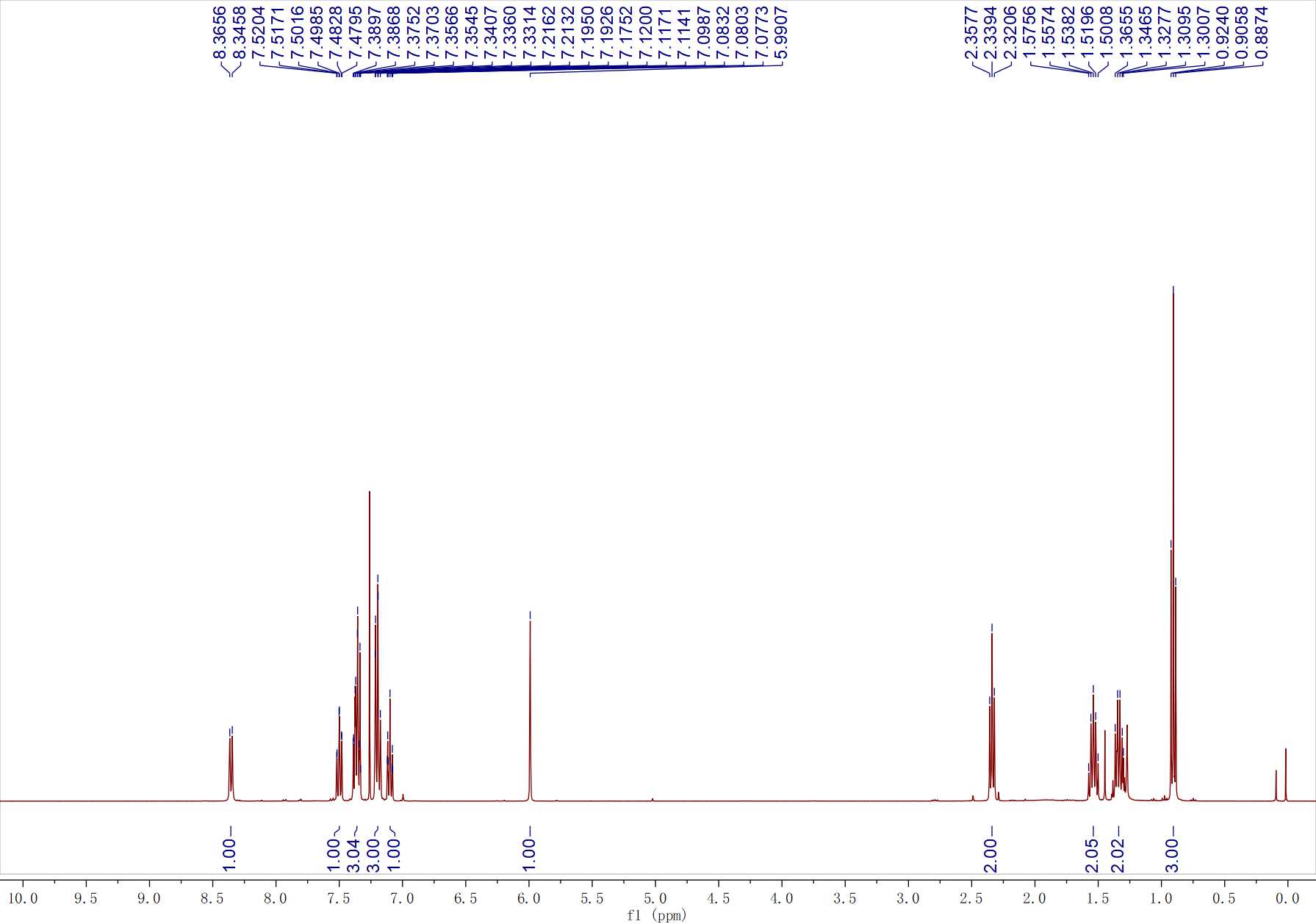


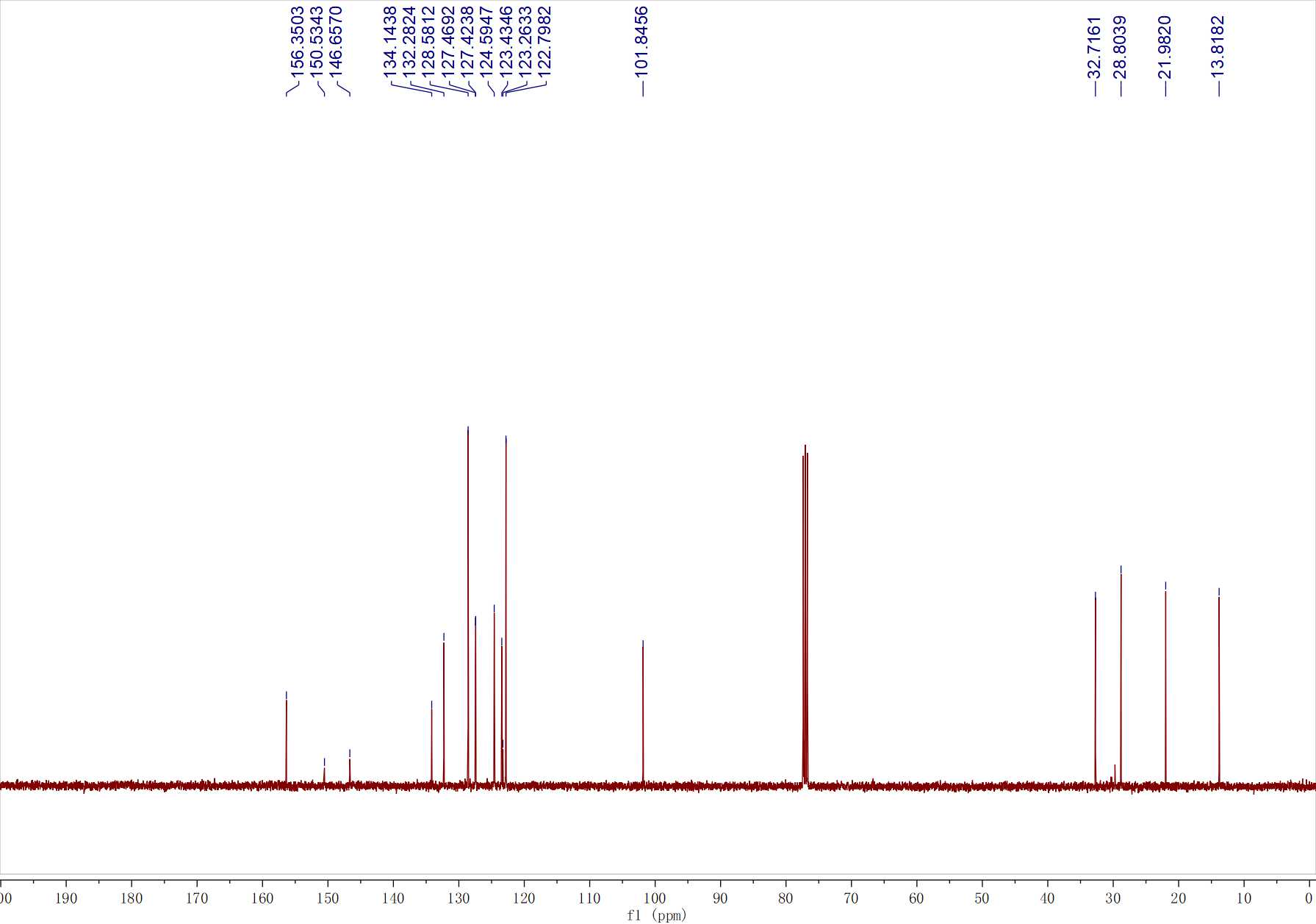


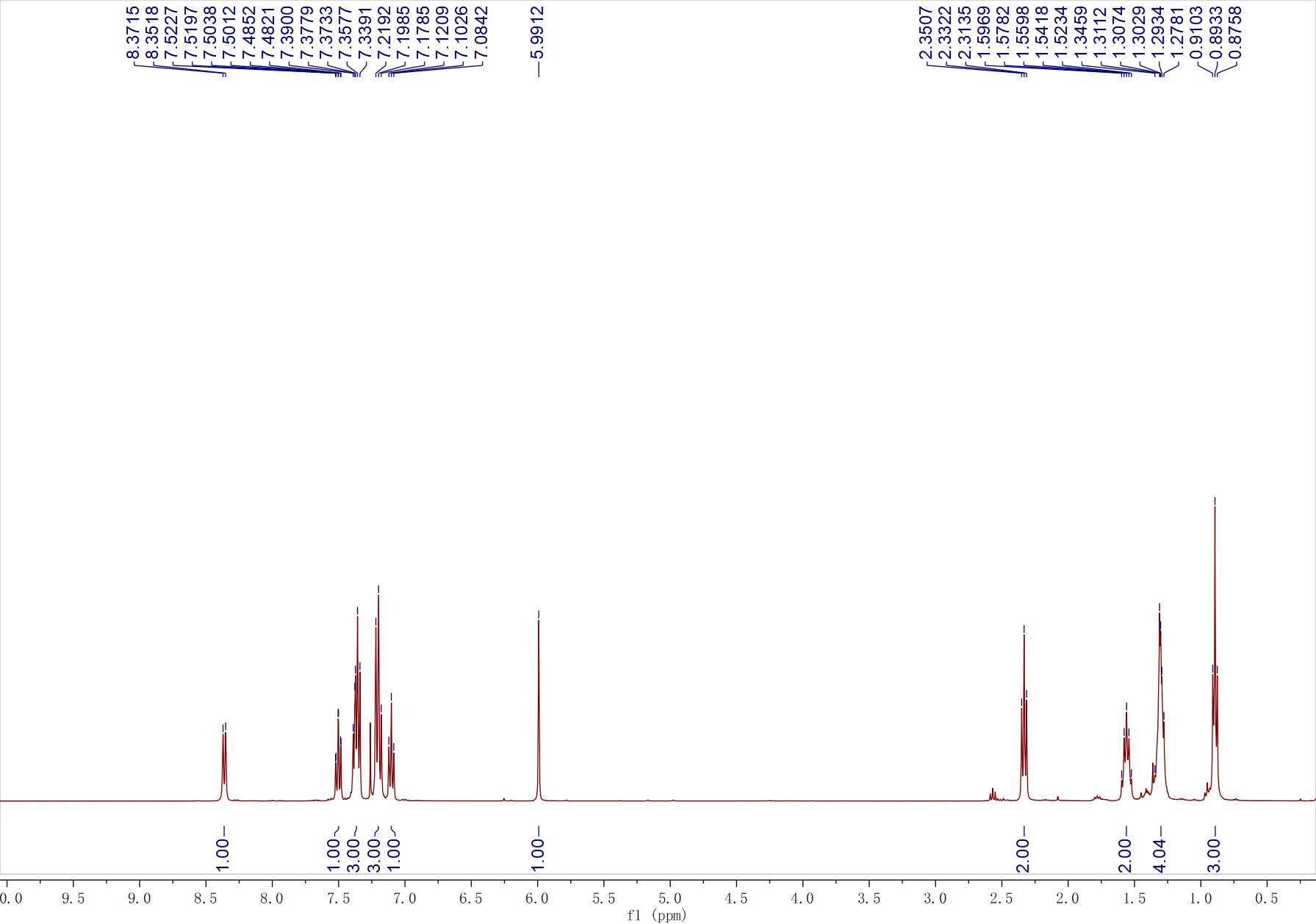


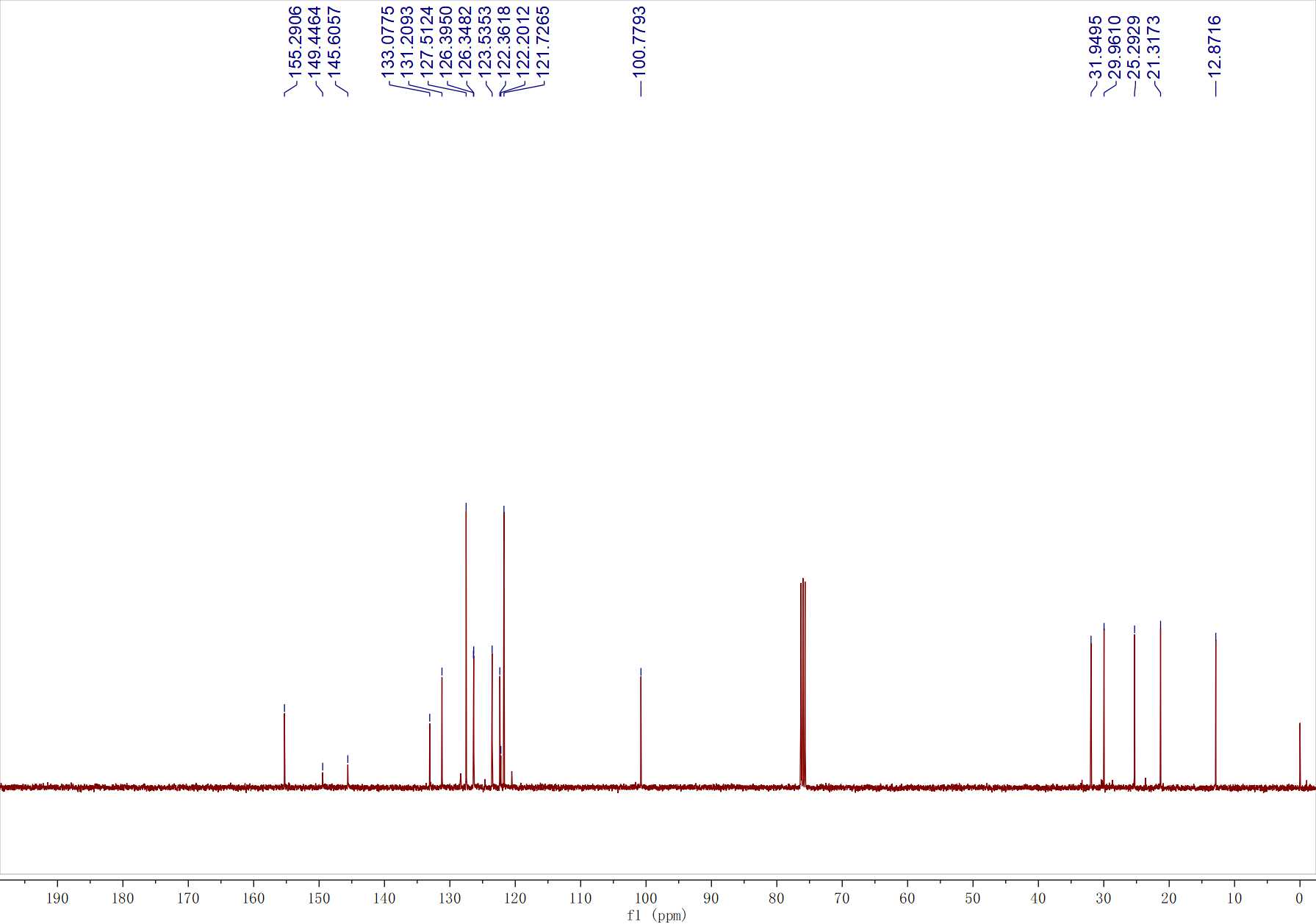


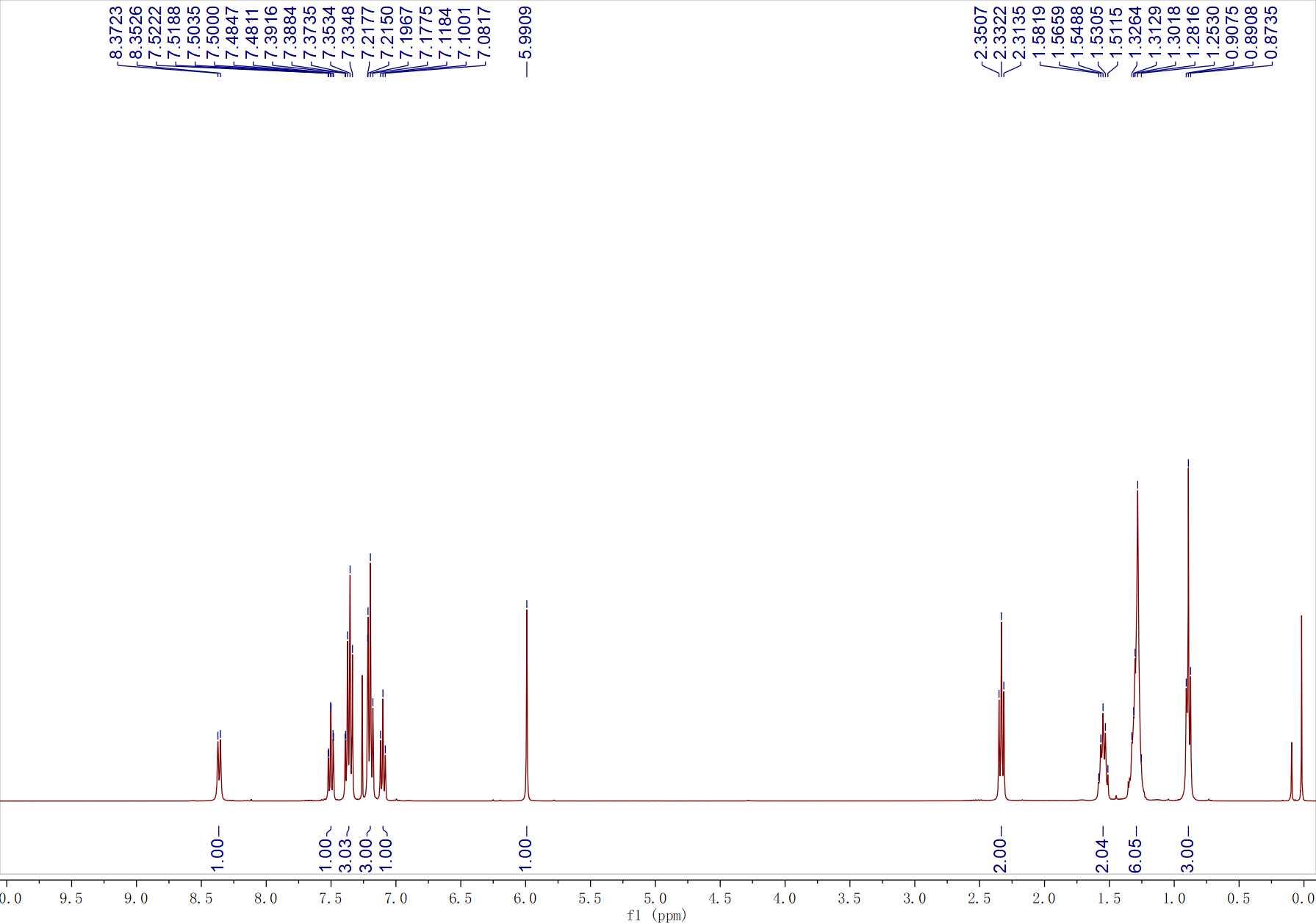


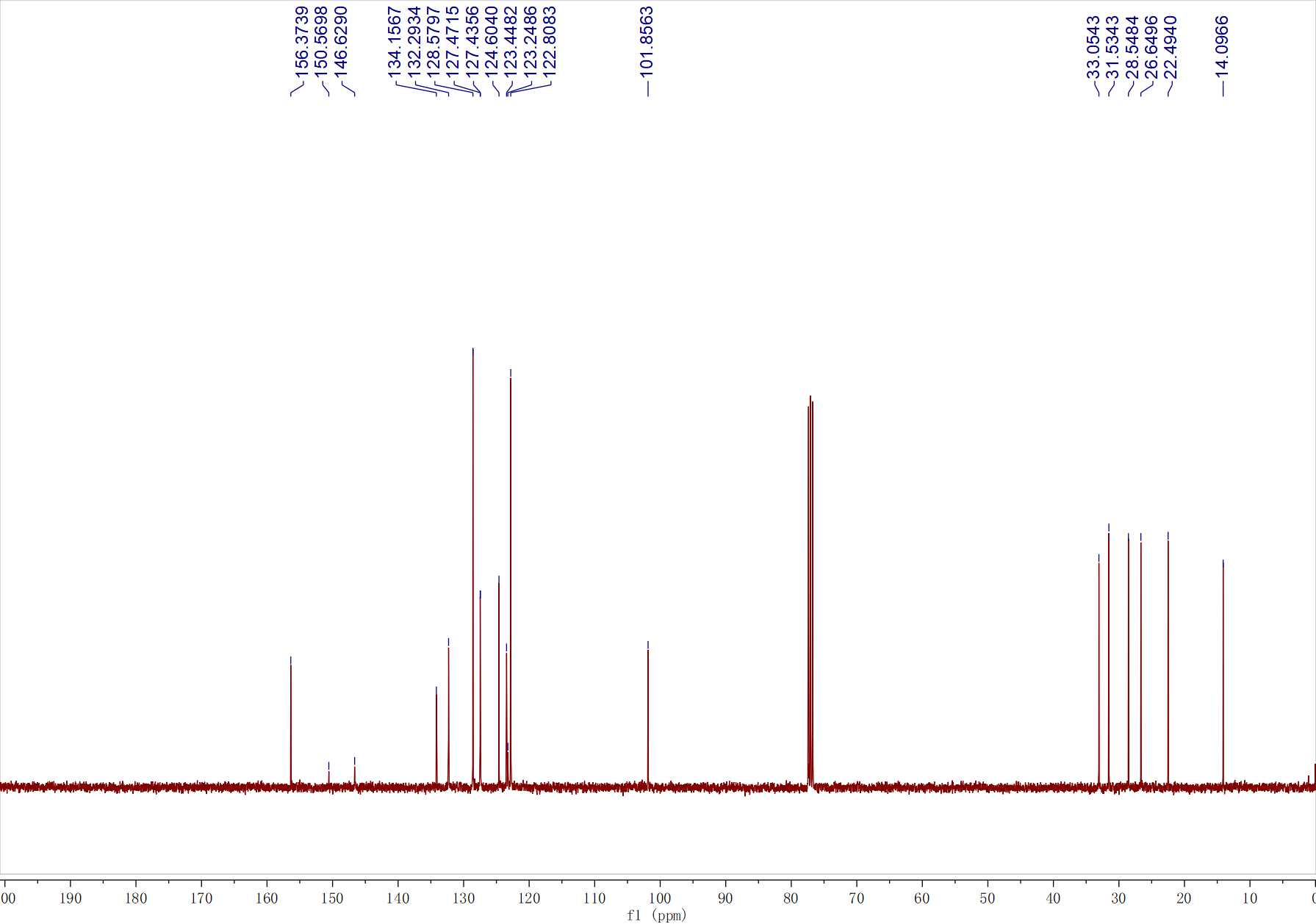


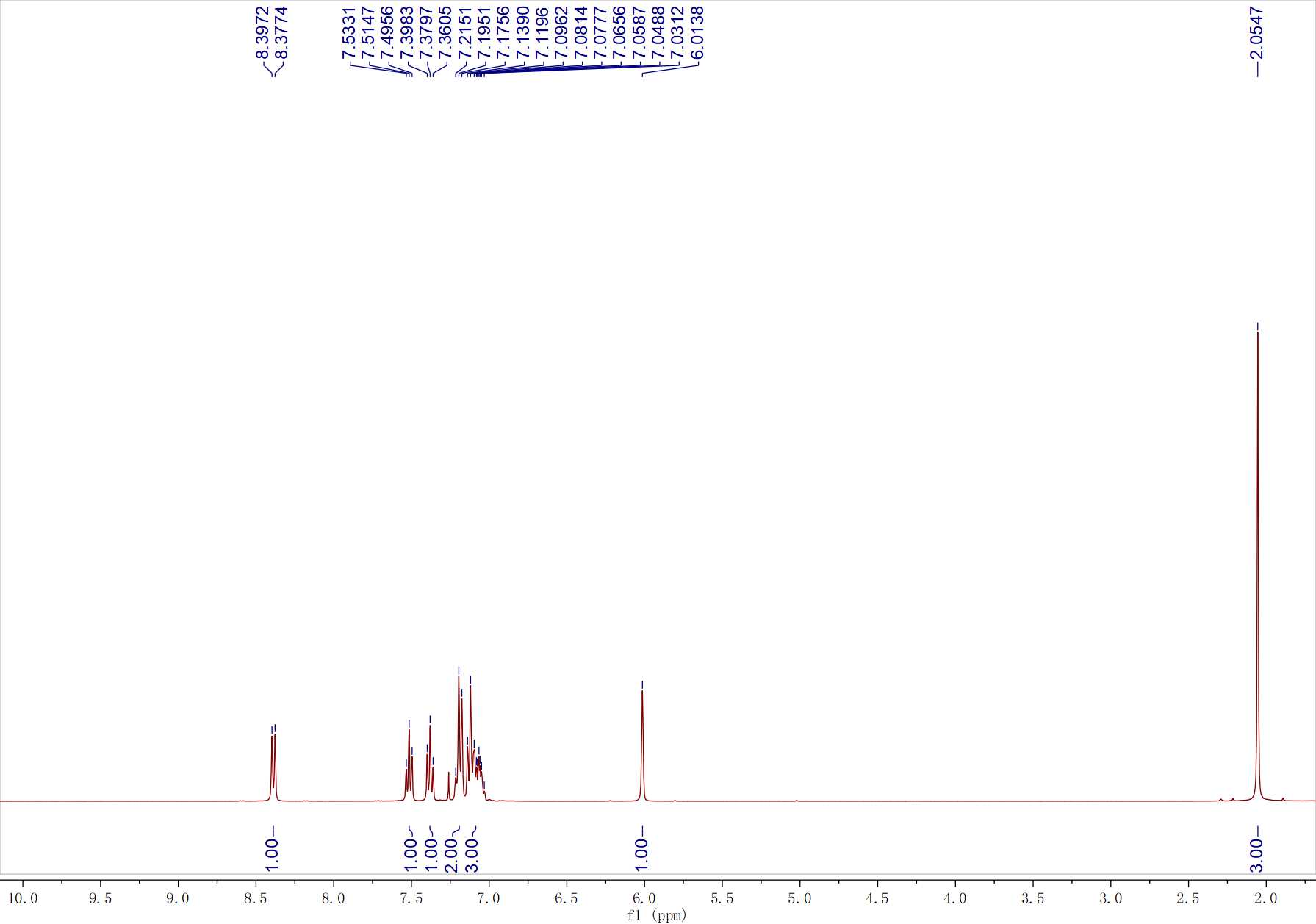


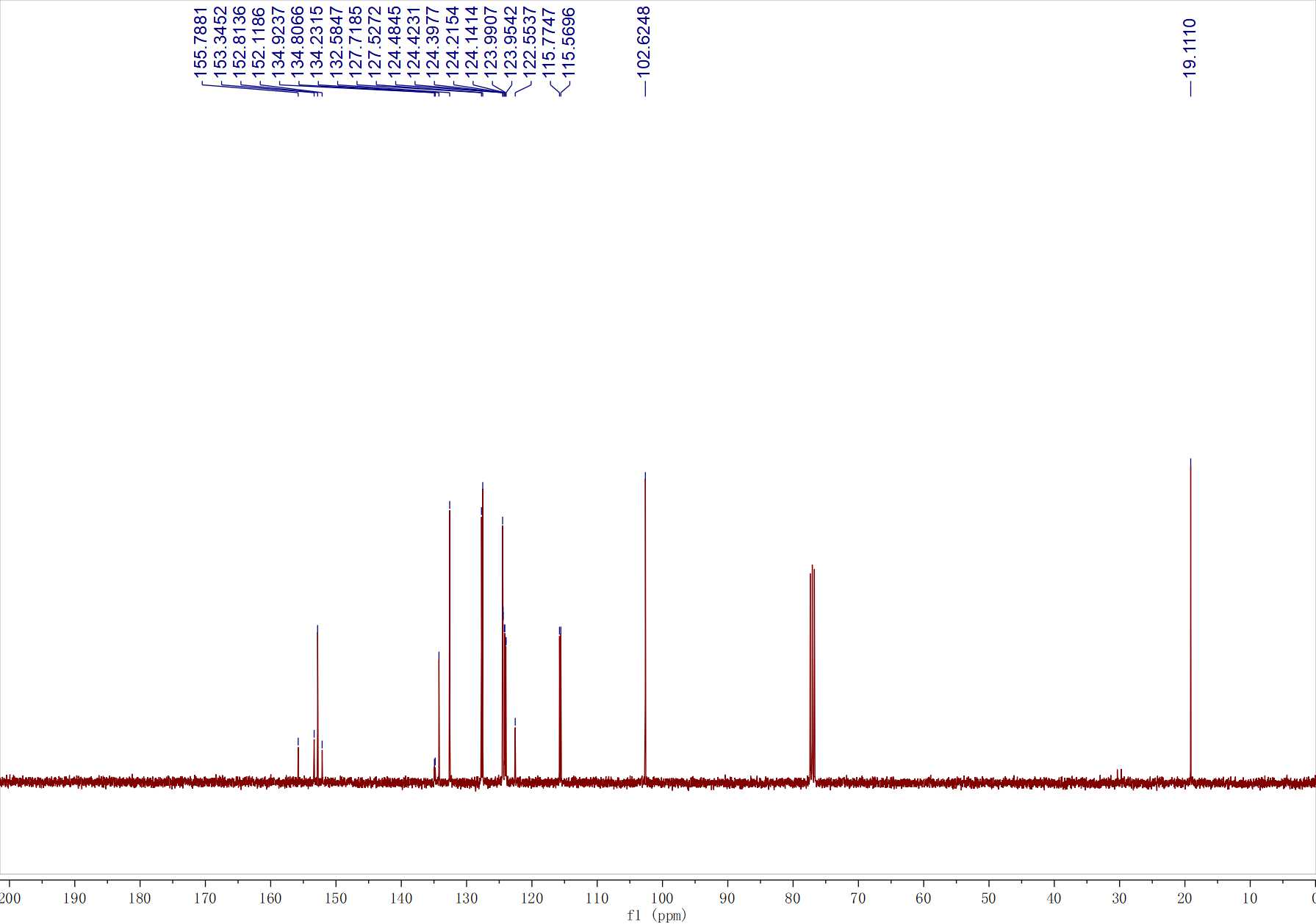

Supplement: Supplementary file 4 — Supplementary Data 1 [file 42004_2023_930_MOESM4_ESM.docx]
