# Supplementary material for: Chemoselective cycloisomerization of O-alkenylbenzamides via concomitant 1,2-aryl migration/elimination mediated by hypervalent iodine reagents
Source: Commun Chem. 2023 Jun 17;6:126. doi: 10.1038/s42004-023-00930-5 (PMC10276869; doi:10.1038/s42004-023-00930-5)
Supplement: Supplementary file 7 — Supplementary Data 4 [file 42004_2023_930_MOESM7_ESM.docx]

**DFT calculation data**

**Computational Methods**

Quantum chemical calculations were performed using the Gaussian 16 suite of programs.^1^ The B3LYP-D3(BJ) functional, i.e., the B3LYP functional^2^ including the D3 dispersion correction with the Becke-Johnson damping function^3,4^, was used for geometry optimizaions and frequencies calculations, and a mixed basis set of SDD^5,6^ for I and 6-31G(d,p)^7^ for other atoms in conjunction with the SMD^8^ implicit solvation model to account for the solvation effects of the methanol and 1,2-dichloroethane. The recently corrected radius for iodine was used for SMD calculations.^9^ Optimized geometries were verified by frequency computations as minima (zero imaginary frequencies) or transition structures (a single imaginary frequency) at the same level of theory. Intrinsic reaction coordinate (IRC) calculations were used to confirm the connectivity between transition structure and intermediate.^10,11^

More accurate electronic energies were obtained by single point energy calculations at the SMD-M062X/6-311++G(2df,2p)+Def2TZVP(I)^12-18^ level of theory. A factor of RTln(24.46) was added to free energy for each species to account for the 1 atm to 1 M standard state change. All Gibbs energies in solution reported throughout the text are in kilocalories per mole, and the bond lengths are in angstroms. The structures were generated by CYLview.^19^ The Hirshfeld charges analysis was calculated by Multiwfn.^20,21^ The temerature parameters was set as 337.85 K for condition A and 353.15 K for condition B in thermodynamic data calculations.

**SMD-B3LYP-D3(BJ)/6-31G(d,p)+SDD(I) Calculated Cartesian Coordinates and Single Point Energies Calculated Using the SMD-M062X/6-311++G(2df,2p)+Def2TZVP(I)// SMD-B3LYP-D3(BJ)/6-31G(d,p)+SDD(I).**

**Reagent 1**

C 3.01185100 1.00024800 0.68241300

C 3.70683500 -0.00027400 0.00000300

C 3.01169600 -1.00070000 -0.68238800

C 1.61308800 -1.01142000 -0.69021100

C 0.96670100 -0.00007400 0.00002500

C 1.61324200 1.01117700 0.69026100

H 3.55069800 1.77784100 1.21524200

H 4.79249800 -0.00035100 -0.00001000

H 3.55041800 -1.77837400 -1.21522500

H 1.04714600 -1.78109200 -1.20171600

H 1.04742800 1.78092200 1.20179800

I -1.22284800 0.00010000 -0.00002300

C -0.84299800 2.90653500 -0.67469200

H 0.15453100 2.70857700 -1.09678800

H -1.58474800 2.78498600 -1.47934000

H -0.86677500 3.95557600 -0.35150000

O -1.12488500 2.09743100 0.45664600

C -0.84376900 -2.90645100 0.67474800

H -0.86735400 -3.95547400 0.35148000

H 0.15355000 -2.70849500 1.09733000

H -1.58589200 -2.78499400 1.47906500

O -1.12517100 -2.09726700 -0.45665300

Zero-point correction= 0.173242 (Hartree/Particle)

Thermal correction to Energy= 0.189164

Thermal correction to Enthalpy= 0.190234

Thermal correction to Gibbs Free Energy= 0.125209

SCF Done: E(RM062X) = -759.391176500

**TMSOTf**

C 2.78764500 1.40828500 0.55888900

H 2.81546700 2.14027800 -0.25530500

H 2.22955100 1.84031300 1.39492100

H 3.81799000 1.23597700 0.89184500

C 1.69819300 -1.39798400 1.37485200

C 2.87824900 -0.95967500 -1.47879700

H 1.20541200 -2.30492600 1.01211700

H 2.65270500 -1.69238600 1.82768000

H 1.08024800 -0.95136500 2.15916800

H 3.88629300 -1.28773400 -1.19913100

H 2.32785800 -1.83391900 -1.84172500

H 2.97255200 -0.24266500 -2.30087000

Si 2.03280300 -0.19644500 -0.00797900

O 0.45704700 0.22731100 -0.73853800

S -0.79927800 0.87884800 -0.06505100

O -1.40496200 1.83265800 -0.98858200

O -0.53571400 1.26510400 1.32105900

C -1.93027300 -0.60069800 -0.01279800

F -2.07811800 -1.09029100 -1.24118100

F -1.41003500 -1.53344300 0.78335100

F -3.11103800 -0.21127100 0.46443300

Zero-point correction= 0.140346 (Hartree/Particle)

Thermal correction to Energy= 0.159384

Thermal correction to Enthalpy= 0.160454

Thermal correction to Gibbs Free Energy= 0.088955

SCF Done: E(RM062X) = -1370.77769695

**1a'**

C -3.16329400 -0.77286000 -1.36531800

C -2.86383500 -1.89985400 -0.59920300

C -1.89616800 -1.82702700 0.40021800

C -1.20094100 -0.63644400 0.66391600

C -1.48484300 0.48634300 -0.14295900

C -2.47577500 0.41494600 -1.13114700

H -3.91877400 -0.81918600 -2.14338900

H -3.38864000 -2.83444600 -0.77306700

H -1.69081900 -2.70420000 1.00453400

H -2.68270600 1.29254700 -1.73535300

C -0.21956700 -0.58846500 1.78175000

C -0.21576600 0.43074700 2.65100200

H 0.50478400 0.47383300 3.46305800

H -0.93282600 1.24391400 2.58769300

C -0.71836500 1.76310100 -0.05223000

O -1.53630900 2.81926800 0.17477100

N 0.53308200 1.96887100 -0.19863000

H -0.98783900 3.62480700 0.15315000

C 1.43026200 0.92787400 -0.50201800

C 1.24742400 0.05555200 -1.58698300

C 2.59237000 0.81210700 0.27615700

C 2.19701200 -0.92740100 -1.86474700

H 0.36571100 0.15628000 -2.21067700

C 3.53242400 -0.17829000 -0.00032500

H 2.73505000 1.49777500 1.10555000

C 3.33925700 -1.05569200 -1.07093000

H 2.04260000 -1.59469500 -2.70802800

H 4.41989900 -0.26292400 0.62031200

H 4.07469700 -1.82398000 -1.28942000

C 0.74179500 -1.74426900 1.89880000

H 0.22030900 -2.67118400 2.16619800

H 1.25216600 -1.92718400 0.94788300

H 1.49336100 -1.54980200 2.66789400

Zero-point correction= 0.269304 (Hartree/Particle)

Thermal correction to Energy= 0.288936

Thermal correction to Enthalpy= 0.290006

Thermal correction to Gibbs Free Energy= 0.218557

SCF Done: E(RM062X) = -748.644796596

**TS-tautomerism**

C -4.71043500 3.01639500 -1.02190600

C -5.40318800 2.72463500 0.15379700

C -4.87393100 1.81807800 1.06971300

C -3.64625600 1.17629400 0.84106100

C -2.97519600 1.45161500 -0.36961600

C -3.49610100 2.38341400 -1.27465100

H -5.11363400 3.72535700 -1.73823100

H -6.35219500 3.20898700 0.36344200

H -5.41215300 1.62051900 1.99068400

H -2.95377400 2.58684000 -2.19285000

C -3.09065300 0.25132000 1.86698700

C -1.79785700 0.29965500 2.21810300

H -1.39392100 -0.38425800 2.95912900

H -1.10994800 1.02318100 1.79209400

C -1.70113500 0.76754100 -0.75336600

O -0.68689000 1.57286000 -0.90711200

N -1.57310000 -0.51619200 -0.94607600

H -0.26050300 -1.11166500 -0.98276800

C -2.63977100 -1.43299500 -0.81206200

C -3.87116400 -1.26827900 -1.46310400

C -2.41867700 -2.58735900 -0.04600500

C -4.87314000 -2.22682800 -1.31629100

H -4.03740900 -0.39492200 -2.08349000

C -3.42340300 -3.54267300 0.09377700

H -1.46026100 -2.71678800 0.44640900

C -4.65823700 -3.36475900 -0.53497300

H -5.82355600 -2.08561700 -1.82292100

H -3.24111400 -4.42662500 0.69799800

H -5.44126700 -4.10882600 -0.42494700

C -4.05166100 -0.72380200 2.49964100

H -4.79107100 -0.20842000 3.12422100

H -4.60799700 -1.27682800 1.73604800

H -3.52037700 -1.43898800 3.13252900

C 4.73799900 -3.00856800 -1.02658500

C 5.44994500 -2.69089600 0.13078700

C 4.92567400 -1.78251500 1.04782200

C 3.68325200 -1.16374700 0.83836500

C 2.99343000 -1.46471600 -0.35559400

C 3.50859100 -2.39875200 -1.26171100

H 5.13792200 -3.71940700 -1.74270500

H 6.41099100 -3.15719900 0.32574300

H 5.47955500 -1.56596500 1.95502500

H 2.95106200 -2.62052800 -2.16628800

C 3.13074800 -0.23844900 1.86520700

C 1.84583900 -0.30790700 2.24102600

H 1.44254400 0.37614300 2.98217300

H 1.16429600 -1.05015000 1.83731200

C 1.70720600 -0.80257700 -0.72135100

O 0.70295600 -1.62812000 -0.86326900

N 1.55405600 0.47705900 -0.91604100

H 0.32953900 1.01484000 -0.98724400

C 2.60433700 1.42022700 -0.80364400

C 3.82957000 1.26946100 -1.46636900

C 2.36427400 2.57475800 -0.04636500

C 4.81385400 2.24864300 -1.33818500

H 4.00580000 0.39334600 -2.07982700

C 3.35163300 3.55078100 0.07350800

H 1.40806600 2.69002300 0.45318100

C 4.58312300 3.38948400 -0.56570500

H 5.76190100 2.12130500 -1.85247300

H 3.15827900 4.43711000 0.67045500

H 5.35257700 4.14950800 -0.47037600

C 4.08556700 0.75998700 2.46992400

H 4.84391500 0.26378300 3.08722600

H 4.61922400 1.31353400 1.69085700

H 3.55284400 1.47292400 3.10403200

Zero-point correction= 0.533135 (Hartree/Particle)

Thermal correction to Energy= 0.573554

Thermal correction to Enthalpy= 0.574624

Thermal correction to Gibbs Free Energy= 0.455083

SCF Done: E(RM062X) = -1497.30769763

**IM2-1**

C 4.23954300 -1.07542800 -1.94514400

C 5.17547300 -0.75472800 -0.95950600

C 4.76420300 -0.49316700 0.34907900

C 3.40996300 -0.54555100 0.68538900

C 2.49799300 -0.86123300 -0.32051100

C 2.88027400 -1.13424300 -1.63284400

H 4.56078300 -1.27975700 -2.96154400

H 6.23003000 -0.70836700 -1.21238300

H 5.49268100 -0.24749700 1.11510000

H 3.08798000 -0.33662800 1.69852100

H 2.15042200 -1.37948400 -2.39590000

I 0.42250600 -0.91344300 0.15748100

C 0.89608900 -3.22144500 1.90306100

H 1.92306500 -2.88930900 2.09626400

H 0.21639100 -2.79641800 2.65021100

H 0.85976600 -4.31346200 1.96669300

O 0.47902800 -2.91593700 0.56313900

C 0.26631200 1.70009300 -1.90498400

H 0.77887200 2.58376700 -2.29633600

H 0.56475000 0.83822500 -2.50692400

H -0.81559800 1.83822600 -1.97231200

O 0.65615000 1.45641100 -0.54098500

C 0.81686100 2.03485300 2.26710900

H 0.92159400 2.84234600 3.00218000

H -0.13504800 1.53122700 2.46018800

H 1.63207000 1.32676800 2.44547400

C -0.52565700 3.98968500 0.27250100

C 2.54881000 3.50877300 0.16813700

H -0.44544700 4.82178300 0.98219700

H -0.50595600 4.41314900 -0.73728400

H -1.49671100 3.50773300 0.42451800

H 2.60137600 3.88851000 -0.85829900

H 2.77100000 4.34392600 0.84314700

H 3.33529300 2.75545100 0.29164900

Si 0.86942700 2.77500700 0.55174800

O -2.24122300 0.98482500 0.79907000

S -3.07847800 -0.23291000 0.67435300

O -4.38696700 -0.18982400 1.35647700

O -2.31263600 -1.49941100 0.86333000

C -3.50531400 -0.26711500 -1.12673900

F -4.18063100 0.83721900 -1.46883400

F -2.38380300 -0.32378700 -1.86847100

F -4.25541000 -1.33817900 -1.41350900

Zero-point correction= 0.316134 (Hartree/Particle)

Thermal correction to Energy= 0.353378

Thermal correction to Enthalpy= 0.354448

Thermal correction to Gibbs Free Energy= 0.237089

SCF Done: E(RM062X) = -2130.21112574

**TMSOMe**

C -2.32189300 0.00008500 -0.24491900

H -2.46007000 0.89066900 0.38164800

H -3.09796900 0.00056500 -1.01670100

H -2.46022700 -0.89113600 0.38070600

O -1.04949000 0.00030400 -0.88931700

C 1.74899500 0.00059400 -1.26660000

H 2.72982100 0.00061700 -0.77651700

H 1.69863900 -0.88562500 -1.90920300

H 1.69832600 0.88717300 -1.90868100

C 0.45951400 -1.54010700 1.08367900

C 0.45938200 1.53920800 1.08494900

H 1.41398200 -1.57838000 1.62269100

H -0.34009700 -1.56125500 1.83262300

H 0.38108500 -2.45204000 0.48073600

H -0.34027800 1.55973400 1.83385600

H 1.41381400 1.57706800 1.62405200

H 0.38096800 2.45161400 0.48272000

Si 0.37871000 -0.00000800 0.01028300

Zero-point correction= 0.153099 (Hartree/Particle)

Thermal correction to Energy= 0.166056

Thermal correction to Enthalpy= 0.167126

Thermal correction to Gibbs Free Energy= 0.111099

SCF Done: E(RM062X) = -524.401617930

**OTf anion**

O 1.24115900 1.33531900 -0.54868900

S 0.90043400 0.00000100 -0.00021000

O 1.24306800 -0.19203500 1.43005500

O 1.24333300 -1.14259600 -0.88145300

C -0.95255600 -0.00031400 0.00002300

F -1.42584400 0.99181700 0.77090000

F -1.42648200 0.17128400 -1.24431500

F -1.42679400 -1.16350600 0.47385000

Zero-point correction= 0.027073 (Hartree/Particle)

Thermal correction to Energy= 0.035864

Thermal correction to Enthalpy= 0.036934

Thermal correction to Gibbs Free Energy= -0.011031

SCF Done: E(RM062X) = -961.672303987

**IM2-2**

C 2.23065900 2.73145500 0.72559800

C 2.18541000 3.35752200 -0.52080200

C 2.15668500 2.60269900 -1.69581100

C 2.17580100 1.20849000 -1.64003500

C 2.20893000 0.62336200 -0.37737700

C 2.23758400 1.33821700 0.81619800

H 2.24588200 3.31895100 1.63715000

H 2.16694400 4.44106300 -0.57779700

H 2.11843600 3.09220000 -2.66330200

H 2.15104100 0.61679200 -2.54734300

H 2.24792600 0.84517400 1.77913200

I 2.20986900 -1.51846700 -0.25313900

C 4.75513100 -1.23622800 1.28066900

H 5.83200800 -1.44223600 1.26831000

H 4.60862100 -0.16105000 1.43828400

H 4.29825900 -1.80075800 2.10272000

O 4.29031600 -1.66161900 -0.00230200

C -0.93749900 2.84766500 2.00595100

C -0.64508500 1.72057800 2.77178800

C -0.65870200 0.45887600 2.18315200

C -0.96864400 0.28838500 0.82281600

C -1.30927600 1.43538800 0.06580600

C -1.26718600 2.69998100 0.65899100

H -0.91947200 3.83677600 2.45217700

H -0.39277000 1.82076100 3.82257500

H -0.39223500 -0.40433200 2.78150700

H -1.52855800 3.56893600 0.06463000

C -0.89612200 -1.06468500 0.23626900

C -0.45218100 -1.27447200 -1.04346700

H -0.51990600 -2.26520800 -1.48346900

H -0.21331500 -0.45880900 -1.71524000

C -1.85518900 1.36912900 -1.32144400

O -1.17018100 2.16952200 -2.16730800

N -2.86176500 0.71050100 -1.74911200

C -3.64536900 -0.09110600 -0.89523000

C -4.24104500 0.40489800 0.27552100

C -3.88178400 -1.42260000 -1.27081300

C -5.03130300 -0.42858500 1.06657100

H -4.08313400 1.44042000 0.55828500

C -4.66570500 -2.25266100 -0.47119500

H -3.43221600 -1.79573600 -2.18572300

C -5.24171200 -1.76180500 0.70376900

H -5.48534300 -0.03210600 1.97043500

H -4.82851900 -3.28476800 -0.76832600

H -5.85594300 -2.40738700 1.32425300

C -1.29504400 -2.23106800 1.09362000

H -0.60601200 -2.35953800 1.93619200

H -2.29019400 -2.06067600 1.51587600

H -1.30181800 -3.15612800 0.51438100

H -1.62157100 2.14906800 -3.03097100

Zero-point correction= 0.402891 (Hartree/Particle)

Thermal correction to Energy= 0.436315

Thermal correction to Enthalpy= 0.437385

Thermal correction to Gibbs Free Energy= 0.331809

SCF Done: E(RM062X) = -1392.76083767

**TS2-1**

C -0.34556500 1.24901300 1.03530700

C -1.16413000 2.16555300 0.34055100

C -0.77613400 3.50264900 0.21948200

C 0.41492900 3.93357800 0.80250100

C 1.20161700 3.04001600 1.53113900

C 0.81714500 1.70512900 1.66213000

H -1.41711200 4.22125600 -0.28257100

H 0.71361200 4.97269800 0.71112300

H 2.12101300 3.37939800 1.99620100

H 1.44402100 1.01265600 2.21256000

N -2.92687400 0.61427500 0.49679000

C -4.00352500 -0.16466900 0.05791700

C -4.16229400 -0.54719600 -1.28723400

C -4.88345700 -0.67448800 1.02699100

C -5.19613700 -1.40881300 -1.64788300

H -3.47397900 -0.17212600 -2.03641100

C -5.92396300 -1.52182000 0.65401900

H -4.73855800 -0.39189700 2.06517600

C -6.08434300 -1.89394500 -0.68389000

H -5.30828100 -1.70257800 -2.68766700

H -6.60538700 -1.90089600 1.41007600

H -6.89085400 -2.56140700 -0.97209700

C -2.46463300 1.64514200 -0.11699300

C -0.71008500 -0.18731400 1.06381500

C -0.85294200 -0.84237400 2.39688300

C -0.64481600 -0.95128300 -0.10642000

H -1.16731300 -1.88354000 2.30318100

H 0.10882300 -0.81052000 2.92581900

H -1.56802200 -0.28936000 3.01139000

H -1.09245200 -1.94261500 -0.08531300

H -0.68406500 -0.44122900 -1.06320600

I 1.75818200 -1.80320000 -0.18025800

C 2.47859800 0.18406600 -0.66776100

C 1.82705100 0.91784400 -1.64771100

C 3.58326200 0.63698300 0.04106600

C 2.30759400 2.20431400 -1.90990500

H 0.98285000 0.52600700 -2.20116600

C 4.05026700 1.91987000 -0.25284800

H 4.06683500 0.03381300 0.79872600

C 3.41027200 2.70269100 -1.21540400

H 1.81520600 2.80598200 -2.66704500

H 4.90892300 2.30512200 0.28778800

H 3.77276500 3.70379100 -1.42548700

O 3.82842700 -2.42254200 -0.42767000

C 4.39044900 -2.93167200 0.77268900

H 5.42502700 -3.21260500 0.53514500

H 4.41698200 -2.18850000 1.58335100

H 3.87780900 -3.83326900 1.13925300

O -3.13297100 2.25099500 -1.11366600

H -2.58195900 2.91222700 -1.56264300

Zero-point correction= 0.401804 (Hartree/Particle)

Thermal correction to Energy= 0.434594

Thermal correction to Enthalpy= 0.435664

Thermal correction to Gibbs Free Energy= 0.331993

SCF Done: E(RM062X) = -1392.74286273

**TS2-1'**

C -0.24629100 0.71433900 1.27929200

C -1.32280600 1.51023600 0.87076200

C -1.23470100 2.90361900 0.86228400

C -0.04702300 3.49110400 1.28990100

C 1.01674400 2.69803500 1.73817200

C 0.92552400 1.30392500 1.74025600

H -2.08152100 3.50160800 0.54371300

H 0.04930700 4.57173900 1.29229700

H 1.92984000 3.17003800 2.08450000

H 1.75849400 0.70065000 2.08216900

N -3.56530200 1.06047300 -0.03353800

C -4.66207700 0.21505800 -0.29358300

C -5.13858100 -0.72757500 0.63362300

C -5.32999600 0.39022800 -1.51460300

C -6.25798800 -1.49843400 0.32115100

H -4.65607500 -0.83387900 1.59986200

C -6.43389600 -0.40022500 -1.82410600

H -4.96491200 1.13849900 -2.21070700

C -6.90243500 -1.34653500 -0.90823100

H -6.62700800 -2.21886400 1.04501100

H -6.93638100 -0.26895400 -2.77770100

H -7.77025500 -1.95349000 -1.14730800

C -2.49008300 0.72316500 0.51834100

C -0.48607700 -0.76174700 1.13025000

C -0.32191200 -1.59172700 2.37287800

C -0.05152100 -1.37351900 -0.15164900

H -0.65779900 -2.61768900 2.20666500

H 0.74422100 -1.61407600 2.62441600

H -0.85805000 -1.14636700 3.21225300

H -0.35421400 -2.41987600 -0.22580100

H -0.38944600 -0.81287000 -1.02042900

I 2.25568100 -1.59506100 -0.36811300

C 2.47254400 0.51859600 -0.87968000

C 1.51233800 1.13409400 -1.66695500

C 3.57383300 1.17543200 -0.35072700

C 1.65635800 2.50405600 -1.90981600

H 0.67425900 0.59644700 -2.09067000

C 3.69710900 2.54233700 -0.61211900

H 4.31263900 0.65104400 0.23955300

C 2.73890800 3.20620400 -1.38012700

H 0.91539500 3.01075000 -2.51996300

H 4.54362600 3.08356400 -0.20111100

H 2.83820900 4.27036700 -1.56921800

O 4.50101300 -1.62117900 -0.63978500

C 5.13577600 -2.00328200 0.55865400

H 6.21381800 -2.12358300 0.37123000

H 5.02731900 -1.25686500 1.36714300

H 4.77790700 -2.96967800 0.95878600

O -2.16881600 -0.62607300 0.95705600

H -2.56340100 -1.32989800 0.39809500

Zero-point correction= 0.402837 (Hartree/Particle)

Thermal correction to Energy= 0.434913

Thermal correction to Enthalpy= 0.435983

Thermal correction to Gibbs Free Energy= 0.334061

SCF Done: E(RM062X) = -1392.72515909

**IM2-3**

C -0.30531300 1.29910500 0.94786600

C -1.02585300 2.39498700 0.44993400

C -0.50622900 3.69113300 0.44216800

C 0.76961700 3.87187200 0.96940800

C 1.47728800 2.78903900 1.51159400

C 0.94662900 1.49509900 1.51428800

H -1.07989500 4.52531300 0.05087300

H 1.21377200 4.86149000 0.97822400

H 2.46105400 2.95671800 1.93711900

H 1.50887600 0.67611200 1.94622700

N -2.42468500 0.60033300 0.28921500

C -3.56872700 -0.19646800 -0.05210500

C -3.79807100 -0.52627600 -1.38966000

C -4.42560100 -0.62836500 0.96108400

C -4.90513800 -1.30972300 -1.71338100

H -3.11828000 -0.17114200 -2.15660800

C -5.52771100 -1.41469300 0.62491200

H -4.23454700 -0.34431000 1.98977100

C -5.76640900 -1.75601300 -0.70810400

H -5.09255800 -1.57098100 -2.75005300

H -6.20082900 -1.75503400 1.40536800

H -6.62634800 -2.36719100 -0.96446400

C -2.32577000 1.89585400 0.05254000

C -1.10590200 0.02473500 0.77333800

C -1.29202100 -0.74901300 2.07620500

C -0.61386800 -0.85944900 -0.38008600

H -1.91546700 -1.63157800 1.91649800

H -0.31575600 -1.07964900 2.43975200

H -1.74450500 -0.11399200 2.84091000

H -1.24256200 -1.74877900 -0.46288400

H -0.63960200 -0.32719600 -1.32834700

I 1.43543200 -1.79650500 -0.17921000

C 2.42594200 0.05573900 -0.79776300

C 1.81284900 0.89391900 -1.71623000

C 3.63139000 0.34802100 -0.17746600

C 2.43851000 2.11228000 -2.00088900

H 0.88335000 0.64285800 -2.20957100

C 4.23736300 1.57017900 -0.47863500

H 4.08693500 -0.35920900 0.50195000

C 3.63910500 2.45414700 -1.37859400

H 1.97647800 2.78667600 -2.71500500

H 5.17629900 1.82705100 0.00214300

H 4.11224200 3.40562800 -1.60007400

O 3.63155100 -2.57861200 -0.02072900

C 3.88232000 -2.97338700 1.30104500

H 4.80884300 -3.56997000 1.35492100

H 4.01348200 -2.12068700 1.99785300

H 3.08643900 -3.61195100 1.73503600

O -3.33704200 2.54305200 -0.46031600

H -3.14498900 3.49041500 -0.57646000

Zero-point correction= 0.405482 (Hartree/Particle)

Thermal correction to Energy= 0.437795

Thermal correction to Enthalpy= 0.438865

Thermal correction to Gibbs Free Energy= 0.336324

SCF Done: E(RM062X) = -1392.78841397

**IM2-4**

C -0.28741500 0.10143000 1.61758600

C 1.07442400 -0.23028700 1.60347300

C 1.54687700 -1.44980900 2.09344300

C 0.60880800 -2.34660400 2.59963700

C -0.75100800 -2.00970200 2.64052100

C -1.21206100 -0.77880400 2.16258300

H 2.60316500 -1.68466200 2.08532900

H 0.93575100 -3.30779100 2.98215700

H -1.46295900 -2.71663400 3.05357300

H -2.26596100 -0.53308100 2.21643700

N 0.94171000 1.91494500 0.83978800

C 1.31790900 3.14401700 0.20469200

C 1.68939100 3.13334700 -1.14250300

C 1.29719100 4.32950200 0.94001400

C 2.03991000 4.33298800 -1.75992100

H 1.69960600 2.19743400 -1.69096000

C 1.64467200 5.52536700 0.31023700

H 1.02267900 4.31039200 1.98865100

C 2.01424800 5.52809800 -1.03628600

H 2.33010200 4.33338500 -2.80588700

H 1.63112700 6.45206600 0.87522400

H 2.28485800 6.46074900 -1.52189900

C 1.79191100 0.91939500 1.07280200

C -0.49713800 1.47318200 1.01131700

C -1.27001500 2.42003000 1.92501400

C -1.06589000 1.42749800 -0.41365800

H -1.34544500 3.41322800 1.47645100

H -2.28182100 2.03375600 2.07376300

H -0.78497100 2.49597300 2.90069200

H -1.20721200 2.43954900 -0.79826200

H -0.40041200 0.88852200 -1.08413900

I -3.13093700 0.54882500 -0.67971200

C -2.31610000 -1.47707000 -0.70441600

C -1.09767400 -1.71222700 -1.32243100

C -3.03674300 -2.45505700 -0.03441000

C -0.55248300 -2.99680600 -1.22134600

H -0.56820500 -0.94733000 -1.87513800

C -2.47518100 -3.73148500 0.04922600

H -3.99971100 -2.23357000 0.40499400

C -1.23224300 -3.99893300 -0.52892400

H 0.40235400 -3.20171100 -1.69393100

H -3.01214400 -4.51126700 0.58047400

H -0.79818300 -4.99048700 -0.44764200

O -5.15518700 -0.59398000 -0.94480000

C -5.99303500 -0.30521700 0.14020300

H -7.02682700 -0.62690400 -0.07342700

H -5.69139000 -0.81439900 1.07848000

H -6.05772600 0.77589800 0.37988900

Si 3.87097800 -1.95025200 -1.05707200

C 2.58374700 -0.88365700 -1.89725900

H 2.96992900 0.12056400 -2.10182100

H 2.28952500 -1.33081500 -2.85379500

H 1.68429100 -0.79267400 -1.28185500

C 5.42822800 -2.13869700 -2.07650700

H 5.86363000 -1.16287600 -2.31925300

H 6.18747800 -2.73134900 -1.55456100

H 5.20262100 -2.64971500 -3.02017500

C 3.18247300 -3.60756300 -0.52378700

H 3.90833400 -4.17566500 0.06873700

H 2.27236000 -3.48913800 0.07251000

H 2.93409400 -4.21342100 -1.40339200

O 4.26954700 -1.05044100 0.35864600

C 5.28482200 -1.48626000 1.27930400

H 6.26816500 -1.46618200 0.80022900

H 5.28387600 -0.79605800 2.12568300

H 5.07353300 -2.49719800 1.64123500

O 3.05290700 1.05185100 0.83679500

H 3.56559400 0.14307200 0.73010600

Zero-point correction= 0.559609 (Hartree/Particle)

Thermal correction to Energy= 0.606124

Thermal correction to Enthalpy= 0.607194

Thermal correction to Gibbs Free Energy= 0.472732

SCF Done: E(RM062X) = -1917.21045213

**MeOH**

C 0.66645400 -0.01874600 0.00000000

H 1.03120900 -0.54774400 -0.89144000

H 1.09308000 0.98849500 0.00001200

H 1.03120800 -0.54776400 0.89142800

O -0.75301900 0.12338800 0.00000000

H -1.13007100 -0.76761700 0.00000000

Zero-point correction= 0.051280 (Hartree/Particle)

Thermal correction to Energy= 0.055182

Thermal correction to Enthalpy= 0.056252

Thermal correction to Gibbs Free Energy= 0.024880

SCF Done: E(RM062X) = -115.719309377

**IM2-5**

C 1.79405400 1.62603300 -0.72850800

C 2.67024200 2.30073300 0.12273200

C 2.52466300 3.65488300 0.40818600

C 1.46538500 4.33521300 -0.19562900

C 0.60464900 3.67042300 -1.07879700

C 0.76439000 2.30861700 -1.36224400

H 3.22071300 4.15790600 1.07205500

H 1.31501000 5.39123700 0.00564400

H -0.20119100 4.21965800 -1.55489200

H 0.10069400 1.81541600 -2.06278000

N 3.47095000 0.16361500 -0.06061900

C 4.26387900 -1.00234700 0.16981500

C 4.26405900 -1.60165900 1.43397700

C 5.04670200 -1.52719500 -0.86221200

C 5.04394100 -2.73517600 1.66074200

H 3.65718200 -1.17611100 2.22653300

C 5.81547400 -2.66882300 -0.63130300

H 5.05845500 -1.03804600 -1.82971900

C 5.81539300 -3.27316200 0.62758300

H 5.04549700 -3.19919700 2.64233800

H 6.42110000 -3.07901000 -1.43367300

H 6.41811100 -4.15881700 0.80499500

C 3.72720600 1.36922700 0.56649700

O 4.68006700 1.59414600 1.31212400

C 2.16635400 0.15537300 -0.78354500

C 2.25979800 -0.41347100 -2.19415200

C 1.29180600 -0.68417400 0.15960900

H 2.52531500 -1.47333400 -2.17293900

H 1.29960000 -0.30703900 -2.70578900

H 3.01023000 0.13522600 -2.76687100

H 1.64700000 -1.71175800 0.24345400

H 1.17849200 -0.22901700 1.14263700

I -0.82624500 -1.04421700 -0.45387100

C -1.61919700 0.85596200 0.22947900

C -1.36568200 1.23497200 1.53847900

C -2.33659700 1.61215100 -0.68627200

C -1.85320100 2.48269600 1.94163000

H -0.81165100 0.61084100 2.23016200

C -2.82122900 2.84656400 -0.24948500

H -2.50471900 1.28148100 -1.70296200

C -2.57401400 3.28247400 1.05365900

H -1.66660100 2.81662500 2.95721100

H -3.38297600 3.46630300 -0.94083900

H -2.94685800 4.24802800 1.37957100

O -3.66927800 -1.36873100 -0.82119200

C -4.18624700 -0.99447800 -2.10019500

H -4.60435900 0.01901000 -2.08722600

H -3.36670200 -1.02403400 -2.82434300

H -4.96274900 -1.69318800 -2.43270900

C -5.27714900 0.34497000 0.94447000

H -6.04024200 0.30354000 1.73160300

H -4.45758900 0.97296800 1.30574900

H -5.72895500 0.83939700 0.07751900

C -3.61926300 -2.06506900 1.92758000

C -6.14106700 -2.52040600 0.18762300

H -3.14633300 -3.01309100 1.64790200

H -2.83356700 -1.36046200 2.21898500

H -4.23718000 -2.25106100 2.81427700

H -5.80619100 -3.51934200 -0.11476800

H -6.76057500 -2.63328500 1.08550600

H -6.78392600 -2.12435900 -0.60632500

Si -4.68522100 -1.38982600 0.54310300

Zero-point correction= 0.507567 (Hartree/Particle)

Thermal correction to Energy= 0.549694

Thermal correction to Enthalpy= 0.550764

Thermal correction to Gibbs Free Energy= 0.425293

SCF Done: E(RM062X) = -1801.52916652

**TS2-2**

C 3.67702400 -1.20717400 0.67695600

C 4.76512700 -0.37268600 0.34412600

C 6.05467800 -0.86575900 0.31744900

C 6.24102600 -2.22488200 0.61371400

C 5.16443500 -3.05689000 0.95090600

C 3.86256900 -2.56167100 0.99453900

H 6.89306200 -0.21563700 0.09147900

H 7.24375500 -2.63949300 0.59537700

H 5.34678200 -4.10008800 1.18472300

H 3.02309800 -3.19863200 1.24959600

N 2.94451800 1.01831600 0.48252500

C 2.06650400 2.08696600 0.11782100

C 1.98877300 2.48623000 -1.22252200

C 1.26510500 2.69143200 1.08799200

C 1.08770900 3.48587900 -1.58965500

H 2.62480900 2.01362300 -1.96376900

C 0.35771700 3.68206300 0.70907100

H 1.35210200 2.38917600 2.12534600

C 0.26424600 4.07651800 -0.62681000

H 1.02488600 3.79630600 -2.62798200

H -0.27056500 4.14856000 1.46121300

H -0.44242900 4.84762200 -0.91739300

C 4.29076900 1.01973800 0.16427100

O 4.94341900 1.99325100 -0.19596800

C 2.41118000 -0.32591300 0.74266900

C 1.51457300 -0.48753300 1.94980300

C 2.13568200 -1.06876900 -0.47368700

H 0.58565700 0.06686600 1.80492300

H 1.27117300 -1.54296400 2.08968400

H 2.02543400 -0.11485400 2.83945800

H 2.54415000 -0.74583300 -1.42400800

H 1.62988800 -2.02705700 -0.42545400

I -0.47277700 0.08734700 -1.44325900

C -1.72068500 -1.61813800 -1.00721200

C -1.37776900 -2.45376700 0.05269100

C -2.86001300 -1.82633800 -1.78009100

C -2.20958200 -3.54087100 0.33861700

H -0.49283500 -2.27583900 0.65218500

C -3.68008500 -2.91624300 -1.47539600

H -3.11671400 -1.16100100 -2.59626400

C -3.35708000 -3.77283700 -0.42113700

H -1.95369500 -4.20075400 1.16201800

H -4.57239500 -3.08963800 -2.06916200

H -3.99910900 -4.61730200 -0.19111300

O -4.13034300 0.83357800 -0.17292500

C -5.32912900 0.53467700 -0.88352200

H -6.17718100 1.13450800 -0.52674500

H -5.17016500 0.76706400 -1.94122600

H -5.59371400 -0.52672300 -0.80066800

C -5.08386700 2.04760000 2.29574700

H -5.05235800 1.97347200 3.38965900

H -4.70009800 3.03606600 2.01736100

H -6.13641400 1.99884700 1.99361900

C -2.25719000 0.84024300 1.96374200

C -4.73234700 -1.00278200 2.02219700

H -1.70014900 -0.04580900 1.64734100

H -1.79720900 1.71797000 1.49861800

H -2.14574600 0.93726900 3.05041100

H -4.19574500 -1.81494700 1.52066600

H -4.61138500 -1.14046200 3.10384400

H -5.79905300 -1.11071900 1.79583200

Si -4.06665200 0.67668600 1.50735300

Zero-point correction= 0.505658 (Hartree/Particle)

Thermal correction to Energy= 0.547970

Thermal correction to Enthalpy= 0.549040

Thermal correction to Gibbs Free Energy= 0.422550

SCF Done: E(RM062X) = -1801.50756512

**PhI**

C 2.66388700 1.20831500 -0.00000100

C 3.36257100 0.00000000 0.00000000

C 2.66388700 -1.20831500 0.00000100

C 1.26602200 -1.21924500 0.00000000

C 0.59300200 0.00000000 0.00000000

C 1.26602200 1.21924500 0.00000000

H 3.20090500 2.15221600 -0.00000600

H 4.44826000 0.00000000 0.00000000

H 3.20090500 -2.15221600 0.00000600

H 0.72579500 -2.15907800 -0.00000800

H 0.72579500 2.15907800 0.00000800

I -1.56969800 0.00000000 0.00000000

Zero-point correction= 0.090172 (Hartree/Particle)

Thermal correction to Energy= 0.097531

Thermal correction to Enthalpy= 0.098601

Thermal correction to Gibbs Free Energy= 0.053892

SCF Done: E(RM062X) = -529.251014465

**IM2-6**

C 1.87577700 0.52655300 0.35031200

C 1.77210000 -0.83769700 -0.03275000

C 2.88869200 -1.56587400 -0.37336300

C 4.13277700 -0.91308300 -0.35187300

C 4.24670800 0.44874200 -0.03671100

C 3.12350300 1.19131500 0.29774500

H 2.79895300 -2.59791600 -0.69353400

H 5.02546900 -1.46873000 -0.62060600

H 5.22059800 0.92424000 -0.05460600

H 3.20216000 2.23675700 0.57279100

N -0.39103100 -0.08110200 -0.19990500

C -1.82158500 -0.07821800 -0.10240900

C -2.43570700 -0.62074800 1.02941700

C -2.57940700 0.46800500 -1.13950500

C -3.82658800 -0.61370300 1.11982200

H -1.82693700 -1.04280900 1.82186200

C -3.97033500 0.48002900 -1.03522400

H -2.08206000 0.86892000 -2.01584900

C -4.59329800 -0.06028300 0.09139100

H -4.31037800 -1.03612300 1.99483000

H -4.56519600 0.90460900 -1.83763300

H -5.67621900 -0.05267600 0.16753900

C 0.34977800 -1.23911800 -0.21648500

O -0.08004100 -2.37467400 -0.37397300

C 0.36770700 1.11246100 0.16158200

C 0.19490700 2.34219800 -0.68049900

C 0.83907900 1.11417400 1.51249300

H -0.82699100 2.71165100 -0.55350400

H 0.88669600 3.12107000 -0.35579000

H 0.36273300 2.11086700 -1.73388500

H 0.53948600 0.33105900 2.19955200

H 1.24458900 2.02567400 1.93630300

Zero-point correction= 0.259618 (Hartree/Particle)

Thermal correction to Energy= 0.278171

Thermal correction to Enthalpy= 0.279241

Thermal correction to Gibbs Free Energy= 0.209430

SCF Done: E(RM062X) = -747.843958225

**TS2-3**

C -1.96792700 -0.51428500 0.49754000

C -1.77548600 0.81880800 0.06670300

C -2.83485300 1.56831700 -0.40715600

C -4.09788300 0.96702500 -0.48795700

C -4.28435300 -0.37590200 -0.13571900

C -3.22213000 -1.13554200 0.33886400

H -2.66929900 2.58153200 -0.75615400

H -4.93713000 1.54004700 -0.86823100

H -5.26557800 -0.82730900 -0.23147900

H -3.36428800 -2.16493600 0.64664000

N 0.40628400 0.06255700 -0.11408000

C 1.84367000 0.06113700 -0.07523300

C 2.49283900 0.45699000 1.09648800

C 2.56396600 -0.32823500 -1.20519600

C 3.88633300 0.46483800 1.13186300

H 1.90903600 0.75738700 1.96013400

C 3.95775800 -0.32784900 -1.15561100

H 2.03600500 -0.61927300 -2.10677500

C 4.61757800 0.06887600 0.00946300

H 4.39953000 0.77579000 2.03626800

H 4.52621400 -0.62965900 -2.02948000

H 5.70269700 0.07179600 0.04232900

C -0.34366800 1.21543900 -0.06078700

O 0.09181400 2.35328100 -0.15626100

C -0.30942300 -1.15848300 0.16146400

C -0.21904900 -2.27390000 -0.81527800

C -0.90251900 -1.23006300 1.45541300

H 0.80141600 -2.67358400 -0.75642200

H -0.91588700 -3.07220900 -0.55938100

H -0.38940300 -1.91991400 -1.83363600

H -0.54404200 -0.56103300 2.23169700

H -1.29689100 -2.18581300 1.78398300

Zero-point correction= 0.258658 (Hartree/Particle)

Thermal correction to Energy= 0.276652

Thermal correction to Enthalpy= 0.277722

Thermal correction to Gibbs Free Energy= 0.208620
SCF Done: E(RM062X) = -747.842656598

**IM2-7**

C 2.35445700 0.71700700 -0.00781400

C 1.87783100 -0.60162100 0.00027200

C 2.76991900 -1.68851900 0.01680200

C 4.13516400 -1.45201200 0.02525400

C 4.61431600 -0.13444800 0.01746300

C 3.73257600 0.94226200 0.00094700

H 2.37409200 -2.69761300 0.02289900

H 4.83107500 -2.28410700 0.03790000

H 5.68393100 0.05011200 0.02431700

H 4.10848000 1.96045300 -0.00541900

N -0.45582900 0.28570700 -0.01479600

C -1.87530000 -0.03786900 -0.00349200

C -2.52752300 -0.14953300 1.22058600

C -2.52835300 -0.23594300 -1.21642100

C -3.88786600 -0.45753900 1.22415500

H -1.98034600 0.00330900 2.14463900

C -3.88865200 -0.54260000 -1.19729800

H -1.98180300 -0.14850400 -2.14921700

C -4.56604300 -0.65198000 0.01911200

H -4.41421200 -0.54656000 2.16878200

H -4.41581900 -0.69749000 -2.13295600

H -5.62471500 -0.89115600 0.02787700

C 0.44746600 -0.87729800 -0.00658000

O -0.07183100 -1.97274000 -0.00360800

C -0.04492100 1.53315700 -0.02433000

C -1.02298100 2.64871000 -0.01964100

C 1.39469800 1.86006500 -0.03047300

H -1.57542300 2.63873400 0.92702100

H -0.50979600 3.60321600 -0.12851000

H -1.75580000 2.52059800 -0.82119800

H 1.55988900 2.54156200 0.81767800

H 1.55717600 2.49838400 -0.91261800

Zero-point correction= 0.260425 (Hartree/Particle)

Thermal correction to Energy= 0.278870

Thermal correction to Enthalpy= 0.279940

Thermal correction to Gibbs Free Energy= 0.209895

SCF Done: E(RM062X) = -747.904157596

**MeOH-TMS**

C -2.37459300 -0.11748600 -0.13752000

H -2.32083900 0.96572800 -0.05202500

H -3.10047500 -0.40769100 -0.89431900

H -2.58011800 -0.58757900 0.82336600

O -1.05551100 -0.56433400 -0.62009300

C 1.70642900 -1.21464300 -0.56463000

H 2.72652800 -0.94037900 -0.27096200

H 1.49237300 -2.20213800 -0.14234100

H 1.68058500 -1.28443400 -1.65703900

C 0.26515600 0.08141600 1.90394600

C 0.65962800 1.74455400 -0.71905700

H 1.19204500 0.40376600 2.39348600

H -0.52635600 0.77656700 2.20116500

H 0.01647400 -0.91651300 2.27960700

H -0.18116100 2.39061200 -0.44695000

H 1.57841400 2.23318100 -0.37370400

H 0.70466200 1.66834300 -1.81011000

Si 0.52105700 0.06816200 0.06439400

H -1.07255600 -1.50211200 -0.88738100

Zero-point correction= 0.166363 (Hartree/Particle)

Thermal correction to Energy= 0.179636

Thermal correction to Enthalpy= 0.180706

Thermal correction to Gibbs Free Energy= 0.124986

SCF Done: E(RM062X) = -524.821737143

**2a**

C 2.32277600 0.76217100 -0.00004300

C 1.86727800 -0.57873700 0.00008400

C 2.78832300 -1.64203900 0.00019900

C 4.14834200 -1.38500100 0.00019100

C 4.60932400 -0.05434200 0.00004900

C 3.71514500 1.00205300 -0.00007300

H 2.41030600 -2.65852200 0.00028100

H 4.85911600 -2.20531200 0.00027900

H 5.67712300 0.14423600 0.00003100

H 4.07253900 2.02757600 -0.00019100

N -0.41669500 0.23186000 -0.00007800

C -1.83259500 -0.05613000 -0.00001600

C -2.50504500 -0.19221700 1.21336700

C -2.50496600 -0.19324900 -1.21332900

C -3.87417100 -0.46053200 1.20996200

H -1.95736700 -0.08586200 2.14413400

C -3.87409300 -0.46155700 -1.20978000

H -1.95722800 -0.08767100 -2.14414900

C -4.55886000 -0.59400800 0.00012600

H -4.40394700 -0.56519000 2.15168400

H -4.40381500 -0.56701200 -2.15144400

H -5.62448400 -0.80195600 0.00018100

C 0.43624500 -0.87241400 0.00003000

O -0.03432800 -2.02200500 0.00010000

C 0.02633600 1.55829200 -0.00018100

C -1.01272100 2.63724000 -0.00036400

C 1.35959100 1.82159700 -0.00014500

H -1.65932200 2.57093200 0.88055300

H -0.52302800 3.61242000 -0.00039100

H -1.65913200 2.57078400 -0.88140500

H 1.69527100 2.85183500 -0.00028500

Zero-point correction= 0.249099 (Hartree/Particle)

Thermal correction to Energy= 0.266909

Thermal correction to Enthalpy= 0.267979

Thermal correction to Gibbs Free Energy= 0.199905

SCF Done: E(RM062X) = -747.488309466

**Reagent 2**

C 0.62013100 -1.90038100 -1.96178800

C 0.47255800 -3.22812000 -1.55809500

C -0.66582600 -3.62921000 -0.85715800

C -1.66493800 -2.70296800 -0.53850700

C -1.46317900 -1.39660000 -0.94841000

C -0.36419700 -0.95423300 -1.66218600

H 1.50155600 -1.58402600 -2.50873500

H 1.24415800 -3.95325400 -1.79500800

H -0.78697300 -4.66215900 -0.54693000

H -2.56021800 -3.00188500 -0.00829600

H -0.26165700 0.07963400 -1.95824400

I -3.00997300 0.06090200 -0.40329300

O -4.38767900 -1.46243200 -0.23709400

H -4.43865000 -1.67707900 0.71189900

O -1.38451900 1.68883400 -0.71902800

S -0.57241400 2.18992300 0.52531800

O -1.39322200 2.05867500 1.74299900

O -0.07125100 3.54745000 0.25438600

C 0.84063800 1.05214300 0.57293500

C 0.91460300 -0.00627500 1.51181000

C 1.84335600 1.22139500 -0.41766300

C 2.05738300 -0.81651600 1.48304400

C 2.95516700 0.37810900 -0.37750300

C 3.09042800 -0.63803700 0.56826700

H 2.14221700 -1.62382700 2.20136200

H 3.73413900 0.51266400 -1.11934800

C 4.29999600 -1.55521500 0.57068400

H 4.20736600 -2.21095000 1.44494600

C -0.13803700 -0.33244100 2.57240900

H -1.10410300 0.01444400 2.21586300

C 1.80282300 2.25562800 -1.54500700

H 0.79014700 2.63983900 -1.63996700

C -0.27677000 -1.83770700 2.84979000

H -1.17196400 -2.00783900 3.45781200

H 0.57138700 -2.24477500 3.40964100

H -0.38083500 -2.41177300 1.92472000

C 0.16975500 0.42732700 3.87437200

H 1.13767800 0.11503000 4.28334900

H -0.59876700 0.22187900 4.62817200

H 0.19692600 1.50532400 3.70174900

C 2.71874000 3.44384400 -1.20832700

H 3.76177000 3.11917400 -1.11869900

H 2.41699400 3.90881400 -0.26721300

H 2.66752800 4.20181600 -1.99802400

C 2.17601700 1.65339100 -2.91023400

H 2.00865600 2.40081000 -3.69311900

H 1.56594500 0.77685300 -3.14907600

H 3.22817100 1.35716400 -2.96546200

C 5.61372100 -0.77000700 0.70438600

H 6.46510100 -1.45650300 0.76503400

H 5.61337000 -0.14616600 1.60409000

H 5.77518000 -0.11535700 -0.15886500

C 4.31362900 -2.44389700 -0.68466100

H 5.16180200 -3.13677600 -0.65790300

H 4.40405100 -1.83770700 -1.59290800

H 3.39358500 -3.03156100 -0.76047400

Zero-point correction= 0.463654 (Hartree/Particle)

Thermal correction to Energy= 0.504455

Thermal correction to Enthalpy= 0.505573

Thermal correction to Gibbs Free Energy= 0.384944

SCF Done: E(RM062X) = -1814.24338409

**LiClO_4_**

Li 2.33092000 -0.00000600 0.00007000

Cl -0.18555200 0.00000100 -0.00000600

O -1.00933800 1.22312500 -0.00014100

O 0.76968700 -0.00024900 -1.19623700

O 0.76961300 -0.00003600 1.19628400

O -1.00975800 -1.22284000 0.00007900

Zero-point correction= 0.016768 (Hartree/Particle)

Thermal correction to Energy= 0.022238

Thermal correction to Enthalpy= 0.023182

Thermal correction to Gibbs Free Energy= -0.012235

SCF Done: E(RM062X) = -768.409774717

**1a(DCE)**

C -3.18002300 -0.91734800 -1.26929300

C -2.87275000 -1.93355100 -0.36342200

C -1.91188200 -1.72192300 0.62254900

C -1.22751200 -0.50050600 0.73124800

C -1.51737500 0.50493800 -0.21654200

C -2.50726200 0.29869100 -1.18455500

H -3.93297100 -1.07079100 -2.03586200

H -3.38769000 -2.88792100 -0.41700900

H -1.70327500 -2.51098000 1.33700000

H -2.72533600 1.09579900 -1.88802900

C -0.24557900 -0.30061600 1.83224500

C -0.22700300 0.83489600 2.54353000

H 0.49708300 0.98580800 3.33922200

H -0.93417300 1.63843700 2.36425500

C -0.82507600 1.83883700 -0.25518500

O -1.46463400 2.88591900 -0.15407600

N 0.53140700 1.87086900 -0.44983400

H 0.93343300 2.79000800 -0.30213600

C 1.44294200 0.80343100 -0.61837200

C 1.17753600 -0.26208000 -1.48708000

C 2.65429600 0.84215800 0.08342200

C 2.10628000 -1.29332800 -1.62104400

H 0.25529900 -0.27950400 -2.05468400

C 3.58598500 -0.18206400 -0.07285100

H 2.85051200 1.67047800 0.75751400

C 3.31236300 -1.26026100 -0.91762200

H 1.88963900 -2.11849900 -2.29262300

H 4.52141600 -0.14257800 0.47687900

H 4.03438600 -2.06258100 -1.03209600

C 0.70806900 -1.43359600 2.11861300

H 0.18026300 -2.31510500 2.50117800

H 1.23125800 -1.74372900 1.20881600

H 1.44931600 -1.13833900 2.86517700

Zero-point correction= 0.269975 (Hartree/Particle)

Thermal correction to Energy= 0.291374

Thermal correction to Enthalpy= 0.292492

Thermal correction to Gibbs Free Energy= 0.215765

SCF Done: E(RM062X) = -748.663719290

**IM3-1**

C 1.50111100 -1.13434400 -2.58363900

C 1.93991600 -2.41416000 -2.23925400

C 1.27135600 -3.15431500 -1.26217900

C 0.16061400 -2.61821900 -0.60789200

C -0.25249200 -1.34517800 -0.98560500

C 0.38410500 -0.58038400 -1.95704000

H 2.02350400 -0.55818000 -3.33913400

H 2.81030800 -2.83555800 -2.73155700

H 1.61607800 -4.14677900 -0.99245800

H -0.34571100 -3.17511100 0.17098400

H 0.03643800 0.41066600 -2.22030100

I -1.97089100 -0.51665600 -0.02148400

O -3.17875000 -1.74828300 -1.29231300

H -3.24775100 -2.62526300 -0.87746600

O -0.61181800 0.83818100 1.10705100

S 0.52304800 0.27854400 2.05469700

O 0.19554500 -1.12133400 2.37548600

O 0.67969100 1.21129600 3.18186900

C 2.00205300 0.38293700 1.01504400

C 2.93401000 -0.68365100 0.94264900

C 2.20331200 1.58881500 0.30220000

C 4.00702600 -0.53320700 0.05664600

C 3.28660400 1.65475100 -0.57860200

C 4.19323900 0.60676500 -0.72425100

H 4.73254300 -1.33271300 -0.02785800

H 3.43339300 2.56598400 -1.14849100

C 5.36332000 0.70440400 -1.68743200

H 5.98216400 -0.18830900 -1.53609500

C 2.88629000 -1.92767500 1.82967200

H 1.86290500 -2.29874900 1.83018800

C 1.35892200 2.85582200 0.45005300

H 0.63126400 2.71703400 1.24486100

C 3.79157200 -3.07335200 1.36220000

H 3.59212300 -3.95751500 1.97629900

H 4.85501400 -2.83530200 1.47296300

H 3.60885900 -3.34215800 0.31802200

C 3.24918300 -1.54539900 3.27886100

H 4.28248000 -1.18331200 3.32964900

H 3.16242500 -2.41830500 3.93477000

H 2.60018600 -0.76207400 3.67664900

C 2.23988400 4.04561900 0.86993000

H 2.96586000 4.31679500 0.09679700

H 2.79107800 3.81928000 1.78829500

H 1.61132500 4.92254900 1.05868800

C 0.57903700 3.16650300 -0.83563400

H -0.00467700 4.08513600 -0.71157900

H -0.11556400 2.35713900 -1.07361900

H 1.24965300 3.30843900 -1.69014800

C 6.23586900 1.93693700 -1.40247500

H 7.11241800 1.94396800 -2.05921300

H 6.58592100 1.94390300 -0.36521100

H 5.68231400 2.86578100 -1.57719700

C 4.87946900 0.69982100 -3.14688400

H 5.72941900 0.76239800 -3.83518800

H 4.22215300 1.55381100 -3.34503700

H 4.32406000 -0.21480100 -3.37569700

Li -4.77657000 -0.98365200 -1.86313600

Cl -5.63402000 0.73783200 -0.21487800

O -4.76922200 0.97653400 -1.44767600

O -6.76245100 1.68304500 -0.17248900

O -6.12657400 -0.69187300 -0.38710400

O -4.78642500 0.83245800 1.00213800

Zero-point correction= 0.482075 (Hartree/Particle)

Thermal correction to Energy= 0.532213

Thermal correction to Enthalpy= 0.533332

Thermal correction to Gibbs Free Energy= 0.388195

SCF Done: E(RM062X) = -2582.68278507

**ArSO_3_^-^**

O -2.97840400 -0.28474400 1.16043100

S -2.48028000 0.51389500 0.00256100

O -2.65586900 1.97694600 0.22432700

C -0.68348000 0.14133500 -0.06103800

C 0.32202300 1.13839600 -0.10741200

C -0.31823200 -1.22668200 -0.05252000

C 1.66190400 0.73561600 -0.02947500

C 1.03813500 -1.56359500 0.01784200

C 2.04506300 -0.60223900 0.05185400

H 2.43267700 1.49850800 -0.03827400

H 1.31831700 -2.61275700 0.04743300

C 3.50805200 -1.00139300 0.14377300

H 3.54129000 -2.09615600 0.20967600

C 0.05834600 2.64021900 -0.22434600

H -0.94275800 2.77262400 -0.62650300

C -1.31421700 -2.38532300 -0.12145700

H -2.30447700 -1.98316500 -0.31385800

C 0.07398200 3.29604300 1.16570100

H -0.13206400 4.37038200 1.08950300

H 1.05123600 3.17395400 1.64860400

H -0.68959900 2.84990200 1.80713700

C 1.03970600 3.34989100 -1.17096300

H 2.05555500 3.41035200 -0.76583000

H 0.70149800 4.37796100 -1.34236100

H 1.09481000 2.84809700 -2.14292300

C -0.98806700 -3.33805800 -1.28317300

H -0.02820700 -3.84874800 -1.15130100

H -0.95424000 -2.79723700 -2.23489200

H -1.76226800 -4.10980200 -1.36197000

C -1.37440400 -3.13385000 1.21772000

H -2.10738400 -3.94790900 1.17092200

H -1.67435000 -2.45018400 2.01594300

H -0.40433500 -3.57250100 1.47925400

C 4.17036000 -0.43405900 1.40909500

H 5.20853900 -0.77521200 1.48936100

H 3.63645700 -0.75025500 2.31107500

H 4.17932300 0.66128900 1.39209100

C 4.28478700 -0.58331100 -1.11523400

H 5.32445100 -0.92525900 -1.06128700

H 4.29646100 0.50648800 -1.22659300

H 3.83226600 -1.00699300 -2.01769800

O -3.00728500 0.05609400 -1.31734200

Zero-point correction= 0.357413 (Hartree/Particle)

Thermal correction to Energy= 0.385948

Thermal correction to Enthalpy= 0.387066

Thermal correction to Gibbs Free Energy= 0.293854

SCF Done: E(RM062X) = -1209.38707533

**IM3-2**

C 1.15549000 2.74978000 1.67650800

C 1.56107100 3.22506300 0.42601900

C 1.76309500 2.34600000 -0.63794800

C 1.56211400 0.97437700 -0.46959200

C 1.15993700 0.54592400 0.78927800

C 0.94721700 1.38569600 1.87932600

H 0.99023900 3.43730400 2.49921300

H 1.71797700 4.28884100 0.28134500

H 2.08042100 2.71302300 -1.60700000

H 1.72490500 0.28773600 -1.28891600

H 0.62249200 1.00876600 2.84121700

I 0.91514600 -1.56699900 1.10616000

O 2.87854100 -1.55109300 2.11078200

C -2.23593000 3.32380200 1.17208500

C -2.47346000 2.43564800 2.22114400

C -2.43113900 1.06677900 1.98877200

C -2.15082500 0.55024600 0.70494600

C -1.97194100 1.46730800 -0.36818200

C -1.99121900 2.83612200 -0.11346700

H -2.25269500 4.39446400 1.34719900

H -2.67332900 2.80663100 3.22061600

H -2.56786200 0.38685600 2.82048200

H -1.84388100 3.52285200 -0.93934000

C -2.02077000 -0.89139500 0.54306800

C -1.11002100 -1.44873500 -0.36687400

H -1.20283700 -2.50712000 -0.59578900

H -0.64191100 -0.84262000 -1.13280300

C -1.84818500 1.08145900 -1.81981800

O -0.92438000 1.51733500 -2.50475000

N -2.80656300 0.26645000 -2.35611400

H -2.59403500 -0.03937700 -3.30027000

C -3.95504100 -0.29806700 -1.74182000

C -4.82911300 0.47694300 -0.97083500

C -4.20919100 -1.66231300 -1.93022700

C -5.92591400 -0.12630400 -0.35718600

H -4.64815500 1.53886300 -0.85067300

C -5.32378100 -2.25172000 -1.33515900

H -3.52721900 -2.25155000 -2.53567100

C -6.17801600 -1.48986000 -0.53498500

H -6.59509900 0.47718100 0.24825700

H -5.51584700 -3.30924500 -1.48764200

H -7.03877300 -1.95136300 -0.06150200

C -2.81752200 -1.80693500 1.41321600

H -2.56756500 -1.67664000 2.47196200

H -3.88071000 -1.56487800 1.30753900

H -2.65312500 -2.85073700 1.14342700

H 2.74096600 -1.20914800 3.01087100

Li 4.15723200 -0.84112800 0.99188900

Cl 4.97973800 -0.18629700 -1.30776200

O 3.95444500 -1.25893200 -0.94154800

O 4.36200000 0.83141500 -2.18140100

O 6.16400900 -0.80333700 -1.93528300

O 5.35049000 0.42429800 0.04226000

Zero-point correction= 0.393952 (Hartree/Particle)

Thermal correction to Energy= 0.437331

Thermal correction to Enthalpy= 0.438449

Thermal correction to Gibbs Free Energy= 0.307219

SCF Done: E(RM062X) = -2121.91708844

**TS3-1**

C 1.48828500 1.60610100 1.42817700

C 2.26604700 0.44231400 1.62551700

C 2.07872200 -0.32800600 2.77241800

C 1.12778200 0.04860900 3.72446100

C 0.38604600 1.21499800 3.54939100

C 0.57302300 2.00108000 2.41191900

H 2.70550700 -1.19209200 2.96678300

H 0.99532300 -0.55329600 4.61711200

H -0.33918700 1.51914100 4.29648700

H -0.02596000 2.89402500 2.26856600

N 3.88603500 -1.00260600 0.53002700

C 4.96111300 -1.45453400 -0.26813100

C 5.70109800 -0.62009400 -1.11779000

C 5.27702000 -2.81889600 -0.17720200

C 6.74728300 -1.16513400 -1.86369400

H 5.45779200 0.42970100 -1.18933200

C 6.32193600 -3.34626000 -0.92964800

H 4.70142900 -3.45853200 0.48709600

C 7.06441500 -2.52129200 -1.77836600

H 7.31827600 -0.51451300 -2.51934500

H 6.55576500 -4.40344400 -0.84994500

H 7.88023800 -2.93169500 -2.36489000

C 3.37847000 0.24612300 0.64483600

O 3.76809000 1.22665900 -0.01268500

C 1.60079500 2.37063900 0.17392000

C 1.84175500 3.83261900 0.24743500

C 1.28261000 1.76240300 -1.06405300

H 1.73699600 4.32184300 -0.72219100

H 1.17862800 4.30154300 0.98390600

H 2.86139300 3.98175000 0.62428600

H 1.56774800 2.30287700 -1.96340300

H 1.33507100 0.68087200 -1.13362000

I -1.14349600 2.00459700 -1.15880900

C -1.27279100 0.11649200 -0.12116800

C -0.82893800 -1.01818800 -0.79183300

C -1.83338400 0.10456400 1.15236600

C -0.94840700 -2.24048400 -0.12649500

H -0.41484500 -0.96935200 -1.79206600

C -1.94640000 -1.13512000 1.78649100

H -2.17257400 1.00958000 1.64055800

C -1.50399900 -2.29723900 1.15278300

H -0.61897600 -3.14593000 -0.62515100

H -2.38134400 -1.17996900 2.77901400

H -1.60314700 -3.25333400 1.65467500

O -3.38168200 1.84847500 -1.28779100

H 3.43140500 -1.72859700 1.06955400

Li -4.08926600 0.19846000 -0.88421900

Cl -5.25014900 -1.94886800 -0.26225800

O -4.70231900 -2.99089600 0.62853600

O -6.59254700 -2.30738500 -0.76361800

O -5.29980400 -0.60524200 0.46105100

O -4.30268800 -1.69449400 -1.43150500

H -3.73346400 2.57829700 -0.75025300

Zero-point correction= 0.394296 (Hartree/Particle)

Thermal correction to Energy= 0.436680

Thermal correction to Enthalpy= 0.437798

Thermal correction to Gibbs Free Energy= 0.308019

SCF Done: E(RM062X) = -2121.91268306

**TS3-1'**

C 1.63167300 1.60113200 -0.88528300

C 2.11697200 2.31443800 0.23015600

C 1.61309200 3.57471600 0.54767200

C 0.64680500 4.14552500 -0.27926700

C 0.20740600 3.46876800 -1.42216800

C 0.70376100 2.20460900 -1.74064500

H 2.00278800 4.10689300 1.40886300

H 0.24969900 5.12814200 -0.04792900

H -0.53607900 3.92535200 -2.06637100

H 0.33133600 1.67272800 -2.60839400

N 3.98615600 0.84216500 0.09566500

C 4.88594300 -0.14903600 0.53212200

C 4.65098900 -0.88287700 1.70483700

C 5.97696000 -0.45802200 -0.29297500

C 5.52666900 -1.91092300 2.04814800

H 3.80941600 -0.64409200 2.34118300

C 6.84109100 -1.48877500 0.06273100

H 6.13998000 0.11479700 -1.20118500

C 6.62024800 -2.21778800 1.23483500

H 5.35041500 -2.47496000 2.95855900

H 7.68801800 -1.72182300 -0.57464800

H 7.29664200 -3.01973000 1.51242300

C 3.23144300 1.68603200 0.97105300

O 3.46305000 1.80168900 2.15762900

C 2.09256000 0.21090000 -1.07248400

C 2.60553300 -0.19430600 -2.40939900

C 1.73460300 -0.79362700 -0.11110000

H 3.05806300 -1.18728300 -2.38808900

H 1.76248500 -0.21259600 -3.11554600

H 3.31174100 0.54453000 -2.79723600

H 2.36078700 -1.68554500 -0.13166000

H 1.51762100 -0.44378900 0.89498800

I -0.33382500 -1.79990000 -0.69045100

C -1.37408000 0.07233900 -0.35139600

C -1.21045000 0.70646900 0.87206300

C -2.19634000 0.54072800 -1.37153700

C -1.91819700 1.89456400 1.07495400

H -0.57251800 0.30729200 1.65134300

C -2.90716100 1.71884200 -1.12945500

H -2.29819500 0.01725800 -2.31505200

C -2.76497700 2.39272700 0.08453100

H -1.81183100 2.41469900 2.02090300

H -3.56625500 2.10438600 -1.90006200

H -3.32070000 3.30705400 0.26113600

O -2.44537600 -2.62625900 -1.00931700

H 4.26913200 1.30739700 -0.76416300

H -2.55445800 -2.74269800 -1.96678000

Li -3.76360900 -1.68737900 -0.16178200

Cl -5.61520700 -0.39444500 0.96074400

O -5.68256000 1.07658400 1.06929500

O -4.32167100 -0.91566200 1.57973000

O -6.78056500 -1.04375500 1.59592100

O -5.51242100 -0.81376900 -0.50321800

Zero-point correction= 0.393898 (Hartree/Particle)

Thermal correction to Energy= 0.436097

Thermal correction to Enthalpy= 0.437216

Thermal correction to Gibbs Free Energy= 0.308497

SCF Done: E(RM062X) = -2121.90177842

**IM3-3**

C 2.64628800 2.45335900 -0.60860700

C 3.86499200 1.76196100 -0.56672700

C 5.09485100 2.42208600 -0.63700000

C 5.07007300 3.80882900 -0.74672400

C 3.85047500 4.50435200 -0.78432200

C 2.62359100 3.83674400 -0.71618700

H 6.03143700 1.87475200 -0.61347200

H 6.00209800 4.36006900 -0.80577900

H 3.86096800 5.58594500 -0.87135500

H 1.68813300 4.38422100 -0.74781800

N 4.37189100 -0.64764500 -0.32684000

C 4.09971800 -2.03610000 -0.16051700

C 2.88023000 -2.62310300 -0.51993600

C 5.13922500 -2.80857200 0.37023400

C 2.70861200 -3.99213100 -0.31684700

H 2.08717700 -2.04009400 -0.96565100

C 4.95083000 -4.17397900 0.56362300

H 6.08141100 -2.33673000 0.63354000

C 3.73317000 -4.76920700 0.22642200

H 1.76585300 -4.45113900 -0.59686500

H 5.75651900 -4.77031600 0.97902400

H 3.58613100 -5.83335000 0.37972600

C 3.53817900 0.35952500 -0.44593000

O 2.23635700 0.16053500 -0.44318400

C 1.50635100 1.47080700 -0.52375200

C 0.64278100 1.42818800 -1.77359500

C 0.75084500 1.60005900 0.78787900

H -0.08412700 0.61457200 -1.72655800

H 0.10861400 2.37747900 -1.86393500

H 1.26784300 1.29238800 -2.65836800

H 1.42753800 1.70592500 1.63623200

H 0.04479100 2.42959100 0.75924700

I -0.45388800 -0.24783600 1.21722800

C -2.28346200 0.90205000 1.30981600

C -2.98871100 0.89449900 2.50857400

C -2.70960800 1.56381600 0.16460000

C -4.19236100 1.60381800 2.55118400

H -2.63114000 0.35218100 3.37476300

C -3.91651500 2.26706900 0.23775300

H -2.15418000 1.52501600 -0.76310600

C -4.65181900 2.28708300 1.42279000

H -4.76444800 1.61775700 3.47325400

H -4.28132200 2.77758500 -0.64656700

H -5.58973500 2.83100300 1.46619800

O -2.14953700 -1.95629700 1.67017300

H 5.35556100 -0.38958600 -0.28251600

Li -3.37292900 -1.59372200 0.41273900

Cl -4.40472800 -1.03013500 -1.84821400

O -4.60828200 0.33669700 -2.37410400

O -4.89013100 -2.05490500 -2.79674600

O -2.93247200 -1.25930400 -1.53227600

O -5.10079300 -1.19021700 -0.50100100

H -1.69466600 -2.80396700 1.55694600

Zero-point correction= 0.396770 (Hartree/Particle)

Thermal correction to Energy= 0.439091

Thermal correction to Enthalpy= 0.440209

Thermal correction to Gibbs Free Energy= 0.308708

SCF Done: E(RM062X) = -2121.94757255

**IM3-4**

C 1.21176300 2.23782200 -1.70224700

C -0.01212500 1.60428900 -1.95814700

C -0.16264700 0.67117300 -2.98907800

C 0.95528900 0.40041800 -3.77486800

C 2.17002500 1.06265600 -3.55143700

C 2.31187500 1.99184800 -2.51448100

H -1.11393800 0.18578900 -3.17214700

H 0.87701600 -0.32033800 -4.58175300

H 3.01898600 0.85309500 -4.19429700

H 3.25472800 2.50497300 -2.36532800

N -2.21663600 1.79662200 -0.85038300

C -3.15135200 2.23256300 0.12685600

C -2.79156000 2.87000200 1.32237700

C -4.49335300 1.93282100 -0.14137300

C -3.78980000 3.21310300 2.23320300

H -1.75839700 3.09185300 1.54509500

C -5.47635800 2.28052200 0.77929000

H -4.74743200 1.42292200 -1.06156700

C -5.13055500 2.92647500 1.96828400

H -3.51161000 3.70584900 3.15957000

H -6.51302300 2.04222100 0.56538300

H -5.89829300 3.19982300 2.68511600

C -0.96575800 2.13974500 -1.00131800

O -0.40633700 3.06952100 -0.24076000

C 1.06485000 3.15460000 -0.51238200

C 1.39489100 4.60896300 -0.79834600

C 1.65876500 2.63600700 0.79649900

H 1.13950600 5.23344300 0.06112600

H 2.46709500 4.70627100 -0.99658800

H 0.85257400 4.95753600 -1.67977000

H 1.51194100 3.36238400 1.59652000

H 1.21873600 1.67961800 1.07233800

I 3.85506100 2.20737600 0.77656700

C 3.39142700 0.09747700 0.85346200

C 3.20958300 -0.47590400 2.10811800

C 3.29965900 -0.61068600 -0.34098600

C 2.91317000 -1.84198900 2.15525900

H 3.29582800 0.10366500 3.01975200

C 3.00620200 -1.97522600 -0.26109700

H 3.45229400 -0.13548700 -1.30086100

C 2.81450400 -2.58598600 0.97926800

H 2.76821300 -2.31722300 3.11994300

H 2.93839600 -2.55336600 -1.17582700

H 2.59366100 -3.64652300 1.02777200

O 6.09761100 1.25606000 0.81417200

H -2.59668800 1.07962100 -1.54093500

O -2.19227000 -2.10062000 -2.66030900

S -3.46281400 -1.36964200 -2.43353300

O -4.56633500 -1.85023600 -3.29296200

O -3.27943200 0.13640000 -2.58566600

C -3.91659100 -1.53406000 -0.66685300

C -5.27474300 -1.61835200 -0.26266600

C -2.88334400 -1.42790700 0.30585700

C -5.55503700 -1.57121200 1.11020400

C -3.24133600 -1.40346500 1.65628400

C -4.56659500 -1.46152400 2.08283900

H -6.58709800 -1.62534100 1.43609100

H -2.45647400 -1.33493700 2.40015200

C -4.91982700 -1.43240400 3.55936200

H -6.01452700 -1.43251800 3.63107100

C -6.46884800 -1.73367800 -1.21541000

H -6.15190800 -2.33288700 -2.06548800

C -1.38536300 -1.40936800 -0.00535100

H -1.23517900 -0.97888700 -0.99228100

C -7.69287300 -2.42616800 -0.59810600

H -8.42193400 -2.63151600 -1.38919400

H -8.19908900 -1.80686000 0.15037300

H -7.42966300 -3.37984900 -0.12877200

C -6.87044200 -0.35108800 -1.75939800

H -7.71365700 -0.44631300 -2.45305800

H -6.04626800 0.11815800 -2.29963500

H -7.18023100 0.31219000 -0.94345300

C -0.54994500 -0.58523200 0.98859700

H -0.42730700 -1.09250600 1.95054600

H -0.98955700 0.39646700 1.18681600

H 0.45272000 -0.44223500 0.57556900

C -0.85038100 -2.85201500 -0.05820200

H 0.20648300 -2.85522600 -0.34405600

H -1.40471500 -3.44286700 -0.78841800

H -0.93690900 -3.32935800 0.92503200

C -4.40185500 -0.15768100 4.24314400

H -4.70678400 -0.13496800 5.29524400

H -4.78999100 0.73933600 3.75244600

H -3.30783100 -0.10824300 4.21164300

C -4.40031100 -2.69035500 4.27538800

H -4.70477000 -2.68998800 5.32787900

H -3.30603000 -2.73622100 4.24255000

H -4.78826900 -3.60108200 3.80759400

Li 6.15843400 -0.51047400 0.54461200

Cl 6.66873600 -2.92652100 -0.05321300

O 5.66198200 -3.96000200 -0.37494200

O 8.04294600 -3.47031900 -0.11708700

O 6.52980400 -1.73976800 -0.99832800

O 6.40344100 -2.33591600 1.32578300

H 6.61154000 1.77074600 0.17528000

Zero-point correction= 0.755882 (Hartree/Particle)

Thermal correction to Energy= 0.828235

Thermal correction to Enthalpy= 0.829354

Thermal correction to Gibbs Free Energy= 0.634766

SCF Done: E(RM062X) = -3331.36902140

**TS3-2**

C 1.30115500 1.89136100 -1.92014700

C 0.12021900 1.16590800 -2.11443000

C 0.07392400 0.06141000 -2.96948000

C 1.24253100 -0.28311700 -3.64718800

C 2.41278200 0.47178700 -3.49300500

C 2.45456700 1.57023600 -2.62718700

H -0.83619800 -0.50657800 -3.11006900

H 1.23851000 -1.13646200 -4.31717000

H 3.30286300 0.20122700 -4.05215200

H 3.36472900 2.14964000 -2.52287800

N -2.16006200 1.46083800 -1.20362700

C -3.11259500 2.10564800 -0.37296200

C -2.79533700 2.73626700 0.84142000

C -4.45227000 2.02657100 -0.77930500

C -3.81071000 3.29107400 1.61786800

H -1.76938200 2.78824500 1.17867100

C -5.45845000 2.58851800 0.00237300

H -4.69323800 1.52668500 -1.70880400

C -5.14325500 3.22654600 1.20340100

H -3.55627200 3.77393200 2.55653000

H -6.48998600 2.52414400 -0.32955100

H -5.92706500 3.66430600 1.81369500

C -0.92447400 1.81739700 -1.32405500

O -0.41796900 2.91066500 -0.72430700

C 1.05071200 2.98395000 -0.90838100

C 1.39396400 4.38192200 -1.39391500

C 1.57307300 2.68641800 0.49966100

H 1.06762800 5.12759000 -0.66457200

H 2.47677000 4.47218900 -1.52669600

H 0.91516900 4.57942700 -2.35544800

H 1.37884400 3.52519100 1.16919100

H 1.12355700 1.77816900 0.89694800

I 3.76730000 2.29410000 0.66450400

C 3.34167600 0.20915500 1.05003300

C 3.22597200 -0.18574700 2.37931900

C 3.20805300 -0.65994600 -0.02823300

C 2.95520000 -1.53454000 2.63067800

H 3.34225500 0.51772000 3.19514400

C 2.94052400 -2.00312700 0.25469900

H 3.30939700 -0.32514300 -1.05160100

C 2.81520900 -2.43683300 1.57520100

H 2.85997300 -1.87110300 3.65791000

H 2.84192000 -2.70322400 -0.56701300

H 2.61274400 -3.48231400 1.78126800

O 6.02597300 1.37896900 0.95863500

H -2.66254000 0.42829700 -1.95140900

O -2.01838200 -2.48521000 -2.18207800

S -3.32568000 -1.80625000 -2.14791500

O -4.36490600 -2.42883300 -2.97834000

O -3.17495900 -0.31753500 -2.64025100

C -3.86904900 -1.59940100 -0.42422500

C -5.25455600 -1.57907400 -0.10829500

C -2.88525900 -1.34251000 0.57247000

C -5.61087200 -1.28178400 1.21288100

C -3.32756400 -1.06189200 1.86747600

C -4.67720000 -1.01922800 2.21031300

H -6.66067700 -1.25966500 1.47836300

H -2.58923100 -0.87563200 2.63734000

C -5.11605000 -0.73727400 3.63611900

H -6.21267900 -0.71810200 3.64038900

C -6.39490000 -1.84461300 -1.09700400

H -6.05882300 -2.61131100 -1.79058000

C -1.37024600 -1.42878700 0.37048000

H -1.13123000 -1.17169100 -0.65758900

C -7.67877400 -2.36853500 -0.43643200

H -8.36692700 -2.70388200 -1.21934100

H -8.20188800 -1.59975100 0.14233400

H -7.48173000 -3.21907400 0.22419300

C -6.71213300 -0.58390800 -1.92123300

H -7.52060400 -0.79187100 -2.63089000

H -5.84479300 -0.24716500 -2.49112600

H -7.03761600 0.23224600 -1.26685800

C -0.56306900 -0.47693600 1.26715100

H -0.53829100 -0.80409600 2.31140900

H -0.95586400 0.54261000 1.24374500

H 0.47180000 -0.45834600 0.91528400

C -0.90476800 -2.87845700 0.60193700

H 0.16752800 -2.96800400 0.40134400

H -1.43493800 -3.57055900 -0.05397300

H -1.08139600 -3.17467800 1.64249800

C -4.61773300 0.63036700 4.12663300

H -4.96922400 0.82211000 5.14636400

H -4.97787000 1.43515400 3.48040900

H -3.52302900 0.67362100 4.13632900

C -4.66057300 -1.86197700 4.58124200

H -5.03128200 -1.68479900 5.59675400

H -3.56734000 -1.91756100 4.62789000

H -5.03234500 -2.83589400 4.24660200

Li 6.10991700 -0.38082000 0.68096900

Cl 6.62656700 -2.77341100 -0.00478300

O 5.62135800 -3.82985900 -0.24805600

O 8.00258800 -3.27883500 -0.20305200

O 6.36961100 -1.57920800 -0.91426700

O 6.47486800 -2.20916100 1.40223700

H 6.59797300 1.90268200 0.37939100

Zero-point correction= 0.751138 (Hartree/Particle)

Thermal correction to Energy= 0.823102

Thermal correction to Enthalpy= 0.824220

Thermal correction to Gibbs Free Energy= 0.630672

SCF Done: E(RM062X) = -3331.36254359

**IM3-5**

C 1.30734600 1.89009800 -1.92952500

C 0.12560400 1.16651700 -2.12113800

C 0.07817100 0.06028200 -2.97315500

C 1.24608000 -0.28916000 -3.64997500

C 2.41795500 0.46356100 -3.49739800

C 2.46082100 1.56462400 -2.63493500

H -0.83236200 -0.50695300 -3.11387700

H 1.24035000 -1.14424800 -4.31780300

H 3.30779600 0.18957500 -4.05528900

H 3.37193600 2.14292600 -2.53215300

N -2.15306000 1.45830200 -1.21534500

C -3.10189100 2.10773600 -0.38676500

C -2.78626900 2.73971900 0.82847000

C -4.44317000 2.03335200 -0.79142200

C -3.80031300 3.29770500 1.60436000

H -1.76016400 2.79064000 1.16574800

C -5.44851900 2.59982700 -0.01186600

H -4.68648400 1.53359500 -1.72055100

C -5.13291500 3.23764300 1.18922100

H -3.54493300 3.78052600 2.54290100

H -6.47999400 2.53861700 -0.34493400

H -5.91586200 3.67841400 1.79852000

C -0.92204800 1.81935900 -1.33321700

O -0.40971100 2.92010800 -0.73981900

C 1.05655600 2.98653100 -0.92121800

C 1.40928900 4.38173100 -1.40871800

C 1.57643400 2.68950200 0.48822400

H 1.08488900 5.13083800 -0.68193000

H 2.49284500 4.46677000 -1.53939500

H 0.93350300 4.57980400 -2.37166800

H 1.38242300 3.52874100 1.15725400

H 1.12558000 1.78187900 0.88544300

I 3.76979700 2.29515200 0.65677300

C 3.34245700 0.21205800 1.04919000

C 3.23002600 -0.17949500 2.37977900

C 3.20407500 -0.65961000 -0.02641500

C 2.95806700 -1.52724400 2.63541800

H 3.34965700 0.52583600 3.19349700

C 2.93565300 -2.00172400 0.26070300

H 3.30251700 -0.32737600 -1.05095500

C 2.81380900 -2.43202700 1.58265200

H 2.86539200 -1.86104600 3.66378900

H 2.83365600 -2.70375100 -0.55896200

H 2.61074500 -3.47675000 1.79196400

O 6.03054600 1.37598900 0.95553100

H -2.71833900 0.34044400 -2.02630000

O -2.01107200 -2.48271400 -2.16549200

S -3.33580600 -1.84481800 -2.13026000

O -4.36568100 -2.49181400 -2.94899700

O -3.22438400 -0.35168300 -2.67012900

C -3.87260800 -1.60489000 -0.41195500

C -5.25917500 -1.57716200 -0.09841500

C -2.88660400 -1.33835500 0.58043900

C -5.61397800 -1.26723300 1.21977400

C -3.32973200 -1.04499800 1.87220200

C -4.67924200 -0.99854500 2.21472000

H -6.66353300 -1.24018800 1.48526000

H -2.59146700 -0.85196300 2.64024000

C -5.11745000 -0.70664300 3.63859600

H -6.21399600 -0.68593800 3.64284600

C -6.40055800 -1.84617700 -1.08501800

H -6.07090400 -2.62434100 -1.76874900

C -1.37150200 -1.42489800 0.37981500

H -1.13226200 -1.16756100 -0.64807800

C -7.68879000 -2.35260300 -0.41942100

H -8.37852400 -2.69147600 -1.19932200

H -8.20627600 -1.57300200 0.14973700

H -7.49886800 -3.19708100 0.25091600

C -6.70812500 -0.59235300 -1.92365900

H -7.51761800 -0.80312800 -2.63117000

H -5.83881900 -0.26590200 -2.49651200

H -7.02852500 0.23274200 -1.27818400

C -0.56585900 -0.46984200 1.27425200

H -0.54189700 -0.79314800 2.31973200

H -0.95897300 0.54927400 1.24537500

H 0.46918500 -0.45265000 0.92292000

C -0.90542100 -2.87361200 0.61651400

H 0.16639100 -2.96346700 0.41404500

H -1.43632100 -3.57025500 -0.03403300

H -1.07966200 -3.16470600 1.65889100

C -4.61703100 0.66309800 4.12089100

H -4.96754000 0.86039600 5.13986300

H -4.97705600 1.46465300 3.47073900

H -3.52230900 0.70540100 4.12945500

C -4.66324400 -1.82662600 4.59000200

H -5.03402600 -1.64294500 5.60428600

H -3.57009600 -1.88303700 4.63720700

H -5.03613200 -2.80202200 4.26099700

Li 6.10845100 -0.38426300 0.68273600

Cl 6.62117100 -2.78035300 0.00535400

O 5.61439000 -3.83687000 -0.23119200

O 7.99624500 -3.28806400 -0.19392400

O 6.36337000 -1.58979200 -0.90853200

O 6.47329000 -2.21020500 1.41036900

H 6.60253100 1.89605900 0.37305700

Zero-point correction= 0.753732 (Hartree/Particle)

Thermal correction to Energy= 0.826172

Thermal correction to Enthalpy= 0.827291

Thermal correction to Gibbs Free Energy= 0.632576

SCF Done: E(RM062X) = -3331.36226314

**IM3-6**

C 1.73916000 -0.00849200 -1.73000600

C 2.64510300 1.02896100 -1.50611800

C 2.52977900 2.25554800 -2.15871400

C 1.47915500 2.41604800 -3.06205600

C 0.58410400 1.36602000 -3.31090100

C 0.70744600 0.13924400 -2.65012300

H 3.24492100 3.04903500 -1.96913400

H 1.35861100 3.35793700 -3.58775500

H -0.21790200 1.50552200 -4.02889600

H 0.01465700 -0.66774800 -2.86130500

N 4.62763900 1.26081600 -0.10348200

C 5.60732000 0.80952600 0.79454700

C 5.95481000 -0.53602400 1.03350700

C 6.32164400 1.81663200 1.47079400

C 6.97699000 -0.84680400 1.92980900

H 5.43317700 -1.32887000 0.51459700

C 7.33015300 1.49726000 2.37570000

H 6.06074100 2.85118500 1.26982400

C 7.66468300 0.16074200 2.61039500

H 7.23572000 -1.88855500 2.09832500

H 7.86080900 2.29187100 2.89229000

H 8.45512400 -0.09229100 3.31056100

C 3.66038400 0.56370300 -0.55879400

O 3.38714000 -0.74469300 -0.24795400

C 2.11357900 -1.17819200 -0.84705100

C 2.36972100 -2.47536700 -1.59975800

C 1.20613500 -1.34684800 0.37198200

H 2.71057300 -3.25447800 -0.91198000

H 1.45554300 -2.81746200 -2.09364700

H 3.13022500 -2.31651200 -2.36758300

H 1.58315600 -2.13370200 1.02744800

H 1.10830100 -0.41460300 0.92736000

I -0.89177900 -1.98267700 -0.05362200

C -1.58099000 0.06835300 -0.04959100

C -1.36930900 0.82671500 1.09420300

C -2.23385400 0.54307000 -1.18284500

C -1.83447500 2.14537100 1.09007900

H -0.87291100 0.42084600 1.96818000

C -2.70048100 1.86082400 -1.15331100

H -2.38639100 -0.08081800 -2.05461300

C -2.49912300 2.65739000 -0.02458400

H -1.68735800 2.75910300 1.97273500

H -3.21802700 2.25626800 -2.02106400

H -2.86801400 3.67722200 -0.01164800

O -3.31842900 -2.27247700 -0.46955000

H -3.52383800 -3.13963700 -0.09260800

Li -4.29726500 -0.92213900 0.15160900

Cl -5.89174900 0.76902100 1.18191800

O -7.18050400 0.40172900 1.80907700

O -4.74929400 0.02413000 1.85974800

O -5.65958300 2.22811300 1.23568400

O -5.85262800 0.27980600 -0.26015000

Zero-point correction= 0.382390 (Hartree/Particle)

Thermal correction to Energy= 0.412768

Thermal correction to Enthalpy= 0.413712

Thermal correction to Gibbs Free Energy= 0.316168

SCF Done: E(RM062X) = -2121.50205139

**S4**

O 3.03316900 0.26613400 1.09822700

S 2.39281400 -0.54174500 0.05422000

O 2.67376900 -1.97884700 0.07895800

C 0.64439600 -0.13815200 -0.05041900

C -0.34675300 -1.15358700 -0.10594700

C 0.30719400 1.23738300 -0.02083100

C -1.67969400 -0.73855400 -0.05449200

C -1.05081800 1.56640400 0.02054400

C -2.05532400 0.60319300 0.02105500

H -2.45472700 -1.49439000 -0.07731300

H -1.33217900 2.61352100 0.05618000

C -3.51811700 1.00238000 0.08532000

H -3.55298500 2.09715200 0.13589900

C -0.07895500 -2.65674300 -0.18069700

H 0.87581700 -2.81214500 -0.67689300

C 1.29635800 2.40358100 -0.03740000

H 2.30359400 2.02926500 -0.19190000

C 0.02780000 -3.24470500 1.23760000

H 0.25337900 -4.31543900 1.18921100

H -0.91727300 -3.12012000 1.77821200

H 0.81875000 -2.75854400 1.81378700

C -1.13302100 -3.41890100 -0.99839000

H -2.10024900 -3.48067700 -0.48999100

H -0.78679000 -4.44541300 -1.15610700

H -1.29179700 -2.96137100 -1.98005800

C 1.00454000 3.36537600 -1.20098400

H 0.03895400 3.86889100 -1.09314400

H 1.00668900 2.83542500 -2.15873400

H 1.77812800 4.13956000 -1.23968500

C 1.28905100 3.13408200 1.31437700

H 2.02055700 3.94923000 1.30628400

H 1.54971200 2.44949300 2.12632000

H 0.30621000 3.56588600 1.53089100

C -4.19208200 0.44786900 1.35045000

H -5.23048700 0.79039300 1.41129300

H -3.67004300 0.77664300 2.25467700

H -4.20034400 -0.64740900 1.34528600

C -4.27277000 0.56254800 -1.17953100

H -5.31166000 0.90709900 -1.14337400

H -4.28494900 -0.52864700 -1.27316600

H -3.80784000 0.97293700 -2.08161200

O 2.84795000 0.02586300 -1.42056500

H 3.81457600 -0.10166300 -1.51047600

Zero-point correction= 0.368806 (Hartree/Particle)

Thermal correction to Energy= 0.398333

Thermal correction to Enthalpy= 0.399451

Thermal correction to Gibbs Free Energy= 0.304239

SCF Done: E(RM062X) = -1209.83323198

**TS3-3**

C -2.14655000 2.47062800 0.23927500

C -3.46352700 2.28626700 -0.23548000

C -4.20371700 3.36541500 -0.69269900

C -3.59978000 4.62779600 -0.66772400

C -2.29377000 4.81535700 -0.18348200

C -1.54364300 3.73824200 0.27688600

H -5.22297300 3.23033500 -1.03658700

H -4.15917500 5.48860800 -1.01945600

H -1.86576500 5.81154500 -0.17154700

H -0.52876600 3.86498800 0.63686200

N -4.95400700 0.37577800 -0.40608700

C -5.35582100 -0.95780600 -0.26393000

C -4.63747900 -1.98178400 0.39013500

C -6.60385800 -1.26552800 -0.83930600

C -5.16662200 -3.26923400 0.45250000

H -3.68206000 -1.77160800 0.85001800

C -7.12105400 -2.55531400 -0.77603400

H -7.14776700 -0.46841300 -1.33591200

C -6.40281800 -3.56434700 -0.12865500

H -4.60644300 -4.04905700 0.95993400

H -8.08325800 -2.77384700 -1.22915900

H -6.80259400 -4.57239300 -0.07602000

C -3.84960000 0.88917000 -0.04527600

O -2.80308600 0.23785900 0.59305600

C -1.66707900 1.09125300 0.73847100

C -1.02179700 0.97712200 2.09395200

C -0.89240500 1.18163300 -0.48564700

H -0.71492100 -0.05602700 2.26861100

H -0.14080200 1.61959700 2.14546900

H -1.73747900 1.27489400 2.86247300

H -1.32696200 0.89271200 -1.43505000

H 0.05108300 1.71307300 -0.48724800

I 0.71304700 -1.29602300 -0.60219300

C 1.91424700 -0.49179000 1.00061900

C 2.53451400 0.74142700 0.81652200

C 2.02943200 -1.22322400 2.18138000

C 3.29501000 1.26171000 1.86869300

H 2.46690400 1.27434100 -0.12313200

C 2.79543800 -0.68295900 3.21818200

H 1.54633500 -2.18569600 2.29764300

C 3.42166500 0.55630200 3.06551800

H 3.80261200 2.21022300 1.73099800

H 2.89855300 -1.24003500 4.14396800

H 4.01690700 0.96557700 3.87539900

O 4.14676700 -3.37433400 0.16497700

H 3.81477900 -3.86342700 -0.59760000

Li 4.27397200 -1.67350000 -0.03784400

Cl 5.41392100 0.36216600 -1.18751100

O 6.52002500 0.23129700 -2.16388100

O 4.19467400 -0.40530100 -1.66682800

O 5.06262000 1.78845100 -0.97256700

O 5.79463400 -0.29108900 0.12737000

Zero-point correction= 0.379308 (Hartree/Particle)

Thermal correction to Energy= 0.422515

Thermal correction to Enthalpy= 0.423634

Thermal correction to Gibbs Free Energy= 0.288629

SCF Done: E(RM062X) = -2121.45503425

**OH-LiClO_4_**

O -3.48656500 -0.01696300 -0.09297000

H -3.96301700 0.14595300 0.72975700

Li -1.77910400 -0.01404300 -0.03566200

Cl 0.78889000 0.00084300 0.00304300

O 1.62086300 1.21110400 -0.19236000

O -0.15601600 0.19794500 1.17765600

O 1.63001900 -1.19944000 0.21955000

O -0.12215200 -0.20741600 -1.19618800

Zero-point correction= 0.028192 (Hartree/Particle)

Thermal correction to Energy= 0.039241

Thermal correction to Enthalpy= 0.040359

Thermal correction to Gibbs Free Energy= -0.014569

SCF Done: E(RM062X) = -844.376451986

**PhI(DCE)**

C -0.00000100 1.20831500 -2.66388700

C 0.00000000 0.00000000 -3.36257100

C 0.00000100 -1.20831500 -2.66388700

C 0.00000000 -1.21924500 -1.26602200

C 0.00000000 0.00000000 -0.59300200

C 0.00000000 1.21924500 -1.26602200

H -0.00000600 2.15221600 -3.20090500

H 0.00000000 0.00000000 -4.44826000

H 0.00000600 -2.15221600 -3.20090500

H -0.00000800 -2.15907800 -0.72579500

H 0.00000800 2.15907800 -0.72579500

I 0.00000000 0.00000000 1.56969800

Zero-point correction= 0.090179 (Hartree/Particle)

Thermal correction to Energy= 0.098145

Thermal correction to Enthalpy= 0.099263

Thermal correction to Gibbs Free Energy= 0.051861

SCF Done: E(RM062X) = -529.252543950

**IM3-7**

C 2.65658000 0.34457400 -0.08287400

C 1.64431200 -0.62114400 0.02964200

C 1.97501400 -1.97639900 0.20238800

C 3.30725400 -2.35883100 0.25951900

C 4.31758100 -1.39576100 0.15210100

C 3.99274900 -0.05239500 -0.01692200

H 1.17825000 -2.70548000 0.29033500

H 3.56290500 -3.40477300 0.39075900

H 5.35944700 -1.69474600 0.20088800

H 4.77521700 0.69495000 -0.10256400

N -0.75569700 -0.99646500 -0.00189500

C -2.11975300 -0.67539800 0.03196300

C -2.98038500 -1.53317800 -0.67092400

C -2.65378200 0.38935000 0.77911600

C -4.35222700 -1.29759700 -0.67237900

H -2.55403400 -2.36684900 -1.21871800

C -4.03041000 0.59690200 0.79385800

H -2.00188400 1.02648900 1.36415100

C -4.88215600 -0.23350800 0.06102600

H -5.00901200 -1.95457900 -1.23358800

H -4.43948900 1.41203200 1.38254600

H -5.95335300 -0.05951600 0.07283500

C 0.24119400 -0.23894900 -0.01680000

O -0.01938700 1.21551800 -0.08491800

C 0.87954100 2.11712600 -0.19280100

C 0.39568600 3.50590800 -0.19504100

C 2.30835600 1.78674800 -0.30061900

H 0.51243900 3.88192900 0.83302300

H 1.02076400 4.12981500 -0.83799600

H -0.65769300 3.56025000 -0.47131700

H 2.85681100 2.46504700 0.36724000

H 2.59727700 2.12186000 -1.31250800

Zero-point correction= 0.258661 (Hartree/Particle)

Thermal correction to Energy= 0.279046

Thermal correction to Enthalpy= 0.280165

Thermal correction to Gibbs Free Energy= 0.204304

SCF Done: E(RM062X) = -747.853969624

**H_2_O-LiClO_4_**

Li -1.64395900 -0.00003200 0.04769300

Cl 0.87500600 0.00000100 -0.00277100

O 1.70159600 1.22352900 -0.01927100

O -0.04541800 -0.00059600 1.21681000

O 1.70164400 -1.22348000 -0.02042100

O -0.09438800 0.00055000 -1.18402200

O -3.50047700 0.00001400 0.04802800

H -4.02341400 0.77453100 -0.21264100

H -4.02346300 -0.77457800 -0.21231800

Zero-point correction= 0.041213 (Hartree/Particle)

Thermal correction to Energy= 0.052657

Thermal correction to Enthalpy= 0.053775

Thermal correction to Gibbs Free Energy= -0.001193

SCF Done: E(RM062X) = -844.866900494

**3a**

C 2.66174800 0.40630600 0.04184800

C 1.65107200 -0.57951800 -0.03945700

C 1.99705200 -1.93418200 -0.15917000

C 3.33190200 -2.31298100 -0.19816200

C 4.33910000 -1.33928800 -0.11772000

C 4.00875800 0.00395300 0.00094400

H 1.20399100 -2.67055600 -0.21867900

H 3.59528500 -3.36167000 -0.29067900

H 5.38274400 -1.63708200 -0.14815500

H 4.78618400 0.75936400 0.06389800

N -0.71486500 -1.01797600 -0.03666700

C -2.07572400 -0.67938400 -0.04721800

C -2.62705800 0.37832800 -0.79589700

C -2.94595500 -1.51852100 0.67131700

C -4.00455600 0.58946400 -0.80530900

H -1.97650200 1.02218800 -1.37530300

C -4.31966700 -1.28874100 0.67300100

H -2.51996800 -2.34662400 1.22945800

C -4.85852400 -0.23308700 -0.06645800

H -4.41288300 1.40623800 -1.39393100

H -4.97168500 -1.94214100 1.24557600

H -5.92993800 -0.05795000 -0.07319900

C 0.24534500 -0.17173500 0.00247500

O -0.01706200 1.17659300 0.11540200

C 0.97082800 2.13025100 0.19731800

C 0.39388000 3.49705800 0.31548500

C 2.27203000 1.79181200 0.16502900

H -0.24928600 3.56687900 1.19990000

H 1.18928700 4.24011400 0.39429400

H -0.22520000 3.73008200 -0.55842300

H 3.02713100 2.56583900 0.23053100

Zero-point correction= 0.248064 (Hartree/Particle)

Thermal correction to Energy= 0.267741

Thermal correction to Enthalpy= 0.268860

Thermal correction to Gibbs Free Energy= 0.194799

SCF Done: E(RM062X) = -747.455191412

**V. References**

1. Frisch, M. J., Trucks, G.W., Schlegel, H. B., Scuseria, G. E., Robb, M. A., Cheeseman, J. R., Scalmani, G., Barone, V., Petersson, G. A., Nakatsuji, H., Li, X., Caricato, M., Marenich, A. V., Bloino, J., Janesko, B. G., Gomperts, R., Mennucci, B., Hratchian, H. P., Ortiz, J. V., Izmaylov, A. F., Sonnenberg, J. L., Williams-Young, D., Ding, F., Lipparini, F., Egidi, F., Goings, J., Peng, B., Petrone, A., Henderson, T., Ranasinghe, D., Zakrzewski, V. G., Gao, J., Rega, N., Zheng, G., Liang, W., Hada, M., Ehara, M., Toyota, K., Fukuda, R., Hasegawa, J., Ishida, M., Nakajima, T., Honda, Y., Kitao, O., Nakai, H., Vreven, T., Throssell, K., Montgomery, Jr., J. A., Peralta, J. E., Ogliaro, F., Bearpark, M. J., Heyd, J. J., Brothers, E. N., Kudin, K. N., Staroverov, V. N., Keith, T. A., Kobayashi, R., Normand, J., Raghavachari, K., Rendell, A. P., Burant, J. C., Iyengar, S. S., Tomasi, J., Cossi, M., Millam, J. M., Klene, M., Adamo, C., Cammi, R., Ochterski, J. W., Martin, R. L., Morokuma, K., Farkas, O., Foresman, J. B. & Fox, D.J. Gaussian 16, Revision A. 03. Gaussian, Inc., Wallingford CT, (2016).

2. Stephens, P. J., Devlin, F. J., Chabalowski, C. F. & Frisch, M. J. Ab Initio Calculation of Vibrational Absorption and Circular Dichroism Spectra Using Density Functional Force Fields. *J. Phys. Chem*. **98**, 11623-11627,(1994).

3. Grimme, S., Antony, J., Ehrlich, S. & Krieg, H. A consistent and accurate ab initio parametrization of density functional dispersion correction (DFT-D) for the 94 elements H-Pu. *J. Chem. Phys.* **132**, 154104*,* (2010).

4. Grimme, S., Ehrlich, S. & Goerigk, L. Effect of the damping function in dispersion corrected density functional theory. *J. Comput. Chem*. **32**, 1456-1465, (2011).

5. Igel-Mann, G., Stoll, H. & Preuss, H. Pseudopotentials for main group elements (IIIa through VIIa). *Mol. Phys.* **65**, 1321−1328*,* (1988).

6. Andrae, D., Hȁußermann, U., Dolg, M., Stoll, H. & Preuß, H. Energy-adjusted ab initio pseudopotentials for the second and third row transition elements. *Theor. Chim. Acta.* **77**, 123-141, (1990).

7. Hariharan, P. C. & Pople, J. A. The influence of polarization functions on molecular orbital hydrogenation energies. *Theor. Chim. Acta.* **28**, 213-222, (1973).

8. Marenich, V., Cramer, C. J. & Truhlar, D. G. Universal Solvation Model Based on Solute Electron Density and on a Continuum Model of the Solvent Defined by the Bulk Dielectric Constant and Atomic Surface Tensions. *J. Phys. Chem. B.* **113**, 6378–6396, (2009).

9. Engelage, E., Schulz, N., Heinen, F., Huber, S. M., Truhlar, D. G. & Cramer, C. J. Refined SMD Parameters for Bromine and Iodine Accurately Model Halogen-Bonding Interactions in Solution. *Chem. Eur. J.* **24**, 15983-15987*,* (2018).

10. Fukui, K. The path of chemical reactions - the IRC approach. *Acc. Chem. Res.* **14**, 363–368, (1981).

11. Fukui, K. Formulation of the reaction coordinate*.* Formulation of the reaction coordinate. *J. Phys. Chem.* **74**, 4161–4163, (1970).

12. Zhao, Y. & Truhlar, D. G. The M06 suite of density functionals for main group thermochemistry, thermochemical kinetics, noncovalent interactions, excited states, and transition elements: two new functionals and systematic testing of four M06-class functionals and 12 other functionals. *Theor. Chim. Acta.*, **120**, 215-241, (2008).

13. McLean, A. D. & Chandler, G. S. Contracted Gaussian basis sets for molecular calculations. I. Second row atoms, Z=11–18. *J. Chem. Phys*. **72**, 5639-5648, (1980).

14. Krishnan, R., Binkley, J. S., Seeger, R. & Pople, J. A. Self‐consistent molecular orbital methods. XX. A basis set for correlated wave functions. *J. Chem. Phys.* **72**, 650-654, (1980).

15. Petersson, G. A., Bennett, A., Tensfeldt, T. G., Al-Laham, M. A. & Shirley, W. A. A complete basis set model chemistry. I. The total energies of closed‐shell atoms and hydrides of the first‐row elements. *J. Chem. Phys.* **89**, 2193-2218, (1988).

16. Frisch, M. J., Pople, J. A. & Binkley, J. S. Self‐consistent molecular orbital methods 25. Supplementary functions for Gaussian basis sets. *J. Chem. Phys.* **80**, 3265-3269, (1984).

17. Weigend, F. & Ahlrichs, R. Balanced basis sets of split valence, triple zeta valence and quadruple zeta valence quality for H to Rn: Design and assessment of accuracy. *Phys. Chem. Chem. Phys*. **7**, 3297-3305, (2005).

18. Sun, T.-Y., Chen, K., Zhou, H., You, T., Yin, P. & Wang, X. Revisiting the effect of f-functions in predicting the right reaction mechanism for hypervalent iodine reagents. *J. Comput. Chem.* **42**, 470-474, (2021).

19. CYLview, 1.0b; C. Y. Legault, Université de Sherbrooke: Quebec, Canada, (2009) (<http://www.cylview.org).>

20. Wang, B., Rong, C., Chattaraj, P. K. & Liu, S. A comparative study to predict regioselectivity, electrophilicity and nucleophilicity with Fukui function and Hirshfeld charge. *Theor. Chem. Acc.* **138**: 124, (2019).

21. Lu, T., Chen, Q. *Conceptual Density Functional Theory: Towards a New Chemical Reactivity Theory* *Ch. 31* (WILEY-VCH, Weinheim, Germany, 2022).
